# Desymmetrization of unactivated bis-alkenes *via* chiral Brønsted acid-catalysed hydroamination†

**DOI:** 10.1039/d0sc00001a

**Published:** 2020-05-20

**Authors:** Zhang-Long Yu, Yong-Feng Cheng, Na-Chuan Jiang, Jian Wang, Li-Wen Fan, Yue Yuan, Zhong-Liang Li, Qiang-Shuai Gu, Xin-Yuan Liu

**Affiliations:** Shenzhen Grubbs Institute, Department of Chemistry, Guangdong Provincial Key Laboratory of Catalysis, Southern University of Science and Technology Shenzhen 518055 China liuxy3@sustech.edu.cn; Academy for Advanced Interdisciplinary Studies, Southern University of Science and Technology Shenzhen 518055 China guqs@sustech.edu.cn; Shenzhen Key Laboratory of Small Molecule Drug Discovery and Synthesis, Department of Chemistry, Southern University of Science and Technology Shenzhen 518055 China

## Abstract

Although great success has been achieved in catalytic asymmetric hydroamination of unactivated alkenes using transition metal catalysis and organocatalysis, the development of catalytic desymmetrising hydroamination of such alkenes remains a tough challenge in terms of attaining a high level of stereocontrol over both remote sites and reaction centers at the same time. To address this problem, here we report a highly efficient and practical desymmetrising hydroamination of unactivated alkenes catalysed by chiral Brønsted acids with both high diastereoselectivity and enantioselectivity. This method features a remarkably broad alkene scope, ranging from mono-substituted and *gem*-/1,2-disubstituted to the challenging tri- and tetra-substituted alkenes, to provide access to a variety of diversely functionalized chiral pyrrolidines bearing two congested tertiary or quaternary stereocenters with excellent efficiency under mild and user-friendly synthetic conditions. The key to success is indirect activation of unactivated alkenes by chiral Brønsted acids *via* a concerted hydroamination mechanism.

## Abstract 2

Highly stereoselective desymmetrising hydroamination of various unactivated alkenes has been achieved using chiral Brønsted acid catalysis *via* a concerted mechanism.

## Graphical abstract

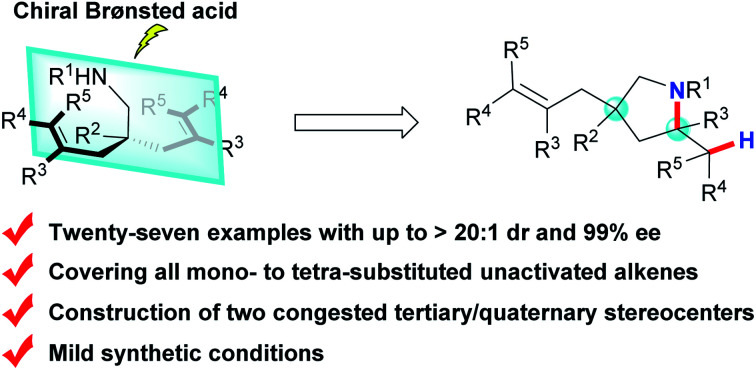

## Introduction

**Scheme 1:**
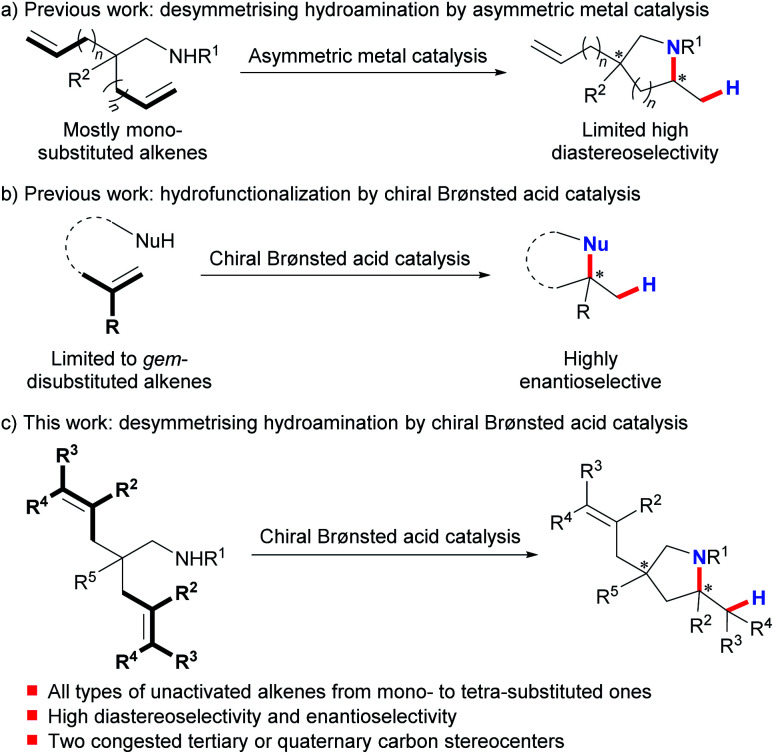
Desymmetrising hydroamination by asymmetric metal catalysis and chiral Brønsted acid catalysis.

The desymmetrization of prochiral or *meso* compounds represents a powerful tool for the preparation of complex chiral organic molecules with congested tertiary and quaternary stereocenters, which are ubiquitous in many natural products and biologically relevant molecules.^1^ This field has been extensively investigated using transition metal catalysis^2^ and organocatalysis.^3^ On the other hand, much effort has been devoted over the past decades to the development of asymmetric hydroamination of alkenes as an efficient way to prepare enantioenriched amines.^4^ In sharp contrast, desymmetrising hydroamination has been reported under metal catalysis with poorly controlled diastereoselectivity.^5–7^ And only recently have Sadow *et al.* successfully achieved highly stereoselective desymmetrising hydroamination of two mono-substituted alkene substrates using their developed chiral zirconium complex (Scheme 1a).^8^ Although of limited functional group compatibility due to the presence of the highly air- and moisture-sensitive early-transition-metal catalyst, this work represents the state-of-the-art in this field. Nonetheless, the limited number of highly stereoselective examples (≥10 : 1 dr, ≥90% ee) as well as the confined alkene scope highlights the challenge in attaining a high level of stereocontrol over both remote sites and reaction centers at the same time. Clearly, a conceptually different approach is very desirable to achieve a highly efficient and practical desymmetrising hydroamination with a broad alkene scope under mild synthetic conditions.

Chiral Brønsted acids have recently evolved into fundamentally significant tools for asymmetric catalysis.^9^ Nevertheless, activation of unactivated alkenes by chiral Brønsted acids for nucleophilic attack^10^ has been impeded by their inherently low basicity. And until recently, List's,^10*d*^ Terada's,^10*e*^ and our groups^10*c*^ have independently disclosed highly enantioselective hydrofunctionalization of minimally functionalized geminally disubstituted alkenes (Scheme 1b). This limited alkene scope emphasizes the remaining difficulty and also untapped space in applying chiral Brønsted acids for asymmetric hydrofunctionalization of unactivated alkenes. To this end, we herein describe the successful realization of desymmetrising hydroamination of unactivated alkenes *via* indirect activation of alkenes by chiral Brønsted acids through a concerted pathway under mild conditions (Scheme 1c).^11^ Advantageously, this process tolerates not only common mono-substituted and *gem*-/1,2-disubstituted unactivated alkenes but also the challenging tri- and tetra-substituted ones. Thus, it provides a straightforward access to diversely functionalized chiral pyrrolidines bearing two congested tertiary or quaternary stereocenters with both high diastereoselectivity and enantioselectivity. These enantioenriched pyrrolidines constitute the core structures of many biologically active natural products and pharmaceutical chemicals.^12^

## Results and discussion

### Desymmetrising hydroamination of unactivated alkenes

At the outset, we examined different N-protecting groups for their potential to efficiently participate in the desymmetrising hydroamination of geminally disubstituted alkenes in the presence of a chiral *N*-triflyl phosphoramide (*R*)-**A1** (Table 1).^13^ The *tert*-butoxycarbonyl-protected amine **1A** failed to undergo the desired hydroamination reaction, while the tosyl-protected one **1B** afforded the corresponding hydroamination product **3** with promising enantioselectivity albeit of low yield and diastereoselectivity. Fortunately, the use of an *N*-arylaminocarbonyl protecting group in **1C** resulted in remarkable improvements in both the yield and the diastereoselectivity, although the enantioselectivity was only moderate (65% ee). These encouraging results have prompted us to further investigate the thiocarbonyl analog **1D**, which delivered superior stereoselectivity as well as reactivity. The higher acidity of a thiourea compared with a urea may account for the improvements over reactivity and stereoselectivity by invoking stronger hydrogen bonding interactions with the chiral Brønsted acid.

**Table 1:** Evaluation of different protecting groups^a^

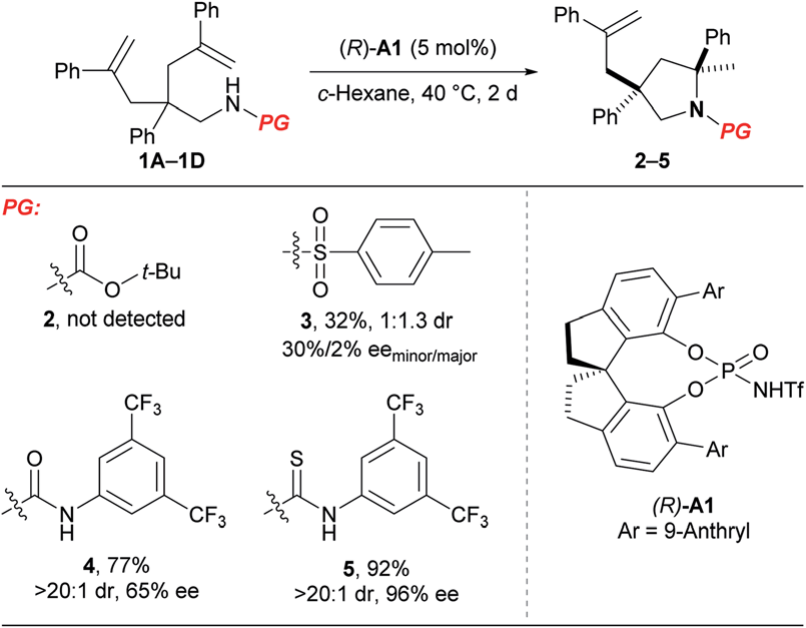

a Reactions were run on a 0.1 mmol scale at 40 °C; isolated yields were shown; dr and ee values were determined by ^1^H NMR and HPLC analysis, respectively.

With the optimal protecting group for amine in hand, we then carried out further condition optimization by screening other Brønsted acids, solvents, catalyst loadings, and reaction temperatures (Table 2). The results indicated that: (i) the reactivity and enantioselectivity were greatly affected primarily by chiral Brønsted acid catalysts while the diastereoselectivity remained excellent all the time (entries 1–8); (ii) more acidic chiral *N*-triflyl phosphoramides^13^ generally provided better enantiocontrol as well as reactivity compared with chiral phosphoric acids (entries 1, 5, and 6 *vs.* entries 2–4, 7, and 8); (iii) solvent significantly affected the reaction rate (entries 1 and 9–13) and *c*-hexane provided the highest yield within the shortest reaction time (entry 13). Although the reaction worked at room temperature, slight heating guaranteed high reactivity with a catalyst loading as low as 5 mol% (entries 13–16). Therefore, the optimal reaction conditions are as follows: 5 mol% (*R*)-**A1** in cyclohexane at 40 °C.

**Table 2:** Screening results of reaction conditions^a^

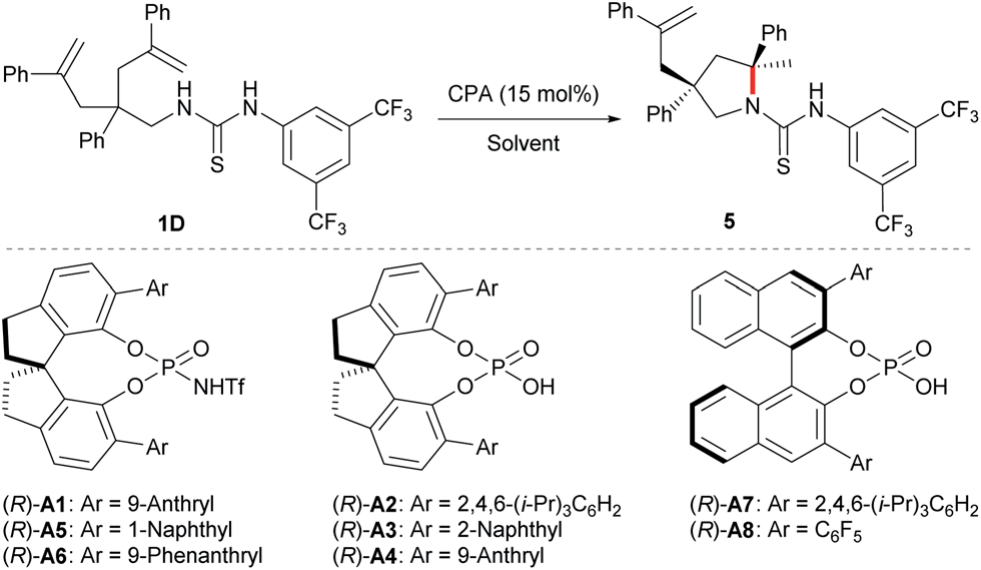						
Entry	Catalyst	Solvent	Time (h)	Yield (%)	Dr	Ee (%)
1	(*R*)-**A1**	CCl_4_	24	91	>20 : 1	97
2	(*R*)-**A2**	CCl_4_	72	12	>20 : 1	0
3	(*R*)-**A3**	CCl_4_	48	64	>20 : 1	43
4	(*R*)-**A4**	CCl_4_	72	68	>20 : 1	27
5	(*R*)-**A5**	CCl_4_	72	70	>20 : 1	90
6	(*R*)-**A6**	CCl_4_	72	78	>20 : 1	93
7	(*R*)-**A7**	CCl_4_	72	75	>20 : 1	70
8	(*R*)-**A8**	CCl_4_	48	82	>20 : 1	86
9	(*R*)-**A1**	CH_2_Cl_2_	72	85	>20 : 1	99
10	(*R*)-**A1**	CH_3_CN	72	88	>20 : 1	99
11	(*R*)-**A1**	EtOAc	17	88	>20 : 1	99
12	(*R*)-**A1**	THF	72	75	>20 : 1	99
13	(*R*)-**A1**	*c*-Hexane	4	92	>20 : 1	99
14	(*R*)-**A1**^b^	*c*-Hexane	24	92	>20 : 1	97
15	(*R*)-**A1**^c^	*c*-Hexane	96	82	>20 : 1	96
16^d^	(*R*)-**A1**^c^	*c*-Hexane	24	92	>20 : 1	96

a Reactions were run on a 0.025 mmol scale at room temperature; yield was determined by ^1^H NMR of the crude reaction residue; ee was determined by HPLC analysis on a chiral stationary phase.

b 10 mol% (*R*)-**A1**.

c 5 mol% (*R*)-**A1**.

d The reaction was run at 40 °C.

Next, we investigated the substrate scope of the current method (Table 3). Firstly, the *N*-aryl groups possessing electron-withdrawing (CF_3_ and Cl) substituents, a slightly electron-donating (Me) substituent, or no substituents on either the *para*- or *meta*-positions all provided high reaction efficiency and stereoselectivity (**5–9**). Secondly, for the aryl ring on the tether, various substituents of different electronic properties at the *ortho*-, *meta*-, or *para*-positions were all well tolerated, giving the expected products **10–12** as apparently single diastereoisomers in 79–90% yields with 91–96% ee. In addition, a reactive terminal triple bond appended on this aryl ring readily survived our reaction conditions to afford the corresponding product **13**. Further, a polar unprotected phenol group on the tether was compatible with the reaction conditions to deliver product **14**. Besides these monocyclic aryl rings, a bicyclic naphthalene ring was also suitable for this reaction to provide product **15**. Of particular note is that two substrates bearing relatively labile electron-rich heteroaryl rings on the tether both underwent the current reaction smoothly to deliver the desired products **16** and **17** with excellent results. Nonetheless, no reaction occurred for substrates bearing polar 3-pyridinyl or unprotected 3-indolyl groups on the tether (for structures, see Fig. S1†), possibly due to the disruption of necessary substrate–catalyst hydrogen bonding interactions. Interestingly, the deleterious effect caused by removing the aryl group (*R*^2^) on the tether (33% yield, 1.2 : 1 dr, 42% and 13% ee) was readily overcome mainly by facile switching the chiral Brønsted acid to (*R*)-**A2**. Accordingly, product **18** bearing one tertiary stereocenter and one quaternary stereocenter was forged in 75% yield with excellent stereoselectivity (11 : 1 dr, 92% ee) under modified conditions. Thirdly, for the aryl ring on the alkene moiety, both electron-rich and -deficient phenyl rings as well as a naphthalene ring all were well accommodated in this transformation, smoothly delivering the corresponding products **19–24**. Overall, the good compatibility with aryl halides, terminal alkynes, and electron-rich heterocycles, as mentioned above, as well as the remaining unreacted alkenes leaves a huge synthetic space for further derivatization.

**Table 3:** Substrate scope^a^

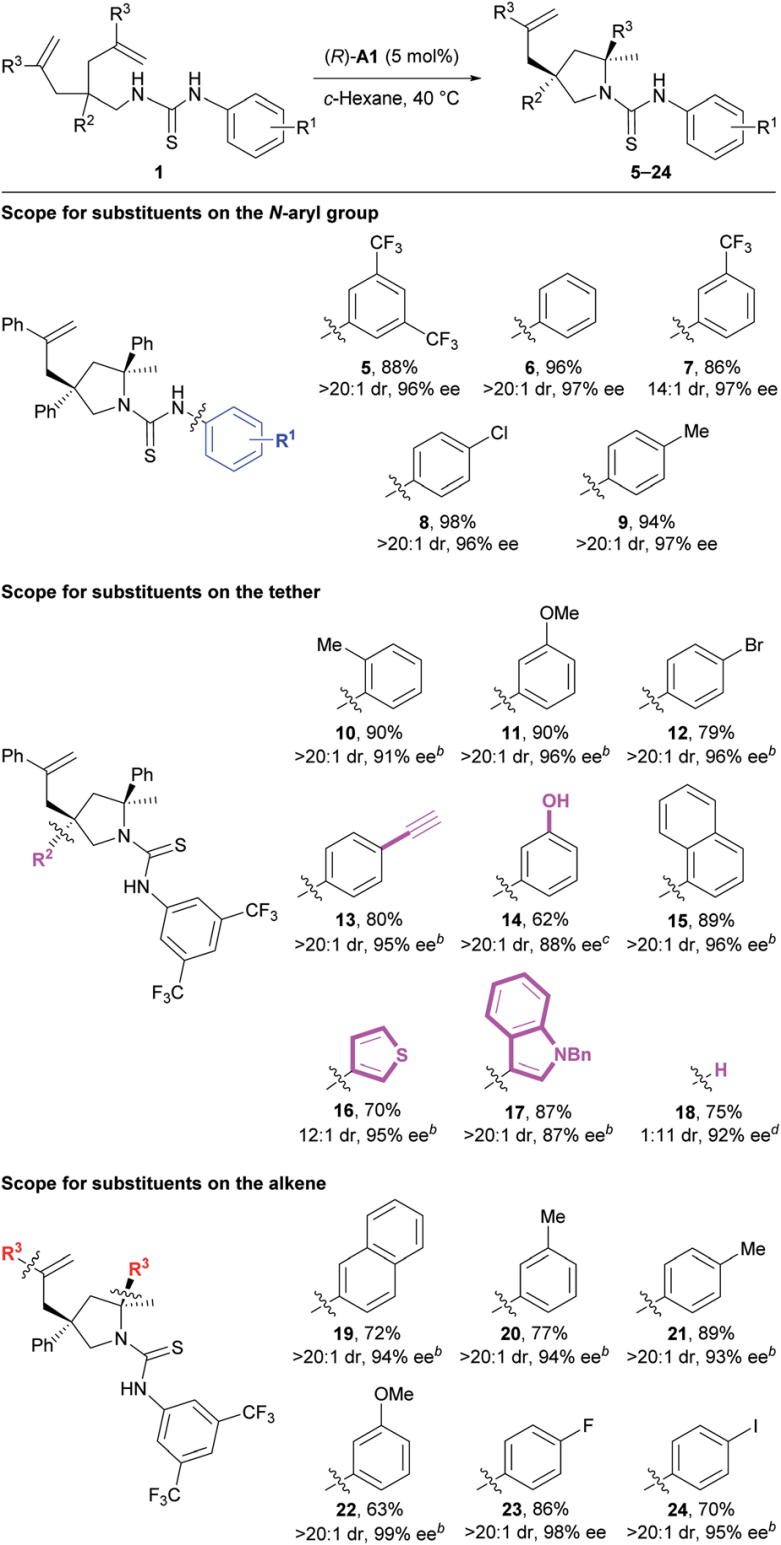

a Reactions were run on a 0.10 mmol scale at 40 °C; isolated yields were shown; dr and ee values were determined by ^1^H NMR and HPLC analysis, respectively.

b The reaction was run at 60 °C.

c The reaction was run using (*R*)-**A1** (15 mol%) in CCl_4_ at 60 °C on a 0.050 mmol scale.

d The reaction was run using (*R*)-**A2** (Table 1, 15 mol%) in 1,2-dichlorobenzene at 60 °C for 24 h; In the major diastereomer, the unreacted alkene side chain is *trans* toward the phenyl ring on the pyrrolidine ring of **18**; results under standard conditions shown above: 33%, 1 : 1.2 dr, 13% and 42% ee.

As discussed in the introduction, the application of common chiral Brønsted acids for activating unactivated alkenes towards nucleophilic attack *via* direct protonation has been prohibited by the fact that such alkenes are not basic enough. Given the above-mentioned robust catalytic activity for styrene-type alkenes, we were naturally eager to challenge our catalytic system by exploring other types of alkenes (Table 4). To our delight, substrates featuring aliphatic geminally disubstituted alkenes were also applicable for this reaction, and the desired products **25** and **26** were obtained with excellent yields and stereoselectivity. More encouragingly, a substrate bearing even-challenging monosubstituted alkenyl groups also underwent the current reaction smoothly, delivering the desired product **27** in 82% yield with 96% ee and larger than 20 : 1 dr. Encouraged by these results, we continued to examine less activated 1,2-disubstituted alkenes and obtained the desired product with excellent stereoselectivity (**28**, >20 : 1 dr, 95% ee), albeit in 25% yield, using 30 mol% catalyst (*R*)-**A1**. Most importantly, one major advantage of the current process over metal-catalysed ones would lie on efficient desymmetrising hydroamination of sterically demanding multisubstituted alkenes. In this regard, both tri- and tetra-substituted alkenes bearing electronically distinct alkyl and phenyl substituents all readily gave rise to products **29–31** in 75–86% yields with excellent enantio- and diastereoselectivity (Table 4). Of particular note is that such sterically demanding multisubstituted alkenes are generally problematic for stereoselective hydroamination, which has only been rarely investigated with poor enantiocontrol.^14^ On the whole, all these results clearly indicate a broad substrate scope and good functional group tolerance, highlighting the generality of this transformation.

**Table 4:** Scope for other unactivated alkenes^a^

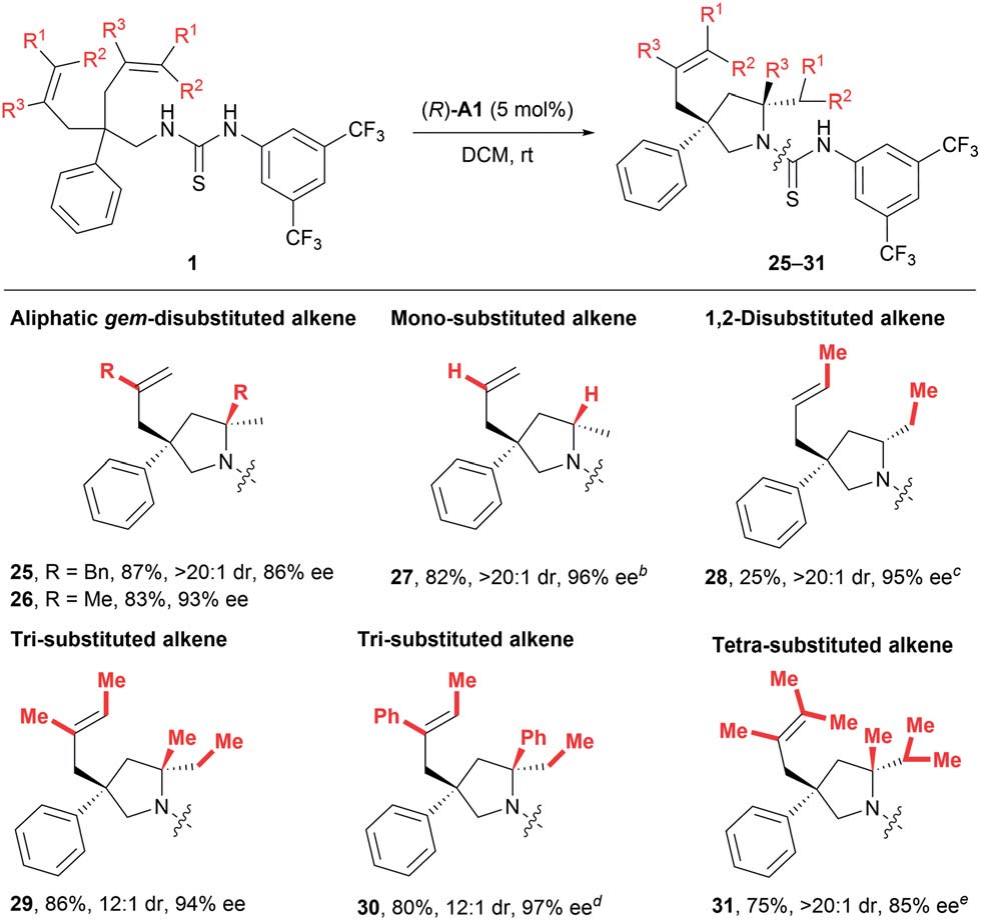

a Reactions were run on a 0.10 mmol scale at room temperature; isolated yields are shown; dr and ee values were determined by ^1^H NMR and HPLC analysis, respectively.

b The reaction was run in *c*-hexane and (*R*)-**A1** (15 mol%) was used.

c The reaction was run in *c*-hexane at 60 °C and (*R*)-**A1** (30 mol%) was used.

d (*R*)-**A1** (15 mol%) was used.

e (*R*)-**A1** (20 mol%) was used.

### Transformation and application

**Scheme 2:**
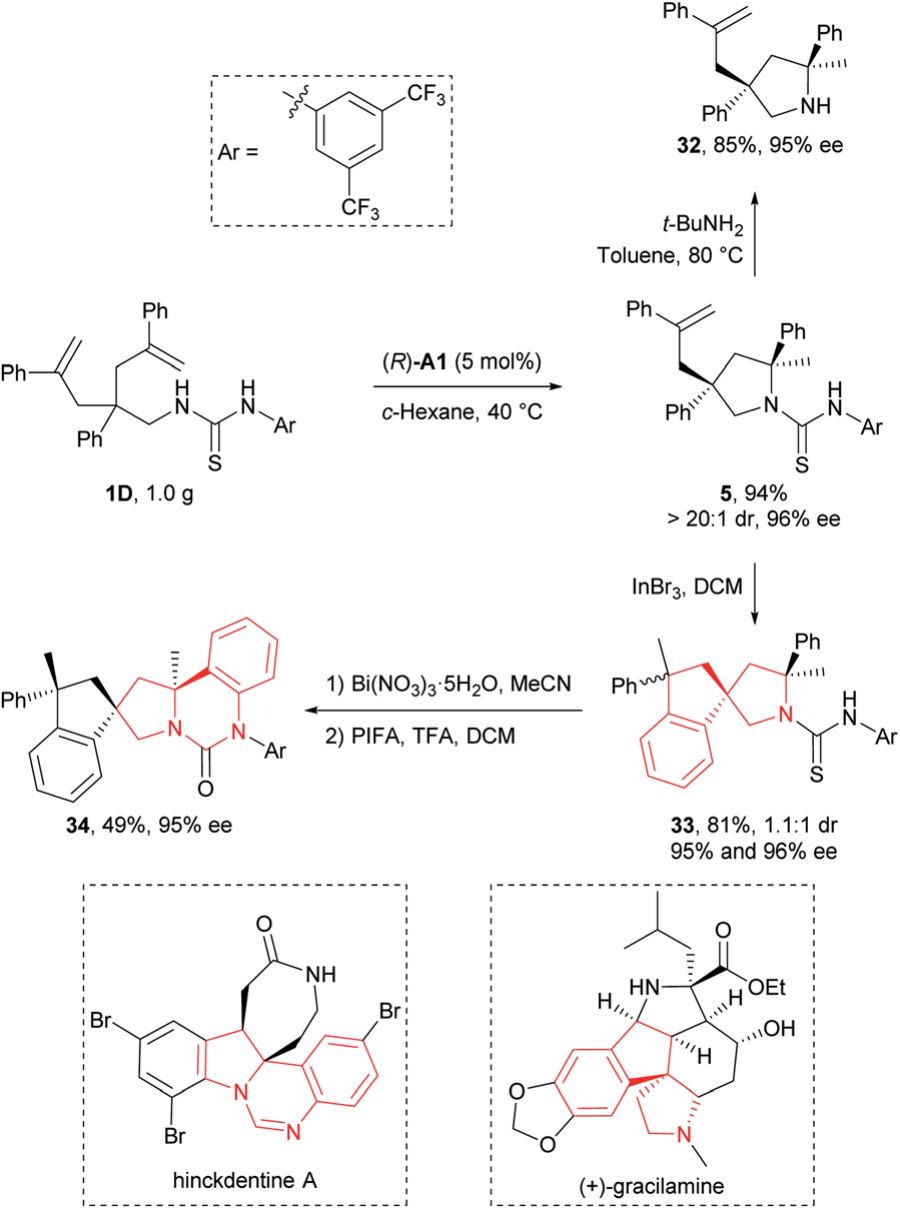
Representative product transformation and application.

To illustrate the synthetic applicability of this transformation, we performed a gram-scale preparation of **5** (Scheme 2), which showed no changes in both reaction efficiency and stereoselectivity. In addition, we have readily removed the *N*-arylaminothiocarbonyl group in product **5** by straightforward treatment with *tert*-butylamine at elevated temperature, furnishing free secondary amine **32** in 85% yield (Scheme 2). More importantly, this group also provided additional synthetic potential for further derivatization. For example, **5** underwent a hydroarylative cyclization under the catalysis of InBr_3_ to yield spirocyclic pyrrolidines **33** as a 1.1 : 1 mixture of diastereomers in 81% overall yield. Next, we converted the remaining thiocarbonyl in one diastereomer of **33** into carbonyl using Bi(NO_3_)_3_·5H_2_O, and further forged a new fused ring by subsequent smooth oxidation with [bis(trifluoroacetoxy)iodo]benzene (PIFA) (Scheme 2). Remarkably, the resulting polycyclic compound **34** and its precursor **33** possess the core structural elements of many biologically active natural alkaloids like gracilamine^15^ and hinckdentine A^16^ (Scheme 2). An important aspect of the transformations discussed above is that no significant enantiopurity erosion has ever occurred, setting a solid foundation for future applications in asymmetric synthesis of enantioenriched azaheterocycles.

### Mechanistic investigations

**Scheme 3:**
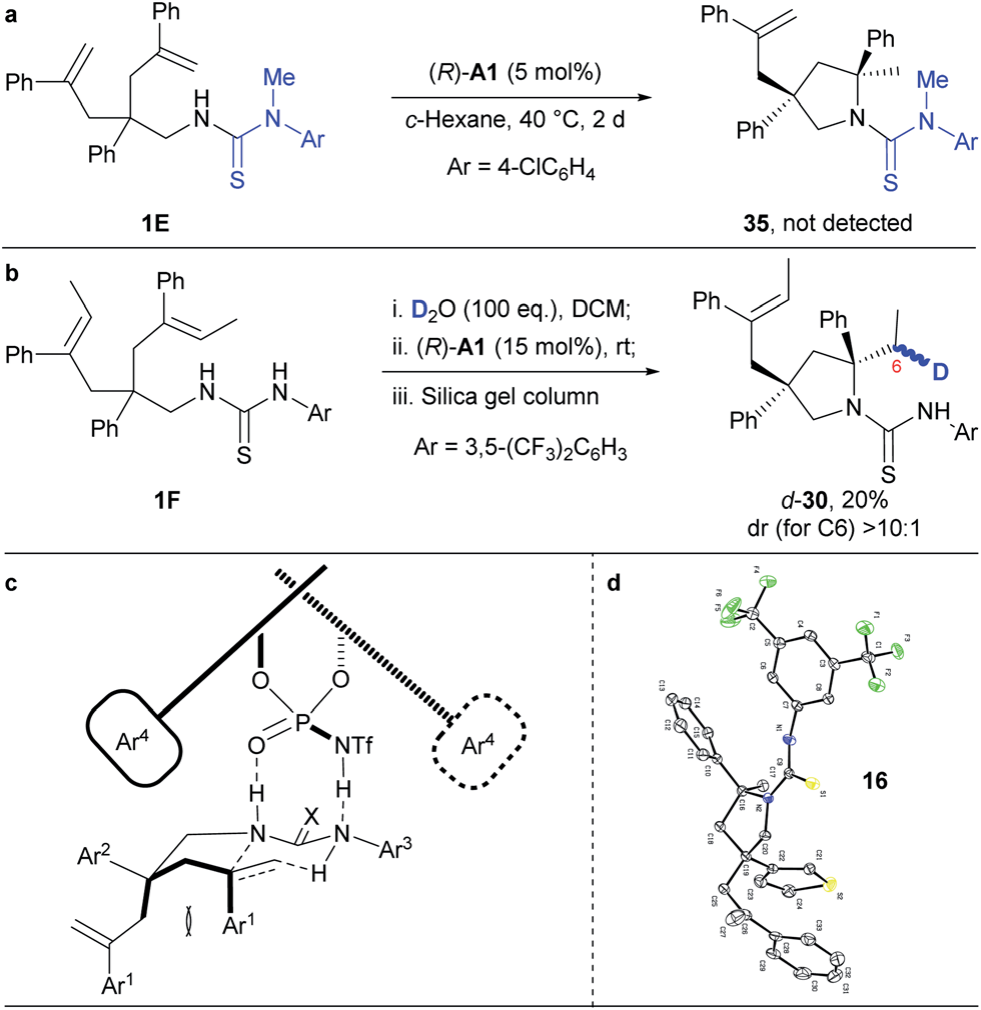
Mechanistic study.

To get insights into the reaction mechanism, we have conducted some control experiments (Scheme 3). When the N–H bond in the *N*-arylaminothiocarbonyl group was masked with a methyl group, the reaction failed to occur under the standard reaction conditions (Scheme 3a). This is consistent with a speculative multiple hydrogen bonding formation between phosphoric acid and the thiourea moiety, which may have prepared the substrate in an organized conformation for subsequent hydroamination. Furthermore, deuterated **1F** (Scheme 3b), prepared by overnight stirring with D_2_O, afforded *d*-**30** as an almost single diastereomer in regard to C6 under the otherwise standard conditions. This result strongly supports a concerted hydroamination pathway. On the basis of the above mechanistic investigations and previous studies,^5–7^ we propose that the desymmetrising hydroamination reaction proceeds through a concerted mechanism. Thus, the chiral phosphoramide indirectly activates alkenes through a hydrogen bonding relay with the N–H in the *N*-arylaminothiocarbonyl protecting group while its relatively basic oxygen deprotonates the protected amine by forming hydrogen bonding with the remaining N–H (Scheme 3c). This extensive hydrogen-bonding formation may have significantly lowered the original high barrier for direct protonation of unactivated alkenes other than the geminally disubstituted ones by a common chiral Brønsted acid. On the basis of the proposed mechanism and given the absolute configuration of **16** determined by X-ray crystallographic analysis (Scheme 3d and Fig. S2;† CCDC 1853110), we have accordingly set up a possible optimal transition state (Scheme 3c) that has led to the formation of most of our hydroamination products according to the Simón and Goodman's model.^17^ In this transition state, the si-face attack of the geminally disubstituted alkenes by protected amines was favored due to the steric hindrance between the Ar^1^ groups on the alkenes and the bulky 3,3′-substituents on the chiral phosphoramide catalyst during the re-face approaching. The *trans* configuration of the sterically demanding Ar^1^ and Ar^2^ groups alleviated their otherwise severe *pseudo* 1,3-diaxial interaction. As a proof of this model, the unreacted alkene side chain is *trans* to the Ar^1^ group in **18**, as determined by 2D NMR studies, where the steric interaction between the relative bulky side chain and the Ar^1^ group became predominant.

## Conclusion

In summary, chiral Brønsted acids have been successfully employed to realize highly diastereoselective and enantioselective desymmetrising hydroamination of unactivated alkenes. In contrast to traditional metal-catalysed desymmetrising hydroamination of unactivated alkenes, this protocol readily accommodates mono-substituted, *gem*-disubstituted, 1,2-disubstituted, tri-substituted, and tetra-substituted unactivated alkenes with good functional group tolerance. Thus, this mild reaction provides rapid access to a diverse range of enantioenriched pyrrolidines with multiple congested carbon stereocenters. Further studies including detailed investigation of the origin of stereocontrol and the development of desymmetrization of other types of alkenes with chiral Brønsted acids are currently underway.

## Conflicts of interest

There are no conflicts to declare.

## Supplementary Material

SC-011-D0SC00001A-s001

SC-011-D0SC00001A-s002
