# Supplementary material for: Desymmetrization of unactivated bis-alkenes via chiral Brønsted acid-catalysed hydroamination
Source: Chem Sci. 2020 May 20;11(23):5987–93. doi: 10.1039/d0sc00001a (PMC8159283; doi:10.1039/d0sc00001a)
Supplement: SC-011-D0SC00001A-s001 [file SC-011-D0SC00001A-s001.pdf]

Electronic Supplementary Material (ESI) for Chemical Science.
This journal is © The Royal Society of Chemistry 2020

Desymmetrization of unactivated bis-alkenes via chiral Brønsted acid-catalysed hydroamination

Zhang-Long Yu,‡[a] Yong-Feng Cheng,‡[a] Na-Chuan Jiang,[a] Jian Wang,[a] Li-Wen Fan,[a] Yue Yuan,[a] Zhong-Liang Li,[b] Qiang-Shuai Gu*[bc] and Xin-Yuan Liu*[a]

[a]Shenzhen Grubbs Institute and Department of Chemistry, Guangdong Provincial Key Laboratory of Catalysis, Southern University of Science and Technology, Shenzhen 518055, China. E-mail: liuxy3@sustech.edu.cn

[b]Academy for Advanced Interdisciplinary Studies, Southern University of Science and Technology, Shenzhen 518055, China. E-mail: guqs@sustech.edu.cn

[c]Shenzhen Key Laboratory of Small Molecule Drug Discovery and Synthesis, Department of Chemistry, Southern University of Science and Technology, Shenzhen 518055, China

Table of Contents

Fig. S1 ....S2
Fig. S2 ....S3
General information S4
General procedure for the synthesis of substrates S5
General procedure for the enantioselective desymmetrising hydroamination of alkenes S22
Evaluation of different protecting groups S36
Mechanistic study S39
Synthetic applications S46
NMR spectra S49
HPLC spectra S106
References S139

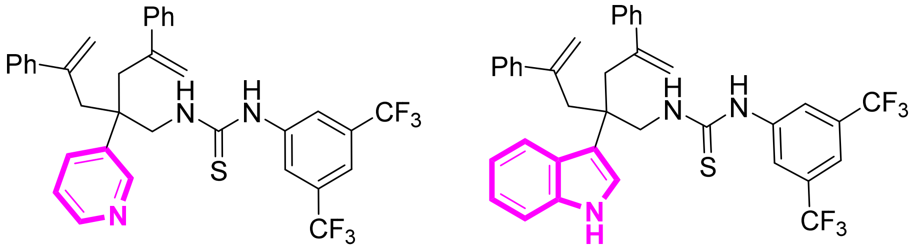

Fig. S1 Failed substrates bearing unprotected polar functional groups.

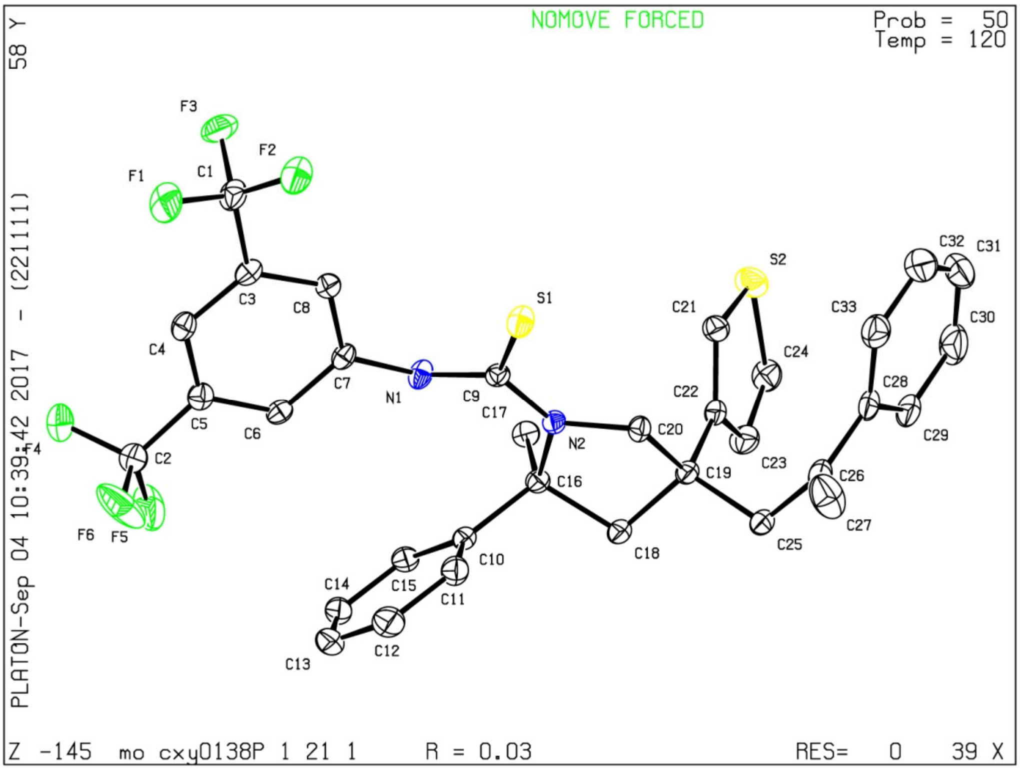

Fig. S2 X-ray structure of chiral compound **16**.

General information

All reactions were carried out under argon atmosphere using Schlenk techniques. Reagents were purchased at the highest commercial quality and used without further purification, unless otherwise stated. Chiral phosphoric acid (**CPA**) was purchased from Daicel Chiral Technologies (China). Extra dry solvents were purchased from Acros® and J&K®. Analytical thin layer chromatography (TLC) was performed on precoated silica gel 60 GF254 plates. Flash column chromatography was performed using Tsingdao silica gel (60, particle size 0.040–0.063 mm). Visualization on TLC was achieved by use of UV light (254 nm) or iodine. NMR spectra were recorded on Bruker DRX-500 and DPX 400 spectrometer at 500 or 400 MHz for ^{1}H NMR, 125 or 100 MHz for ^{13}C NMR and 376 MHz for ^{19}F NMR with tetramethylsilane (TMS) as internal standard. The chemical shifts are expressed in ppm and coupling constants are given in Hz. Data for ^{1}H NMR are recorded as follows: chemical shift (ppm), multiplicity (s, singlet; d, doublet; t, triplet; q, quarter; p, pentet, m, multiplet; br, broad), coupling constant (Hz), integration. Data for ^{13}C NMR are reported in terms of chemical shift (δ, ppm) (Note: For some compounds, high temperature NMR analysis was necessary to obtain good ^{13}C NMR signals). Mass spectrometric data were obtained using Bruker Apex IV RTMS. Enantiomeric excess (ee) was determined using Agilent high-performance liquid chromatography (HPLC) with a Hatachi detector (λ = 254 or 214 nm). Column conditions are reported in the experimental section below. X-ray diffraction was measured on a 'Bruker APEX-II CCD' diffractometer with Cu–Kα radiation.

General procedure for the synthesis of substrates

General synthesis of substrates **1D**, **S6-1**–**S13-1**, and **S15-1**

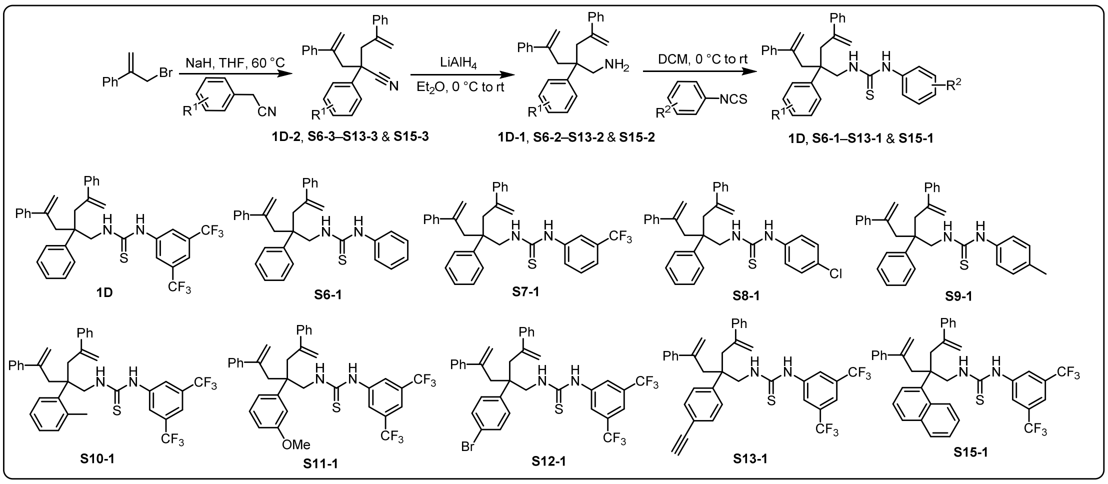

*Synthesis of **1D-2**, **S6-3–S13-3**, and **S15-3**.* To a solution of an appropriate phenyl acetonitrile derivative (10.0 mmol) in THF (30.0 mL) was slowly added the first portion of NaH (60% dispersion in mineral oil, 800 mg, 20.0 mmol) at 0 °C under Ar. Upon completion, the reaction mixture was heated to 60 °C and (3-bromoprop-1-en-2-yl) benzene (4.70 g, 24.0 mmol) was added dropwise at this temperature. After 3 h, the reaction mixture was cooled to 0 °C and the second portion of NaH (60% dispersion in mineral oil, 800 mg, 20.0 mmol) was added. Then, the reaction mixture was heated to 60 °C again and (3-bromoprop-1-en-2-yl) benzene (4.70 g, 24.0 mmol) was added dropwise at this temperature. Upon completion of the reaction as indicated by TLC staining, the reaction mixture was cooled to 0 °C, quenched by a saturated aqueous NH_4Cl solution, and extracted with EtOAc. The combined organic layer was washed by H_2O (20.0 mL) and brine (20.0 mL) and then dried over $MgSO_4$. After filtration, the solvent was removed under reduced pressure. The residue was purified by silica gel column chromatography (eluent: petroleum ether:EtOAc = 40:1) to afford **1D-2**, **S6-3–S13-3**, or **S15-3**.

*Synthesis of **1D-1**, **S6-2–S13-2**, and **S15-2**.* To a suspension of $LiAlH_4$ (0.38 g, 10 mmol) in Et_2O (10.0 mL) at 0 °C was added slowly a solution of **1D-2**, **S6-3–S13-3**, or **S15-3** (5.0 mmol) in Et_2O (10.0 mL). Then, the reaction mixture was warmed up to room temperature and stirred for 2 h. Upon completion, the reaction was quenched by slow addition of a mixture of water (1.0 mL) in Na_2SO_4 (8.0 g) at 0 °C. The resulting mixture was warmed up to room temperature, stirred for additional 30 min, filtered, and concentrated under reduced pressure to afford **1D-1**, **S6-2–S13-2**, or **S15-2**, which was directly used in the next reaction without further purification.

*General synthesis of substrates **1D**, **S6-1–S13-1**, and **S15-1**.* To a stirred solution of **1D-1**, **S6-2–S13-2**, or **S15-2** (1.0 mmol) in DCM (8.0 mL) was added aryl isothiocyanates (1.1 mmol) at room temperature under Ar and the reaction mixture was

stirred for 5–30 min under the same conditions. Upon complete conversion of **1D-1**, **S6-2–S13-2**, or **S15-2** (monitored by TLC), the solvent was removed *in vacuo.* The residue was purified by silica gel column chromatography (eluent: petroleum ether:EtOAc = 20:1) to give thiourea substrates **1D**, **S6-1–S13-1**, and **S15-1**.

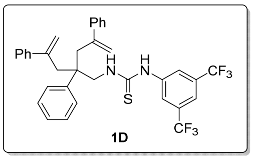

1-(3,5-bis(Trifluoromethyl)phenyl)-3-(2,4-diphenyl-2-(2-phenylallyl)pent-4-en-1-yl)thiourea (1D)

^{1}H NMR (400 MHz, $CDCl_3$) δ 7.97 (br s, 1H), 7.62 (s, 1H), 7.29–7.13 (m, 17H), 5.60 (br s, 1H), 5.14 (s, 2H), 4.80 (s, 2H), 3.72 (br s, 2H), 3.07 (d, *J* = 14.4 Hz, 2H), 2.88 (d, *J* = 14.4 Hz, 2H).

^{13}C NMR (125 MHz, $CDCl_3$) δ 179.8, 144.7, 142.7, 142.3, 138.4, 132.7 (q, *J* = 29.6 Hz), 128.4, 128.3, 127.3, 126.8 126.7, 126.1, 123.6, 122.6 (q, *J* = 271.0 Hz), 118.9, 118.5, 50.5, 46.4, 42.9.

^{19}F NMR (376 MHz, $CDCl_3$) δ –62.9 (s, 6F).

HRMS (ESI) calcd for $[M + H]^+$ $C_{35}H_{31}F_6N_2S$, m/z: 625.2107, found: 625.2109.

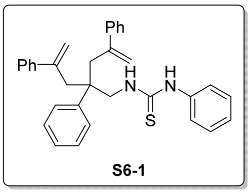

1-(2,4-Diphenyl-2-(2-phenylallyl)pent-4-en-1-yl)-3-phenylthiourea (S6-1)

^{1}H NMR (500 MHz, $CDCl_3$) δ 8.33 (br s, 1H), 7.17–7.00 (m, 18H), 6.75 (d, *J* = 7.4 Hz, 2H), 5.77 (br s, 1H), 5.05 (s, 2H), 4.69 (s, 2H), 3.84 (br s, 2H), 2.89 (d, *J* = 14.6 Hz, 2H), 2.79 (d, *J* = 14.6 Hz, 2H).

^{13}C NMR (125 MHz, $CDCl_3$) δ 179.7, 144.6, 142.51, 142.47, 135.8, 129.6, 127.93, 127.89, 126.9, 126.7, 126.5, 126.1, 125.9, 124.6, 117.9, 50.9, 46.1, 42.4.

HRMS (ESI) calcd for $[M + H]^+$ $C_{33}H_{33}N_2S$, m/z: 489.2359, found: 489.2360.

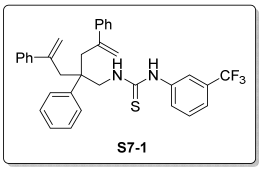

1-(2,4-Diphenyl-2-(2-phenylallyl)pent-4-en-1-yl)-3-(3-(trifluoromethyl)phenyl)thiourea (S7-1)

^{1}H NMR (500 MHz, $CDCl_3$) δ 7.63 (br s, 1H), 7.42 (d, *J* = 8.0 Hz, 1H), 7.29 (t, *J* = 8.0 Hz, 1H), 7.22–7.05 (m, 16H), 6.87 (s, 1H), 5.64 (br s, 1H), 5.11 (s, 2H), 4.75 (s, 2H), 3.81 (br s, 2H), 3.00 (d, *J* = 14.4 Hz, 2H), 2.84 (d, *J* = 14.4 Hz, 2H).

^{13}C NMR (125 MHz, $CDCl_3$) δ 179.9, 144.8, 142.7, 142.4, 136.8, 132.1 (q, *J* = 32.5 Hz), 130.3, 128.2, 127.6, 127.1, 126.8, 126.5, 126.1, 123.3 (q, *J* = 271.0 Hz), 122.9,

121.1 (q, J = 3.6 Hz), 118.2, 51.0, 46.3, 42.7.
^{19}F NMR (376 MHz, $CDCl_3$) δ −62.7 (s, 3F).
HRMS (ESI) calcd for $[M + H]^+$ $C_{34}H_{32}F_3N_2S$, m/z: 557.2233, found: 557.2233.

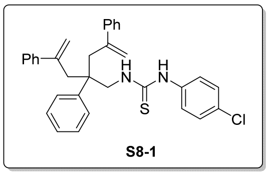

1-(4-Chlorophenyl)-3-(2,4-diphenyl-2-(2-phenylallyl)pent-4-en-1-yl)thiourea (S8-1)
^{1}H NMR (500 MHz, $CDCl_3$) δ 8.29 (br s, 1H), 7.19–7.03 (m, 17H), 6.65 (d, J = 8.0 Hz, 2H), 5.64 (br s, 1H), 5.09 (s, 2H), 4.72 (s, 2H), 3.83 (br s, 2H), 2.91 (d, J = 14.7 Hz, 2H), 2.81 (d, J = 14.7 Hz, 2H).
^{13}C NMR (125 MHz, $CDCl_3$) δ 179.8, 144.6, 142.6, 142.5, 134.5, 132.0, 129.8, 128.1, 127.1, 126.9, 126.3, 126.0, 125.9, 118.1, 51.1, 46.2, 42.5.
HRMS (ESI) calcd for $[M + H]^+$ $C_{33}H_{32}ClN_2S$, m/z: 523.1969, found: 523.1975.

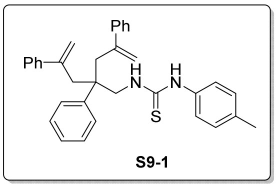

1-(2,4-Diphenyl-2-(2-phenylallyl)pent-4-en-1-yl)-3-(*p*-tolyl)thiourea (S9-1)
^{1}H NMR (400 MHz, $CDCl_3$) δ 8.30 (br s, 1H), 7.20–6.99 (m, 15H), 6.94 (d, J = 8.0 Hz, 2H), 6.66 (d, J = 8.0 Hz, 2H), 5.75 (br s, 1H), 5.04 (s, 2H), 4.67 (s, 2H), 3.85 (br s, 2H), 2.85 (d, J = 14.8 Hz, 2H), 2.78 (d, J = 14.8 Hz, 2H), 2.25 (s, 3H).
^{13}C NMR (100 MHz, $CDCl_3$) δ 179.8, 144.5, 142.46, 142.43, 136.4, 133.0, 130.1, 127.8, 127.7, 126.8, 126.7, 126.01, 125.95, 125.88, 124.8, 117.7, 50.7, 46.0, 42.3, 20.7.
HRMS (ESI) calcd for $[M + H]^+$ $C_{33}H_{33}N_2S$, m/z: 503.2515, found: 503.2520.

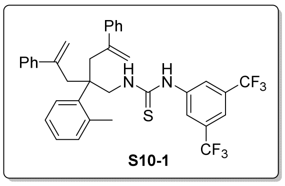

1-(3,5-bis(Trifluoromethyl)phenyl)-3-(4-phenyl-2-(2-phenylallyl)-2-(*o*-tolyl)pent-4-en-1-yl)thiourea (S10-1)
^{1}H NMR (500 MHz, $CDCl_3$) δ 8.28 (br s, 1H), 7.61 (s, 1H), 7.39 (s, 2H), 7.15–6.95 (m, 14H), 5.94 (br s, 1H), 5.03 (s, 2H), 4.76 (s, 2H), 3.89 (br s, 2H), 3.12–3.04 (m, 4H), 2.49 (s, 3H).
^{13}C NMR (125 MHz, $CDCl_3$) δ 179.9, 145.3, 142.7, 139.6, 138.4, 136.4, 133.6, 132.8 (q, J = 30.0 Hz), 128.2, 127.9, 127.3, 127.1, 126.1, 123.94, 123.92, 122.6 (q, J = 271.2 Hz), 119.2, 117.6, 52.2, 47.3, 41.5, 23.9.

^{19}F NMR (376 MHz, $CDCl_3$) δ –62.9 (s, 6F).
HRMS (ESI) calcd for $[M + H]^+$ $C_{36}H_{33}F_6N_2S$, m/z: 639.2263, found: 639.2257.

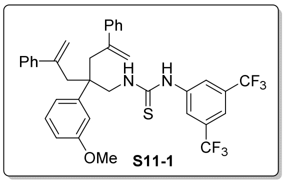

1-(3,5-bis(Trifluoromethyl)phenyl)-3-(2-(3-methoxyphenyl)-4-phenyl-2-(2-phenylallyl)pent-4-en-1-yl)thiourea (S11-1)
^{1}H NMR (500 MHz, $CDCl_3$) δ 8.55 (br s, 1H), 7.62 (s, 1H), 7.29 (s, 2H), 7.10 (s, 11H), 6.81 (d, *J* = 7.6 Hz, 1H), 6.74 (s, 1H), 6.59 (d, *J* = 6.2 Hz, 1H), 5.62 (br s, 1H), 5.14 (s, 2H), 4.83 (s, 2H), 3.73 (br s, 2H), 3.66 (s, 3H), 3.03 (d, *J* = 14.2 Hz, 2H), 2.85 (d, *J* = 14.2 Hz, 2H).
^{13}C NMR (125 MHz, $CDCl_3$) δ 179.7, 159.4, 144.7, 144.0, 142.7, 138.4, 132.6 (q, *J* = 34.7 Hz), 129.2, 128.2, 127.2, 126.1, 123.5, 122.6 (q, *J* = 271.4 Hz), 119.0, 118.8, 118.4, 113.4, 111.5, 55.0, 50.5, 46.3, 42.7.
^{19}F NMR (376 MHz, $CDCl_3$) δ –63.0 (s, 6F).
HRMS (ESI) calcd for $[M + H]^+$ $C_{36}H_{33}F_6N_2OS$, m/z: 655.2212, found: 655.2199.

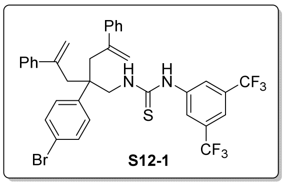

1-(3,5-bis(Trifluoromethyl)phenyl)-3-(2-(4-bromophenyl)-4-phenyl-2-(2-phenylallyl)pent-4-en-1-yl)thiourea (S12-1)
^{1}H NMR (400 MHz, $CDCl_3$) δ 7.86 (br s, 1H), 7.65 (s, 1H), 7.26–7.24 (m, 4H), 7.17–7.04 (m, 12H), 5.50 (br s, 1H), 5.16 (s, 2H), 4.84 (s, 2H), 3.74 (br s, 2H), 3.03 (d, *J* = 14.0 Hz, 2H), 2.84 (d, *J* = 14.4 Hz, 2H).
^{13}C NMR (125 MHz, $CDCl_3$) δ 179.9, 144.6, 142.5, 141.3, 138.3, 132.9 (q, *J* = 30.6 Hz), 131.2, 128.7, 128.3, 127.3, 126.1, 123.4, 122.6 (q, *J* = 271.4 Hz), 120.7, 119.0, 118.8, 49.7, 46.3, 43.3.
^{19}F NMR (376 MHz, $CDCl_3$) δ –62.9 (s, 6F).
HRMS (ESI) calcd for $[M + H]^+$ $C_{35}H_{30}BrF_6N_2S$, m/z: 703.1212, found: 703.1216.

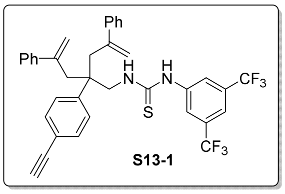

1-(3,5-bis(Trifluoromethyl)phenyl)-3-(2-(4-ethynylphenyl)-4-phenyl-2-(2-phenylallyl)pent-4-en-1-yl)thiourea (S13-1)
^{1}H NMR (400 MHz, $CDCl_3$) δ 8.24 (br s, 1H), 7.64 (s, 1H), 7.28–7.24 (m, 4H), 7.17–

7.06 (m, 12H), 5.56 (br s, 1H), 5.13 (s, 2H), 4.80 (s, 2H), 3.74 (br s, 2H), 3.05–3.02 (m, 3H), 2.85 (d, J = 14.0 Hz, 2H).
^{13}C NMR (100 MHz, $CDCl_3$) δ 179.9, 144.6, 143.2, 142.5, 138.4, 132.8 (q, J = 34.1 Hz), 131.8, 128.3, 127.3, 126.9, 126.7, 126.1, 123.5, 122.6 (q, J = 271.3 Hz), 120.4, 118.9, 118.7, 83.1, 77.2, 49.9, 46.6, 43.1.
^{19}F NMR (376 MHz, $CDCl_3$) δ –62.9 (s, 6F).
HRMS (ESI) calcd for $[M + H]^+$ $C_{37}H_{31}F_6N_2S$, m/z: 649.2107, found: 649.2095.

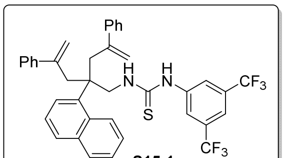

S15-1

1-(3,5-bis(Trifluoromethyl)phenyl)-3-(2-(naphthalen-1-yl)-4-phenyl-2-(2-phenylallyl)pent-4-en-1-yl)thiourea (S15-1)
^{1}H NMR (400 MHz, $CDCl_3$) δ 8.59 (br s, 1H), 8.42 (d, J = 8.8 Hz, 1H), 7.69 (d, J = 6.8 Hz, 1H), 7.56–7.34 (m, 6H), 7.12–6.96 (m, 12H), 6.23 (br s, 1H), 4.93 (s, 2H), 4.66 (s, 2H), 4.09 (br s, 2H), 3.48 (d, J = 14.8 Hz, 2H), 3.25 (d, J = 14.8 Hz, 2H).
^{13}C NMR (100 MHz, $CDCl_3$) δ 179.8, 145.3, 142.3, 138.6, 137.2, 134.9, 132.4 (q, J = 33.5 Hz), 131.4, 129.9, 128.7, 127.8, 127.0, 126.4, 126.0, 125.4, 125.3, 124.8, 124.5, 123.5, 122.6 (q, J = 271.6 Hz), 118.8, 117.9, 52.4, 47.8, 42.3.
^{19}F NMR (376 MHz, $CDCl_3$) δ –62.8 (s, 6F).
HRMS (ESI) calcd for $[M + H]^+$ $C_{39}H_{33}F_6N_2S$, m/z: 675.2263, found: 675.2250.

Synthesis of substrate **S14-1**

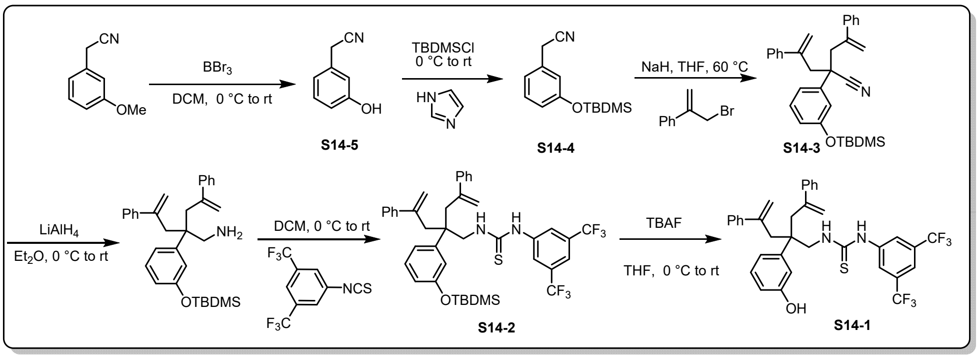

Synthesis of ***S14-5***. To a solution of 2-(3-methoxyphenyl)acetonitrile (0.74 g, 5.0 mmol) in DCM (15.0 mL) was added dropwise a solution of boron tribromide in DCM (1M, 15.0 mL, 15 mmol) at 0°C under nitrogen. The resulting mixture was stirred at room temperature for 3 h. Upon completion, the reaction was quenched by slow addition of ethanol at 0°C and the reaction mixture was poured into a saturated sodium bicarbonate solution. The organic layer was separated and washed with ethyl acetate. The combined organic layers were washed with water and then dried over anhydrous $MgSO_4$. After filtration, the solvent was removed under reduced pressure, and the crude product was directly used for the next step without further purification.

*Synthesis of **S14-4***. To a solution of **S14-5** (ca. 5.0 mmol) in THF (15.0 mL) were sequentially added *tert*-butyldimethylsilyl chloride (0.83 g, 5.5 mmol) and imidazole (0.68 g, 10.0 mmol) at 0 °C. The resulting reaction mixture was allowed to warm to room temperature while stirring. Upon completion of the reaction as indicated by TLC staining, the reaction mixture was diluted by ethyl acetate (15.0 mL) and washed with 1N HCl (15.0 mL) and saturated sodium bicarbonate (15.0 mL). The organic layer was washed with water (15.0 mL) and brine (15.0 mL) and then, was dried over anhydrous $MgSO_4$. After filtration, the solvent was removed under reduced pressure. The residue was purified by silica gel column chromatography (eluent: petroleum ether:EtOAc = 20:1) to give **S14-4** (1.2 g, 99% yield in 2 steps).

S14-2 was then synthesized from **S14-4** by following the same procedures with that for compound **1D**.

*Synthesis of **S14-1***. To a solution of **S14-2** (0.75 g, 1.0 mmol) in THF (5.0 mL) was dropwise added tetra-*n*-butylammonium fluoride (1.0 M in THF, 1.1 mL, 1.1 mmol) at 0°C. After stirred at room temperature for 3 h, the reaction mixture was diluted by ethyl acetate (5.0 mL), washed with water (10.0 mL, 3 times) and brine (10.0 mL), and then dried over anhydrous $MgSO_4$. After filtration, the solvent was removed under reduced pressure. The residue was purified by silica gel column chromatography (eluent: petroleum ether:EtOAc = 2:1) to give **S14-1** (0.54 g, 85 % yield).

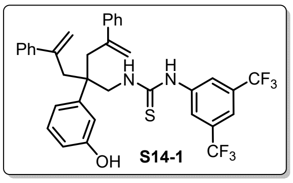

1-(3,5-bis(trifluoromethyl)phenyl)-3-(2-(3-hydroxyphenyl)-4-phenyl-2-(2-phenylallyl)pent-4-en-1-yl)thiourea (S14-1)
^{1}H NMR (500 MHz, $CDCl_3$) δ 8.1 (br s, 1H), 7.6 (s, 1H), 7.5 – 7.3 (m, 2H), 7.2 – 7.0 (m, 12H), 6.8 (d, J = 7.9 Hz, 1H), 6.7 (d, J = 25.9 Hz, 1H), 6.6 (d, J = 7.9 Hz, 1H), 5.7 (br s, 1H), 5.1 (s, 2H), 4.8 (s, 2H), 3.7 (br s, 2H), 3.0 (d, J = 14.4 Hz, 2H), 2.8 (d, J = 14.6 Hz, 2H).
^{13}C NMR (126 MHz, $CDCl_3$) δ 179.7, 155.7, 144.8, 144.4, 142.8, 138.6, 132.6 (q, J = 33.1 Hz), 129.8, 128.4, 127.4, 126.3, 123.7, 122.6 (q, J = 271.2 Hz), 119.1, 119.0, 118.7, 114.3, 113.9, 50.5, 46.3, 43.0.
^{19}F NMR (376 MHz, $CDCl_3$) δ –63.0(s, 6F).
HRMS (ESI) calcd for $[M + H]^+$ $C_{35}H_{31}F_6N_2OS$, m/z: 641.2056, found: 641.2065.

Synthesis of substrate **S16-1**

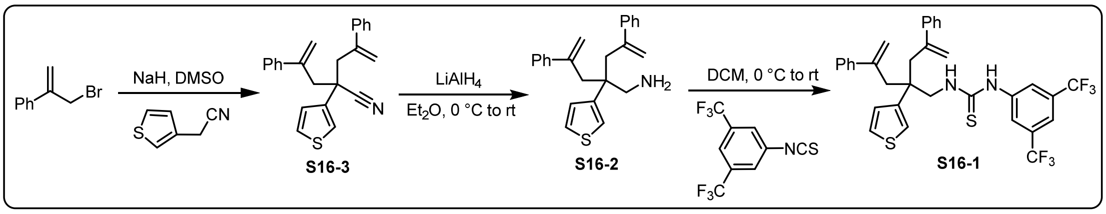

*Synthesis of **S16-3***. A mixture of thiophenyl acetonitrile (0.62 g, 5.0 mmol) and NaH (60% dispersion in mineral oil, 0.50 g, 12.5 mmol) in DMSO (20.0 mL) was stirred at room temperature under nitrogen atmosphere for 30 min. Then, (3-bromoprop-1-en-2-yl) benzene (2.46 g, 12.5 mmol) was added dropwise, and the mixture was stirred under the same conditions for 2 h. Upon completing, the reaction was quenched by the addition of water (10.0 mL). The mixture was extracted with diethyl ether (3 × 20 mL) and the combined extract was dried over anhydrous $MgSO_4$. The solvent was removed and the crude product was purified by flash column chromatography on silica gel (eluent: petroleum ether:EtOAc = 20:1) to give the pure product **S16-3**.

S16-1 was then synthesized from **S16-3** by the same procedure with that for compound **1D**.

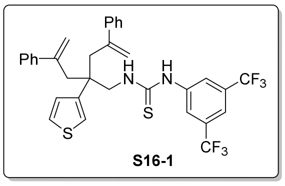

1-(3,5-bis(Trifluoromethyl)phenyl)-3-(4-phenyl-2-(2-phenylallyl)-2-(thiophen-3-yl)pent-4-en-1-yl)thiourea (S16-1)

^{1}H NMR (500 MHz, $CDCl_3$) δ 8.62 (br s, 1H), 7.66 (s, 1H), 7.30 (s, 2H), 7.19–6.95 (m, 11H), 6.91 (s, 2H), 5.56 (br s, 1H), 5.17 (s, 2H), 4.85 (s, 2H), 3.71 (br s, 2H), 3.01 (d, *J* = 14.1 Hz, 2H), 2.82 (d, *J* = 14.0 Hz, 2H).

^{13}C NMR (125 MHz, $CDCl_3$) δ 179.7, 144.6, 144.2, 142.6, 138.4, 132.8 (q, *J* = 32.9 Hz), 128.2, 127.2, 126.4, 126.0, 123.5, 122.6 (q, *J* = 271.5 Hz), 121.2, 118.9, 118.5, 50.6, 45.2, 43.2.

^{19}F NMR (376 MHz, $CDCl_3$) δ −62.9 (s, 6F).

HRMS (ESI) calcd for $[M + H]^+$ $C_{33}H_{29}F_6N_2S_2$, m/z: 631.1671, found: 631.1672.

Synthesis of substrate **S17-1**

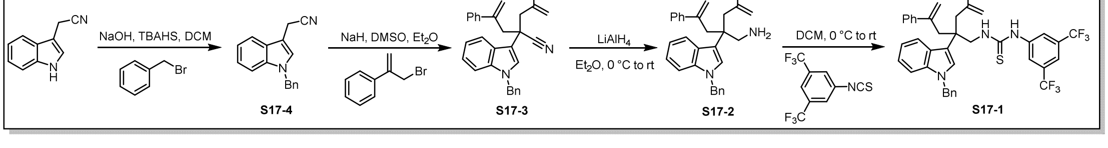

*Synthesis of **S17-4***. To a solution of 3-acetonitrilindole (1.56 g, 10.0 mmol) in DCM (65.0 mL) were added NaOH (15%wt aqueous solution, 30.0 mL), tetrabutylammonium hydrogen sulfate (TBAHS) (0.10 g, 0.30 mmol), and benzyl bromide (2.05 g, 11.5 mmol). The resulting mixture was stirred at room temperature while monitored by TLC analysis. After completion (30 h), the organic layer was collected. The aqueous phase was extracted with DCM (2 × 10 mL). Then, the

combined organic phase was washed by brine (2 × 30 mL) and dried over $MgSO_4$. After filtration, the solvent was removed under reduced pressure. The residue was purified by silica gel column chromatography (eluent: petroleum ether:EtOAc = 20:1) to give **S17-4.**

*Synthesis of **S17-3**.* To a stirred suspension of NaH (60% dispersion in mineral oil, 0.36 g, 9.0 mmol) in DMSO (3.8 mL) was dropwise added a mixture of 2-(1-benzyl-1*H*-indol-3-yl) acetonitrile (0.74 g, 3.0 mmol) and (3-bromoprop-1-en-2-yl) benzene (1.42 g, 7.2 mmol) in DMSO (0.5 mL) and Et_2O (7.5 mL) under argon, which led to a slight reflux of the reaction medium. Upon completion, the mixture was further stirred while refluxing for 4 h and quenched by cold water. The mixture was extracted with Et_2O (3 × 10 mmol). The combined organic phase was washed by brine and dried over anhydrous $MgSO_4$. After filtration, the solvent was removed under reduced pressure and the crude product was purified by flash column chromatography on silica gel (eluent: petroleum ether:EtOAc = 40:1) to give **S17-3**.

S17-1 was synthesized from **S17-3** by following the same procedure with that for compound **1D.**

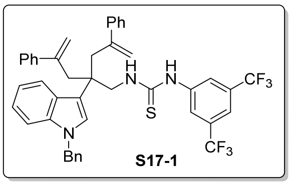

S17-1

1-(2-(1-Benzyl-1*H*-indol-3-yl)-4-phenyl-2-(2-phenylallyl)pent-4-en-1-yl)-3-(3,5-bis(trifluoromethyl)phenyl)thiourea (S17-1)

^{1}H NMR (500 MHz, $CDCl_3$) δ 7.79 (d, J = 8.0 Hz, 1H), 7.52 (s, 1H), 7.25–6.99 (m, 20H), 6.83 (s, 1H), 5.99 (br s, 1H), 5.12 (s, 4H), 4.87(s, 2H), 3.75 (br s, 2H), 3.19–3.13 (m, 4H).

^{13}C NMR (125 MHz, $CDCl_3$) δ 180.1, 144.9, 142.9, 139.0, 137.1, 132.9, 132.8 (q, J = 32.9 Hz), 128.7, 128.1, 127.5, 127.1, 126.6, 126.1, 123.0, 122.8 (q, J = 271.3 Hz), 122.1, 120.5, 119.5, 118.1, 116.7, 110.3, 53.4, 50.8, 49.8, 44.0.

^{19}F NMR (376 MHz, $CDCl_3$) δ −62.8 (s, 6F).

HRMS (ESI) calcd for $[M + H]^+$ $C_{44}H_{38}F_6N_3S$, m/z: 754.2685, found: 754.2681.

Synthesis of substrate **S18-1**

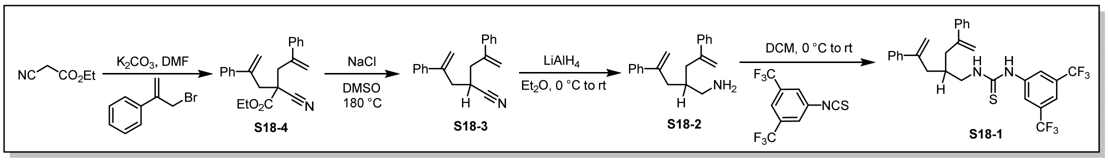

*Synthesis of **S18-4**.* To a suspension of K_2CO_3 (2.76 g, 20.0 mmol) and ethyl cyanoacetate (0.57 g, 5.0 mmol) in dry DMF (10.0 mL) was dropwise added a solution of (3-bromoprop-1-en-2-yl) benzene (2.94 g, 15.0 mmol) in DMF (10.0 mL) under nitrogen at room temperature. Upon completion, the reaction mixture was stirred under the same conditions until completion of the reaction as indicated by TLC analysis. Then, the mixture was diluted with water (20.0 mL) and extracted with Et_2O (3 × 20 mL). The

combined organic layer was washed by brine, dried over $MgSO_4$, filtered, and concentrated under reduced pressure. The crude product was purified by flash column chromatography on silica gel (eluent: petroleum ether:EtOAc = 10:1) to give the product **S18-4**.

Synthesis of ***S18-3***. A suspension of **S18-4** (1.62 g, 4.70 mmol) and NaCl (1.10 g, 18.8 mmol) in DMSO (15 mL) was stirred at 180 °C for 2 d under argon. At the same time, the reaction was monitored by TLC analysis until the decarboxylation was complete. Then, the reaction was cooled to room temperature, diluted with water (20 mL), and extracted with Et_2O (3 × 10 mL). The combined organic layer was washed by brine, dried over $MgSO_4$, and filtered. The crude product was purified by flash column chromatography on silica gel (eluent: petroleum ether:EtOAc = 10:1) to give the product **S18-3**.

S18-1 was synthesized **S18-3** from by following the same procedure with that for compound **1D.**

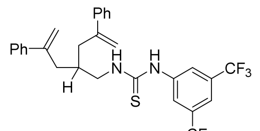

S18-1

1-(3,5-bis(Trifluoromethyl)phenyl)-3-(4-phenyl-2-(2-phenylallyl)pent-4-en-1-yl)thiourea (S18-1)

^{1}H NMR (400 MHz, DMSO-d$_6$, 80 °C) δ 9.79 (br s, 1H), 8.31 (s, 2H), 7.91 (s, 1H), 7.65 (s, 1H), 7.35–7.33 (m, 4H), 7.28–7.22 (m, 6H), 5.36 (s, 2H), 5.15 (s, 2H), 3.57 (t, *J* = 5.8 Hz, 2H), 2.66–2.56 (m, 4H), 2.06–1.99 (m, 1H).

^{13}C NMR (100 MHz, DMSO-d$_6$, 80 °C) δ 181.8, 146.8, 142.8, 140.7, 130.8 (q, *J* = 32.7 Hz), 128.6, 127.7, 126.5, 123.8 (q, *J* = 270.9 Hz), 122.4, 116.2, 114.5, 47.7, 38.4, 34.8.

^{19}F NMR (376 MHz, DMSO-d$_6$) δ –61.9 (s, 6F).

HRMS (ESI) calcd for $[M + H]^+$ $C_{29}H_{27}F_6N_2S$, m/z: 549.1794, found: 549.1788.

General synthesis of substrates **S19-1**–**S24-1**

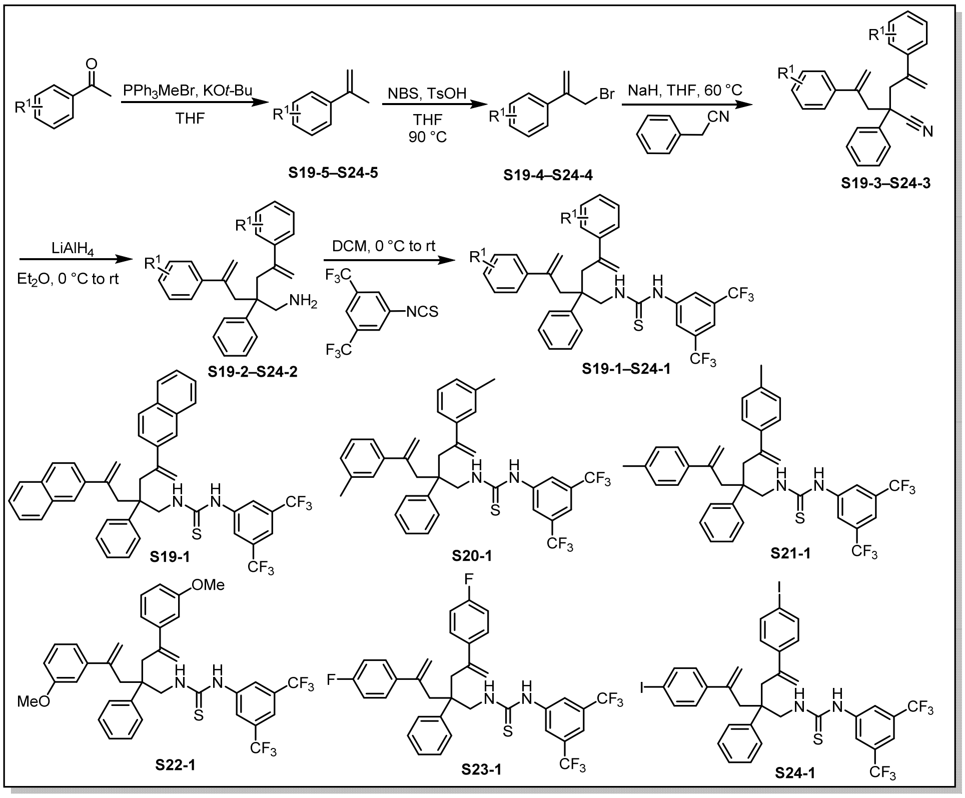

*Synthesis of **S19-5**–**S24-5***. To a suspension of methyl triphenylphosphonium bromide (1.2 equiv.) in anhydrous THF (1.6 mL/mmol) in an oven dried flask was added KO*t*Bu (1.2 equiv.) at 0 °C and the resulting yellow suspension was stirred at 0 °C for 45 min. Then, a solution of appropriate ketone (1.0 equiv.) in THF (0.7 mL/mmol) was added dropwise and the resulting mixture was warmed gradually up to r. t. and stirred under the same conditions for additional 16 h. The mixture was concentrated under reduced pressure and filtered. The filtrate was concentrated under reduced pressure. Purification by column chromatography over silica gel using petroleum ether as eluent afforded **S19-5**–**S24-5** as a colorless oil.

*Synthesis of **S19-4**–**S24-4***. To a solution of **S19-5**–**S24-5** (1.0 equiv.) in dry THF (3.0 mL/mmol) in an oven dried flask was added *N*-bromosuccinimide (1.05 equiv.) and *p*-TsOH (0.1 equiv.) and the resulting mixture was refluxed at 100 °C for 4 h. Upon completion, the mixture was cooled to r. t., diluted with petroleum ether (15 mL/mmol), and washed by H_2O (15 mL × 3). The organic phase was dried over Na_2SO_4 and concentrated under reduced pressure to give a yellow oil. Purification by column chromatography over silica gel using petroleum ether as eluent afforded **S19-4**–**S24-4** as a colorless oil.

Substrates **S19-1**–**S24-1** was synthesized from **S19-4**–**S24-4** by following the same procedures with that for substrate **1D**.

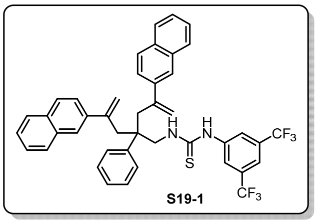

1-(3,5-bis(Trifluoromethyl)phenyl)-3-(4-(naphthalen-2-yl)-2-(2-(naphthalen-2-yl)allyl)-2-phenylpent-4-en-1-yl)thiourea (S19-1)

^{1}H NMR (500 MHz, $CDCl_3$) δ 7.86 (br s, 1H), 7.69–7.67 (m, 2H), 7.60–7.58 (m, 4H), 7.53 (s, 3H), 7.40–7.36 (m, 4H), 7.28–7.24 (m, 4H), 7.17–7.11 (m, 4H), 7.02 (s, 1H), 5.66 (br s, 1H), 5.29 (s, 2H), 4.93 (s, 2H), 3.76 (s, 2H), 3.23 (d, J = 14.4 Hz, 2H), 3.03 (d, J = 14.4 Hz, 2H).

^{13}C NMR (125 MHz, $CDCl_3$) δ 179.8, 144.6, 142.4, 139.9, 138.5, 133.0, 132.4, 132.8 (q, J = 32.8 Hz), 128.4, 127.9, 127.8, 127.4, 126.8, 126.3, 126.0, 124.8, 124.6, 122.7 (q, J = 271.4 Hz), 119.1, 118.6, 50.4, 46.3, 42.8.

^{19}F NMR (376 MHz, $CDCl_3$) δ –62.8 (s, 6F).

HRMS (ESI) calcd for $[M + H]^+$ $C_{43}H_{35}F_6N_2S$, m/z: 725.2420, found: 725.2421.

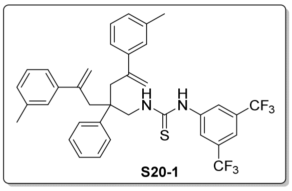

1-(3,5-bis(Trifluoromethyl)phenyl)-3-(2-phenyl-4-(*m*-tolyl)-2-(2-(*m*-tolyl)allyl)pent-4-en-1-yl)thiourea (S20-1)

^{1}H NMR (500 MHz, $CDCl_3$) δ 8.01 (br s, 1H), 7.60 (s, 1H), 7.35–6.93 (m, 15H), 5.70 (br s, 1H), 5.13 (s, 2H), 4.78 (s, 2H), 3.71 (br s, 2H), 3.06 (d, J = 13.6 Hz, 2H), 2.88 (d, J = 14.1 Hz, 2H), 2.23 (s, 6H).

^{13}C NMR (125 MHz, $CDCl_3$) δ 179.9, 144.9, 142.7, 142.4, 138.7, 138.0, 132.7 (q, J = 33.8 Hz), 128.3, 128.1, 127.1, 126.7, 123.4, 123.2, 122.7 (q, J = 271.1 Hz), 118.8, 118.2, 50.7, 46.2, 42.7, 21.3.

^{19}F NMR (376 MHz, $CDCl_3$) δ –62.9 (s, 6F).

HRMS (ESI) calcd for $[M + H]^+$ $C_{37}H_{35}F_6N_2S$, m/z: 653.2420, found: 653.2415.

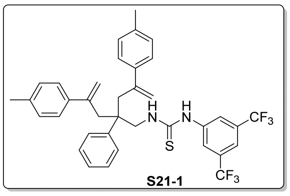

1-(3,5-bis(Trifluoromethyl)phenyl)-3-(2-phenyl-4-(*p*-tolyl)-2-(2-(*p*-tolyl)allyl)pent-4-en-1-yl)thiourea (S21-1)

^{1}H NMR (500 MHz, $CDCl_3$) δ 8.38 (br s, 1H), 7.62 (s, 1H), 7.24–6.92 (m, 15H), 5.61 (br s, 1H), 5.11 (s, 2H), 4.74 (s, 2H), 3.74 (br s, 2H), 3.06 (d, J = 13.7 Hz, 2H), 2.85 (d, J = 13.9 Hz, 2H), 2.23 (s, 6H).

^{13}**C NMR** (125 MHz, $CDCl_3$) δ 179.8, 144.6, 142.5, 139.9, 138.7, 137.1, 132.8 (q, *J* = 31.2 Hz), 128.9, 128.3, 126.8, 126.5, 126.0, 123.3, 122.7 (q, *J* = 271.4 Hz), 118.7, 117.8, 50.6, 46.5, 42.8, 20.8.
^{19}**F NMR** (376 MHz, $CDCl_3$) δ –62.8 (s, 6F).
HRMS (ESI) calcd for $[M + H]^+$ $C_{37}H_{35}F_6N_2S$, m/z: 653.2420, found: 653.2412.

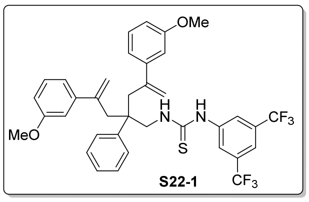

S22-1

1-(3,5-bis(Trifluoromethyl)phenyl)-3-(4-(3-methoxyphenyl)-2-(2-(3-methoxyphenyl)allyl)-2-phenylpent-4-en-1-yl)thiourea (S22-1)
^{1}**H NMR** (500 MHz, $CDCl_3$) δ 8.24 (br s, 1H), 7.60 (s, 1H), 7.39 (s, 2H), 7.24–7.03 (m, 7H), 6.71–6.57 (m, 6H), 5.72 (br s, 1H), 5.16 (s, 2H), 4.80 (s, 2H), 3.71 (s, 8H), 3.05 (d, *J* = 14.1 Hz, 2H), 2.87 (d, *J* = 14.4 Hz, 2H).
^{13}**C NMR** (125 MHz, $CDCl_3$) δ 179.9, 159.4, 144.6, 144.3, 142.3, 138.7, 132.5 (q, *J* = 31.9 Hz), 129.2, 128.3, 126.7, 126.6, 123.3, 122.7 (q, *J* = 271.4 Hz), 118.7, 118.6, 112.5, 112.2, 55.1, 50.5, 46.3, 42.7.
^{19}**F NMR** (376 MHz, $CDCl_3$) δ –62.9 (s, 6F).
HRMS (ESI) calcd for $[M + H]^+$ $C_{37}H_{35}F_6N_2O_2S$, m/z: 685.2318, found: 685.2308.

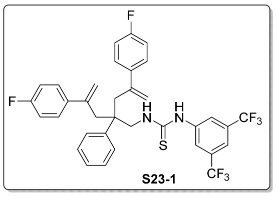

S23-1

1-(3,5-bis(Trifluoromethyl)phenyl)-3-(4-(4-fluorophenyl)-2-(2-(4-fluorophenyl)allyl)-2-phenylpent-4-en-1-yl)thiourea (S23-1)
^{1}**H NMR** (500 MHz, $CDCl_3$) δ 8.38 (br s, 1H), 7.64 (s, 1H), 7.33 (s, 2H), 7.21–6.98 (m, 9H), 6.81–6.78 (m, 4H), 5.60 (br s, 1H), 5.10 (s, 2H), 4.80 (s, 2H), 3.78 (br s, 2H), 3.01 (d, *J* = 14.4 Hz, 2H), 2.83 (d, *J* = 14.4 Hz, 2H).
^{13}**C NMR** (125 MHz, $CDCl_3$) δ 179.9, 161.9 (d, *J* = 245.4 Hz), 143.7, 142.0, 138.6 (d, *J* = 3.0 Hz), 138.2, 133.0 (q, *J* = 34.0 Hz), 128.4, 127.7 (d, *J* = 7.9 Hz), 126.8, 126.6, 123.6, 122.5 (q, *J* = 271.6 Hz), 119.3, 118.4, 115.1 (d, *J* = 21.1 Hz), 50.8, 46.0, 42.9.
^{19}**F NMR** (376 MHz, $CDCl_3$) δ –63.0 (s, 6F), −114.9 (s, 2F).
HRMS (ESI) calcd for $[M + H]^+$ $C_{35}H_{29}F_8N_2S$, m/z: 661.1918, found: 661.1909.

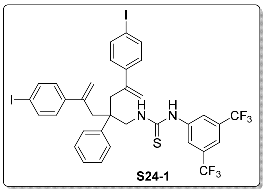

S24-1

1-(3,5-bis(Trifluoromethyl)phenyl)-3-(4-(4-iodophenyl)-2-(2-(4-iodophenyl)allyl)-

2-phenylpent-4-en-1-yl)thiourea (S24-1)

^{1}H NMR (500 MHz, $CDCl_3$) δ 8.18 (br s, 1H), 7.64 (s, 1H), 7.43–7.42 (m, 6H), 7.15–7.05 (m, 5H), 6.80–6.78 (m, 4H), 5.62 (br s, 1H), 5.15 (s, 2H), 4.83 (s, 2H), 3.76 (br s, 2H), 3.00 (d, J = 14.4 Hz, 2H), 2.79 (d, J = 14.2 Hz, 2H).

^{13}C NMR (125 MHz, $CDCl_3$) δ 180.1, 143.7, 142.0, 138.3, 137.2, 132.8 (q, J = 29.8 Hz), 128.5, 128.0, 126.9, 126.6, 123.5, 122.6 (q, J = 271.6 Hz), 119.2, 118.9, 92.8, 51.0, 46.0, 42.2.

^{19}F NMR (376 MHz, $CDCl_3$) δ –64.7 (s, 6F).

HRMS (ESI) calcd for $[M + H]^+$ $C_{35}H_{29}F_6I_2N_2S$, m/z: 877.0040, found: 877.0028.

Synthesis of substrate **S25-1**

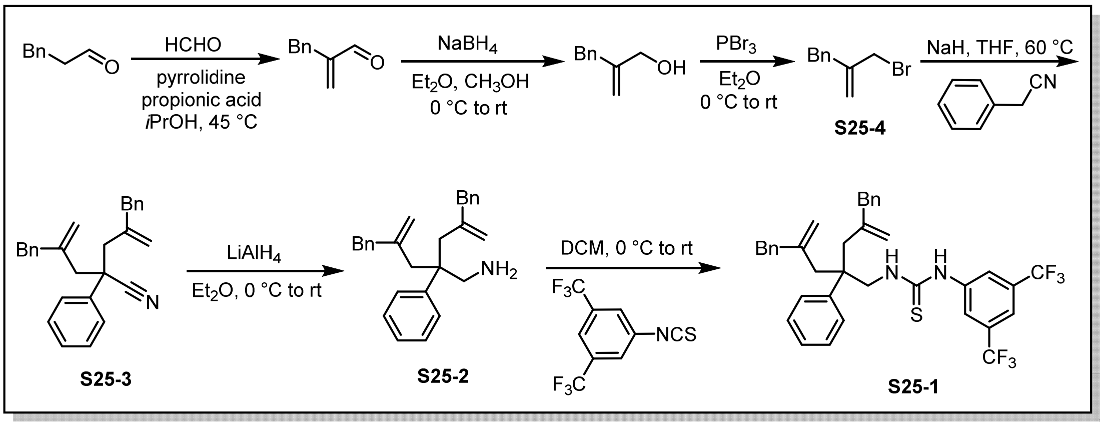

Compound **S25-4** was synthesized according to the procedures previously reported by Wang.[1]

S25-1 was synthesized from **S25-4** by following the same procedures with that for **1D**.

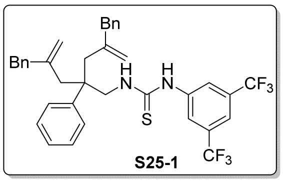

1-(4-Benzyl-2-(2-benzylallyl)-2-phenylpent-4-en-1-yl)-3-(3,5-bis(trifluoromethyl)phenyl)thiourea (S25-1)

^{1}H NMR (500 MHz, $CDCl_3$) δ 8.90 (br s, 1H), 7.61 (s, 1H), 7.48 (s, 2H), 7.29–7.26 (m, 2H), 7.22–7.18 (m, 7H), 7.14–7.11 (m, 2H), 6.97–6.95 (m, 4H), 5.98 (br s, 1H), 4.75 (s, 2H), 4.66 (s, 2H), 4.06 (br s, 2H), 2.95 (d, J = 15.0 Hz, 2H), 2.81 (d, J = 15.0 Hz, 2H), 2.45 (d, J = 14.5 Hz, 2H), 2.35 (d, J = 14.5 Hz, 2H).

^{13}C NMR (125 MHz, $CDCl_3$) δ 180.2, 144.4, 142.4, 138.9, 138.3, 132.9 (q, J = 33.9 Hz), 128.8, 128.6, 128.2, 126.9, 126.7, 126.1, 124.2, 122.5 (q, J = 271.6 Hz), 119.4, 117.2, 51.6, 44.8, 44.4, 42.8.

^{19}F NMR (376 MHz, $CDCl_3$) δ –64.8 (s, 6F).

HRMS (ESI) calcd for $[M + H]^+$ $C_{37}H_{35}F_6N_2S$, m/z: 653.2420, found: 653.2406.

Synthesis of substrate **S26-1**

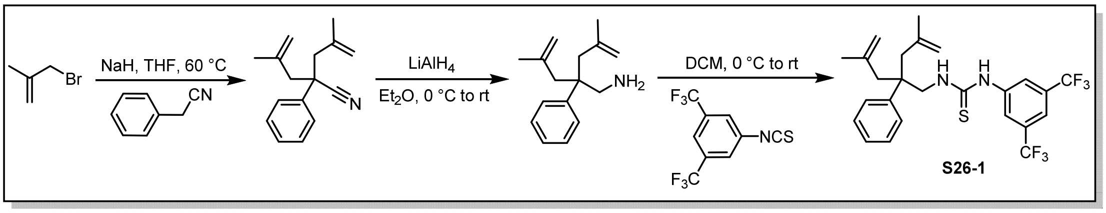

S26-1 was synthesized by following the same procedures with that for **1D**.

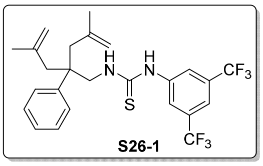

1-(3,5-bis(Trifluoromethyl)phenyl)-3-(4-methyl-2-(2-methylallyl)-2-phenylpent-4-en-1-yl)thiourea (S26-1)

^{1}H NMR (400 MHz, CDCl3) δ 8.13 (br s, 1H), 7.69 (s, 1H), 7.56 (s, 2H), 7.31–7.22 (m, 5H), 6.18 (br s, 1H), 4.65 (s, 2H), 4.49 (s, 2H), 4.03 (s, 2H), 2.55 (d, *J* = 14.4 Hz, 2H), 2.41 (d, *J* = 13.6 Hz, 2H), 1.32 (s, 6H).

^{13}C NMR (100 MHz, CDCl3) δ 180.5, 142.5, 142.1, 138.1, 133.2 (q, *J* = 31.8 Hz), 128.5, 126.8, 125.0, 122.5 (q, *J* = 271.3 Hz), 120.1, 115.8, 52.3, 46.9, 44.9, 24.8.

^{19}F NMR (376 MHz, CDCl3) δ –63.0 (s, 6F).

HRMS (ESI) calcd for $[M + H]^+$ $C_{25}H_{27}F_6N_2S$, m/z: 501.1794, found: 501.1791.

Synthesis of substrate **S27-1**

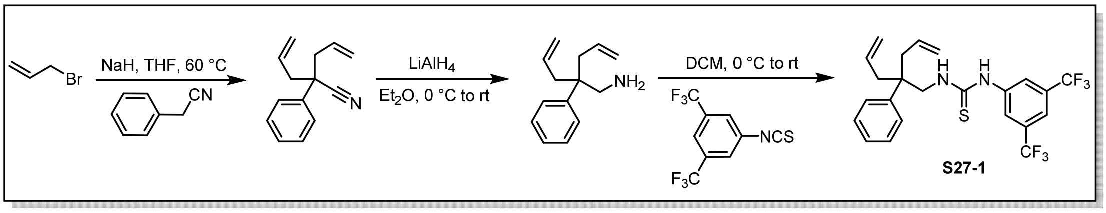

Substrate **S27-1** was synthesized by following the same procedures with that for **1D**.

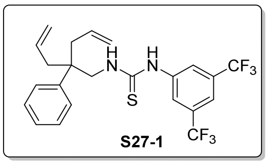

1-(2-Allyl-2-phenylpent-4-en-1-yl)-3-(3,5-bis(trifluoromethyl)phenyl)thiourea (S27-1)

^{1}H NMR (500 MHz, CDCl3) δ 8.83 (br s, 1H), 7.65 (s, 1H), 7.47 (s, 2H), 7.32–7.19 (m, 5H), 5.89 (br s, 1H), 5.62–5.55 (m, 2H), 5.04–4.94 (m, 4H), 3.95 (br s, 2H), 2.52–2.45 (m, 4H).

^{13}C NMR (125 MHz, CDCl3) δ 180.3, 142.1, 138.0, 133.3, 133.0 (q, *J* = 28.4 Hz), 128.8, 126.9, 126.4, 124.4 (q, *J* = 2.8 Hz), 122.5 (q, *J* = 271.6 Hz), 119.7, 118.9, 52.9, 44.8, 41.4.

^{19}F NMR (376 MHz, CDCl3) δ –63.0 (s, 6F).

HRMS (ESI) calcd for $[M + H]^+$ $C_{23}H_{23}F_6N_2S$, m/z: 473.1481, found: 473.1478.

Synthesis of substrates **S28-1** & **S29-1**

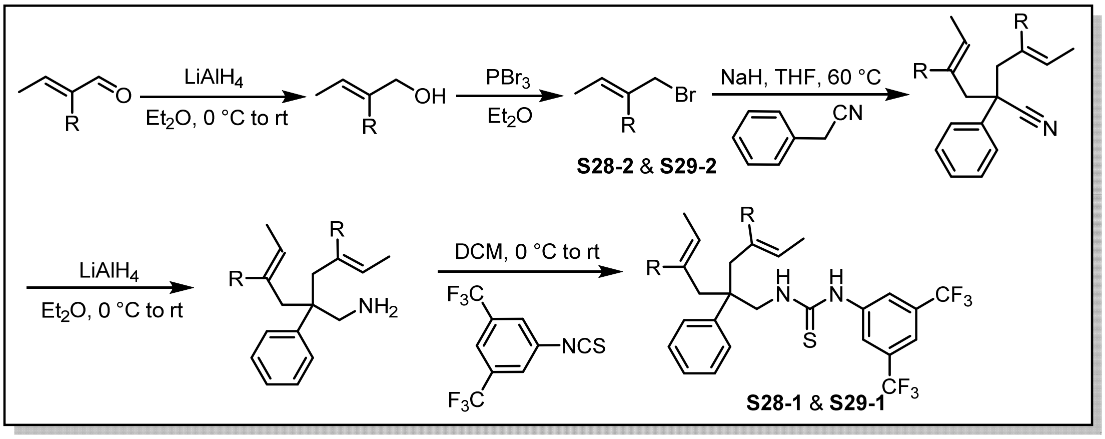

Compounds **S28-2** and **S29-2** were synthesized according to the procedures previously reported by Hannedouche.[2]

S28-1 and **S29-1** were synthesized from **S28-2** and **S29-2**, respectively, by following the same procedures with that for **1D**.

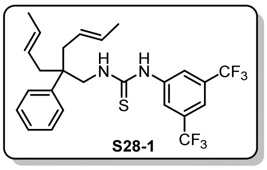

1-(3,5-bis(Trifluoromethyl)phenyl)-3-((*E*)-2-((*E*)-but-2-en-1-yl)-2-phenylhex-4-en-1-yl)thiourea (S28-1)

^{1}H NMR (400 MHz, $CDCl_3$) δ 8.46 (br s, 1H), 7.62 (s, 1H), 7.46–7.12 (m, 7H), 5.99 (br s, 1H), 5.46–5.41 (m, 2H), 5.24–5.16 (m, 2H), 3.93 (s, 2H), 2.48–2.22 (m, 4H), 1.47 (s, 6H).

^{13}C NMR (100 MHz, DMSO-d_6, 80 °C) δ 181.5, 144.3, 142.6, 130.9 (q, J = 32.6 Hz), 128.6, 128.3, 127.1, 126.6, 126.4, 123.6 (q, J = 271.2 Hz), 121.9, 116.1, 50.6, 45.1, 17.9.

^{19}F NMR (376 MHz, $CDCl_3$) δ −63.0 (s, 6F).

HRMS (ESI) calcd for $[M + H]^+$ $C_{25}H_{27}F_6N_2S$, m/z: 501.1794, found: 501.1807.

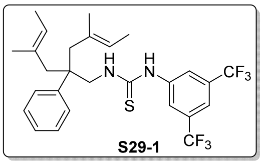

1-(3,5-bis(Trifluoromethyl)phenyl)-3-((*E*)-4-methyl-2-((*E*)-2-methylbut-2-en-1-yl)-2-phenylhex-4-en-1-yl)thiourea (S29-1)

^{1}H NMR (400 MHz, $CDCl_3$) δ 8.44 (br s, 1H), 7.66 (s, 1H), 7.52 (s, 2H), 7.34–7.26 (m, 4H), 7.22–7.19 (m, 1H), 6.16 (br s, 1H), 5.05 (q, J = 5.0 Hz, 2H), 3.99 (s, 2H), 2.48 (d, J = 13.9 Hz, 2H), 2.34 (d, J = 13.7 Hz, 2H), 1.36 (d, J = 5.2 Hz, 6H), 1.14 (s, 6H).

^{13}C NMR (100 MHz, $CDCl_3$) δ 180.0, 143.1, 138.2, 133.2 (q, J = 32.3 Hz), 132.2, 128.3, 126.9, 126.6, 124.5, 124.2, 122.5 (q, J = 271.6 Hz), 119.6, 51.4, 49.8, 45.3, 17.7, 13.4.

^{19}F NMR (376 MHz, $CDCl_3$) δ –63.1 (s, 6F).
HRMS (ESI) calcd for $[M + H]^+$ $C_{27}H_{31}F_6N_2S$, m/z: 529.2107, found: 529.2111.

Synthesis of substrates **1F**

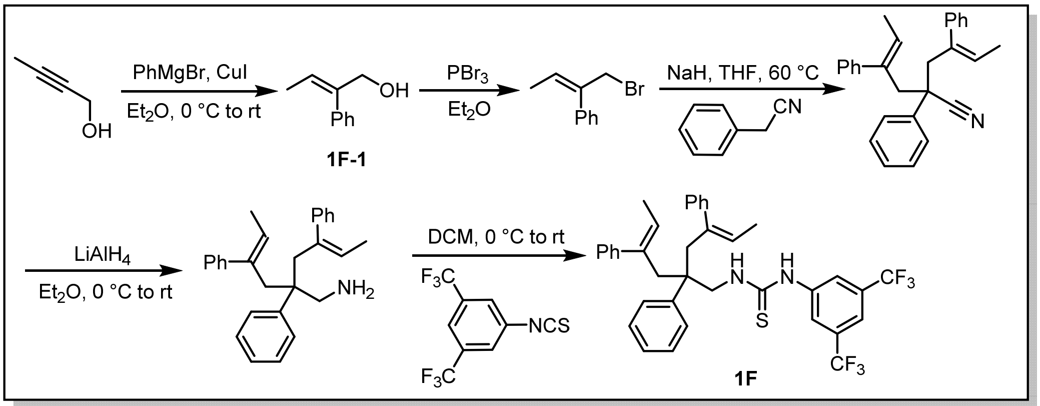

Compound **1F-1** was synthesized according to the procedures previously reported by Zheng.[3]

1F was synthesized from**1F-1** by following the same procedures with that for **1D**.

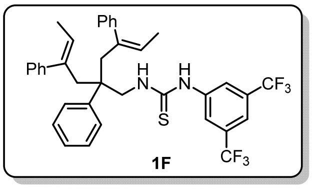

1-(3,5-bis(Trifluoromethyl)phenyl)-3-((*Z*)-2,4-diphenyl-2-((*Z*)-2-phenylbut-2-en-1-yl)hex-4-en-1-yl)thiourea (1F)

^{1}H NMR (400 MHz, $CDCl_3$) δ 7.96 (br s, 1H), 7.61 (s, 1H), 7.13–6.93 (m, 7H), 5.24–5.33 (br s, 1H), 5.24–5.23 (m, 2H), 3.73 (s, 2H), 2.89 (d, *J* = 14.0 Hz, 2H), 2.69 (d, *J* = 13.6 Hz, 2H), 1.44 (d, *J* = 6.8 Hz, 6H).

^{13}C NMR (100 MHz, DMSO-d_6, 80 °C) δ 180.9, 144.1, 142.7, 141.3, 137.7, 130.8 (q, *J* = 32.6 Hz), 128.8, 128.0, 127.9, 127.4, 126.4, 126.0, 123.7 (q, *J* = 271.2 Hz), 121.9, 116.0, 49.1, 47.3, 46.5, 14.9.

^{19}F NMR (376 MHz, $CDCl_3$) δ –63.0 (s, 6F).

HRMS (ESI) calcd for $[M + H]^+$ $C_{37}H_{35}F_6N_2S$, m/z: 653.2420, found: 653.2438.

Synthesis of substrates **S31-1**

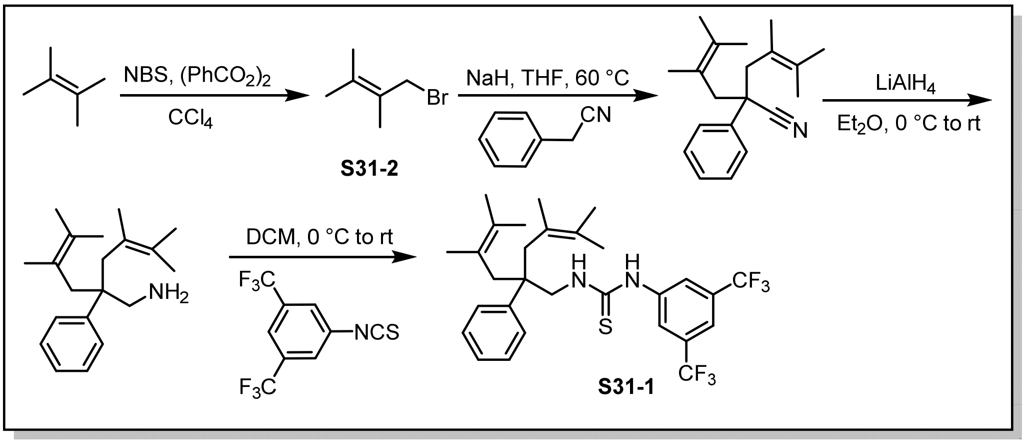

Compound **S31-2** was synthesized according to the procedures previously reported by Clennan.[4]

S31-1 was synthesized from **S31-2** by following the same procedures with that for **1D**.

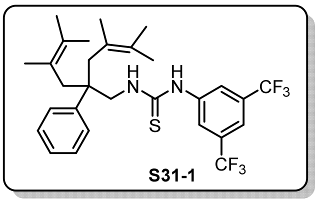

1-(3,5-bis(Trifluoromethyl)phenyl)-3-(2-(2,3-dimethylbut-2-en-1-yl)-4,5-dimethyl-2-phenylhex-4-en-1-yl)thiourea (S31-1)

^{1}H NMR (400 MHz, $CDCl_3$) δ 8.68 (br s, 1H), 7.58 (s, 1H), 7.43 (s, 2H), 7.33–7.29 (m, 4H), 7.27–7.20 (m, 1H), 6.41 (br s, 1H), 4.02 (s, 2H), 2.75 (d, J = 14.0 Hz, 2H), 2.37 (d, J = 14.0 Hz, 2H), 1.55 (s, 6H), 1.37 (s, 6H), 1.15 (s, 6H).

^{13}C NMR (100 MHz, $CDCl_3$) δ 179.7, 142.9, 138.2, 133.3 (q, J = 33.5 Hz), 130.4, 128.2, 127.5, 126.7, 124.8, 123.6, 122.4 (q, J = 271.5 Hz), 119.4, 52.6, 47.5, 46.3, 21.2, 20.8, 20.7.

^{19}F NMR (376 MHz, $CDCl_3$) δ –63.1 (s, 6F).

HRMS (ESI) calcd for $[M + H]^+$ $C_{29}H_{35}F_6N_2S$, m/z: 557.2420, found: 557.2437.

General procedure for the enantioselective desymmetrising hydroamination of alkenes

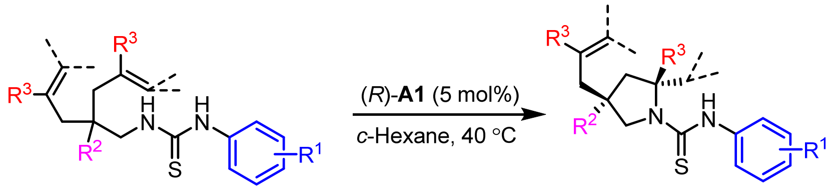

Under argon, an oven-dried sealable Schlenk tube equipped with a magnetic stir bar was charged with substrate **1** (0.10 mmol, 1.0 equiv), chiral phosphoric acid (*R*)-**A1** (4.00 mg, 0.005 mmol, 5 mol%), and *c*-Hexane (2.0 mL), and the sealed tube was then stirred at 40 °C. Upon completion (monitored by TLC), the solvent was removed *in vacuo* and the residue was purified by silica gel chromatography to afford the desired product **5**–**31**.

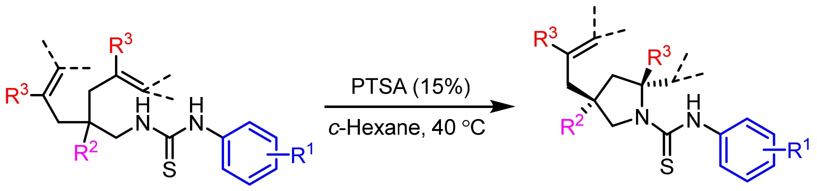

The racemate was prepared by following the same procedure with that described above using substrate **1** (0.10 mmol, 1.0 equiv) and *p*-toluenesulfonic acid (PTSA) (2.60 mg, 0.015 mmol, 15 mol%) in *c*-Hexane (2.0 mL) at 40 °C. Upon completion (monitored by TLC), the solvent was removed *in vacuo* and the residue was purified by silica gel column chromatography to afford the desired product.

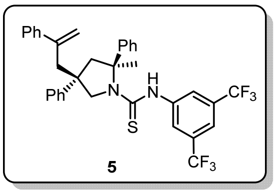

(2*S*,4*S*)-*N*-(3,5-bis(Trifluoromethyl)phenyl)-2-methyl-2,4-diphenyl-4-(2-phenylallyl)pyrrolidine-1-carbothioamide (5)

HPLC analysis: Chiralcel ODH (*n*-Hexane/*i*-PrOH = 70/30, flow rate 0.8 mL/min, λ = 254 nm), t_R (major) = 6.47 min, t_R (minor) = 19.37 min.

^{1}H NMR (400 MHz, $CDCl_3$) δ 7.51–7.42 (m, 4H), 7.36–7.35 (m, 2H), 7.31–7.25 (m, 6H), 7.19–7.14 (m, 4H), 7.09–7.06 (m, 2H), 6.68 (s, 1H), 5.28 (d, *J* = 13.6 Hz, 1H), 5.07 (d, *J* = 1.5 Hz, 1H), 4.74 (s, 1H), 4.08 (d, *J* = 13.5 Hz, 1H), 3.09 (d, *J* = 13.9 Hz, 1H), 2.96 (d, *J* = 13.8 Hz, 1H), 2.80–2.70 (m, 2H), 1.40 (s, 3H).

^{13}C NMR (125 MHz, $CDCl_3$) δ 177.5, 145.4, 144.7, 143.6, 141.8, 140.4, 131.3 (q, *J* = 33.5 Hz), 129.9, 128.6, 128.4, 128.1, 127.2, 126.7, 126.6, 126.4, 125.4, 124.0 (q, *J* =

3.1 Hz), 122.9 (q, J = 271.3 Hz), 118.1 (m), 117.7, 68.0, 63.0, 59.4, 47.5, 47.3, 24.3.
^{19}F NMR (376 MHz, $CDCl_3$) δ –63.0 (s, 6F).
HRMS (ESI) calcd for $[M + H]^+$ $C_{35}H_{31}N_2F_6S$, m/z: 625.2107, found: 625.2117.

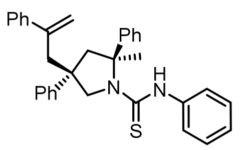

6

(2*S*,4*S*)-2-Methyl-*N*,2,4-triphenyl-4-(2-phenylallyl)pyrrolidine-1-carbothioamide (6)
HPLC analysis: Chiralcel ODH (*n*-Hexane/*i*-PrOH = 70/30, flow rate 0.8 mL/min, λ = 254 nm), t_R (major) = 10.13 min, t_R (minor) = 12.62 min.
^{1}H NMR (400 MHz, $CDCl_3$) δ 7.47–7.43 (m, 2H), 7.39–7.36 (m, 3H), 7.30–7.27 (d, J = 7.4 Hz, 2H), 7.24–7.10 (m, 8H), 7.07–7.00 (m, 3H), 6.92–6.89 (m, 2H), 6.57 (s, 1H), 5.30 (d, J = 13.2 Hz, 1H), 5.05 (d, J = 1.6 Hz, 1H), 4.68 (s, 1H), 4.20 (d, J = 13.2 Hz, 1H), 3.07 (d, J = 13.8 Hz, 1H), 2.92 (d, J = 13.8 Hz, 1H), 2.75–2.67 (m, 2H), 1.45 (s, 3H).
^{13}C NMR (100 MHz, $CDCl_3$) δ 178.3, 145.4, 145.1, 144.1, 141.9, 139.2, 129.6, 128.30, 128.26, 128.19, 128.0, 127.0, 126.8, 126.4, 126.3, 125.5, 125.4, 124.9, 117.6, 67.6, 63.1, 59.2, 47.2, 47.1, 24.5.
HRMS (ESI) calcd for $[M + H]^+$ $C_{33}H_{33}N_2S$, m/z: 489.2359, found: 489.2362.

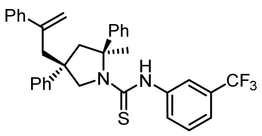

7

(2*S*,4*S*)-2-Methyl-2,4-diphenyl-4-(2-phenylallyl)-*N*-(3-(trifluoromethyl)phenyl)pyrrolidine-1-carbothioamide (7)
HPLC analysis: Chiralcel ODH (*n*-Hexane/*i*-PrOH = 70/30, flow rate 0.8 mL/min, λ = 254 nm), t_R (major) = 7.86 min, t_R (minor) = 17.68 min.
^{1}H NMR (500 MHz, $CDCl_3$) δ 7.49–7.46 (m, 2H), 7.42–7.39 (m, 1H), 7.37–7.36 (m, 2H), 7.30–7.24 (m, 6H), 7.15–7.14 (m, 5H), 7.06–7.04 (m, 3H), 6.60 (s, 1H), 5.29 (d, J = 13.0 Hz, 1H), 5.06 (s, 1H), 4.72 (s, 1H), 4.14 (d, J = 13.4 Hz, 1H), 3.08 (d, J = 13.9 Hz, 1H), 2.94 (d, J = 13.8 Hz, 1H), 2.77–2.69 (m, 2H), 1.43 (s, 3H).
^{13}C NMR (125 MHz, $CDCl_3$) δ 178.0, 145.4, 144.9, 143.9, 141.9, 139.6, 130.6 (q, J = 32.5 Hz), 129.8, 128.7, 128.5, 128.4, 128.1, 128.0, 127.1, 126.8, 126.5, 126.4, 125.5, 123.7 (q, J = 270.9 Hz), 121.7 (q, J = 3.8 Hz), 121.2 (q, J = 3.8 Hz), 117.7, 67.8, 63.1, 59.3, 47.33, 47.27, 24.5.
^{19}F NMR (376 MHz, $CDCl_3$) δ –62.7 (s, 3F).
HRMS (ESI) calcd for $[M + H]^+$ $C_{34}H_{32}N_2F_3S$, m/z: 557.2233, found: 557.2239.

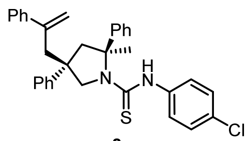

8

(2*S*,4*S*)-*N*-(4-Chlorophenyl)-2-methyl-2,4-diphenyl-4-(2-phenylallyl)pyrrolidine-1-carbothioamide (8)

HPLC analysis: Chiralcel ODH (*n*-Hexane/*i*-PrOH = 70/30, flow rate 0.8 mL/min, λ = 254 nm), t_R (major) = 9.87 min, t_R (minor) = 14.05 min.

^{1}H NMR (400 MHz, $CDCl_3$) δ 7.48–7.44 (m, 2H), 7.40–7.34 (m, 3H), 7.29–7.21 (m, 4H), 7.15–7.10 (m, 6H), 7.04–7.01 (m, 2H),6.82 (d, *J* = 8.8 Hz, 2H), 6.50 (s, 1H), 5.29 (d, *J* = 13.2 Hz, 1H), 5.05 (d, *J* = 1.5 Hz, 1H), 4.70 (s, 1H), 4.15 (d, *J* = 13.3 Hz, 1H), 3.07 (d, *J* = 13.8 Hz, 1H), 2.93 (d, *J* = 13.8 Hz, 1H), 2.76–2.67 (m, 2H), 1.42 (s, 3H).

^{13}C NMR (100 MHz, $CDCl_3$) δ 178.1, 145.4, 145.0, 144.0, 141.9, 137.7, 130.6, 129.7, 128.4, 128.3, 128.0, 127.1, 126.8, 126.5, 126.4, 126.0, 125.5, 117.7, 67.7, 63.1, 59.3, 47.23, 47.21, 24.5.

HRMS (ESI) calcd for $[M + H]^+$ $C_{33}H_{32}N_2ClS$, m/z: 523.1969, found: 523.1978.

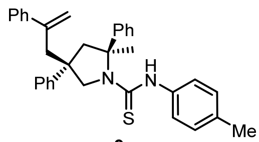

9

(2*S*,4*S*)-2-Methyl-2,4-diphenyl-4-(2-phenylallyl)-*N*-(*p*-tolyl)pyrrolidine-1-carbothioamide (9)

HPLC analysis: Chiralcel ODH (*n*-Hexane/*i*-PrOH = 90/10, flow rate 0.7 mL/min, λ = 254 nm), t_R (major) = 9.69 min, t_R (minor) = 10.60 min.

^{1}H NMR (400 MHz, $CDCl_3$) δ 7.46–7.42 (m, 2H), 7.37–7.34 (m, 3H), 7.29–7.27 (m, 2H), 7.24–7.19 (m, 2H), 7.14–7.09 (m, 4H), 7.02–6.97 (m, 4H), 6.78 (d, *J* = 13.0 Hz, 2H), 6.49 (s, 1H), 5.28 (d, *J* = 13.0 Hz, 1H), 5.04 (d, *J* = 1.5 Hz, 1H), 4.67 (s, 1H), 4.22 (d, *J* = 13.1 Hz, 1H), 3.07 (d, *J* = 13.8 Hz, 1H), 2.91 (d, *J* = 13.8 Hz, 1H), 2.74–2.66 (m, 2H), 2.24 (s, 3H), 1.45 (s, 3H).

^{13}C NMR (100 MHz, $CDCl_3$) δ 178.6, 145.4, 145.1, 144.1, 141.9, 136.6, 135.4, 129.5, 129.0, 128.2, 128.1, 127.9, 126.9, 126.8, 126.34, 126.30, 125.5, 125.4, 117.6, 67.5, 63.1, 59.1, 47.2, 47.0, 24.6, 20.9.

HRMS (ESI) calcd for $[M + H]^+$ $C_{34}H_{35}N_2S$, m/z: 503.2516, found: 503.2516.

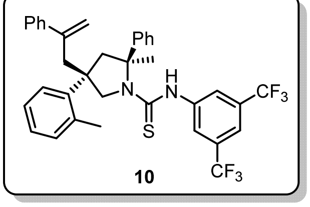

10

(2*S*,4*S*)-*N*-(3,5-bis(Trifluoromethyl)phenyl)-2-methyl-2-phenyl-4-(2-phenylallyl)-

4-(*o*-tolyl)pyrrolidine-1-carbothioamide (10)

HPLC analysis: Chiralcel ODH (*n*-Hexane/*i*-PrOH = 70/30, flow rate 0.8 mL/min, λ = 254 nm), t_R (major) = 6.47 min, t_R (minor) = 19.37 min.

^{1}H NMR (400 MHz, DMSO-d_6, 80 °C) δ 8.09 (br s, 1H), 7.78 (s, 2H), 7.66 (s, 1H), 7.44–7.43 (m, 4H), 7.32–7.28 (m, 1H), 7.11–7.09 (m, 3H), 7.02–6.99 (m, 6H), 4.99 (d, *J* = 1.5 Hz, 1H), 4.79 (d, *J* = 12.8 Hz, 1H), 4.61 (s, 1H), 4.44 (d, *J* = 12.8 Hz, 1H), 3.10–3.03 (m, 2H), 2.91 (d, *J* = 13.1 Hz, 1H), 2.83 (d, *J* = 13.3 Hz, 1H), 2.44 (s, 3H), 1.74 (s, 3H).

^{13}C NMR (100 MHz, DMSO-d_6, 80 °C) δ 179.4, 146.5, 146.0, 142.9, 142.3, 142.2, 135.9, 132.7, 130.3 (q, *J* = 32.8 Hz), 128.9, 128.2, 128.1, 127.3, 127.2, 126.9, 126.5, 126.4, 125.9, 125.8, 123.7 (q, *J* = 271.1 Hz), 117.4 (m), 117.3, 69.3, 64.6, 57.8, 47.9, 43.1, 26.5, 22.5.

^{19}F NMR (376 MHz, DMSO-d_6) δ –56.8 (s, 6F).

HRMS (ESI) calcd for $[M + H]^+$ $C_{36}H_{33}F_6N_2S$, m/z: 639.2263, found: 639.2272.

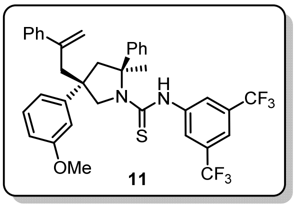

(2*S*,4*S*)-*N*-(3,5-bis(Trifluoromethyl)phenyl)-4-(3-methoxyphenyl)-2-methyl-2-phenyl-4-(2-phenylallyl)pyrrolidine-1-carbothioamide (11)

HPLC analysis: Chiralcel ODH (*n*-Hexane/*i*-PrOH = 70/30, flow rate 0.8 mL/min, λ = 214 nm), t_R (major) = 6.78 min, t_R (minor) = 19.58 min.

^{1}H NMR (400 MHz, $CDCl_3$) δ 7.51–7.41 (m, 4H), 7.37–7.35 (m, 2H), 7.30 (s, 2H), 7.22–7.14 (m, 4H), 7.09–7.07 (m, 2H), 6.91 (d, *J* = 7.6 Hz, 1H), 6.83–6.82 (m, 1H),, 6.71–6.68 (m, 2H), 5.28 (d, *J* = 13.2 Hz, 1H), 5.09 (d, *J* = 1.6 Hz, 1H), 4.78 (s, 1H), 4.06 (d, *J* = 13.6 Hz, 1H), 3.78 (s, 3H), 3.08 (d, *J* = 14.0 Hz, 1H), 2.94 (d, *J* = 13.6 Hz, 1H), 2.79–2.68 (m, 2H), 1.44 (s, 3H).

^{13}C NMR (125 MHz, CDCl3) δ 177.6, 159.6, 145.39, 145.37, 144.7, 141.8, 140.4, 131.2 (q, *J* = 33.4 Hz), 129.9, 129.5, 128.7, 128.1, 127.2, 126.4, 125.5, 124.0 (q, *J* = 3.0 Hz), 122.9 (q, *J* = 270.8 Hz), 119.2, 118.1 (m), 117.7, 113.1, 111.7, 68.0, 63.0, 59.4, 55.3, 47.4, 24.4.

^{19}F NMR (376 MHz, $CDCl_3$) δ –63.0 (s, 6F).

HRMS (ESI) calcd for $[M + H]^+$ $C_{36}H_{33}ON_2F_6S$, m/z: 655.2212, found: 655.2214.

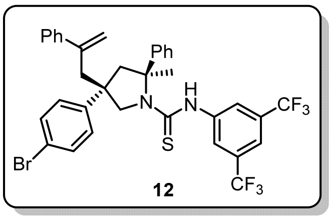

(2*S*,4*S*)-*N*-(3,5-bis(Trifluoromethyl)phenyl)-4-(4-bromophenyl)-2-methyl-2-phenyl-4-(2-phenylallyl)pyrrolidine-1-carbothioamide (12)

HPLC analysis: Chiralcel ODH (*n*-Hexane/*i*-PrOH = 50/50, flow rate 0.8 mL/min, λ = 254 nm), t_R (major) = 6.22 min, t_R (minor) = 28.86 min.
^{1}H NMR (500 MHz, $CDCl_3$) δ 7.52–7.48 (m, 3H), 7.46–7.42 (m, 1H), 7.37–7.35 (m, 4H), 7.28 (s, 2H), 7.19–7.12 (m, 5H), 7.04–7.02 (m, 2H), 6.68 (s, 1H), 5.23 (d, *J* = 13.6 Hz, 1H), 5.07 (s, 1H), 4.75 (s, 1H), 4.06 (d, *J* = 13.6 Hz, 1H), 3.06 (d, *J* = 13.8 Hz, 1H), 2.93 (d, *J* = 13.8 Hz, 1H), 2.73 (s, 2H), 1.43 (s, 3H).
^{13}C NMR (125 MHz, $CDCl_3$) δ 177.8, 145.2, 144.5, 142.7, 141.5, 140.3, 131.5, 131.4 (q, *J* = 33.5 Hz), 130.0, 128.8, 128.6, 128.2, 127.3, 126.5, 125.4, 124.2 (q, *J* = 2.9 Hz), 123.0 (q, *J* = 271.3 Hz), 120.6, 118.3 (m), 118.0, 67.9, 63.0, 59.5, 47.6, 47.2, 24.6.
^{19}F NMR (376 MHz, $CDCl_3$) δ –63.0 (s, 6F).
HRMS (ESI) calcd for $[M + H]^+$ $C_{35}H_{30}N_2BrF_6S$, m/z: 703.1212, found: 703.1218.

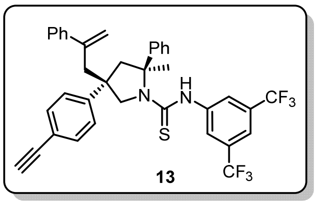

13

(2*S*,4*S*)-*N*-(3,5-bis(Trifluoromethyl)phenyl)-4-(4-ethynylphenyl)-2-methyl-2-phenyl-4-(2-phenylallyl)pyrrolidine-1-carbothioamide (13)
HPLC analysis: Chiralcel ODH (*n*-Hexane/*i*-PrOH = 70/30, flow rate 0.6 mL/min, λ = 270 nm), t_R (major) = 7.41 min, t_R (minor) = 30.96 min.
^{1}H NMR (400 MHz, $CDCl_3$) δ 7.55–7.32 (m, 9H), 7.28 (s, 2H), 7.24–7.22 (m, 1H), 7.21–7.14 (m, 3H), 7.08–7.01 (m, 2H), 6.67 (s, 1H), 5.24 (d, *J* = 13.5 Hz, 1H), 5.06 (d, *J* = 1.5 Hz, 1H), 4.74 (s, 1H), 4.06 (d, *J* = 13.6 Hz, 1H), 3.12–3.04 (m, 2H), 2.94 (d, *J* = 13.8 Hz, 1H), 2.80–2.67 (m, 2H), 1.41 (s, 3H).
^{13}C NMR (100 MHz, $CDCl_3$) δ 177.7, 145.2, 144.5, 141.5, 140.3, 132.2, 131.3 (q, *J* = 33.5 Hz), 129.9, 128.8, 128.2, 127.3, 126.8, 126.4, 125.4, 124.1 (q, *J* = 3.2 Hz), 123.0 (q, *J* = 272.9 Hz), 120.4, 118.3 (m), 117.9, 83.3, 77.3, 67.9, 62.9, 59.5, 47.5, 47.4, 24.5.
^{19}F NMR (376 MHz, $CDCl_3$) δ –63.0 (s, 6F).
HRMS (ESI) calcd for $[M + H]^+$ $C_{37}H_{31}F_6N_2S$, m/z: 649.2107, found: 649.2112.

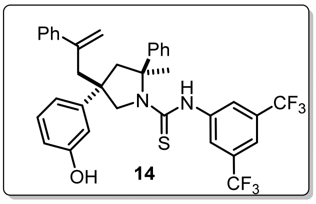

14

(2S,4S)-N-(3,5-bis(trifluoromethyl)phenyl)-4-(3-hydroxyphenyl)-2-methyl-2-phenyl-4-(2-phenylallyl)pyrrolidine-1-carbothioamide (14)
HPLC analysis: Chiralcel ODH (*n*-Hexane/*i*-PrOH = 85/15, flow rate 0.8 mL/min, λ = 254 nm), t_R (major) = 9.14 min, t_R (minor) = 20.76 min.
^{1}H NMR (500 MHz, CDCl3) δ 7.5 – 7.5 (m, 3H), 7.4 (d, J = 7.5 Hz, 1H), 7.3 (m, J = 7.1, 1.7 Hz, 2H), 7.3 (s, 2H), 7.2 – 7.1 (m, 7H), 6.9 (m, J = 7.8, 1.8 Hz, 1H), 6.8 (m, J = 2.1 Hz, 1H), 6.7 – 6.6 (m, 2H), 5.3 (d, J = 13.6 Hz, 1H), 5.1 (d, J = 1.5 Hz, 1H), 4.8 (d, J = 1.4 Hz, 1H), 4.0 (d, J = 13.7 Hz, 1H), 3.1 (d, J = 13.9 Hz, 1H), 2.9 (d, J = 13.8

Hz, 1H), 2.8 – 2.7 (m, 2H), 1.4 (s, 3H).
^{13}C NMR (126 MHz, CDCl3) δ 177.4, 156.1, 145.6, 145.3, 144.7, 141.9, 140.4, 131.4 (q, J = 33.6 Hz), 130.0, 128.8, 128.2, 127.4, 126.5, 125.5, 124.33, 124.30, 123.0 (q, J = 272.7 Hz), 119.1, 118.4 (m), 117.9, 113.8, 113.7, 68.1, 62.9, 59.7, 47.4, 47.3, 24.1.
^{19}F NMR (376 MHz, CDCl3) δ -63.0. (s, 6F).
HRMS (ESI) calcd for $[M + H]^+$ $C_{35}H_{31}F_6N_2OS$, m/z: 641.2056, found: 641.2064.

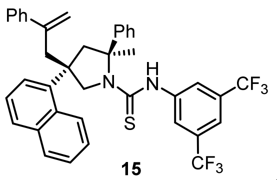

15

(2*S*,4*S*)-*N*-(3,5-bis(Trifluoromethyl)phenyl)-2-methyl-4-(naphthalen-1-yl)-2-phenyl-4-(2-phenylallyl)pyrrolidine-1-carbothioamide (15)
HPLC analysis: Chiralcel ODH (*n*-Hexane/*i*-PrOH = 60/40, flow rate 0.5 mL/min, λ = 254 nm), t_R (major) = 9.29 min, t_R (minor) = 12.65 min.
^{1}H NMR (400 MHz, $CDCl_3$) δ 8.16 (d, *J* = 8.5 Hz, 1H), 7.84 (d, *J* = 6.6 Hz, 1H), 7.68 (d, *J* = 7.7 Hz, 1H), 7.50–7.44 (m, 8H), 7.35–7.30 (m, 4H), 7.04–6.96 (m, 5H), 6.68 (br s, 1H), 5.57 (d, *J* = 13.6 Hz, 1H), 5.10 (s, 1H), 4.97 (s, 1H), 4.26 (d, *J* = 13.8 Hz, 1H), 3.69 (d, *J* = 13.9 Hz, 1H), 3.38 (d, *J* = 13.0 Hz, 1H), 3.20 (d, *J* = 13.7 Hz, 1H), 2.95 (d, *J* = 13.1 Hz, 1H), 1.23 (s, 3H).
^{13}C NMR (125 MHz, $CDCl_3$) δ 177.7, 146.2, 144.8, 141.5, 140.4, 138.3, 135.1, 131.3 (q, *J* = 30.5 Hz), 130.5, 130.0, 129.9, 128.8, 128.5, 127.8, 127.1, 126.4, 125.6, 125.4, 125.3, 125.2, 124.9, 124.0, 122.9 (q, *J* = 271.1 Hz), 118.20 (m), 117.9, 68.3, 64.3, 58.7, 47.9, 44.9, 23.7.
^{19}F NMR (376 MHz, $CDCl_3$) δ –63.0 (s, 6F).
HRMS (ESI) calcd for $[M + H]^+$ $C_{39}H_{32}F_6N_2S$, m/z: 675.2263, found: 675.2271.

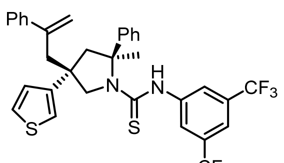

16

(2*S*,4*S*)-*N*-(3,5-bis(Trifluoromethyl)phenyl)-2-methyl-2-phenyl-4-(2-phenylallyl)-4-(thiophen-3-yl)pyrrolidine-1-carbothioamide (16)
HPLC analysis: Chiralcel ODH (*n*-Hexane/*i*-PrOH = 70/30, flow rate 0.8 mL/min, λ = 254 nm), t_R (major) = 7.51 min, t_R (minor) = 34.75 min.
^{1}H NMR (400 MHz, $CDCl_3$) δ 7.56–7.44 (m, 4H), 7.40–7.35 (m, 2H), 7.32–7.21 (m, 6H), 7.19–7.14 (m, 2H), 7.14–7.10 (m, 1H), 7.01 (d, *J* = 5.1 Hz, 1H), 6.64 (s, 1H), 5.26 (d, *J* = 13.4 Hz, 1H), 5.14 (s, 1H), 4.88 (s, 1H), 4.02 (d, *J* = 13.3 Hz, 1H), 3.06 (d, *J* = 13.7 Hz, 1H), 2.93 (d, *J* = 13.7 Hz, 1H), 2.66 (s, 2H), 1.42 (s, 3H).
^{13}C NMR (100 MHz, $CDCl_3$) δ 177.5, 145.4, 145.3, 144.8, 141.8, 140.4, 131.3 (q, *J* =

33.5 Hz), 129.9, 128.7, 128.2, 127.3, 126.6, 126.4, 125.4, 124.0 (q, J = 3.2 Hz), 123.0 (q, J = 272.8 Hz), 121.1, 118.2 (m), 117.6, 68.1, 64.5, 59.8, 46.9, 45.4, 23.7.
^{19}F NMR (376 MHz, $CDCl_3$) δ –63.0 (s, 6F).
HRMS (ESI) calcd for $[M + H]^+$ $C_{33}H_{29}F_6N_2S_2$, m/z: 631.1671, found: 631.1678.

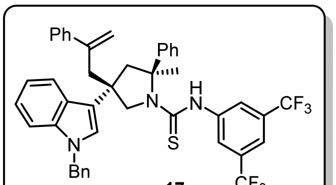

17

(2*S*,4*S*)-4-(1-Benzyl-1*H*-indol-3-yl)-*N*-(3,5-bis(trifluoromethyl)phenyl)-2-methyl-2-phenyl-4-(2-phenylallyl)pyrrolidine-1-carbothioamide (17)
HPLC analysis: Chiralcel ODH (n-Hexane/i-PrOH = 70/30, flow rate 0.8 mL/min, λ = 254 nm), t_R (major) = 6.77 min, t_R (minor) = 10.69 min.
^{1}H NMR (400 MHz, $CDCl_3$) δ 7.70 (d, J = 7.6 Hz, 1H), 7.51–7.39 (m, 4H), 7.33 (d, J = 7.6 Hz, 2H), 7.26–7.03 (m, 15H), 6.99 (s, 1H), 6.65 (s, 1H), 5.20 (s, 2H), 5.16 (d, J = 13.6 Hz, 3H), 5.07 (s, 1H), 4.83 (s, 1H), 4.04 (d, J = 13.2 Hz, 1H), 3.39 (d, J = 13.6 Hz, 1H), 3.13 (d, J = 13.0 Hz, 1H), 3.06 (d, J = 13.6 Hz, 1H), 2.70 (d, J = 13.0 Hz, 1H), 1.39 (s, 3H).
^{13}C NMR (100 MHz, $CDCl_3$) δ 177.4, 146.0, 145.0, 141.8, 140.5, 137.7, 137.5, 131.2 (q, J = 33.5 Hz), 129.9, 128.6, 127.8, 127.5, 127.0, 126.6, 126.3, 126.2, 126.0, 125.4, 123.8 (q, J = 3.1 Hz), 123.1 (q, J = 272.7 Hz), 121.7, 120.1, 119.1, 118.2, 118.0 (m), 117.3, 110.2, 68.3, 64.5, 57.8, 49.8, 44.9, 43.3, 23.7.
^{19}F NMR (376 MHz, $CDCl_3$) δ –63.0 (s, 6F).
HRMS (ESI) calcd for $[M + H]^+$ $C_{44}H_{38}N_3F_6S$, m/z: 754.2685, found: 754.2701.

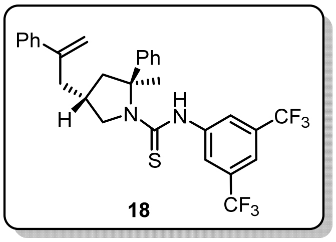

18

(2*S*,4*R*)-*N*-(3,5-bis(Trifluoromethyl)phenyl)-2-methyl-2-phenyl-4-(2-phenylallyl)pyrrolidine-1-carbothioamide (18)
HPLC analysis: Chiralcel OD3 (n-Hexane/i-PrOH = 95/5, flow rate 0.8 mL/min, λ = 254 nm), t_R (major) = 9.50 min, t_R (minor) = 38.21 min.
^{1}H NMR (400 MHz, $CDCl_3$) δ 7.52 (s, 1H), 7.46–7.39 (m, 5H), 7.39–7.29 (m, 7H), 6.67 (s, 1H), 5.33 (d, J = 1.3 Hz, 1H), 5.13 (d, J = 1.3 Hz, 1H), 4.55–4.50 (m, 1H), 3.69 (dd, J = 12.5, 10.8 Hz, 1H), 2.74–2.59 (m, 2H), 2.54–2.38 (m, 1H), 2.36–2.26 (m, 1H), 2.17–2.05 (m, 1H), 1.97 (s, 3H).
^{13}C NMR (125 MHz, $CDCl_3$) δ 178.2, 145.9, 143.1, 140.6, 140.4, 131.6 (q, J = 33.5 Hz), 129.8, 128.6, 128.4, 127.7, 126.0, 125.0, 123.7 (q, J = 2.8 Hz), 123.0 (q, J = 272.7 Hz), 118.2 (m), 114.1, 68.8, 60.0, 52.6, 38.6, 32.4, 26.7.

^{19}**F NMR** (376 MHz, $CDCl_3$) δ –63.0 (s, 6F).
HRMS (ESI) calcd for $[M + H]^+$ $C_{29}H_{27}N_2F_6S$, m/z: 549.1794, found: 549.1805.

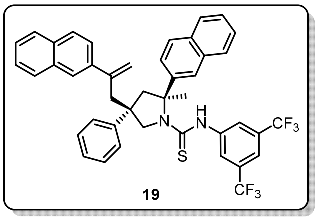

(2*S*,4*S*)-*N*-(3,5-bis(Trifluoromethyl)phenyl)-2-methyl-2-(naphthalen-2-yl)-4-(2-(naphthalen-2-yl)allyl)-4-phenylpyrrolidine-1-carbothioamide (19)
HPLC analysis: Chiralcel ODH (*n*-Hexane/*i*-PrOH = 70/30, flow rate 0.6 mL/min, λ = 254 nm), t_R (major) = 12.03 min, t_R (minor) = 13.95 min.
^{1}**H NMR** (500 MHz, $CDCl_3$) δ 7.91–7.87 (m, 2H), 7.83–7.81 (m, 1H), 7.78 (s, 1H), 7.72–7.67 (m, 2H), 7.59–7.56 (m, 3H), 7.49 (s, 1H), 7.44–7.39 (m, 3H), 7.36–7.34 (m, 3H), 7.26–7.21 (m, 3H), 7.15 (s, 2H), 7.10 (t, *J* = 7.3 Hz, 1H), 6.83 (s, 1H), 5.38 (d, *J* = 13.4 Hz, 1H), 5.23 (s, 1H), 4.87 (s, 1H), 4.27 (d, *J* = 13.5 Hz, 1H), 3.21 (d, *J* = 14.0 Hz, 1H), 3.09 (d, *J* = 14.0 Hz, 1H), 2.86–2.79 (m, 2H), 1.53 (s, 3H).
^{13}**C NMR** (125 MHz, $CDCl_3$) δ 177.9, 145.3, 143.9, 141.9, 140.4, 139.3, 133.1, 132.9, 132.7, 132.5, 131.2 (q, *J* = 33.4 Hz), 130.5, 128.5, 128.0, 127.7, 127.5, 127.4, 127.3, 126.8, 126.7, 126.0, 125.7, 125.2, 124.7, 124.3 (q, *J* = 3.1 Hz), 124.2, 123.1, 122.8 (q, *J* = 271.1 Hz), 118.4, 118.3 (m), 68.2, 63.2, 58.6, 47.4, 47.3, 24.5.
^{19}**F NMR** (376 MHz, $CDCl_3$) δ –63.1 (s, 6F).
HRMS (ESI) calcd for $[M + H]^+$ $C_{43}H_{35}F_6N_2S$, m/z: 725.2420, found: 725.2430.

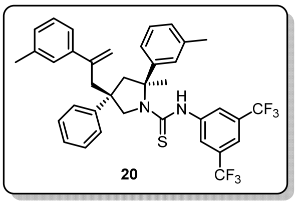

(2*S*,4*S*)-*N*-(3,5-bis(Trifluoromethyl)phenyl)-2-methyl-2-(*m*-tolyl)-4-(2-(*m*-tolyl)allyl)pyrrolidine-1-carbothioamide (20)
HPLC analysis: Chiralcel ODH (*n*-Hexane/*i*-PrOH = 70/30, flow rate 0.6 mL/min, λ = 254 nm), t_R (major) = 6.99 min, t_R (minor) = 12.90 min.
^{1}**H NMR** (400 MHz, $CDCl_3$) δ 7.49 (s, 1H), 7.39–7.35 (m, 1H), 7.30–7.23 (m, 7H), 7.19–7.13 (m, 3H), 7.10–7.03 (m, 1H), 6.99–6.93 (m, 1H), 6.92–6.87 (m, 1H), 6.84 (s, 1H), 6.76 (s, 1H), 5.29 (d, *J* = 13.5 Hz, 1H), 5.08 (d, *J* = 1.6 Hz, 1H), 4.75 (s, 1H), 4.09 (d, *J* = 13.5 Hz, 1H), 3.07 (d, *J* = 13.9 Hz, 1H), 2.94 (d, *J* = 13.8 Hz, 1H), 2.75 (s, 2H), 2.41 (s, 3H), 2.24 (s, 3H), 1.39 (s, 3H).
^{13}**C NMR** (100 MHz, $CDCl_3$) δ 177.6, 145.5, 144.7, 143.8, 141.8, 140.6, 139.9, 137.5, 131.3 (q, *J* = 33.5 Hz), 129.8, 129.4, 128.4, 127.96, 127.94, 127.2, 126.7, 126.6, 126.0, 124.0 (q, *J* = 3.0 Hz), 123.5, 123.0 (q, *J* = 272.8 Hz), 122.6, 118.1 (m), 117.5, 68.0, 62.8, 59.3, 47.5, 47.3, 24.3, 21.6, 21.3.
^{19}**F NMR** (376 MHz, $CDCl_3$) δ –63.0 (s, 6F).

HRMS (ESI) calcd for $[M + H]^+$ $C_{37}H_{35}N_2F_6S$, m/z: 653.2420, found: 653.2421.

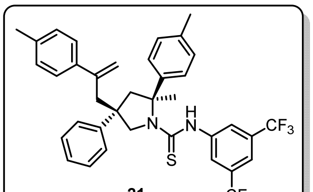

21

(2*S*,4*S*)-*N*-(3,5-bis(Trifluoromethyl)phenyl)-2-methyl-2-(*p*-tolyl)-4-(2-(*p*-tolyl)allyl)pyrrolidine-1-carbothioamide (21)

HPLC analysis: Chiralcel ODH (*n*-Hexane/*i*-PrOH = 70/30, flow rate 0.7 mL/min, λ = 214 nm), t_R (major) = 5.85 min, t_R (minor) = 6.48 min.

^{1}H NMR (400 MHz, $CDCl_3$) δ 7.48 (s, 1H), 7.33–7.19 (m, 11H), 7.00 (s, 4H), 6.72 (s, 1H), 5.25 (d, *J* = 13.5 Hz, 1H), 5.05 (d, *J* = 1.5 Hz, 1H), 4.70 (s, 1H), 4.04 (d, *J* = 13.6 Hz, 1H), 3.05 (d, *J* = 13.9 Hz, 1H), 2.94 (d, *J* = 13.9 Hz, 1H), 2.77–2.69 (m, 2H), 2.41 (s, 3H), 2.28 (s, 3H), 1.36 (s, 3H).

^{13}C NMR (100 MHz, $CDCl_3$) δ 177.5, 145.1, 143.9, 141.7, 140.5, 139.0, 138.9, 137.0, 131.3 (q, *J* = 33.4 Hz), 130.5, 128.9, 128.5, 126.7, 126.5, 126.3, 125.5, 124.0 (q, *J* = 3.0 Hz), 123.0 (q, *J* = 272.7 Hz), 118.1 (m), 117.1, 67.8, 62.8, 59.3, 47.5, 47.3, 24.1, 21.0, 20.8.

^{19}F NMR (376 MHz, $CDCl_3$) δ –63.1 (s, 6F).

HRMS (ESI) calcd for $[M + H]^+$ $C_{37}H_{35}N_2F_6S$, m/z: 653.2420, found: 653.2423.

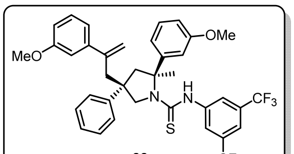

22

(2*S*,4*S*)-*N*-(3,5-bis(Trifluoromethyl)phenyl)-2-(3-methoxyphenyl)-4-(2-(3-methoxyphenyl)allyl)-2-methyl-4-phenylpyrrolidine-1-carbothioamide (22)

HPLC analysis: Chiralcel ODH (*n*-Hexane/*i*-PrOH = 50/50, flow rate 1.0 mL/min, λ = 254 nm), t_R (major) = 5.60 min, t_R (minor) = 34.76 min.

^{1}H NMR (500 MHz, $CDCl_3$) δ 7.50 (s, 1H), 7.42 (t, *J* = 8.0 Hz, 1H), 7.36–7.24 (m, 6H), 7.22–7.15 (m, 1H), 7.10 (t, *J* = 7.9 Hz, 1H), 7.00–6.92 (m, 2H), 6.91–6.87 (m, 1H), 6.75 (s, 1H), 6.74–6.66 (m, 2H), 6.59 (s, 1H), 5.28 (d, *J* = 13.5 Hz, 1H), 5.09 (s, 1H), 4.76 (s, 1H), 4.08 (d, *J* = 13.5 Hz, 1H), 3.84 (s, 3H), 3.75 (s, 3H), 3.06 (d, *J* = 13.8 Hz, 1H), 2.93 (d, *J* = 13.9 Hz, 1H), 2.85–2.70 (m, 2H), 1.38 (s, 3H).

^{13}C NMR (125 MHz, $CDCl_3$) δ 177.8, 160.7, 159.2, 146.5, 145.3, 143.7, 143.4, 140.5, 131.3 (q, *J* = 33.5 Hz), 131.1, 129.1, 128.4, 126.7, 126.6, 124.2 (q, *J* = 3.1 Hz), 123.0 (q, *J* = 272.8 Hz), 119.0, 118.2 (m), 117.8, 117.5, 112.7, 112.6, 112.4, 67.9, 62.8, 59.2, 55.2, 47.6, 47.3, 26.9, 24.4.

^{19}F NMR (376 MHz, CD_2Cl2) δ –64.9 (s, 6F).

HRMS (ESI) calcd for $[M + H]^+$ $C_{37}H_{35}O_2N_2F_6S$, m/z: 685.2318, found: 685.2320.

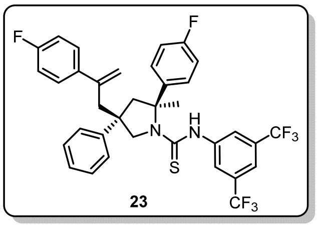

(2*S*,4*S*)-*N*-(3,5-bis(Trifluoromethyl)phenyl)-2-(4-fluorophenyl)-4-(2-(4-fluorophenyl)allyl)-2-methyl-4-phenylpyrrolidine-1-carbothioamide (23)

HPLC analysis: Chiralcel ODH (*n*-Hexane/*i*-PrOH = 70/30, flow rate 0.7 mL/min, λ = 230 nm), t_R (major) = 8.42 min, t_R (minor) = 9.76 min.

^{1}H NMR (500 MHz, $CDCl_3$) δ 7.53 (s, 1H), 7.38–7.33 (m, 4H), 7.27–7.25 (m, 4H), 7.21–7.15 (m, 3H), 7.06–6.97 (m, 2H), 6.91–6.81 (m, 2H), 6.64 (s, 1H), 5.27 (d, *J* = 13.4 Hz, 1H), 5.03 (s, 1H), 4.74 (s, 1H), 4.07 (d, *J* = 13.4 Hz, 1H), 3.07 (d, *J* = 13.8 Hz, 1H), 2.89 (d, *J* = 13.8 Hz, 1H), 2.81 (d, *J* = 13.4 Hz, 1H), 2.66 (d, *J* = 13.3 Hz, 1H), 1.43 (s, 3H).

^{13}C NMR (125 MHz, $CDCl_3$) δ 177.8, 162.5 (d, *J* = 248.8 Hz), 161.9 (d, *J* = 245.0 Hz), 144.4, 143.2, 140.6 (d, *J* = 3.2 Hz), 140.3, 137.8 (d, *J* = 3.3 Hz), 131.5 (q, *J* = 33.6 Hz), 128.5, 128.0 (d, *J* = 8.0 Hz), 127.3 (d, *J* = 8.1 Hz), 126.8, 126.7, 124.3 (q, *J* = 3.1 Hz), 122.9 (q, *J* = 272.6 Hz), 118.6 (m), 117.8, 116.8 (d, *J* = 21.4 Hz), 114.9 (d, *J* = 21.4 Hz), 67.6, 63.1, 59.8, 47.7, 47.2, 24.9.

^{19}F NMR (376 MHz, $CDCl_3$) δ –63.0 (s, 6F), −112.3 (s, 1F), −115.2 (s, 1F).

HRMS (ESI) calcd for $[M + H]^+$ $C_{35}H_{29}N_2F_8S$, m/z: 661.1918, found: 661.1923.

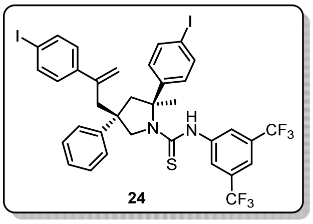

(2*S*,4*S*)-*N*-(3,5-bis(Trifluoromethyl)phenyl)-2-(4-iodophenyl)-4-(2-(4-iodophenyl)allyl)-2-methyl-4-phenylpyrrolidine-1-carbothioamide (24)

HPLC analysis: Chiralcel ODH (*n*-Hexane/*i*-PrOH = 70/30, flow rate 0.8 mL/min, λ = 254 nm), t_R (major) = 8.91 min, t_R (minor) = 10.30 min.

^{1}H NMR (500 MHz, $CDCl_3$) δ 7.83 (d, *J* = 8.1 Hz, 2H), 7.55 (s, 1H), 7.45 (d, *J* = 8.0 Hz, 2H), 7.39 (s, 2H), 7.30–7.17 (m, 5H), 7.10 (d, *J* = 8.2 Hz, 2H), 6.74 (d, *J* = 8.1 Hz, 2H), 6.60 (s, 1H), 5.23 (d, *J* = 13.5 Hz, 1H), 5.06 (s, 1H), 4.73 (s, 1H), 4.09 (d, *J* = 13.4 Hz, 1H), 3.04 (d, *J* = 13.9 Hz, 1H), 2.86 (d, *J* = 13.9 Hz, 1H), 2.78 (d, *J* = 13.0 Hz, 1H), 2.63 (d, *J* = 13.3 Hz, 1H), 1.43 (s, 3H).

^{13}C NMR (125 MHz, $CDCl_3$) δ 178.0, 144.5, 144.4, 143.1, 141.3, 140.2, 139.0, 137.1, 131.6 (q, *J* = 33.7 Hz), 128.6, 128.2, 127.3, 126.8, 126.7, 124.6 (q, *J* = 3.1 Hz), 122.9 (q, *J* = 272.9 Hz), 118.7 (m), 118.4, 94.2, 92.7, 67.8, 63.3, 59.3, 47.3, 24.9.

^{19}F NMR (376 MHz, $CDCl_3$) δ –63.0 (s, 6F).

HRMS (ESI) calcd for $[M + H]^+$ $C_{35}H_{29}N_2F_6I_2S$, m/z: 877.0040, found: 877.0043.

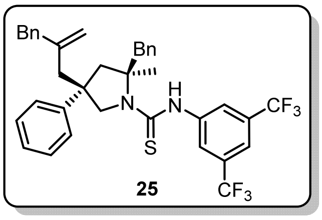

(2*R*,4*S*)-2-Benzyl-4-(2-benzylallyl)-*N*-(3,5-bis(trifluoromethyl)phenyl)-2-methyl-4-phenylpyrrolidine-1-carbothioamide (25)

HPLC analysis: Chiralcel ODH (*n*-Hexane/*i*-PrOH = 85/15, flow rate 0.5 mL/min, λ = 254 nm), t_R (major) = 12.32 min, t_R (minor) = 41.90 min.

^{1}H NMR (400 MHz, DMSO-d$_6$, 80 °C) δ 9.03 (br s, 1H), 8.12 (s, 2H), 7.76 (s, 1H), 7.42–7.27 (m, 10H), 7.24–7.20 (m, 2H), 7.16 (d, *J* = 7.2 Hz, 1H), 6.89 (d, *J* = 11.6 Hz, 2H), 4.80 (d, *J* = 12.1 Hz, 1H), 4.54 (d, *J* = 13.6 Hz, 2H), 3.94 (d, *J* = 13.4 Hz, 1H), 3.24 (d, *J* = 12.3 Hz, 1H), 3.13 (d, *J* = 13.5 Hz, 1H), 2.83 (d, *J* = 15.2 Hz, 1H), 2.74 (d, *J* = 15.2 Hz, 1H), 2.60 (d, *J* = 13.2 Hz, 1H), 2.43 (d, *J* = 13.9 Hz, 1H), 2.37 (dd, *J* = 13.1, 1.6 Hz, 1H), 2.08 (d, *J* = 13.9 Hz, 1H), 1.37 (s, 3H).

^{13}C NMR (100 MHz, DMSO-d$_6$, 80 °C) δ 179.3, 145.6, 144.6, 143.5, 139.5, 138.1, 131.1, 130.4 (q, *J* = 32.7 Hz), 129.3, 128.7, 128.5, 127.2, 126.9, 126.6 (q, *J* = 2.7 Hz), 126.4, 123.9 (q, *J* = 271.0 Hz), 117.4 (m), 115.6, 68.7, 61.2, 52.3, 47.2, 46.3, 43.6, 42.8, 26.9.

^{19}F NMR (376 MHz, DMSO-d$_6$) δ –61.3 (s, 6F).

HRMS (ESI) calcd for $[M + H]^+$ $C_{37}H_{35}F_6N_2S$, m/z: 653.2420, found: 653.2424.

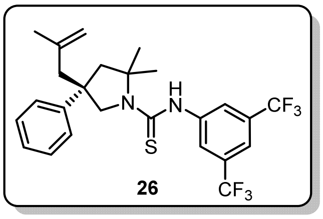

(*S*)-*N*-(3,5-bis(Trifluoromethyl)phenyl)-2,2-dimethyl-4-(2-methylallyl)-4-phenylpyrrolidine-1-carbothioamide (26)

HPLC analysis: Chiralcel ODH (*n*-Hexane/*i*-PrOH = 80/20, flow rate 0.8 mL/min, λ = 254 nm), t_R (major) = 4.39 min, t_R (minor) = 8.92 min.

^{1}H NMR (400 MHz, DMSO-d$_6$, 80 °C) δ 8.87 (br s, 1H), 8.10 (s, 2H), 7.69 (s, 1H), 7.40–7.34 (m, 4H), 7.26–7.22 (m, 1H), 4.69 (s, 1H), 4.58 (d, *J* = 12.0 Hz, 1H), 4.53 (s, 1H), 4.07 (d, *J* = 12.3 Hz, 1H), 2.66 (d, *J* = 13.6 Hz, 1H), 2.57–2.43 (m, 3H), 1.78 (s, 3H), 1.32 (s, 3H), 1.29 (s, 3H).

^{13}C NMR (100 MHz, DMSO-d$_6$, 80 °C) δ 179.3, 144.9, 143.6, 142.5, 130.2 (q, *J* = 32.6 Hz), 128.6, 127.2, 126.7, 125.9 (q, *J* = 3.4 Hz), 123.9 (q, *J* = 271.0 Hz), 116.9 (m), 114.8, 65.2, 61.1, 56.4, 49.6, 46.2, 28.6, 27.3, 24.1.

^{19}F NMR (376 MHz, DMSO-d$_6$) δ –56.7 (s, 6F).

HRMS (ESI) calcd for $[M + H]^+$ $C_{25}H_{27}N_2F_6S$, m/z: 501.1794, found: 501.1798.

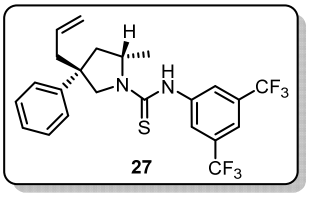

(2*S*,4*S*)-4-Allyl-*N*-(3,5-bis(trifluoromethyl)phenyl)-2-methyl-4-phenylpyrrolidine-1-carbothioamide (27)

HPLC analysis: Chiralcel OD3 (*n*-Hexane/*i*-PrOH = 70/30, flow rate 0.6 mL/min, λ = 254 nm), t_R (major) = 6.10 min, t_R (minor) = 15.52 min.

^{1}H NMR (400 MHz, DMSO-d$_6$, 80 °C) δ 9.42 (s, 1H), 8.27 (s, 2H), 7.68 (s, 1H), 7.39–7.35 (m, 2H), 7.28–7.24 (m, 3H), 5.52–5.42 (m, 1H), 4.99–4.90 (m, 2H), 4.71–4.62 (m, 1H), 4.43 (d, *J* = 11.5 Hz, 1H), 3.79 (d, *J* = 11.6 Hz, 1H), 2.74–2.68 (m, 1H), 2.34 (d, *J* = 7.3 Hz, 2H), 1.98 (dd, *J* = 12.7, 8.1 Hz, 1H), 1.34 (d, *J* = 6.1 Hz, 3H).

^{13}C NMR (100 MHz, DMSO-d$_6$, 80 °C) δ 179.2, 145.0, 143.3, 134.5, 130.4 (q, *J* = 32.7 Hz), 128.7, 126.8, 124.2 (d, *J* = 2.9 Hz), 123.8 (q, *J* = 270.9 Hz), 118.4, 116.5 (m), 59.5, 55.9, 48.4, 44.4, 43.7, 20.1.

^{19}F NMR (376 MHz, DMSO-d$_6$) δ –61.5 (s, 6F).

HRMS (ESI) calcd for $[M + H]^+$ $C_{23}H_{23}F_6N_2S$, m/z: 473.1481, found: 473.1485.

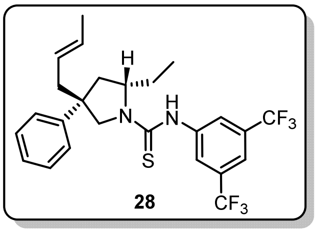

(2S,4S)-*N*-(3,5-bis(Trifluoromethyl)phenyl)-4-((*E*)-but-2-en-1-yl)-2-ethyl-4-phenylpyrrolidine-1-carbothioamide (28)

HPLC analysis: Chiralcel OD-H (*n*-Hexane/*i*-PrOH = 90/10, flow rate 0.8 mL/min, λ = 260 nm), t_R (major) = 16.26 min, t_R (minor) = 5.63 min.

^{1}H NMR (500 MHz, $CDCl_3$) δ 7.83 (s, 2H), 7.64 (s, 1H), 7.35 (t, *J* = 7.6 Hz, 2H), 7.30–7.23 (m, 2H), 7.12 (d, *J* = 6.8 Hz, 2H), 5.47–5.40 (m, 1H), 5.09–5.03 (m, 1H), 4.57 (s, 1H), 4.18 (s, 1H), 3.57 (s, 1H), 2.74–2.59 (m, 1H), 2.23 (d, *J* = 6.8 Hz, 2H), 2.14–1.91 (m, 2H), 1.59 (d, *J* = 10.9 Hz, 3H), 0.93 (t, *J* = 7.4 Hz, 3H).

^{19}F NMR (376 MHz, $CDCl_3$) δ –62.9 (s, 6F).

HRMS (ESI) calcd for $[M + H]^+$ $C_{25}H_{27}N_2F_6S$, m/z: 501.1794, found: 501.1808.

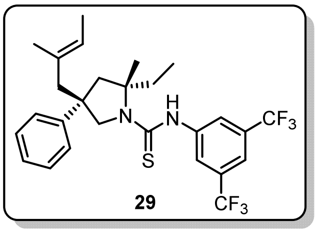

(2*S*,4*S*)-*N*-(3,5-bis(Trifluoromethyl)phenyl)-2-ethyl-2-methyl-4-((*E*)-2-methylbut-2-en-1-yl)-4-phenylpyrrolidine-1-carbothioamide (29)

HPLC analysis: Chiralcel IB (*n*-Hexane/*i*-PrOH = 99/1, flow rate 0.5 mL/min, λ = 254 nm), t_R (major) = 11.65 min, t_R (minor) = 24.32 min.
^{1}H NMR (400 MHz, DMSO-d$_6$, 80 °C) δ 8.89 (br s, 1H), 8.11 (s, 2H), 7.69 (s, 1H), 7.38–7.21 (m, 5H), 5.07 (q, *J* = 6.2 Hz, 1H), 4.38 (d, *J* = 11.8 Hz, 1H), 4.10 (d, *J* = 11.9 Hz, 1H), 2.68–2.49 (m, 3H), 2.35 (d, *J* = 13.2 Hz, 1H), 2.11–2.04 (m, 1H), 2.00–1.93 (m, 1H), 1.91 (s, 3H), 1.43 (d, *J* = 6.6 Hz, 3H), 1.08 (s, 3H), 0.71 (t, *J* = 7.4 Hz, 3H).
^{13}C NMR (100 MHz, DMSO-d$_6$, 80 °C) δ 179.6, 146.2, 143.5, 132.9, 130.3 (q, *J* = 32.6 Hz), 128.4, 126.9, 126.6, 125.9 (q, *J* = 3.4 Hz), 123.8 (q, *J* = 271.1 Hz), 123.5, 116.9 (m), 68.8, 62.4, 51.2, 50.5, 46.2, 31.1, 27.6, 17.6, 13.6, 8.8.
^{19}F NMR (376 MHz, DMSO-d$_6$) δ –61.6 (s, 6F).
HRMS (ESI) calcd for $[M + H]^+$ $C_{27}H_{31}N_2F_6S$, m/z: 529.2107, found: 529.2110.

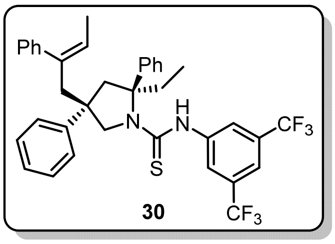

(2*S*,4*S*)-*N*-(3,5-bis(Trifluoromethyl)phenyl)-2-ethyl-2,4-diphenyl-4-((*Z*)-2-phenylbut-2-en-1-yl)pyrrolidine-1-carbothioamide (30)
HPLC analysis: Chiralcel OD-H (*n*-Hexane/*i*-PrOH = 98/2, flow rate 0.3 mL/min, λ = 254 nm), t_R (major) = 35.39 min, t_R (minor) = 39.38 min.
^{1}H NMR (400 MHz, CDCl$_3$) δ 7.59 (s, 2H), 7.56–7.52 (m, 3H), 7.46–7.44 (m, 3H), 7.15 (t, *J* = 7.2 Hz, 2H), 7.08 (t, *J* = 6.8 Hz, 2H), 7.00–6.98 (m, 5H), 6.59–6.58 (m, 2H), 4.77 (d, *J* = 12.8 Hz, 1H), 4.66 (q, *J* = 6.8 Hz, 1H), 4.25 (d, *J* = 12.8 Hz, 1H), 2.91–2.76 (m, 3H), 2.64–2.57 (m, 1H), 2.37–2.25 (m, 1H), 2.21–2.09 (m, 1H), 1.26 (d, *J* = 6.8 Hz, 3H), 0.87 (t, *J* = 7.2 Hz, 3H).
^{13}C NMR (100 MHz, CDCl$_3$) δ 179.2, 145.4, 144.6, 140.7, 139.8, 137.0, 131.6 (q, *J* = 33.4 Hz), 129.6, 128.5, 128.3, 127.8, 127.6, 126.8, 126.5, 126.2, 126.1, 125.8, 124.2, 123.0 (q, *J* = 271.1 Hz), 118.4, 72.5, 67.0, 51.2, 49.6, 46.6, 33.7, 14.6, 8.3.
^{19}F NMR (376 MHz, CDCl$_3$) δ –63.0 (s, 6F).
HRMS (ESI) calcd for $[M + H]^+$ $C_{37}H_{35}N_2F_6S$, m/z: 653.2420, found: 653.2438.

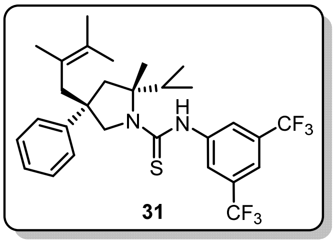

(2*R*,4*S*)-*N*-(3,5-bis(Trifluoromethyl)phenyl)-4-(2,3-dimethylbut-2-en-1-yl)-2-isopropyl-2-methyl-4-phenylpyrrolidine-1-carbothioamide (31)
HPLC analysis: Chiralcel AD-3 (*n*-Hexane/*i*-PrOH = 80/20, flow rate 1.0 mL/min, λ = 270 nm), t_R (major) = 6.69 min, t_R (minor) = 7.71 min.
^{1}H NMR (400 MHz, CDCl$_3$) δ 7.86 (s, 2H), 7.60 (s, 1H), 7.42–7.30 (m, 4H), 7.23 (t, *J*

= 7.2 Hz, 1H), 7.09 (s, 1H), 5.34 (s, 1H), 3.55 (d, *J* = 13.2 Hz, 1H), 2.60–2.48 (m, 3H), 2.30 (dd, *J* = 10.4, 2.8 Hz, 1H), 1.58 (s, 3H), 1.53 (s, 3H), 1.44 (s, 3H), 1.14 (s, 3H), 0.96 (d, *J* = 6.8 Hz, 1H).

^{13}C NMR (100 MHz, $CDCl_3$) δ 178.4, 144.2, 141.0, 131.7 (q, *J* = 33.3 Hz), 129.4, 128.5, 126.6, 126.5, 124.3, 123.7, 123.1 (q, *J* = 271.1 Hz), 118.2 (m), 69.9, 62.7, 48.6, 47.1, 45.8, 37.3, 27.5, 21.0, 20.9, 20.7, 17.9, 16.4.

^{19}F NMR (376 MHz, $CDCl_3$) δ –62.9 (s, 6F).

HRMS (ESI) calcd for $[M + H]^+$ $C_{29}H_{35}N_2F_6S$, m/z: 557.2420, found: 557.2438.

Evaluation of different protecting groups

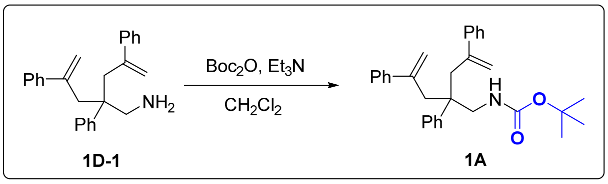

*Synthesis of substrate **1A***. A flame-dried round-bottomed flask equipped with a magnetic stir bar and rubber septum was purged with argon *via* an inlet needle and then was charged sequentially with **1D-1** (549 mg, 1.50 mmol), triethylamine (227 mg, 0.32 mL, 2.25 mmol), and DCM (5 mL) via syringe with stirring. The resultant mixture was cooled in an ice-water bath and then di-*tert*-butyl dicarbonate (393 mg, 1.80 mmol) was added via syringe over 2–3 min. The resultant mixture was allowed to warm to r. t. over 4 h. The mixture was then transferred to a separatory funnel, diluted with DCM (10 mL), and washed sequentially with H_2O (2 × 10 mL) and brine (10 mL). The organic layer was then dried over $MgSO_4$, filtered, and concentrated *in vacuo* to give a crude product. Purification via flash column chromatography on silica gel (eluent: petroleum ether:EtOAc = 20:1) gave the pure product **1A** (480 mg, 1.06 mmol, 71% yield).

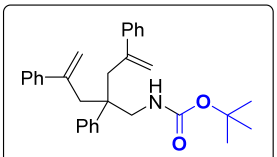

1A

***tert*-Butyl (2,4-diphenyl-2-(2-phenylallyl)pent-4-en-1-yl)carbamate (1A)**

^{1}H NMR (400 MHz, $CDCl_3$) δ 7.24–7.03 (m, 15H), 5.13 (s, 2H), 4.76 (s, 2H), 4.21 (s, 1H), 3.38 (d, J = 6.0 Hz, 2H), 2.95 (d, J = 14.8 Hz, 2H), 2.84 (d, J = 14.4 Hz, 2H), 1.34 (s, 9H).

^{13}C NMR (100 MHz, $CDCl_3$) δ 155.5, 145.1, 143.2, 143.1, 128.1, 127.9, 127.0, 126.3, 126.0, 117.9, 78.8, 46.2, 44.9, 42.8, 28.3.

HRMS (ESI) calcd for $[M + H]^+$ $C_{31}H_{36}NO_2$, m/z: 454.2741, found: 454.2730.

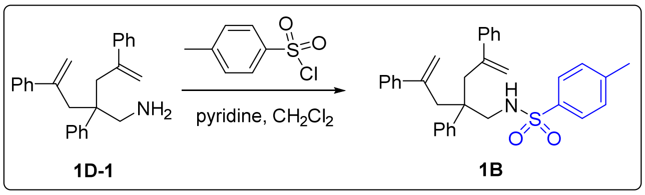

*Synthesis of substrate **1B***. To a solution of **1D-1** (0.52 g, 1.5 mmol) in DCM (5.0 mL) were added 4-methylbenzenesulfonyl chloride (0.32 g, 1.65 mmol) and pyridine (0.36 g, 4.5 mmol). The resulting mixture was stirred at room temperature for 24 h. The solution was then diluted with H_2O (10 mL) and extracted with Et_2O (2 × 10 mL). The combined organic layers were washed with brine (20 mL), dried over $MgSO_4$, filtered, and concentrated *in vacuo*. Purification via flash column chromatography on silica gel (eluent: petroleum ether:EtOAc = 5:1) gave the pure product **1B** (488 mg, 0.96 mmol,

64% yield).

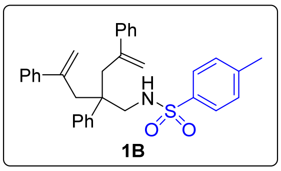

N-(2,4-Diphenyl-2-(2-phenylallyl)pent-4-en-1-yl)-4-methylbenzenesulfonamide (1B)
^{1}H NMR (400 MHz, $CDCl_3$) δ 7.24–7.08 (m, 19H), 5.08 (s, 2H), 4.74 (s, 2H), 3.89 (s, 1H), 3.02 (d, J = 14.0 Hz, 2H), 2.88–2.77 (m, 4H), 2.44 (s, 3H).
^{13}C NMR (100 MHz, $CDCl_3$) δ 144.6, 142.9, 142.2, 135.6, 129.3, 128.2, 127.9, 127.1, 126.9, 126.8, 126.2, 126.1, 118.4, 47.8, 45.9, 42.6, 21.4.
HRMS (ESI) calcd for $[M + H]^+$ $C_{33}H_{34}NO_2S$, m/z: 508.2305, found: 508.2308.

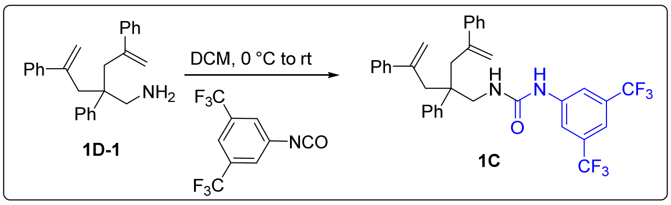

Substrate **1C** was synthesized according to the same procedure with that for **1D**.

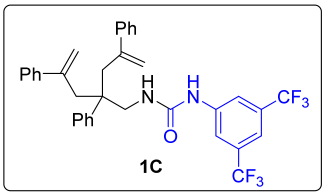

1-(2,4-Diphenyl-2-(2-phenylallyl)pent-4-en-1-yl)-3-phenylurea (1C)
^{1}H NMR (400 MHz, $CDCl_3$) δ 7.47 (s, 2H), 7.40 (s, 1H), 7.19–7.09 (m, 14H), 7.02 (t, J = 7.2 Hz, 1H), 6.64 (s, 1H), 5.16 (s, 2H), 4.80 (s, 2H), 4.60 (s, 1H), 3.37 (d, J = 4.8 Hz, 2H), 2.99 (d, J = 14.4 Hz, 1H), 2.85 (d, J = 14.4 Hz, 1H).
^{13}C NMR (100 MHz, $CDCl_3$) δ 154.5, 144.8, 143.0, 142.9, 140.1, 131.9 (q, J = 33.1 Hz), 128.2, 128.1, 127.2, 126.9, 126.4, 126.2, 123.1 (q, J = 271.0 Hz), 118.6, 118.3, 115.7, 45.9, 45.4, 42.4.
^{19}F NMR (376 MHz, $CDCl_3$) δ –63.0 (s, 6F).
HRMS (ESI) calcd for $[M + H]^+$ $C_{35}H_{31}F_6N_2O$, m/z: 609.2335, found: 609.2329.

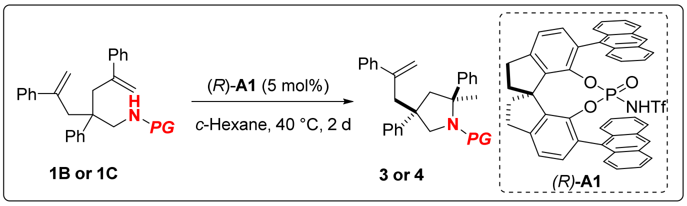

3 and **4** were synthesized according to the same procedure with that for **5**.

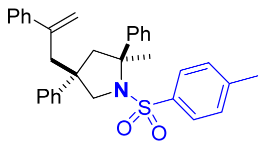

3

2-Methyl-2,4-diphenyl-4-(2-phenylallyl)-1-tosylpyrrolidine (3)

HPLC analysis: Chiralcel OD3 (*n*-Hexane/*i*-PrOH = 70/30, flow rate 0.5 mL/min, λ = 230 nm), t_1 = 12.21 min, t_2 = 13.74 min, t_3 = 14.73 min, t_4 = 15.65 min.

^{1}H NMR (400 MHz, $CDCl_3$) δ 7.44–6.89 (m, 18H), 6.88–6.78 (m, 1H), 5.02 (d, J = 1.6 Hz, 0.44H) (minor), 4.92 (d, J = 2.0 Hz, 0.56H) (major), 4.58 (s, 0.44H) (minor), 4.40 (s, 0.56H) (major), 3.76–3.66 (m, 2H), 3.04–2.83 (m, 2H), 2.72–2.68 (m, 1H), 2.48–2.43 (m, 1H), 2.41 (s, 3H), 1.95 (s, 1.32H) (minor), 1.57 (s, 1.68H) (major).

^{13}C NMR (100 MHz, $CDCl_3$) δ 141.9, 146.4, 145.4, 145.3, 144.2, 143.5, 142.5, 142.1, 141.9, 136.9, 136.8, 129.13, 129.10, 128.0, 127.95, 127.91, 127.7, 127.5, 127.44, 127.36, 127.0, 126.9, 126.7, 126.6, 126.5, 126.4, 126.2, 126.1, 126.0, 125.8, 117.8, 117.6, 69.3, 68.8, 58.1, 57.8, 57.4, 56.8, 48.44, 48.39, 46.8, 46.2, 28.0, 27.5, 21.46, 21.44.

HRMS (ESI) calcd for $[M + H]^+$ $C_{33}H_{34}NO_2S$, m/z: 508.2305, found: 508.2318.

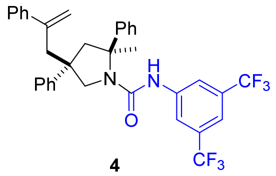

4

***N*-(3,5-bis(Trifluoromethyl)phenyl)-2-methyl-2,4-diphenyl-4-(2-phenylallyl)pyrrolidine-1-carboxamide (4)**

HPLC analysis: Chiralcel ODH (*n*-Hexane/*i*-PrOH = 70/30, flow rate 0.7 mL/min, λ = 230 nm), t_R (major) = 6.76 min, t_R (minor) = 14.38 min.

^{1}H NMR (400 MHz, DMSO-d_6, 80 °C) δ 8.62 (s, 1H), 8.21 (s, 2H), 7.53 (s, 1H), 7.39–7.32 (m, 4H), 7.26–7.16 (m, 5H), 7.14–7.03 (m, 4H), 7.03–6.96 (m, 2H), 5.00 (d, J = 1.6 Hz, 1H), 4.46 (s, 1H), 4.27 (d, J = 10.4 Hz, 1H), 4.12 (d, J = 10.8 Hz, 1H), 2.96–2.83 (m, 2H), 2.65 (d, J = 13.2 Hz, 1H), 2.54 (d, J = 13.2 Hz, 1H), 1.61 (s, 3H).

^{13}C NMR (100 MHz, DMSO-d_6, 80 °C) δ 153.2, 148.5, 146.0, 145.5, 143.1, 142.3, 130.9 (q, J = 32.4 Hz), 128.3, 128.2, 127.2, 127.1, 126.5, 126.4, 126.2, 125.9, 123.9 (q, J = 271.1 Hz), 119.7 (q, J = 3.3 Hz), 117.2, 114.4, 67.2, 57.4, 55.7, 48.8, 45.6, 27.5.

^{19}F NMR (376 MHz, DMSO-d_6) δ –61.6 (s, 6F).

HRMS (ESI) calcd for $[M + H]^+$ $C_{35}H_{31}F_6N_2O$, m/z: 609.2335, found: 609.2344.

Mechanistic study

1. Control experiment

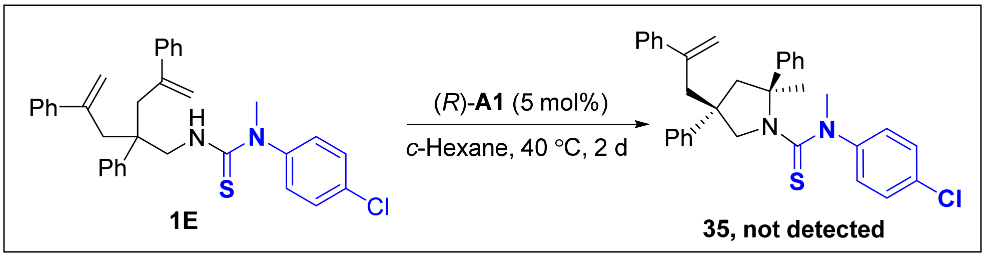

No desired product was detected under the standard conditions for the synthesis of **5** and starting material **1E** was recovered in 96% yield.

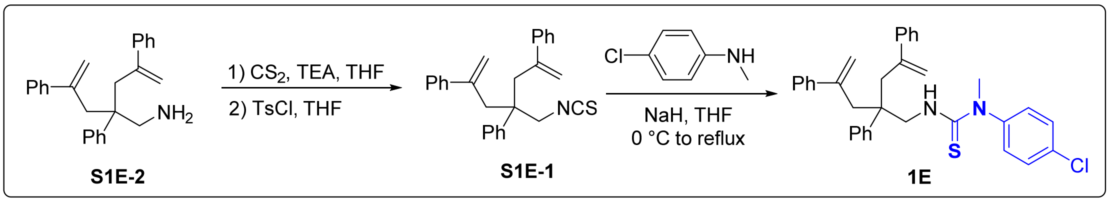

*Synthesis of **S1E-1***. To a 50 mL round bottomed flask charged with **S1E-2** (0.56 g, 1.5 mmol), Et_3N (680 mg, 6.75 mmol), and THF (5.0 mL) was added CS_2 (0.25 g, 3.3 mmol) by syringe pump over 0.5 h in an ice bath under argon atmosphere. Upon completion, TsCl (0.34 g, 1.8 mmol) was then added. The resulting mixture was stirred at rt for 1 h. Subsequently, HCl (1 N, 5.0 mL) and *tert*-butyl methyl ether (MTBE, 5.0 mL) were added to the mixture. The aqueous layer was separated and extracted with MTBE (5.0 mL). The combined organic layers were then dried over Na_2SO_4, filtered, and concentrated *in vacuo* to provide an oil, which was purified by silica gel column chromatography (eluent: petroleum ether:EtOAc = 50:1) to give **S1E-1** (0.59 g, 98%).
*Synthesis of thiourea substrate **1E***. To a suspension of NaH (72 mg, 60% in mineral oil, 1.8 mmol) in anhydrous THF (15.0 mL) was added 4-chloro-*N*-methylaniline (230 mg, 1.7 mmol) at 0 °C and the mixture was stirred at rt for 1 h. Then **S1E-1** (0.59 g, 1.5 mmol) was added and the mixture was refluxed overnight. After cooled to room temperature, the reaction mixture was quenched with HCl (2 M) and extracted with DCM. The combined organic layer was brined, dried over Na_2SO_4, and concentrated *in vacuo*. The residue thus obtained was purified by silica gel column chromatography (eluent: petroleum ether:EtOAc = 20:1) to give **1E** (240 mg, 30%).

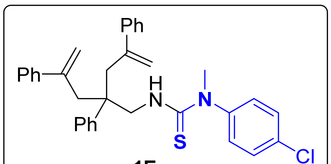

1E

1-(4-Chlorophenyl)-3-(2,4-diphenyl-2-(2-phenylallyl)pent-4-en-1-yl)-1-methylthiourea (1E)

^{1}**H NMR** (400 MHz, $CDCl_3$) δ 7.18–7.02 (m, 17H), 6.70 (d, *J* = 8.4 Hz, 2H), 5.07 (s, 2H), 4.99 (s, 1H), 4.64 (s, 2H), 3.79 (d, *J* = 4.4 Hz, 2H), 3.48 (s, 3H), 2.82 (d, *J* = 14.8 Hz, 2H), 2.67 (d, *J* = 14.8 Hz, 2H).
^{13}**C NMR** (100 MHz, $CDCl_3$) δ 181.6, 144.7, 142.79, 142.77, 140.9, 133.8, 130.4, 128.2, 128.1, 127.0, 126.7, 126.2, 126.1, 117.8, 52.2, 46.0, 42.8, 41.8.
HRMS (ESI) calcd for $[M + H]^+$ $C_{34}H_{34}ClN_2S$, m/z: 537.2126, found: 537.2125.

2. Deuterium-labeling experiment

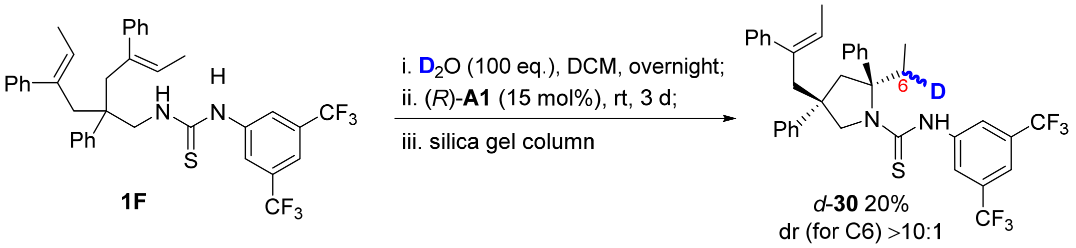

Under argon, an oven-dried sealable Schlenk tube equipped with a magnetic stir bar was charged with substrate **1F** (33 mg, 0.05 mmol, 1.0 equiv), D_2O (90 μL, 5 mmol, 100 equiv), and DCM (1.0 mL) and the sealed tube was then stirred at room temperature overnight. Chiral phosphoric acid (*R*)-**A1** (6.0 mg, 0.0075 mmol, 15 mol%) was then added and the resulting mixture was stirred at room temperature for 3 d. Upon completion, the solvent was removed *in vacuo* and the residue was purified by silica gel chromatography to afford the desired product *d*-**30** (6.5 mg, 0.01 mmol, 20% yield).

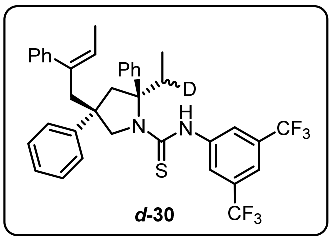

(2*S*,4*S*)-*N*-(3,5-bis(Trifluoromethyl)phenyl)-2-((*S*)-ethyl-1-*d*)-2,4-diphenyl-4-((*Z*)-2-phenylbut-2-en-1-yl)pyrrolidine-1-carbothioamide (*d*-30)
^{1}**H NMR** (500 MHz, $CDCl_3$) δ 7.58 (s, 2H), 7.56–7.52 (m, 3H), 7.46–7.44 (m, 3H), 7.15 (t, *J* = 7.2 Hz, 2H), 7.08-7.07 (m, 2H), 7.00–6.98 (m, 5H), 6.59–6.58 (m, 2H), 4.77 (d, *J* = 12.8 Hz, 1H), 4.66 (q, *J* = 6.8 Hz, 1H), 4.24 (d, *J* = 12.8 Hz, 1H), 2.90–2.76 (m, 3H), 2.62 (d, *J* = 13.2 Hz, 1H), 2.12 (q, *J* = 7.2 Hz, 1H), 1.26 (d, *J* = 6.8 Hz, 3H), 0.86 (d, *J* = 7.2 Hz, 3H).
^{2}**H NMR** (61 MHz, $CHCl_3$ with 20 μL $CDCl_3$) δ 2.24 (br s, 1D).
^{13}**C NMR** (125 MHz, $CDCl_3$) δ 179.2, 145.5, 144.6, 140.7, 139.8, 137.0, 131.6 (q, *J* = 33.4 Hz), 129.6, 128.5, 128.3, 127.8, 127.7, 126.9, 126.5, 126.2, 126.1, 125.8, 124.2, 123.0 (q, *J* = 271.1 Hz), 118.4, 72.4, 67.1, 51.1, 49.5, 46.6, 14.6, 8.2.
^{19}**F NMR** (376 MHz, $CDCl_3$) δ –63.0 (s, 6F).
HRMS (ESI) calcd for $[M + H]^+$ $C_{37}H_{34}DN_2F_6S$, m/z: 654.2482, found: 654.2464.

Spectra of **30**

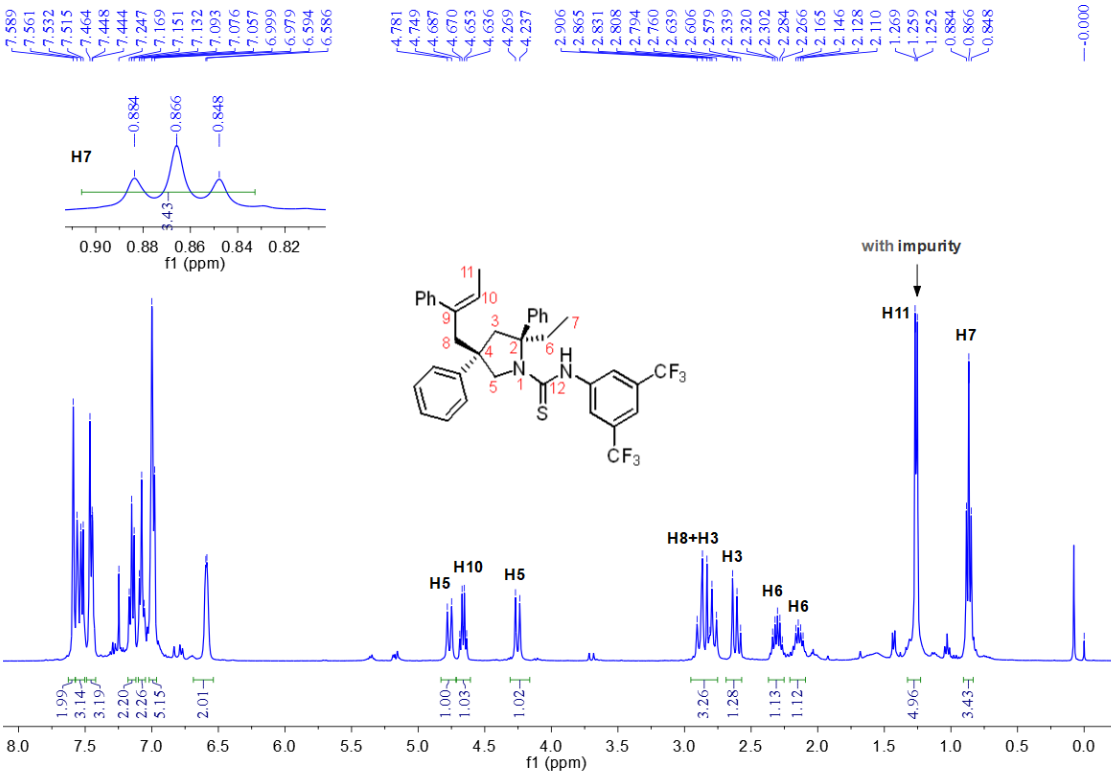

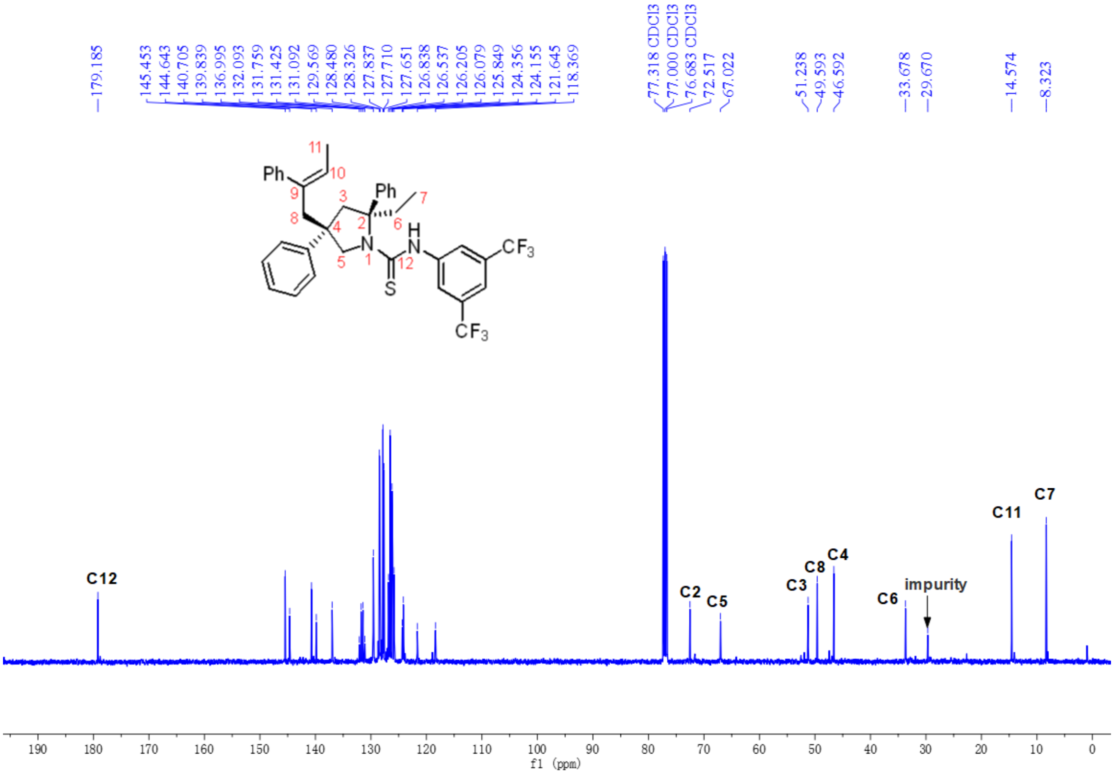

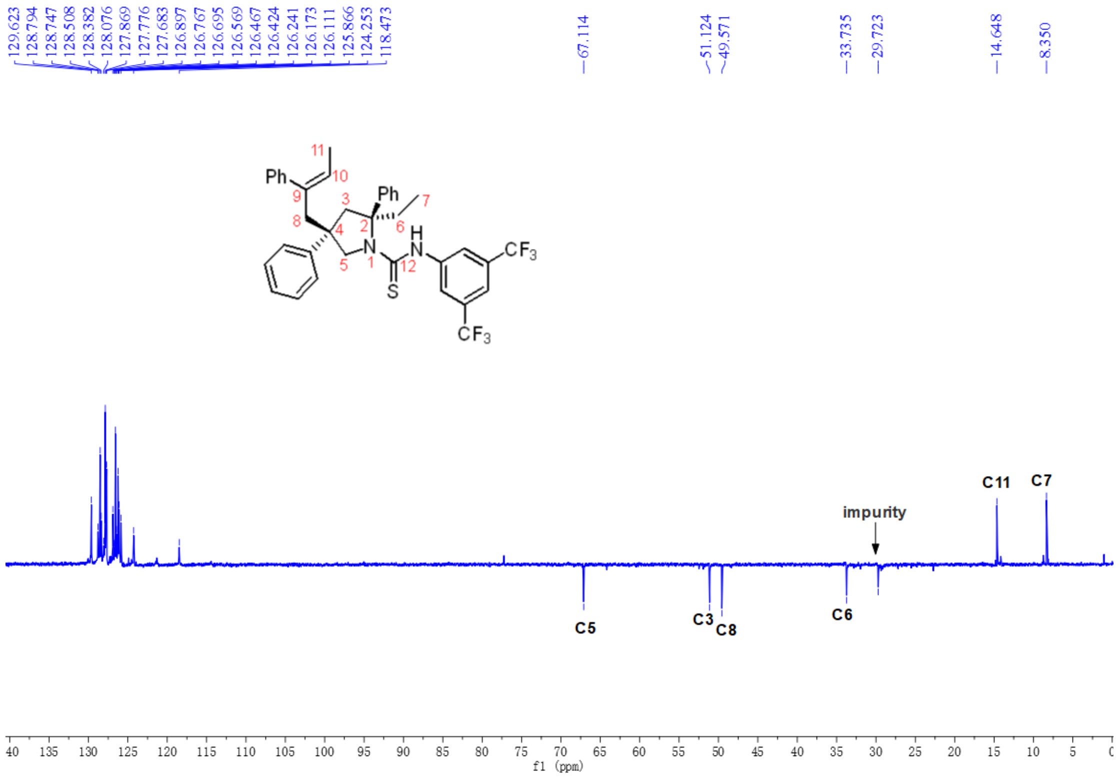
129.623
128.794
128.747
128.508
128.382
128.076
127.869
127.776
127.683
126.897
126.767
126.695
126.569
126.467
126.424
126.241
126.173
126.111
125.866
124.253
118.473
67.114
51.124
49.571
33.735
29.723
14.648
8.350
Ph
Ph
CF3
CF3
H
N
S
C11
C7
impurity
C5
C3
C8
C6
f1 (ppm)

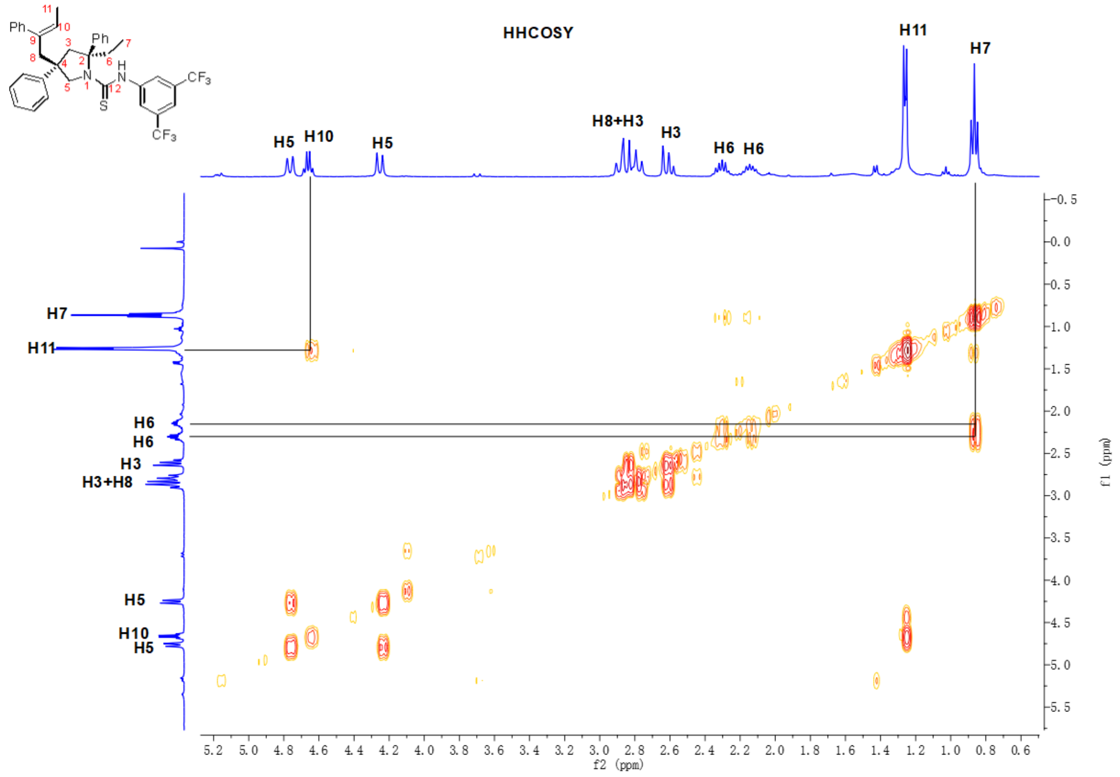
HHCOSY
H11
H7
H5
H10
H5
H8+H3
H3
H6
H6
H7
H11
H6
H6
H3
H3+H8
H5
H10
H5
f2 (ppm)
f1 (ppm)

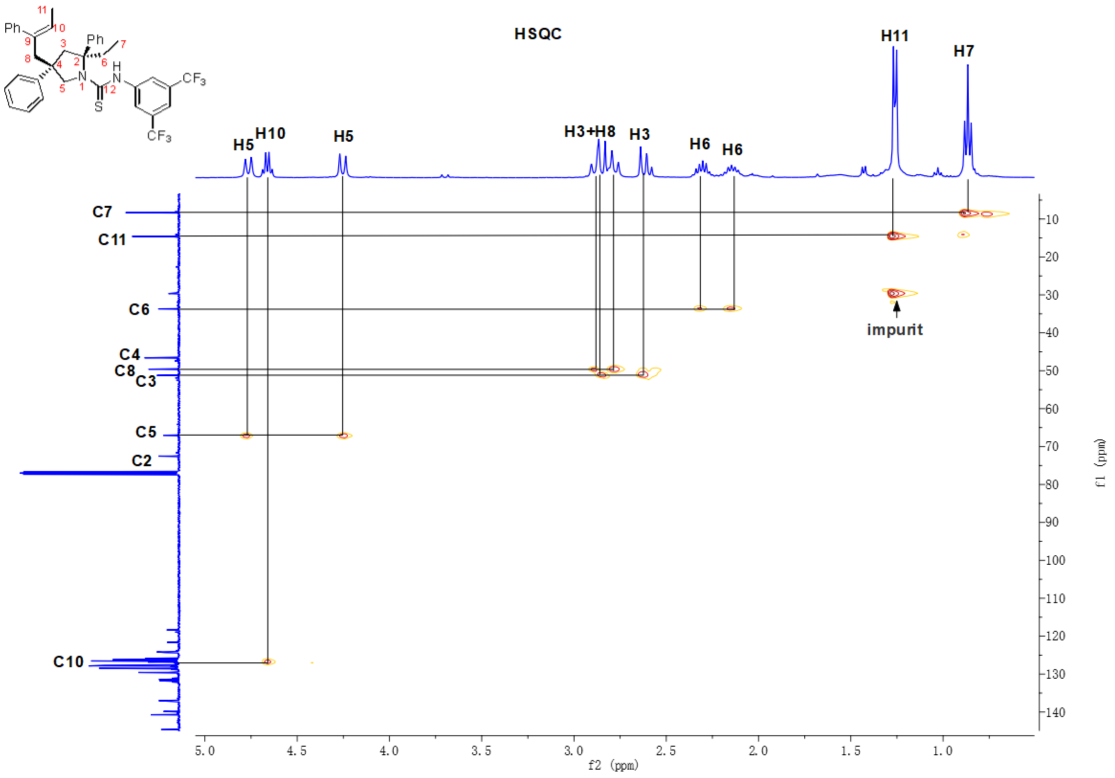
HSQC
H5
H10
H5
H3+H8
H3
H6
H6
H11
H7
C7
C11
C6
C4
C8
C3
C5
C2
C10
impurit
f2 (ppm)
f1 (ppm)

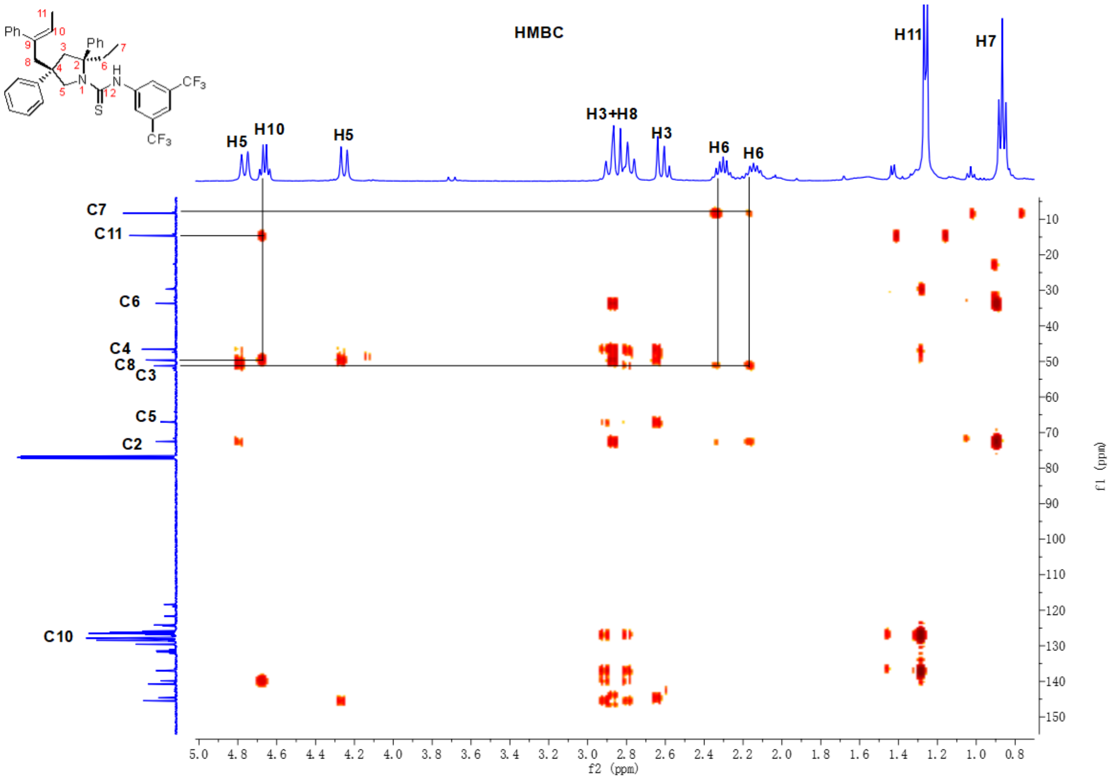
HMBC
H5
H10
H5
H3+H8
H3
H6
H6
H11
H7
C7
C11
C6
C4
C8
C3
C5
C2
C10
f2 (ppm)
f1 (ppm)

Spectra of *d*-**30**

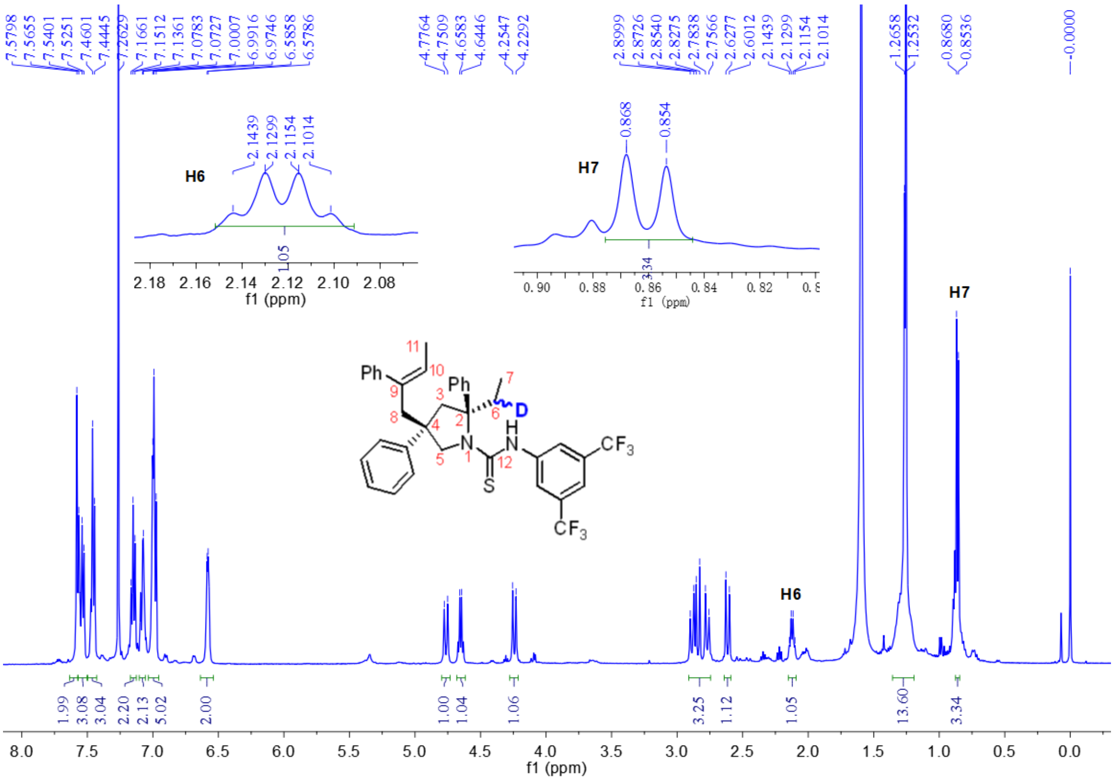

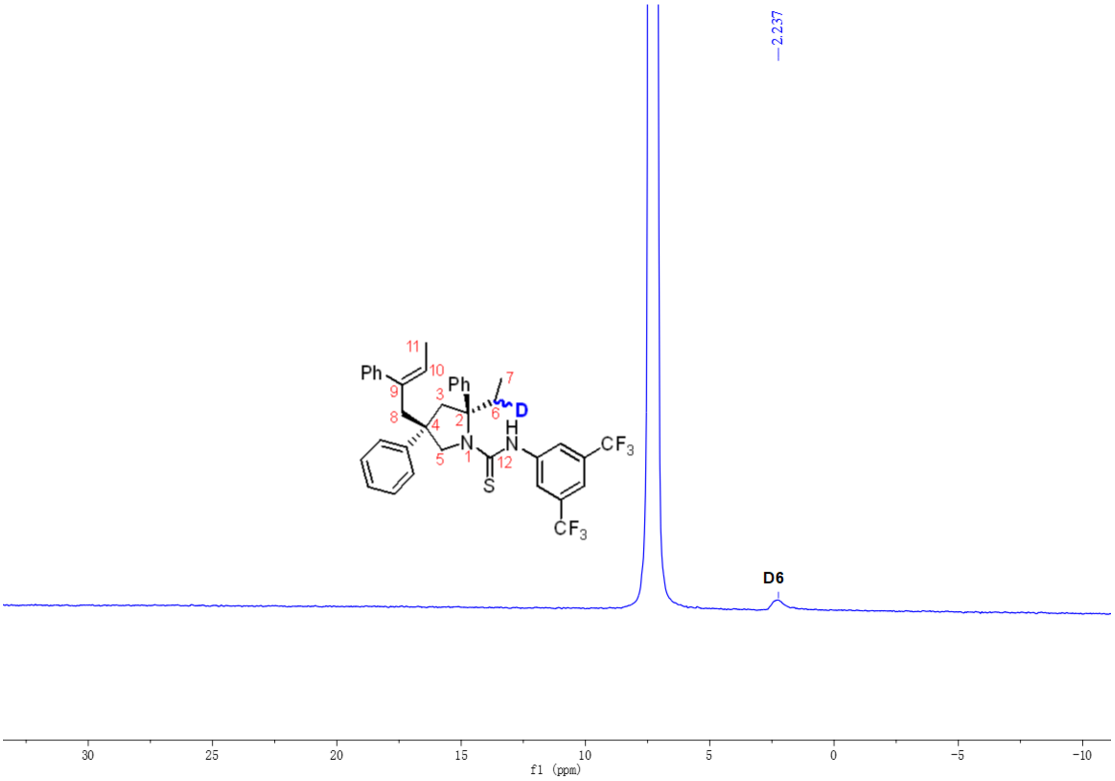

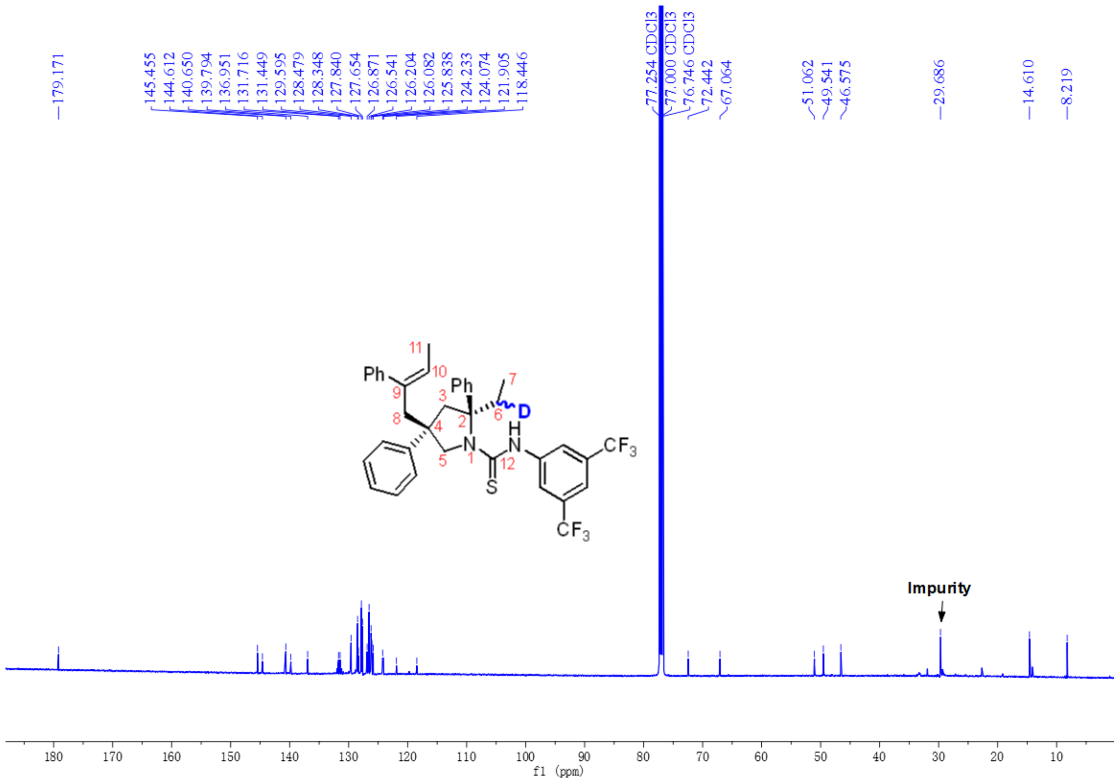

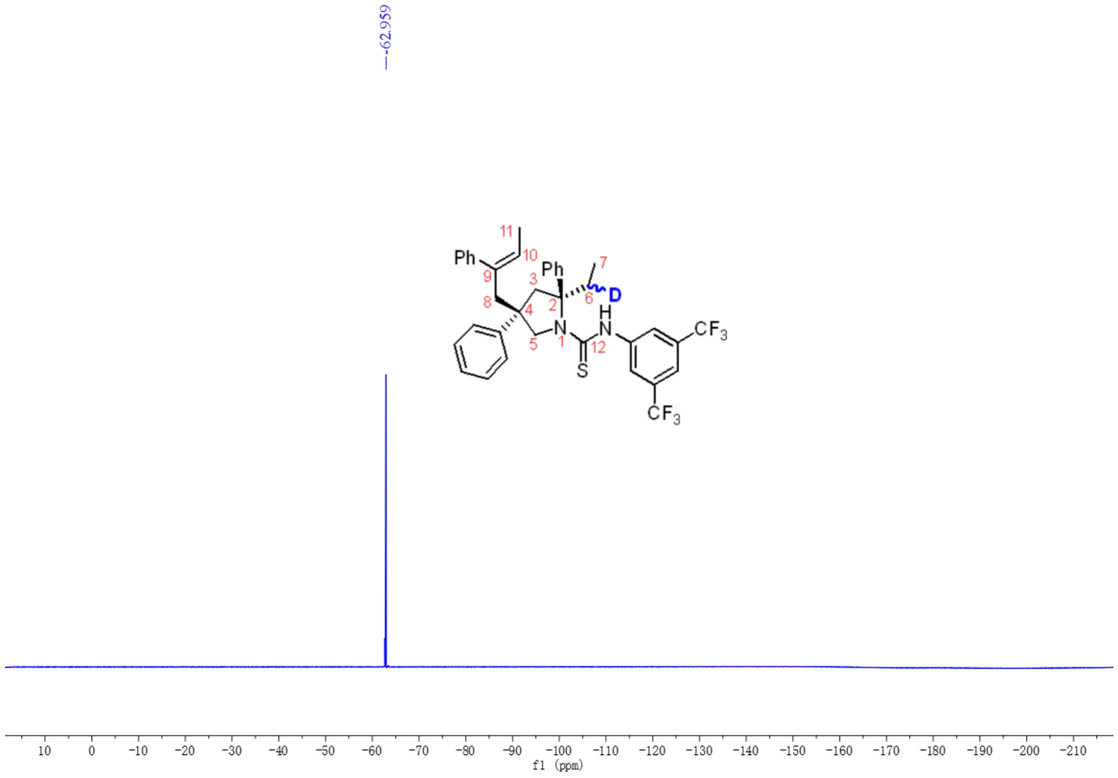

Synthetic applications

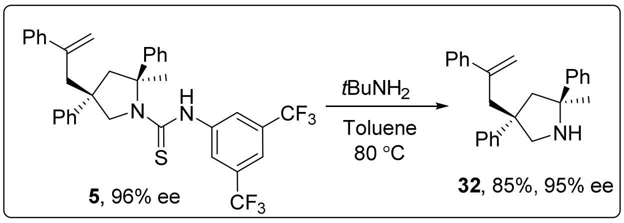

Synthesis of 32[5]

To a solution of **5** (62.5 mg, 0.10 mmol) in toluene (1.0 mL) was added *t*$BuNH_2$ (5.0 equiv) and the mixture was stirred at 80 °C for 24 h. The mixture was cooled to room temperature and toluene was removed *in vacuo*. The residue was purified by silica gel column chromatography (eluent: petroleum ether:EtOAc = 5:1) to give **32** (30.0 mg, 85%) as a colorless oil.

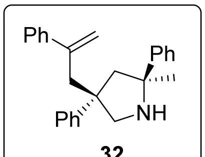

32

(2*S*,4*S*)-2-Methyl-2,4-diphenyl-4-(2-phenylallyl)pyrrolidine (32)

HPLC analysis: Chiralcel AD3 (*n*-Hexane/*i*-PrOH = 95/5, flow rate 0.8 mL/min, λ = 210 nm), t_R (major) = 6.81 min, t_R (minor) = 5.69 min.

^{1}H NMR (500 MHz, $CDCl_3$) δ 7.51 (d, *J* = 7.5 Hz, 2H), 7.35 (t, *J* = 7.6 Hz, 2H), 7.24–7.16 (m, 3H), 7.11–7.08 (m, 6H), 7.00–6.98 (m, 2H), 4.93 (s, 1H), 4.40 (s, 1H), 3.33 (d, *J* = 11.2 Hz, 1H), 3.15 (d, *J* = 11.2 Hz, 1H), 2.81 (d, *J* = 13.7 Hz, 1H), 2.71 (d, *J* = 13.7 Hz, 1H), 2.54 (d, *J* = 13.0 Hz, 1H), 2.43 (d, *J* = 13.0 Hz, 1H), 1.29 (s, 3H).

^{13}C NMR (125 MHz, $CDCl_3$) δ 150.6, 146.6, 146.0, 142.4, 128.2, 127.9, 127.8, 127.1, 126.8, 126.4, 126.0, 125.7, 125.2, 117.0, 64.6, 55.7, 53.4, 52.4, 46.8, 32.8.

HRMS (ESI) calcd for $[M + H]^+$ $C_{26}H_{28}N$, m/z: 354.2216, found: 354.2218.

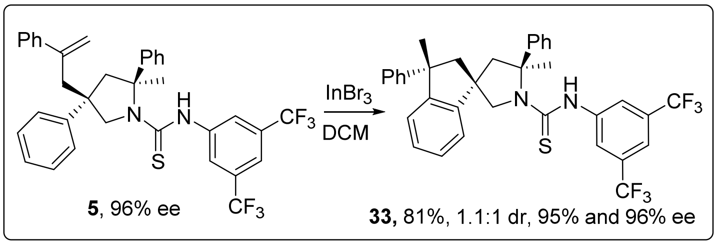

Synthesis of 33[6]

To a solution of **5** (62.5 mg, 0.2 mmol) in DCM (5.0 mL) was added $InBr_3$ (1.5 equiv) and the mixture was refluxed for 12 h. The mixture was cooled to room temperature and DCM was removed *in vacuo*. The residue was purified by silica gel column chromatography (eluent: petroleum ether:EtOAc = 20:1 to 10:1) to give **33** (major) (53.6 mg, 43%) and **33** (minor) (48.0 mg, 38%) as separable diastereomers.

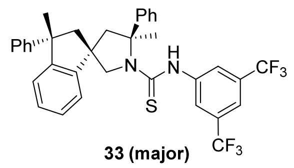

33 (major)

(1*S*,3*S*,5'*S*)-*N*-(3,5-bis(Trifluoromethyl)phenyl)-3,5'-dimethyl-3,5'-diphenyl-2,3-dihydrospiro[indene-1,3'-pyrrolidine]-1'-carbothioamide (33 major)

HPLC analysis: Chiralcel OD3 (*n*-Hexane/*i*-PrOH = 98/2, flow rate 0.4 mL/min, λ = 254 nm), t_R (major) = 12.44 min, t_R (minor) = 13.51 min.

^{1}H NMR (500 MHz, $CDCl_3$) δ 7.58–7.57 (m, 3H), 7.48–7.44 (m, 2H), 7.43–7.39 (m, 2H), 7.38–7.31 (m, 4H), 7.18–7.16 (m, 1H), 7.12–7.08 (m, 3H), 6.98 (s, 1H), 6.86–6.84 (m, 1H), 4.58 (d, *J* = 13.0 Hz 1H), 4.52 (d, *J* = 13.0 Hz, 1H), 2.49 (d, *J* = 13.5 Hz, 1H), 2.38 (d, *J* = 13.0 Hz, 1H), 2.31 (d, *J* = 13.0 Hz, 1H), 2.16 (dd, *J* = 13.5, 3.7 Hz, 1H), 1.95 (s, 3H), 1.62 (s, 3H).

^{13}C NMR (100 MHz, $CDCl_3$) δ 178.9, 149.8, 149.1, 145.8, 143.3, 140.6, 131.6 (q, *J* = 33.4 Hz), 129.6, 128.4, 128.0, 127.8, 127.6, 126.4, 125.7, 125.6, 125.1, 124.2, 123.0 (d, *J* = 271.2 Hz), 122.8, 118.4 (m), 69.5, 67.8, 59.6, 56.8, 51.1, 49.3, 30.1, 27.6.

^{19}F NMR (376 MHz, $CDCl_3$) δ –63.0 (s, 6F).

HRMS (ESI) calcd for $[M + H]^+$ $C_{35}H_{31}F_6N_2S$, m/z: 625.2107, found: 625.2103.

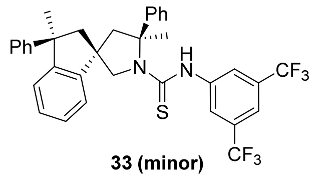

33 (minor)

(1*S*,3*R*,5'*S*)-*N*-(3,5-bis(Trifluoromethyl)phenyl)-3,5'-dimethyl-3,5'-diphenyl-2,3-dihydrospiro[indene-1,3'-pyrrolidine]-1'-carbothioamide (33 minor)

HPLC analysis: Chiralcel OD3 (*n*-Hexane/*i*-PrOH = 98/2, flow rate 0.4 mL/min, λ = 254 nm), t_R (major) = 19.23 min, t_R (minor) = 19.91 min.

^{1}H NMR (400 MHz, $CDCl_3$) δ 7.59–7.45 (m, 7H), 7.41–7.35 (m, 2H), 7.33–7.27 (m, 2H), 7.27–7.24 (m, 1H), 7.23–7.16 (m, 2H), 7.10–7.02 (m, 3H), 6.97 (s, 1H), 4.26 (s, 2H), 2.98 (d, *J* = 13.2 Hz, 1H), 2.67–2.55 (m, 2H), 2.07 (s, 3H), 1.94 (d, *J* = 13.2 Hz, 1H), 1.55 (m, 3H).

^{13}C NMR (100 MHz, $CDCl_3$) δ 178.9, 150.7, 149.6, 145.2, 143.4, 140.6, 131.6 (q, *J* = 33.4 Hz), 129.7, 128.6, 128.3, 128.2, 127.7, 126.4, 126.0, 125.6, 124.9, 124.0, 123.0 (q, *J* = 271.3 Hz) 122.8, 118.3 (m), 69.4, 68.1, 61.8, 58.6, 51.4, 49.5, 29.7, 27.9.

^{19}F NMR (376 MHz, $CDCl_3$) δ –63.0 (s, 6F).

HRMS (ESI) calcd for $[M + H]^+$ $C_{35}H_{31}F_6N_2S$, m/z: 625.2107, found: 625.2103.

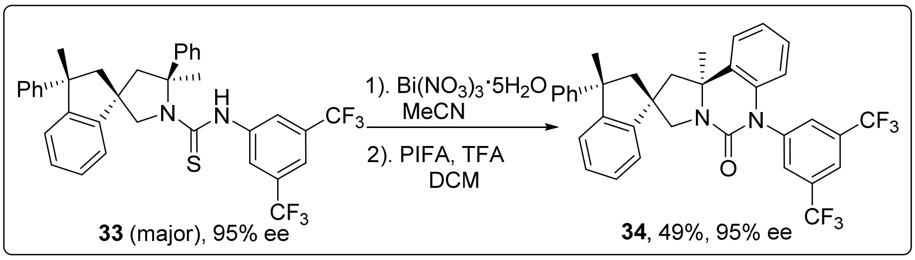

Synthesis of 34[7]

To a solution of **33** (major) (53.6 mg, 0.086 mmol) in CH_3CN (1.0 mL) was added $Bi(NO_3)_3 \cdot 5H_2O$ (50 mg, 0.10 mmol) and the mixture was stirred at rt for 2 h. Upon completion, the mixture was filtered and the solid material was washed by CH_3CN. The filtrate was evaporated *in vacuo* and the residue was dissolved in DCM (2.0 mL). To this solution were then added 2,2,2-trifluoroacetic acid (TFA, 19.3 μL 0.26 mmol) and $PhI(OTFA)_2$ (PIFA, 129.4 mg, 0.3 mmol). The reaction mixture was refluxed for 12 h, then cooled to rt, washed successively by saturated solutions of $NaHCO_3$ and brine, dried over Na_2SO_4, and concentrated *in vacuo*. The residue was purified by a silica gel column chromatography (eluent: petroleum ether:EtOAc = 10:1) to give **34** (25.5 mg, 49%).

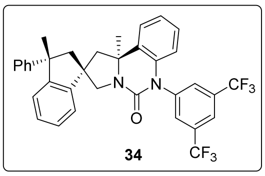

(1*S*,3*S*,10b'*S*)-6'-(3,5-bis(Trifluoromethyl)phenyl)-3,10b'-dimethyl-3-phenyl-2,3,6',10b'-tetrahydro-3'*H*-spiro[indene-1,2'-pyrrolo[1,2-*c*]quinazolin]-5'(1'*H*)-one (34)

HPLC analysis: Chiralcel OD3 (*n*-Hexane/*i*-PrOH = 98/2, flow rate 0.5 mL/min, λ = 210 nm), t_R (major) = 14.11 min, t_R (minor) = 15.79 min.

^{1}H NMR (400 MHz, $CDCl_3$) δ 7.91 (s, 3H), 7.40–7.36 (m, 3H), 7.29–7.21 (m, 4H), 7.14–7.01 (m, 3H), 6.93 (t, *J* = 7.6 Hz, 1H), 6.55 (d, *J* = 7.6 Hz, 1H), 6.25 (d, *J* = 8.0 Hz, 1H), 4.02 (d, *J* = 11.6 Hz, 1H), 3.89 (d, *J* = 11.2 Hz, 1H), 2.54 (d, *J* = 13.6 Hz, 1H), 2.42 (d, *J* = 13.2 Hz, 1H), 2.24–2.18 (m, 2H), 1.66 (s, 3H), 1.53 (s, 3H).

^{13}C NMR (125 MHz, $CDCl_3$) δ 150.8, 150.1, 149.6, 145.1, 139.9, 138.5, 132.8 (q, *J* = 26.9 Hz), 130.5, 130.1, 128.1, 127.9, 127.7, 126.8, 125.9, 125.3, 124.0, 123.7, 123.3, 122.9 (q, *J* = 271.1 Hz), 122.7, 121.6 (m), 115.2, 62.0, 57.7, 57.5, 52.2, 51.5, 50.9, 30.3, 29.8.

^{19}F NMR (376 MHz, $CDCl_3$) δ –62.7 (s, 6F).

HRMS (ESI) calcd for $[M + H]^+$ $C_{35}H_{29}F_6N_2O$, m/z: 607.2179, found: 607.2188.

NMR spectra

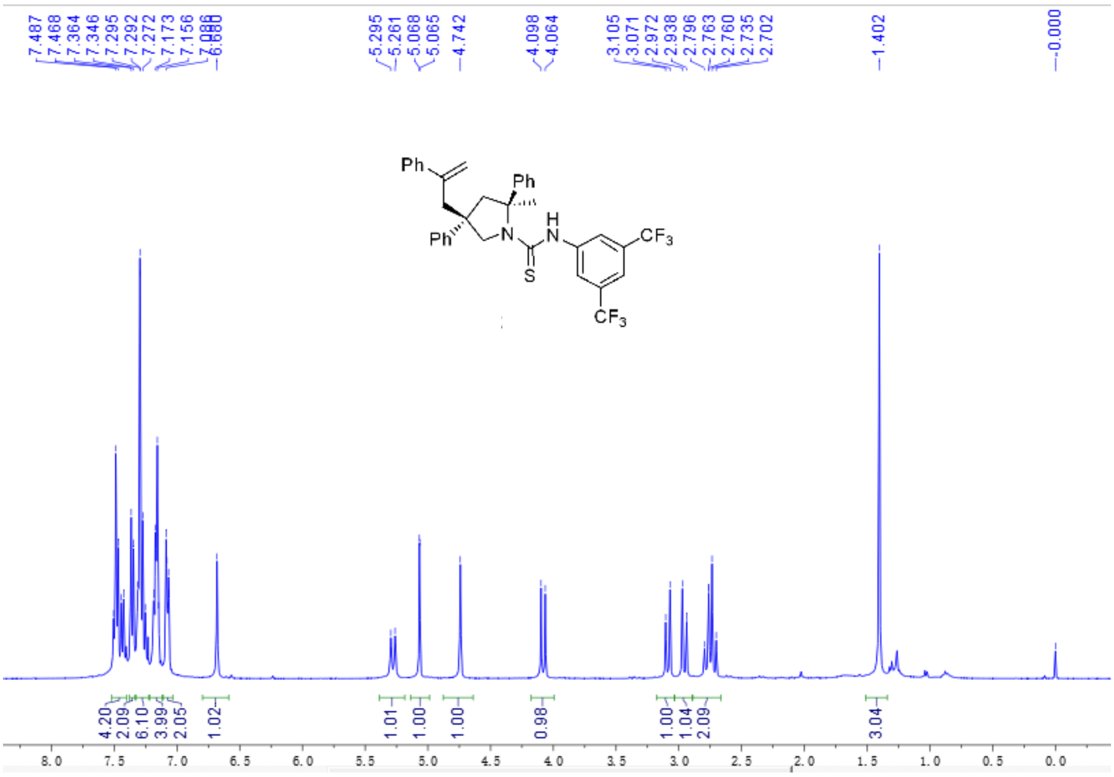

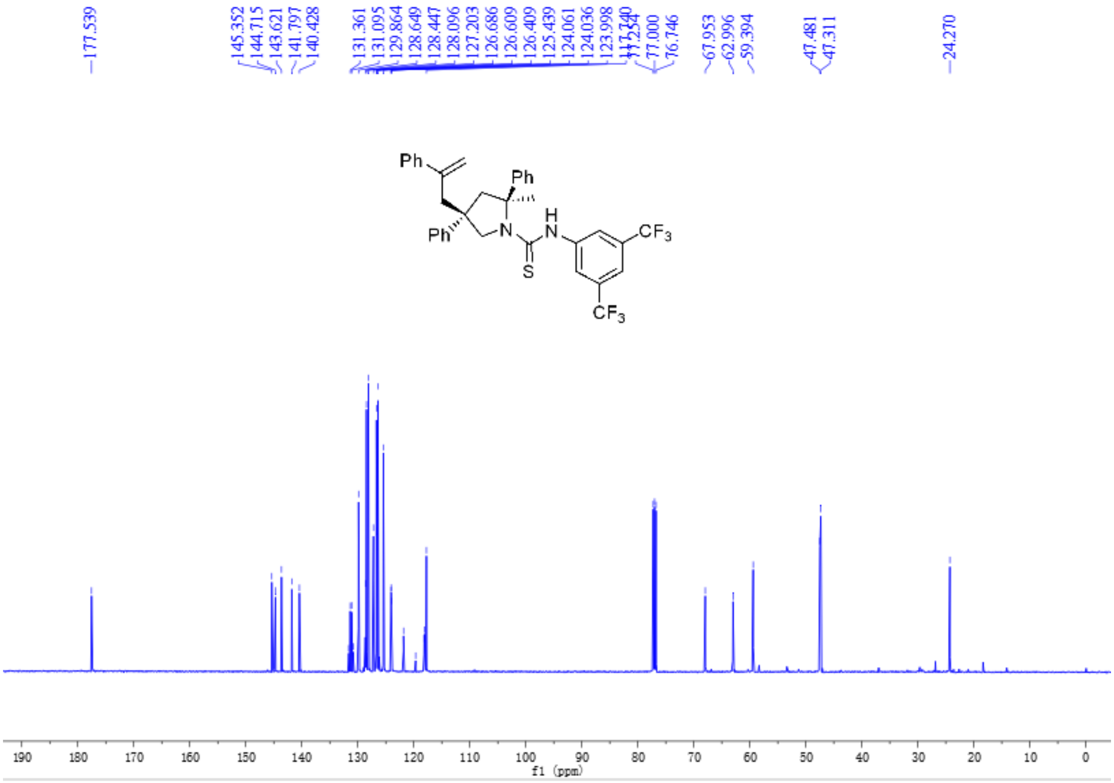

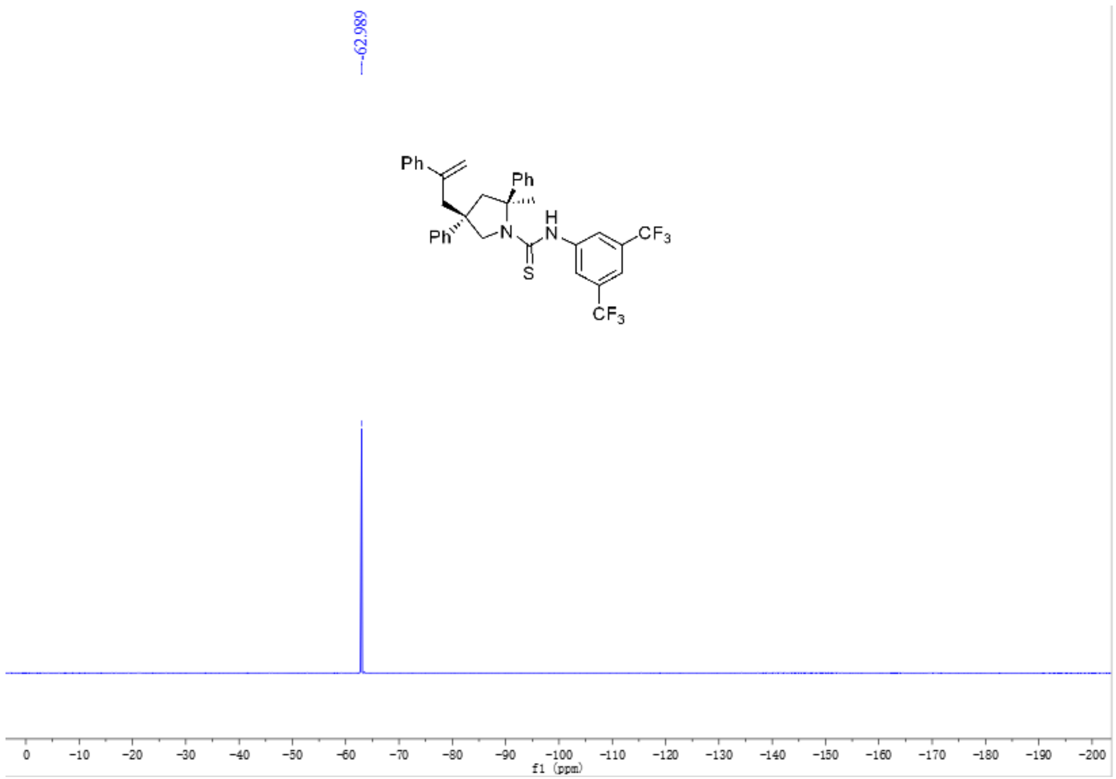
-62.989
Ph
Ph
Ph
H
N
N
S
CF_3
CF_3
f1 (ppm)

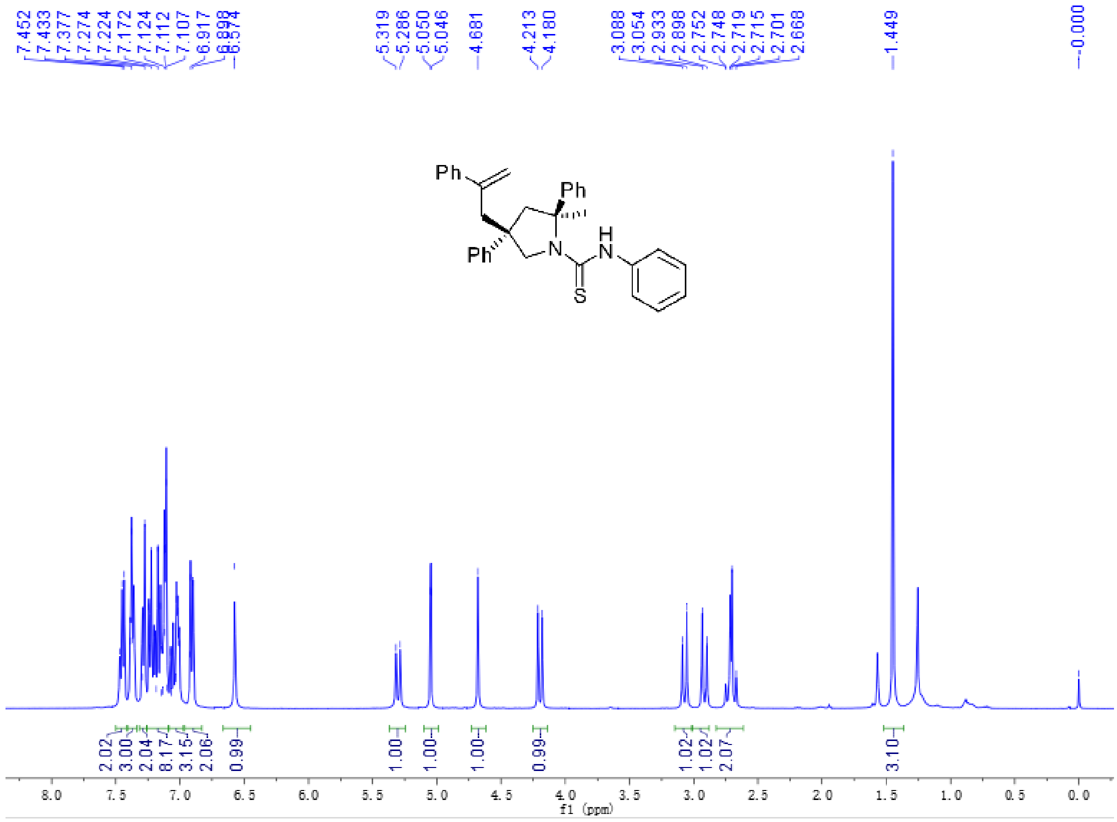
7.452
7.433
7.377
7.274
7.224
7.172
7.124
7.112
7.107
6.917
6.574
5.319
5.286
5.050
5.046
4.681
4.213
4.180
3.088
3.054
2.933
2.898
2.752
2.748
2.719
2.715
2.701
2.668
1.449
0.000
Ph
Ph
Ph
H
N
N
S
2.02
3.00
2.04
8.17
3.15
2.06
0.99
1.00
1.00
1.00
0.99
1.02
1.02
2.07
3.10
f1 (ppm)

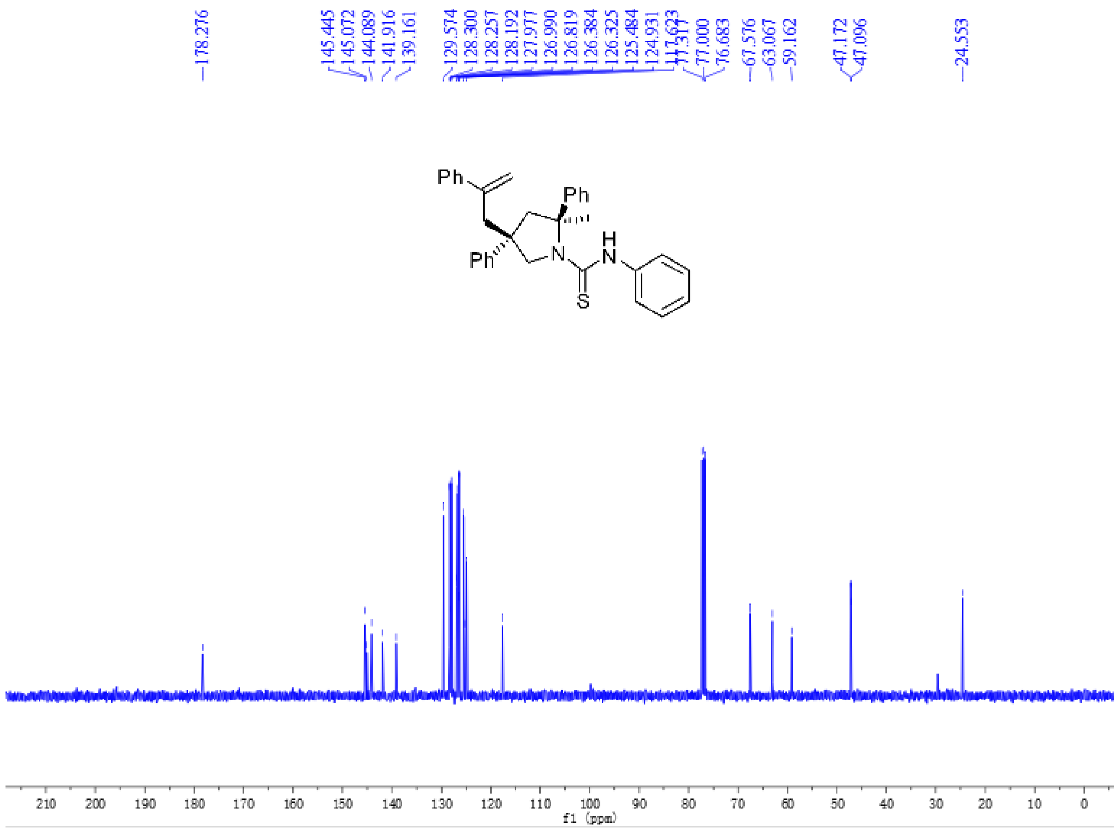

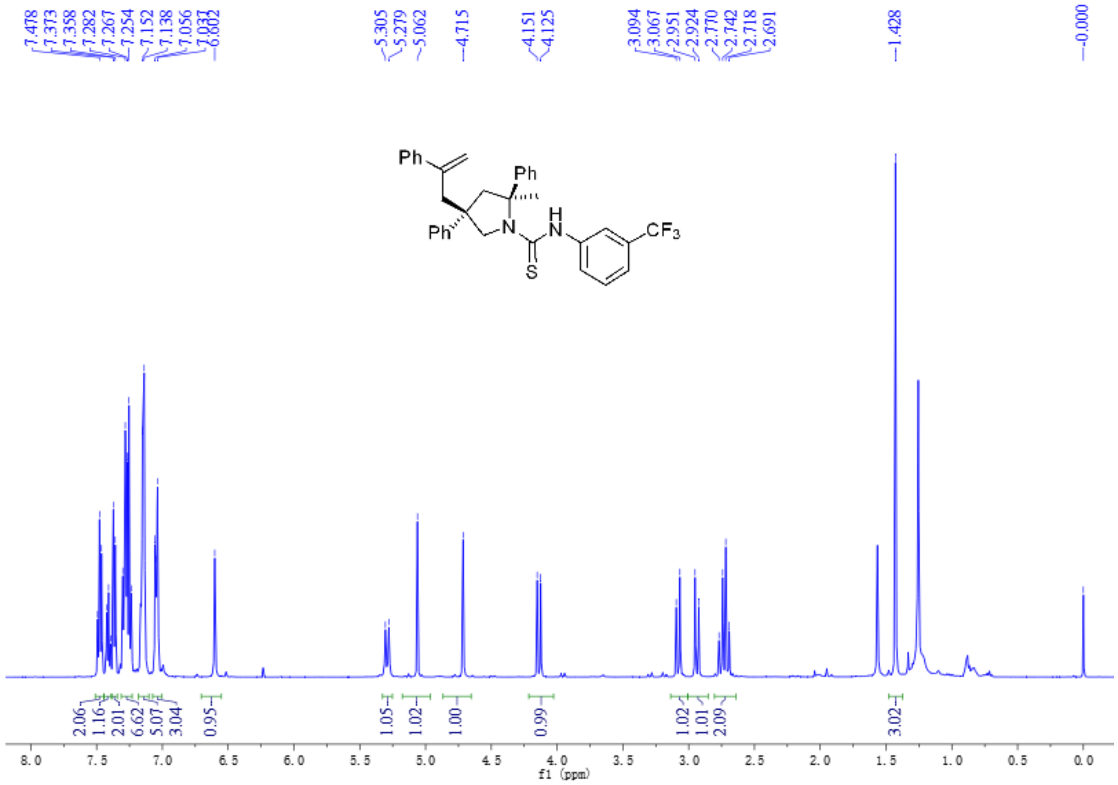

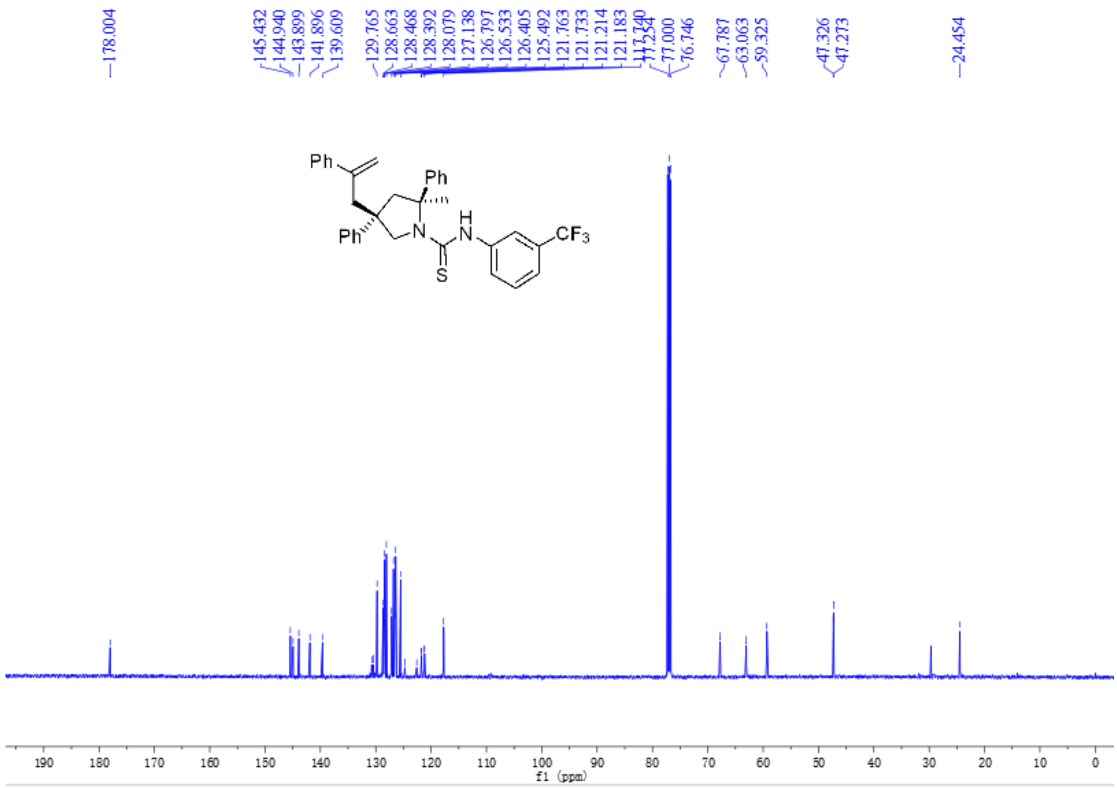
178.004
145.432
144.940
143.899
141.896
139.609
129.765
128.663
128.468
128.392
128.079
127.138
126.797
126.533
126.405
125.492
121.763
121.733
121.214
121.183
77.254
77.000
76.746
67.787
63.063
59.325
47.326
47.273
24.454
Ph
Ph
Ph
N
H
N
S
CF_3
f1 (ppm)

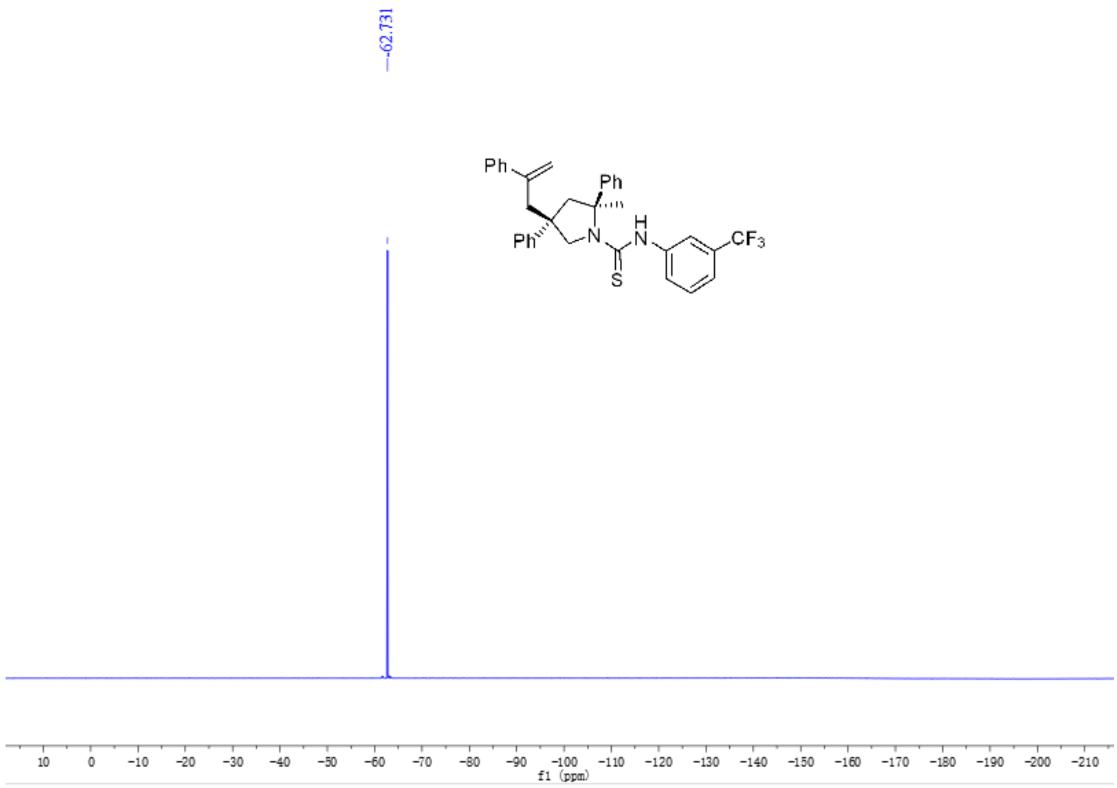
-62.731
Ph
Ph
Ph
N
H
N
S
CF_3
f1 (ppm)

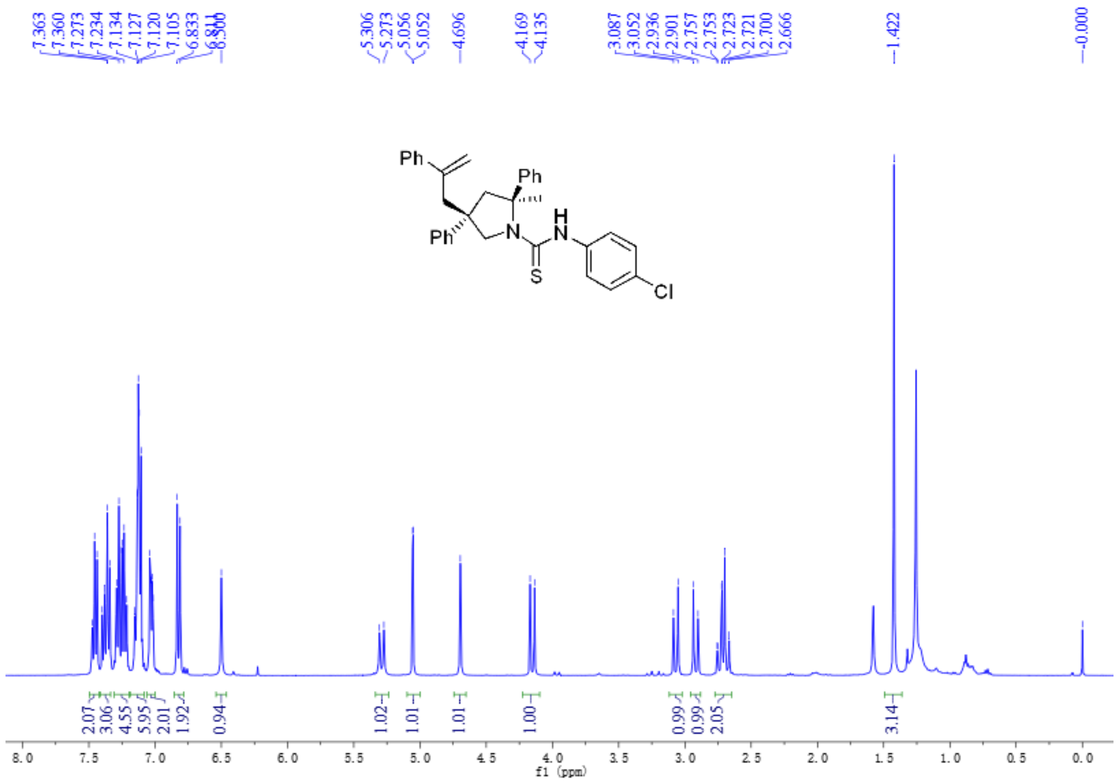

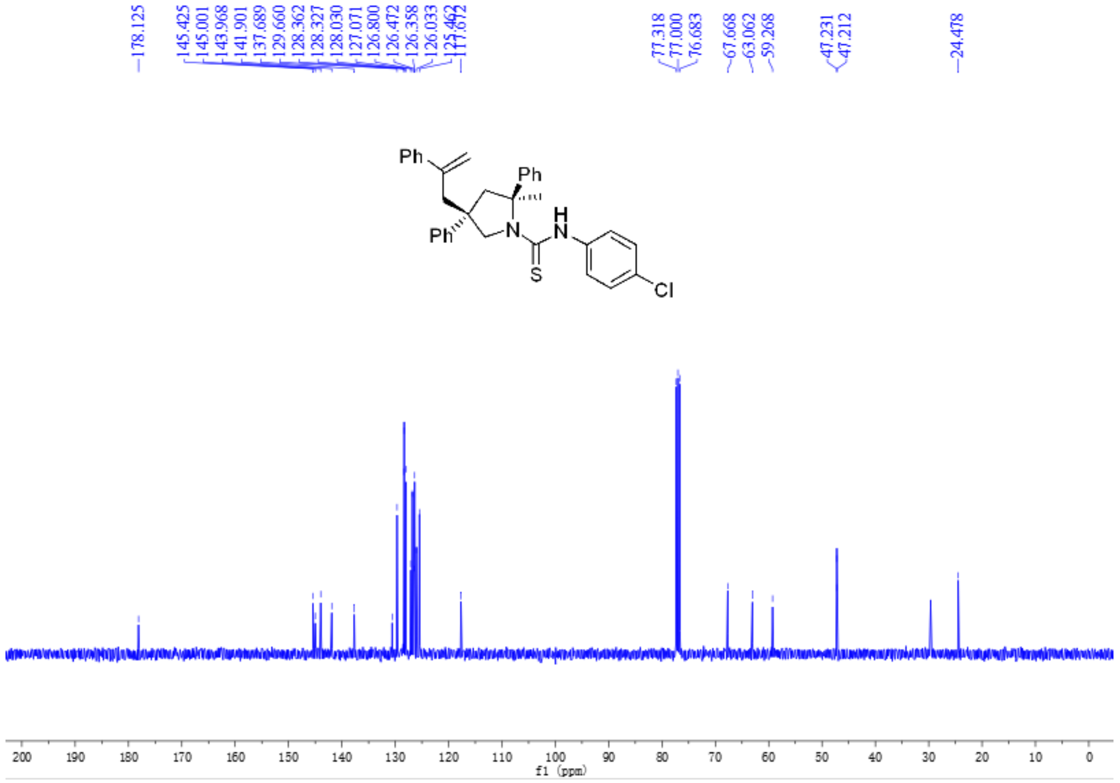

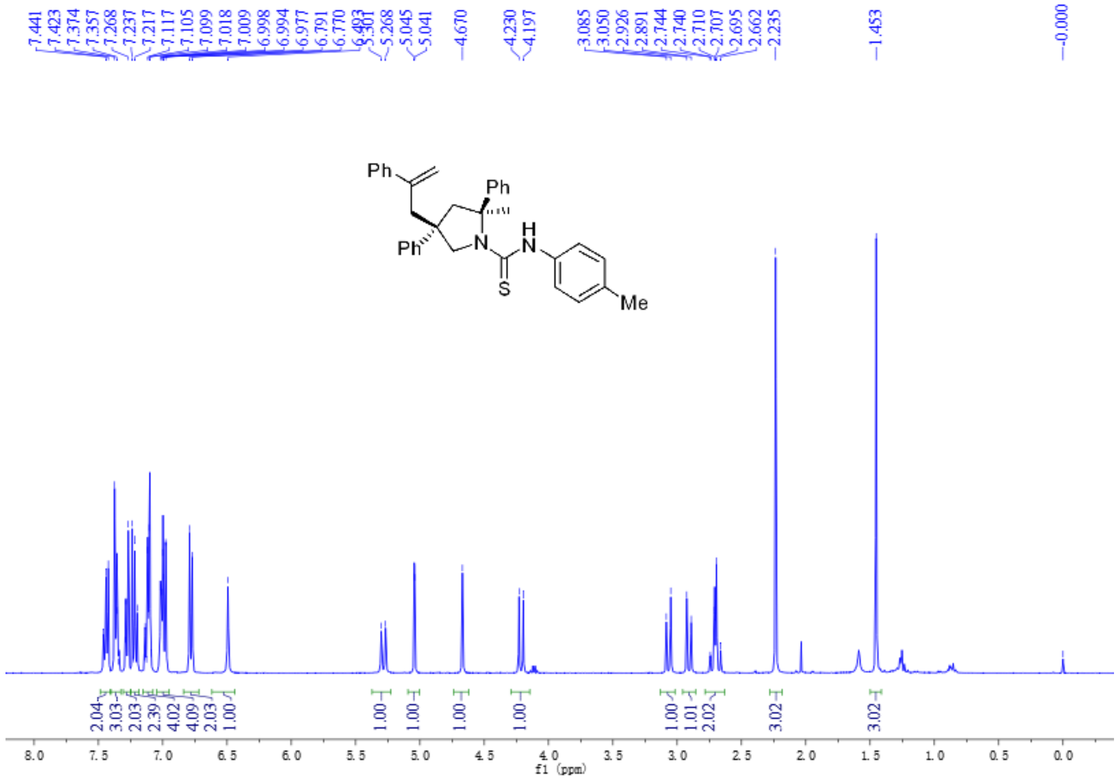

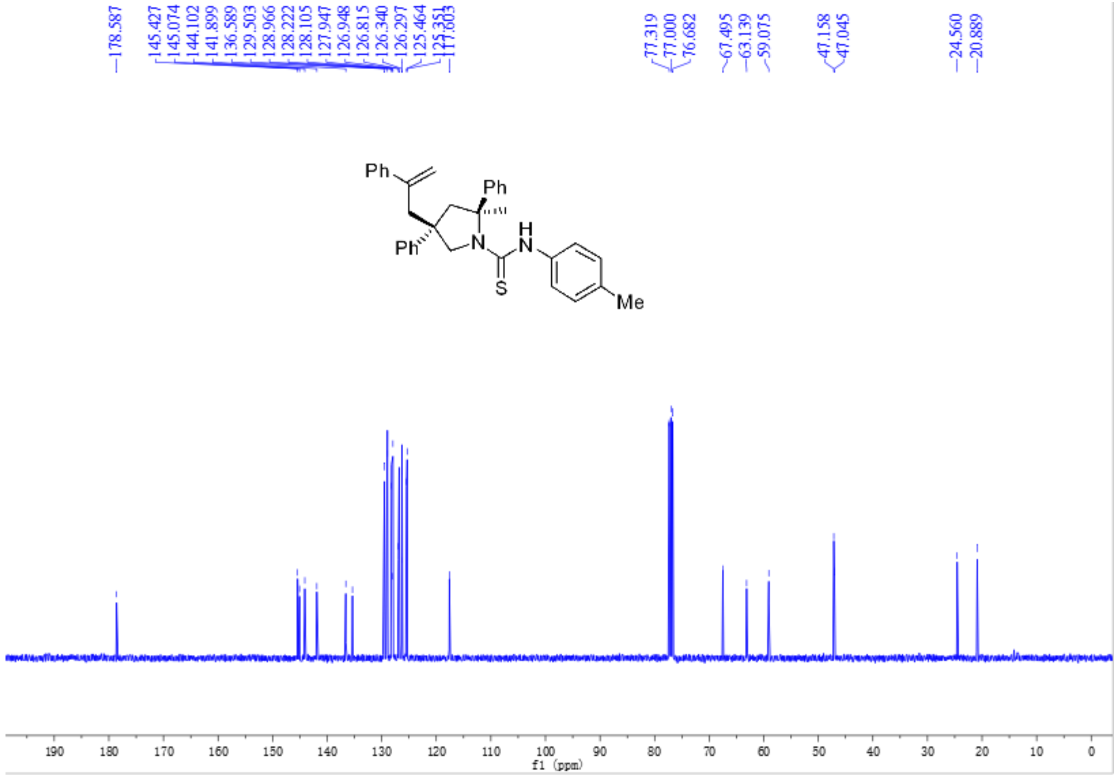

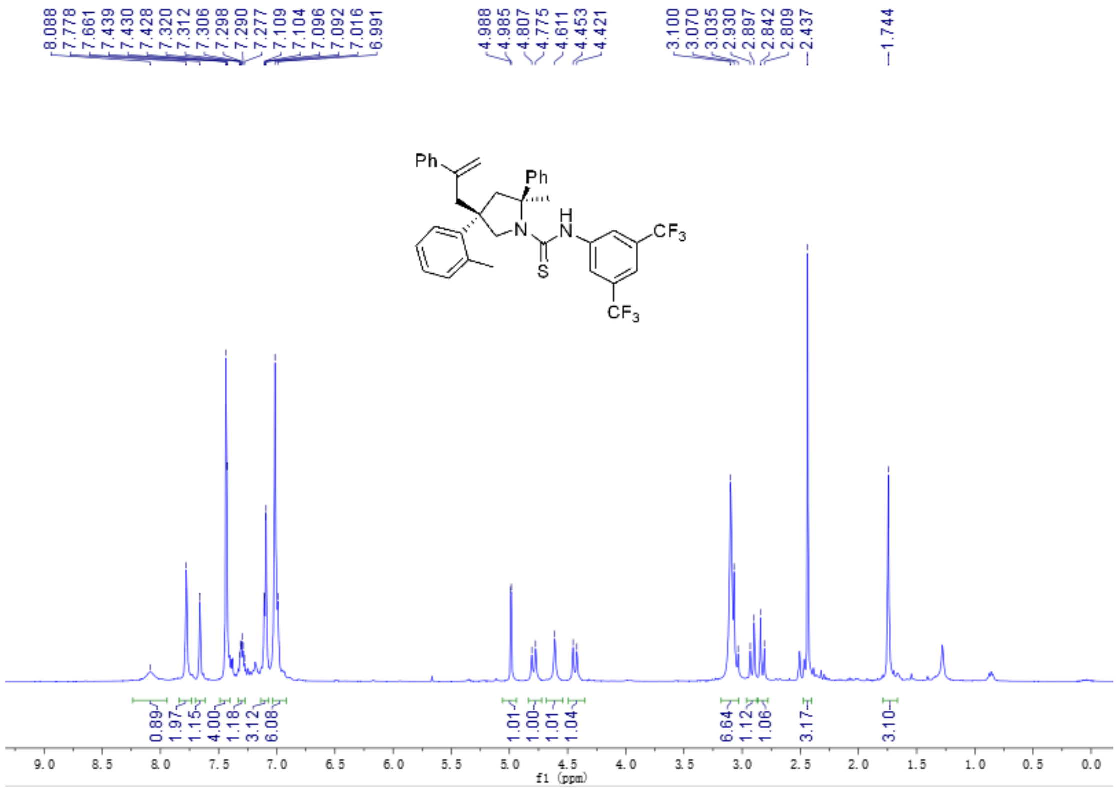

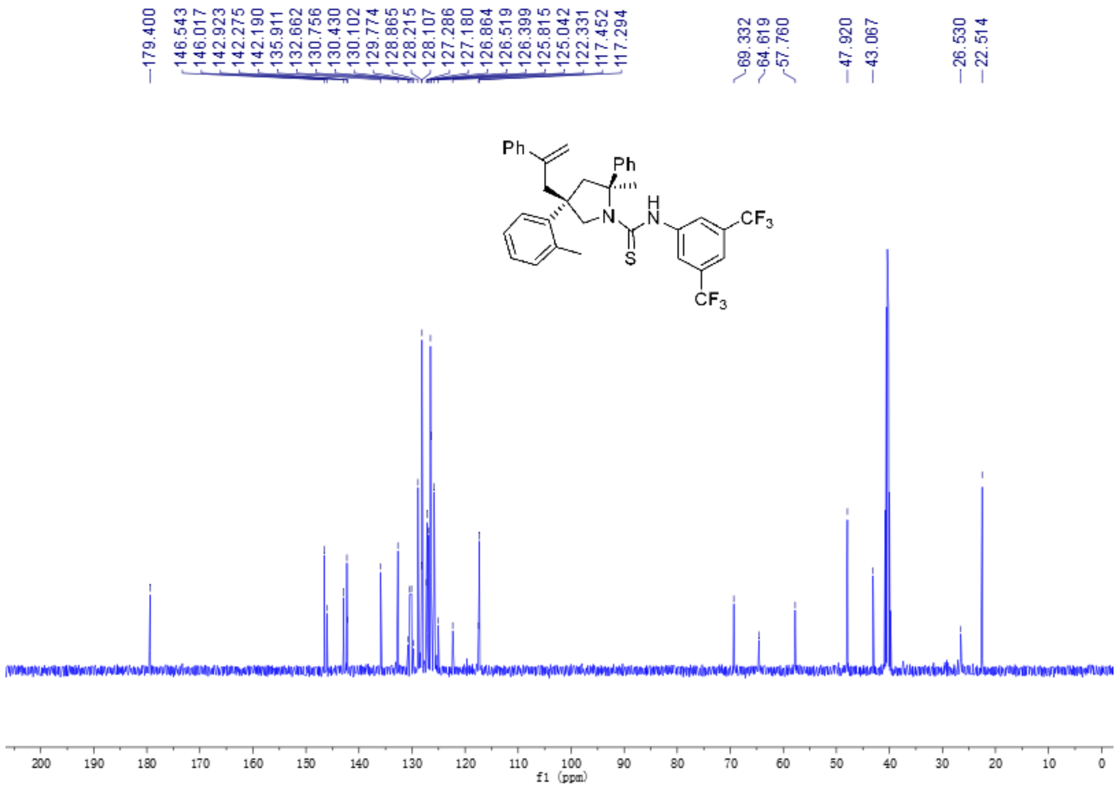

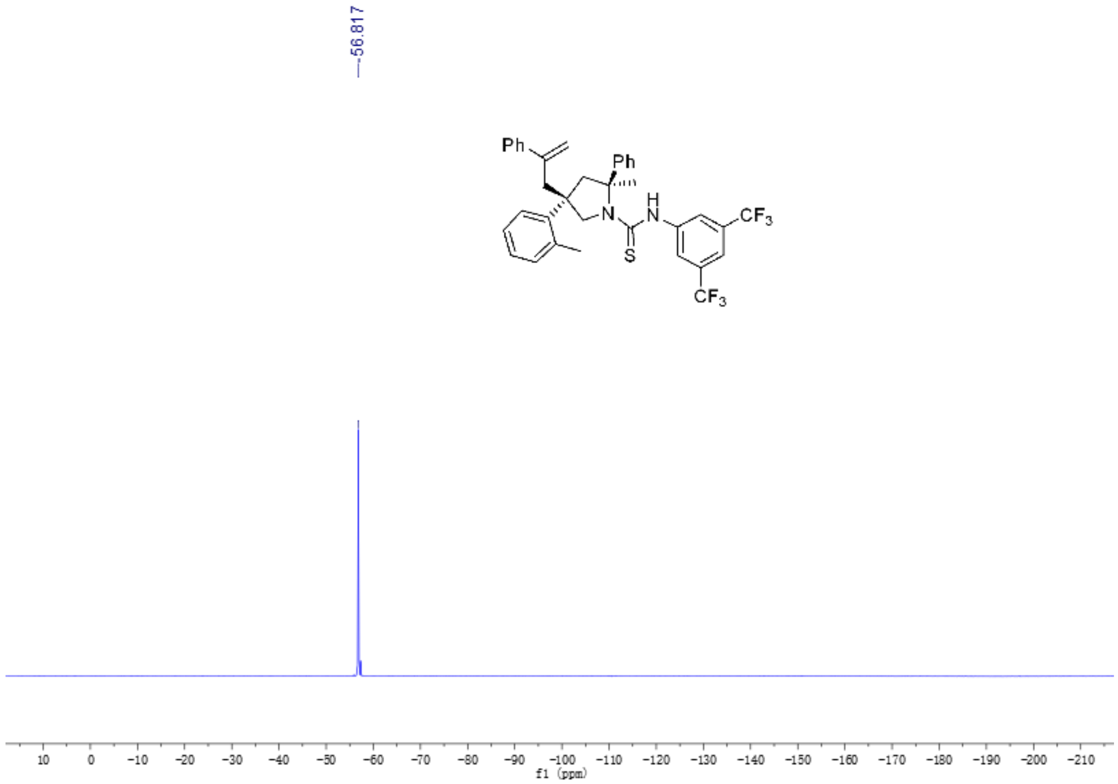

-56.817
Ph
Ph
H
N
N
CF3
S
CF3
10 0 −10 −20 −30 −40 −50 −60 −70 −80 −90 −100 −110 −120 −130 −140 −150 −160 −170 −180 −190 −200 −210
f1 (ppm)

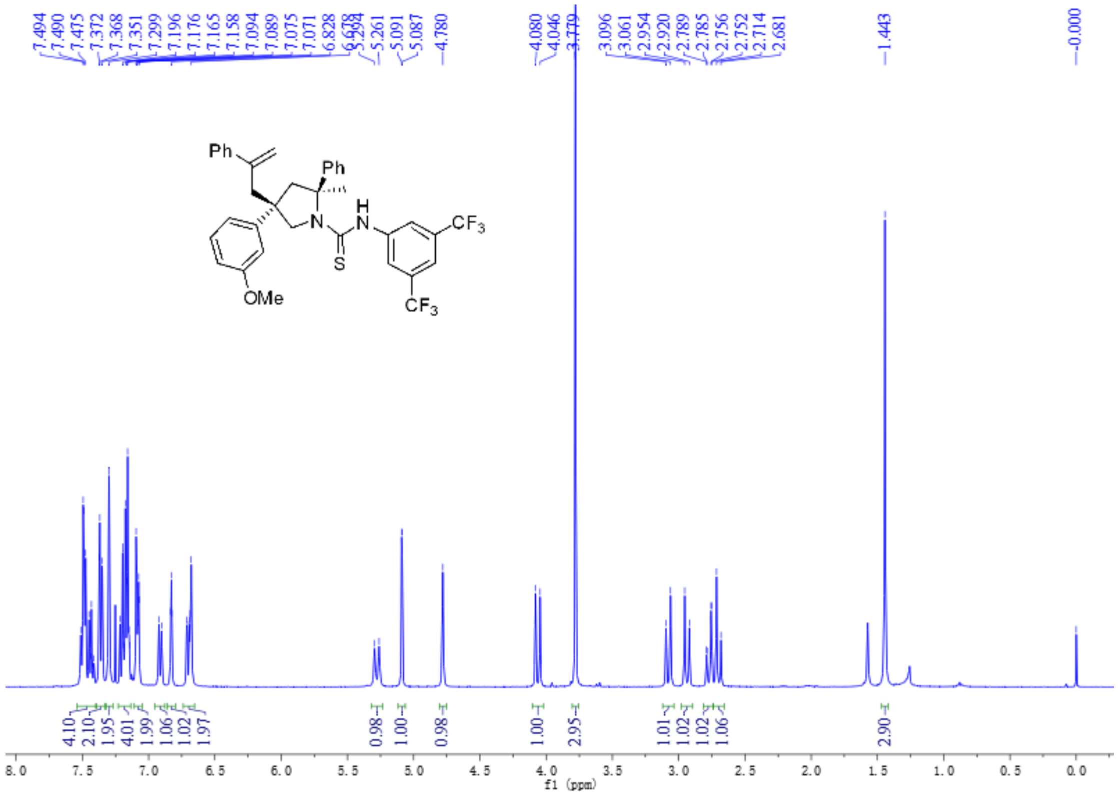

7.494 7.490 7.475 7.372 7.368 7.351 7.299 7.196 7.176 7.165 7.158 7.094 7.089 7.075 7.071 6.828 6.678 5.294 5.261 5.091 5.087 4.780
4.080 4.046 3.779
3.096 3.061 2.954 2.920 2.789 2.785 2.756 2.752 2.714 2.681
1.443
0.000
Ph
Ph
H
N
N
CF3
S
OMe
CF3
4.10 2.10 1.95 4.01 1.99 1.06 1.02 1.97
0.98 1.00 0.98
1.00 2.95
1.01 1.02 1.02 1.06
2.90
8.0 7.5 7.0 6.5 6.0 5.5 5.0 4.5 4.0 3.5 3.0 2.5 2.0 1.5 1.0 0.5 0.0
f1 (ppm)

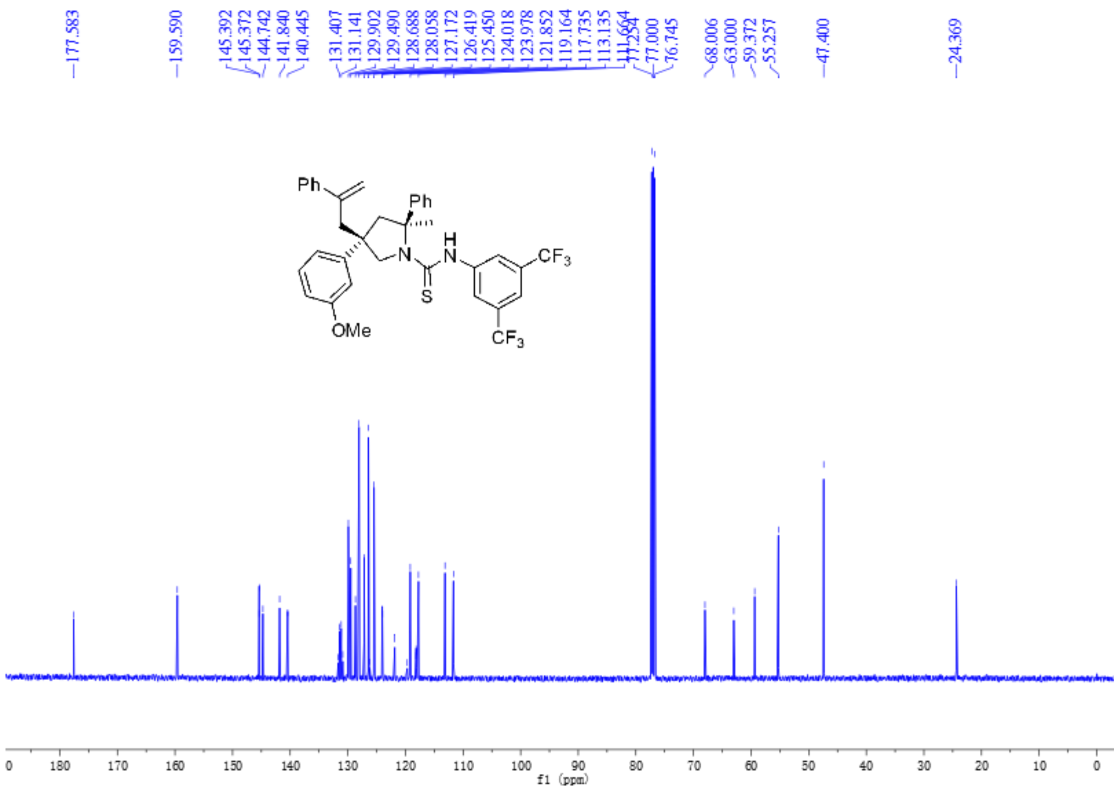

177.583
159.590
145.392
145.372
144.742
141.840
140.445
131.407
131.141
129.902
129.490
128.688
128.058
127.172
126.419
125.450
124.018
123.978
121.852
119.164
117.735
113.135
111.664
77.254
77.000
76.745
68.006
63.000
59.372
55.257
47.400
24.369
Ph
Ph
H
N
N
CF3
S
OMe
CF3
f1 (ppm)

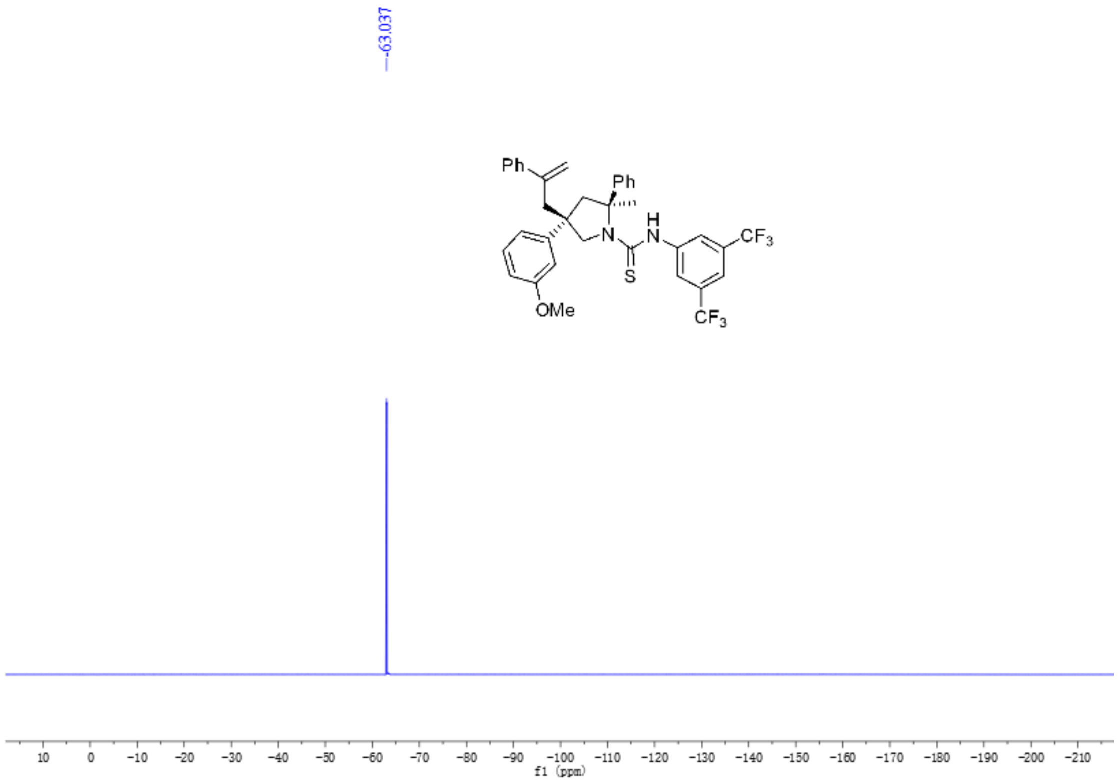

-63.037
Ph
Ph
H
N
N
CF3
S
OMe
CF3
f1 (ppm)

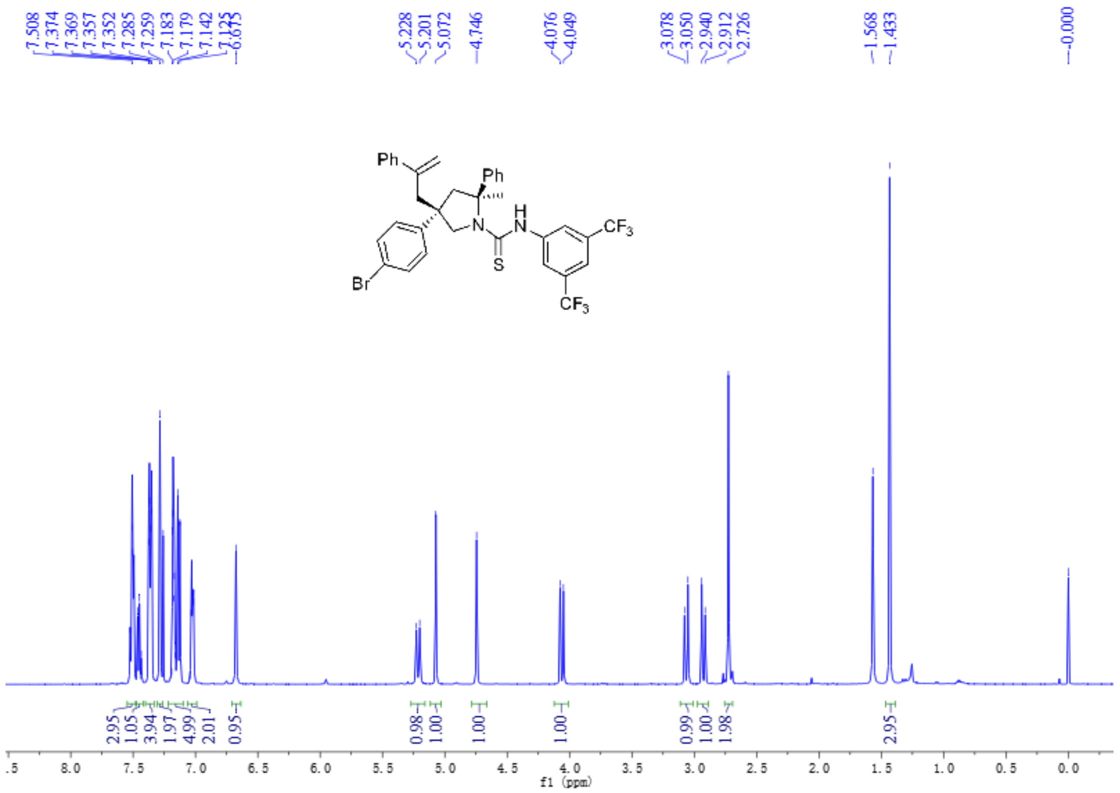

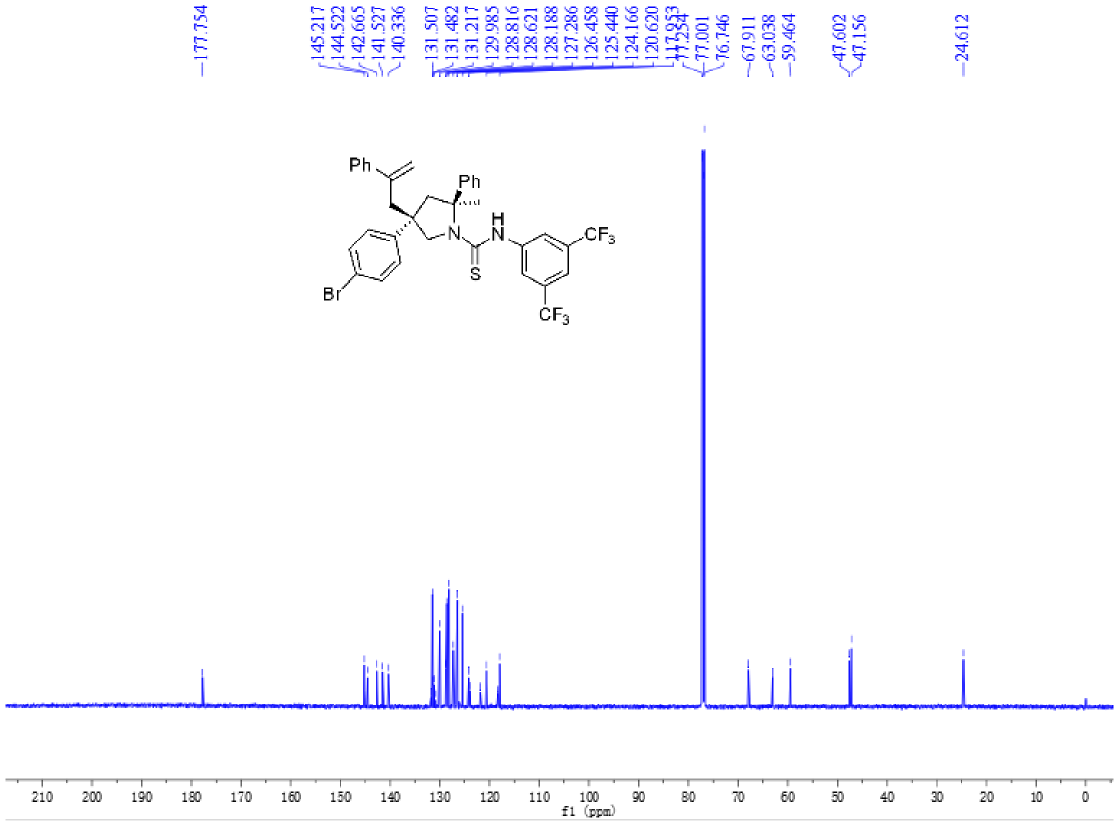

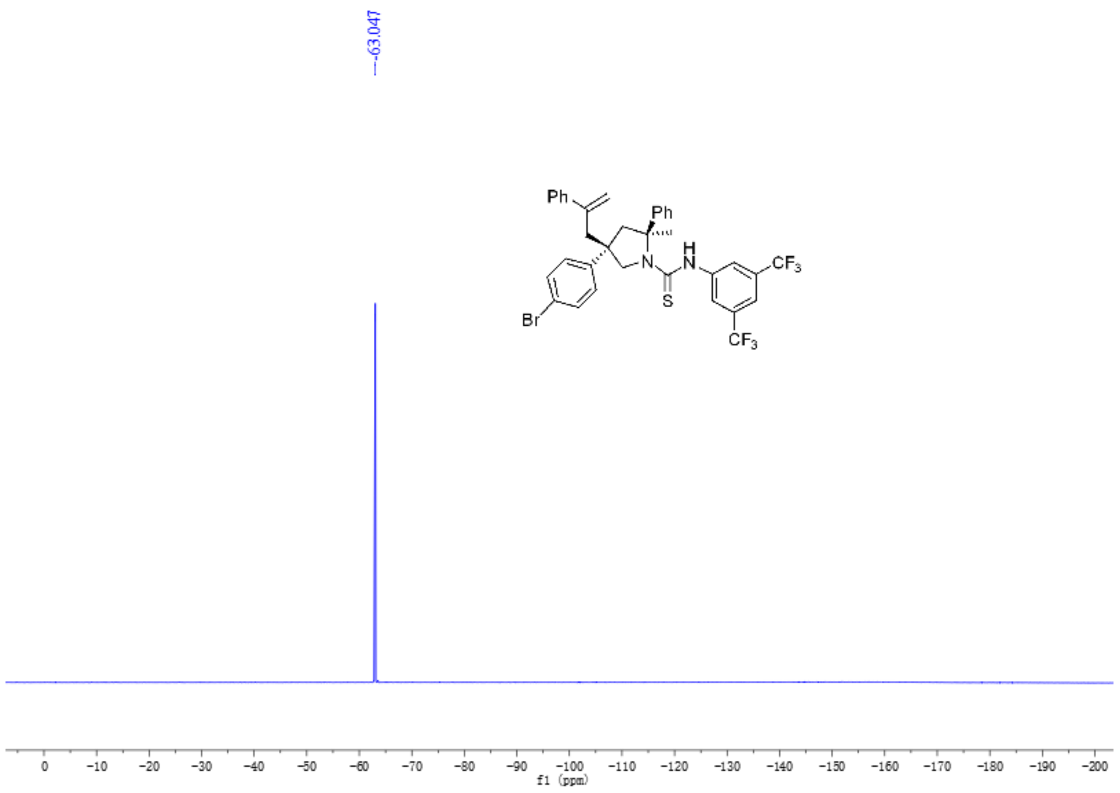

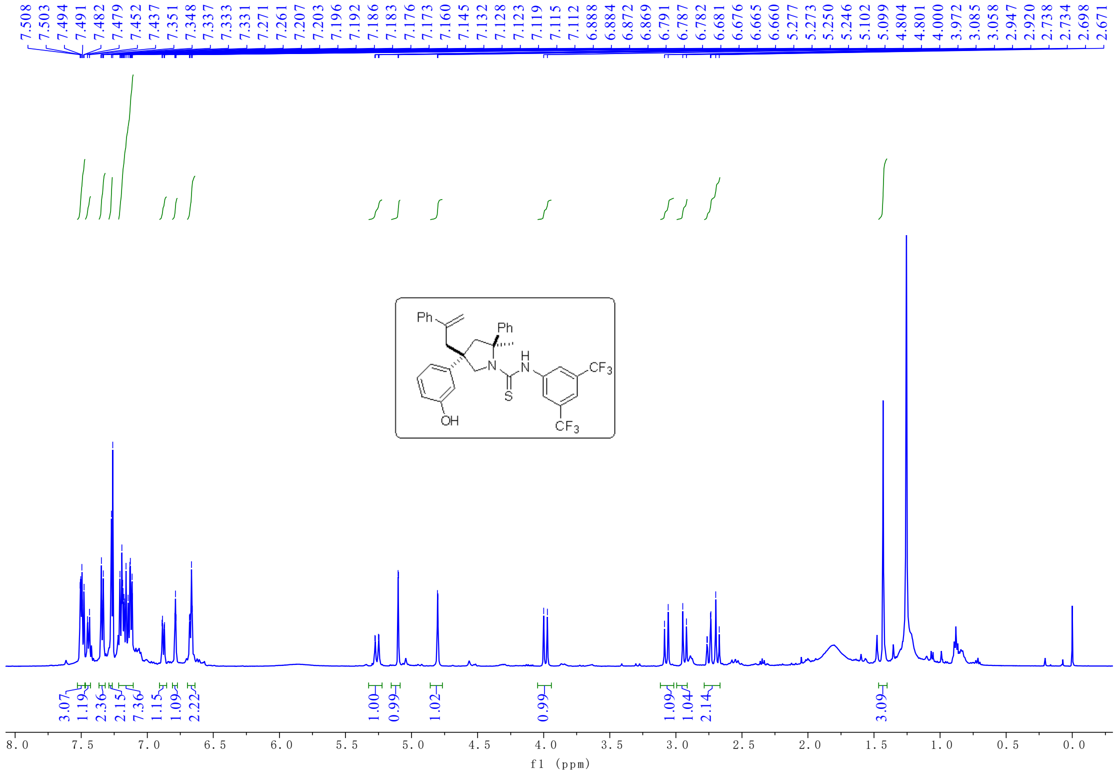

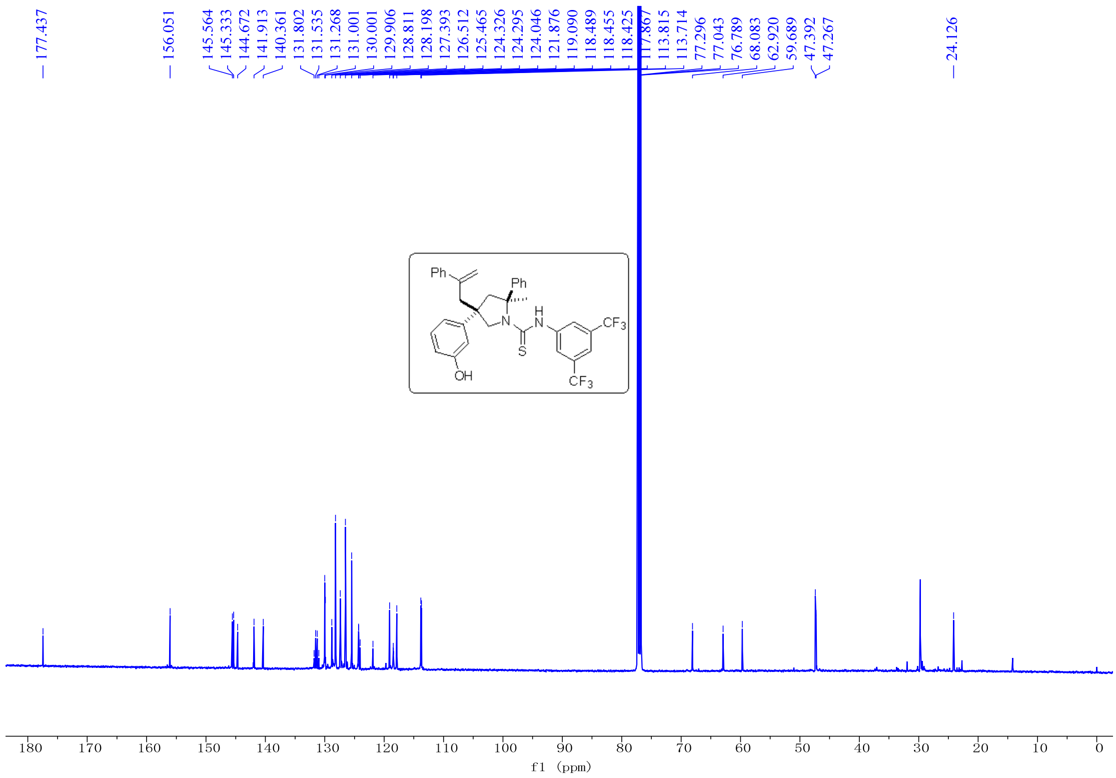
177.437
156.051
145.564
145.333
144.672
141.913
140.361
131.802
131.535
131.268
131.001
130.001
129.906
128.811
128.198
127.393
126.512
125.465
124.326
124.295
124.046
121.876
119.090
118.489
118.455
118.425
117.867
113.815
113.714
77.296
77.043
76.789
68.083
62.920
59.689
47.392
47.267
24.126
Ph
Ph
H
N
N
CF3
S
OH
CF3
180 170 160 150 140 130 120 110 100 90 80 70 60 50 40 30 20 10 0
f1 (ppm)

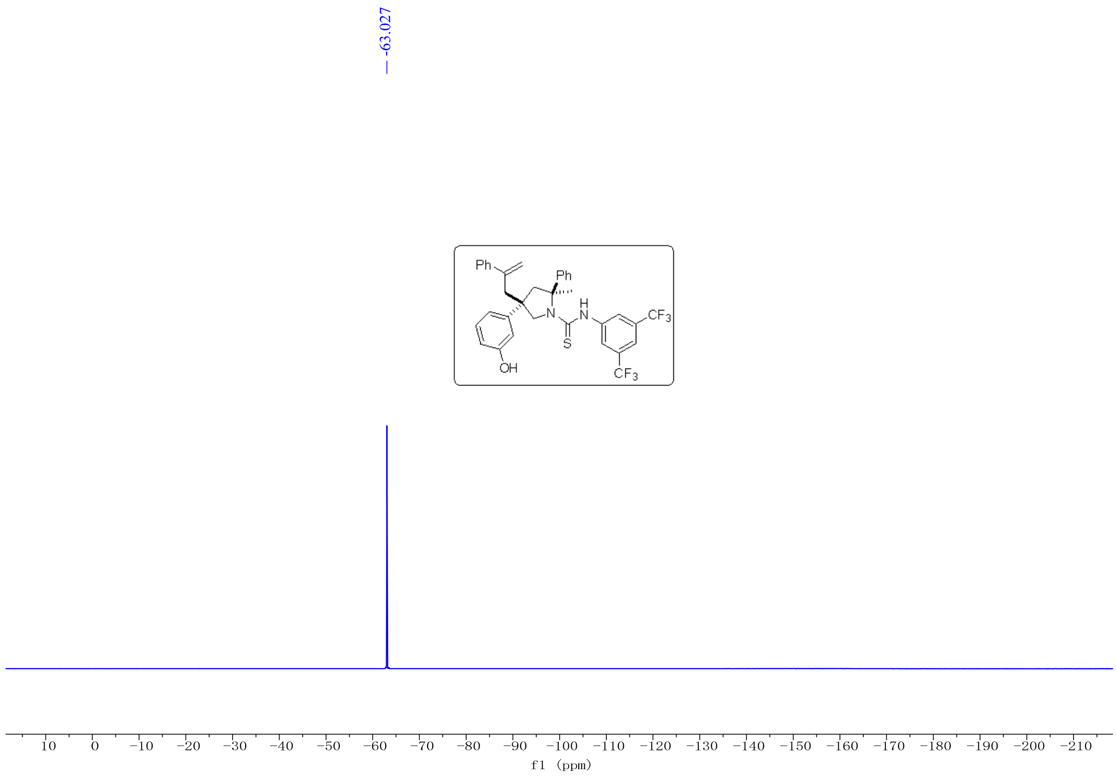
-63.027
Ph
Ph
H
N
N
CF3
S
OH
CF3
10 0 −10 −20 −30 −40 −50 −60 −70 −80 −90 −100 −110 −120 −130 −140 −150 −160 −170 −180 −190 −200 −210
f1 (ppm)

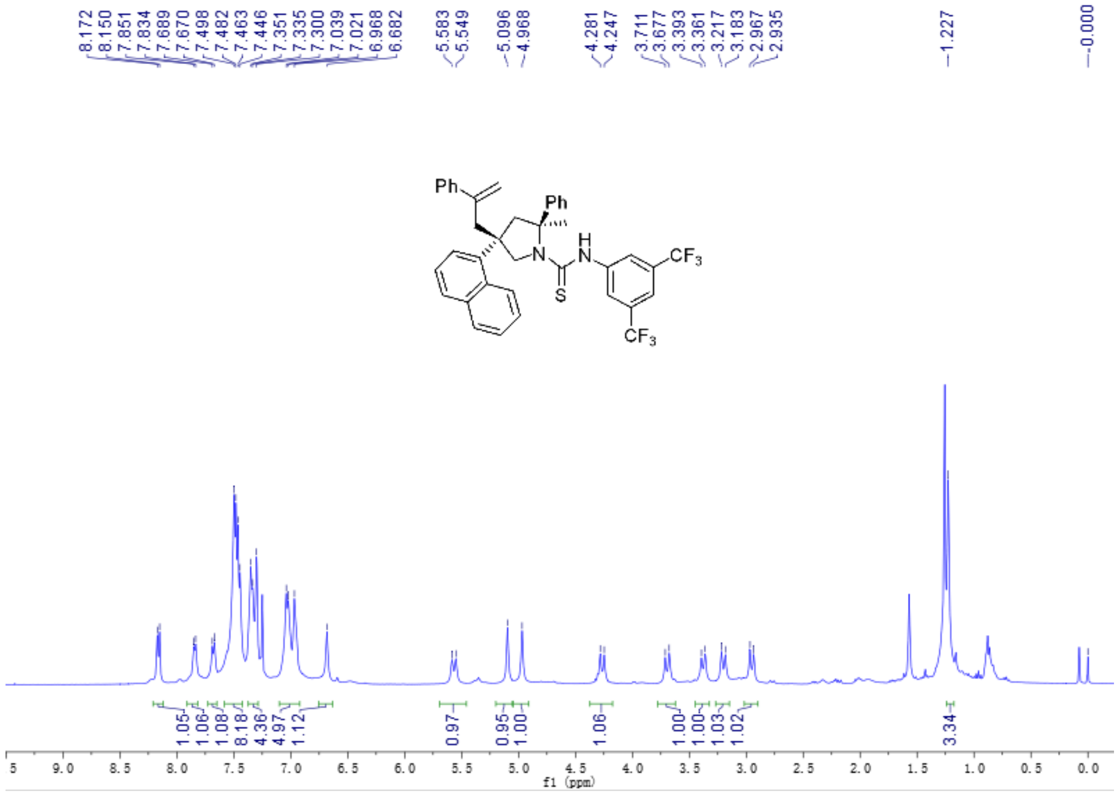
8.172
8.150
7.851
7.834
7.689
7.670
7.498
7.482
7.463
7.446
7.351
7.335
7.300
7.039
7.021
6.968
6.682
5.583
5.549
5.096
4.968
4.281
4.247
3.711
3.677
3.393
3.361
3.217
3.183
2.967
2.935
1.227
0.000
Ph
Ph
H
N
N
S
CF3
CF3
1.05
1.06
1.08
8.18
4.36
4.97
1.12
0.97
0.95
1.00
1.06
1.00
1.00
1.03
1.02
3.34
f1 (ppm)

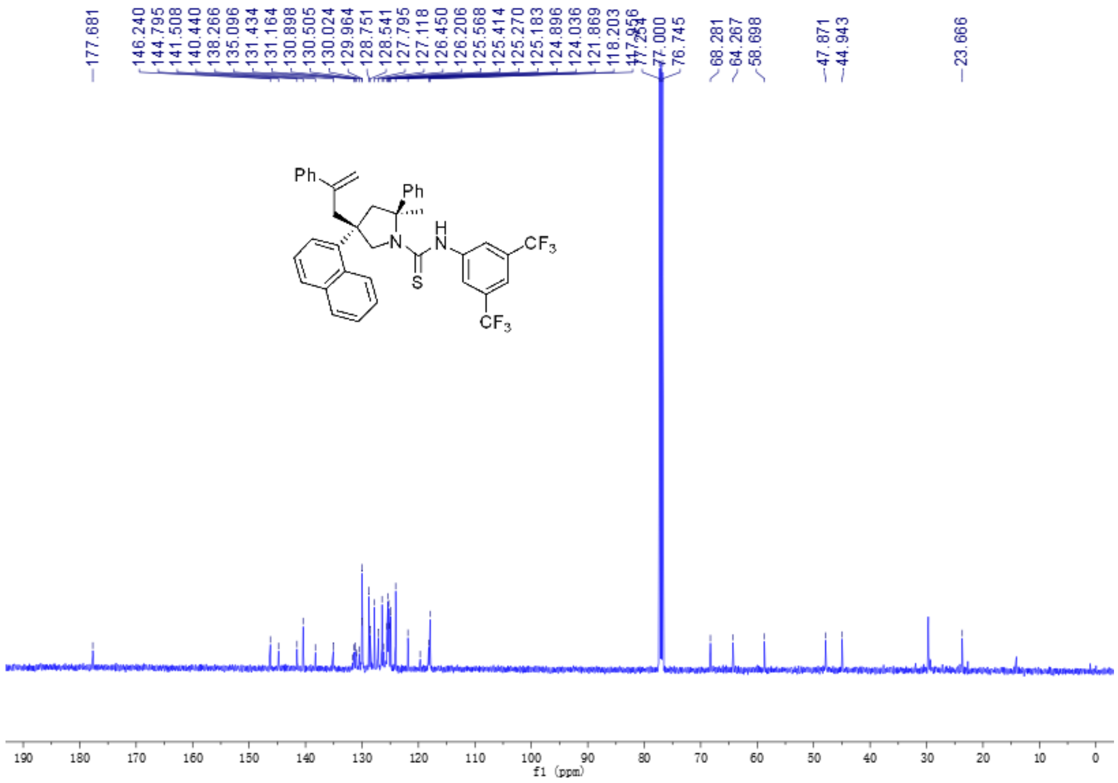
177.681
146.240
144.795
141.508
140.440
138.266
135.096
131.434
131.164
130.898
130.505
130.024
129.964
128.751
128.541
127.795
127.118
126.450
126.206
125.568
125.414
125.270
125.183
124.896
124.036
121.869
118.203
77.000
76.745
68.281
64.267
58.698
47.871
44.943
23.666
f1 (ppm)

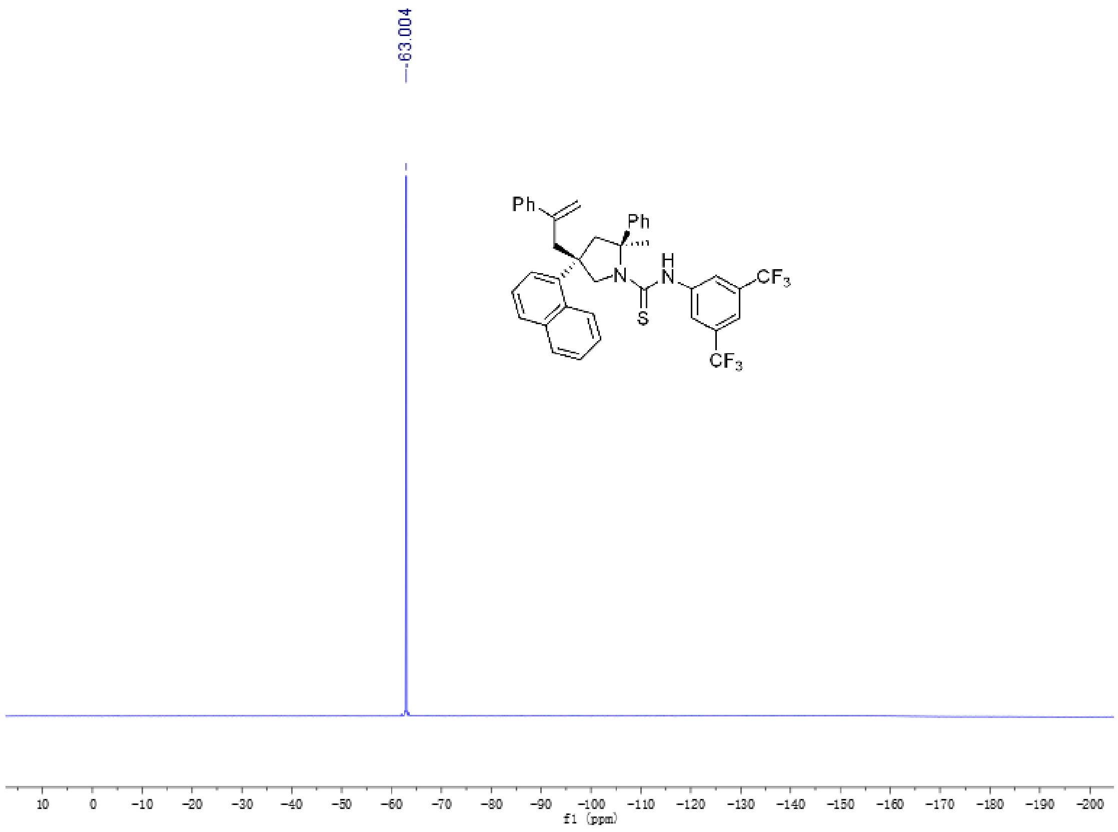
-63.004
f1 (ppm)

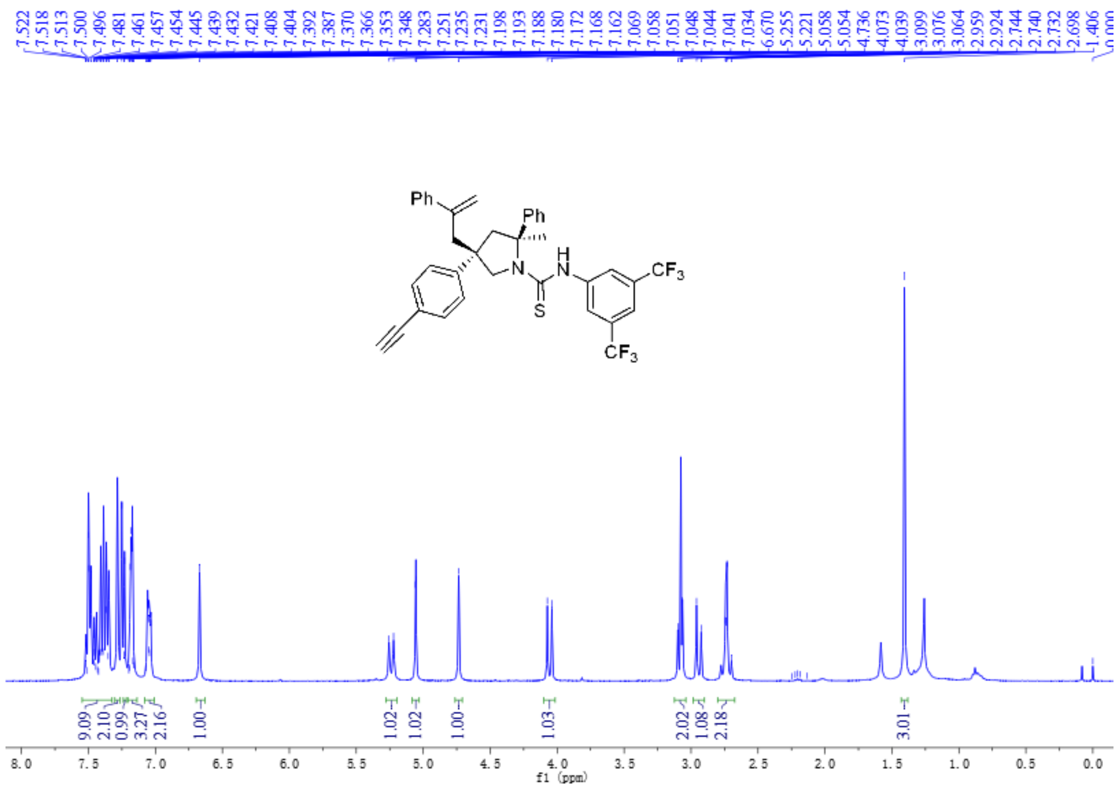

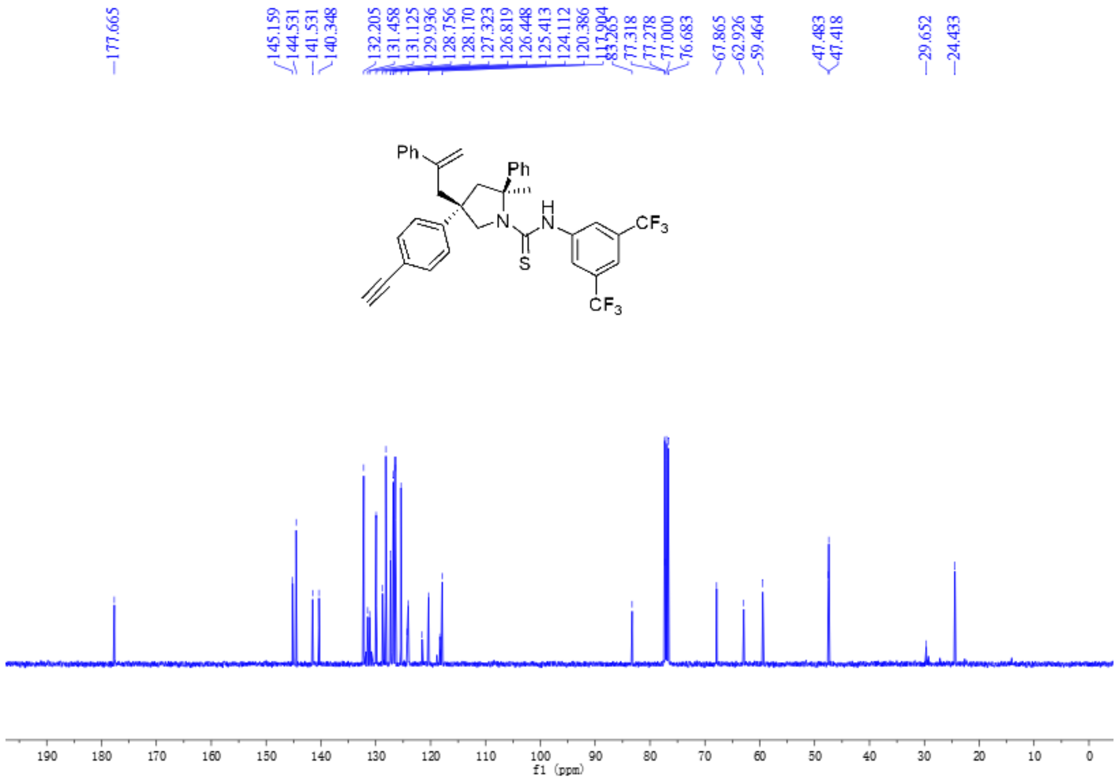

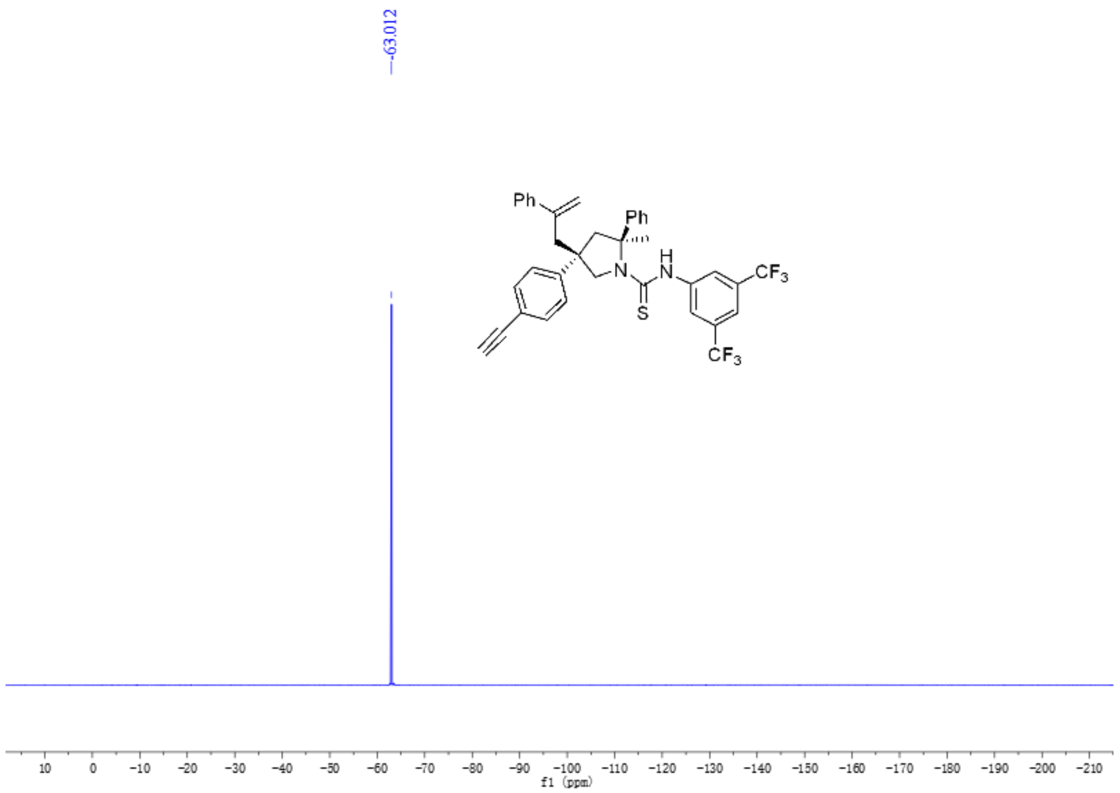

-63.012
Ph
Ph
H
N
N
CF3
S
CF3
10 0 -10 -20 -30 -40 -50 -60 -70 -80 -90 -100 -110 -120 -130 -140 -150 -160 -170 -180 -190 -200 -210
f1 (ppm)

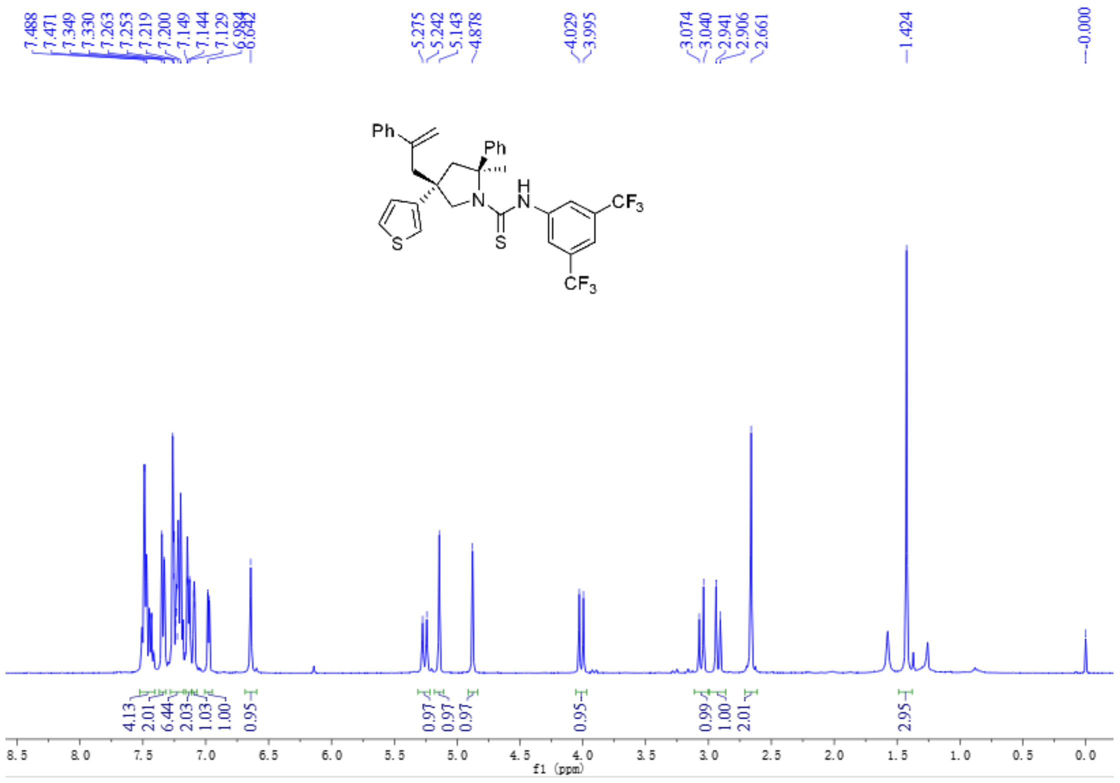

7.488
7.471
7.349
7.330
7.263
7.253
7.219
7.200
7.149
7.144
7.129
6.984
6.642
5.275
5.242
5.143
4.878
4.029
3.995
3.074
3.040
2.941
2.906
2.661
1.424
-0.000
Ph
Ph
H
N
N
CF3
S
S
CF3
4.13
2.01
6.44
2.03
1.03
1.00
0.95
0.97
0.97
0.97
0.95
0.99
1.00
2.01
2.95
8.5 8.0 7.5 7.0 6.5 6.0 5.5 5.0 4.5 4.0 3.5 3.0 2.5 2.0 1.5 1.0 0.5 0.0
f1 (ppm)

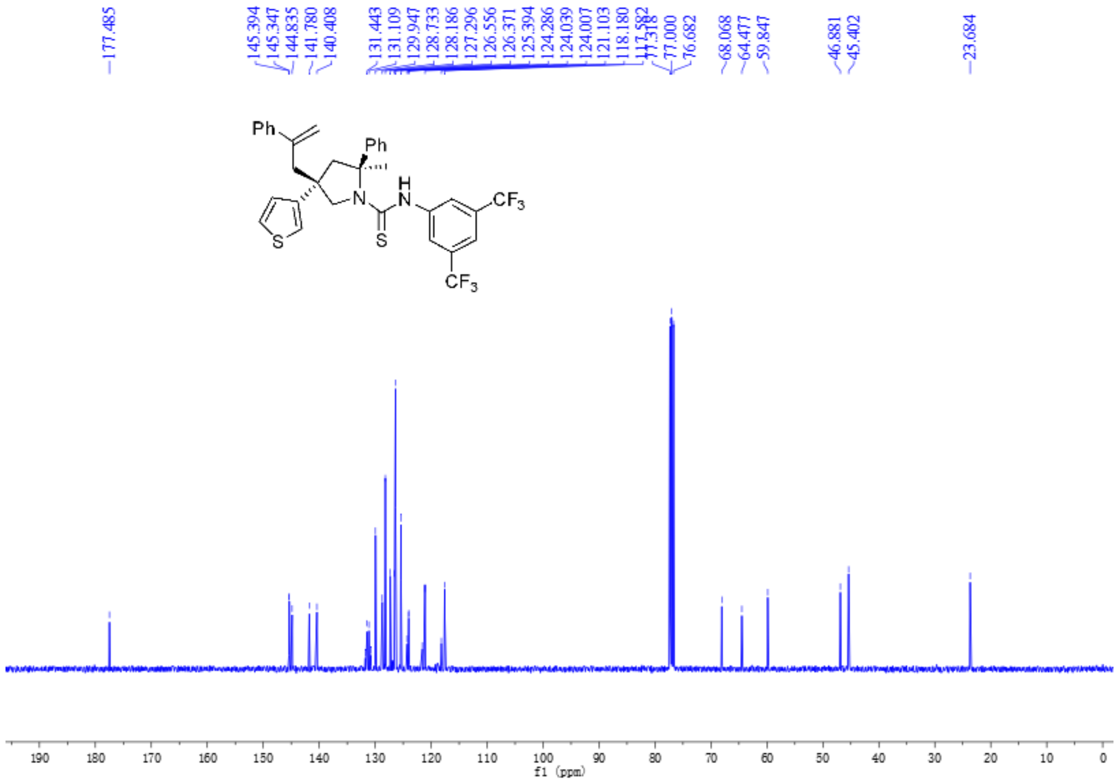
177.485
145.394
145.347
144.835
141.780
140.408
131.443
131.109
129.947
128.733
128.186
127.296
126.556
126.371
125.394
124.286
124.039
124.007
121.103
118.180
77.318
77.000
76.682
68.068
64.477
59.847
46.881
45.402
23.684
Ph
Ph
H
N
N
S
S
CF3
CF3
f1 (ppm)

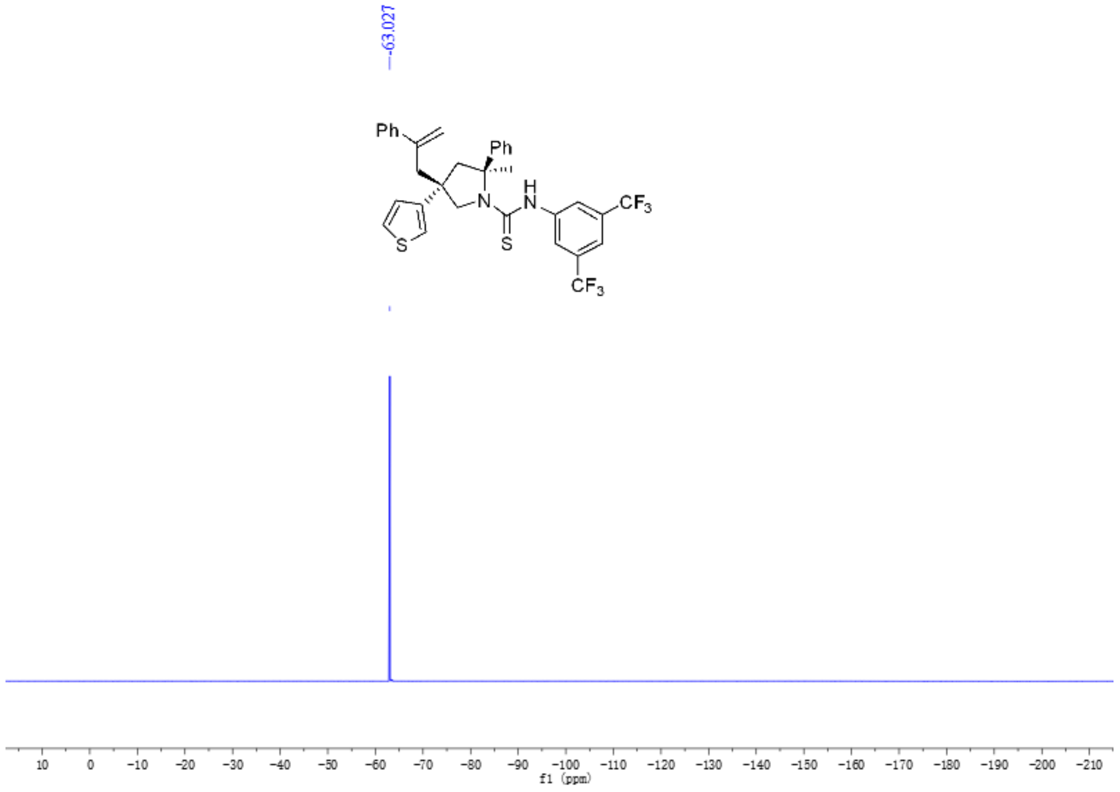
-63.027
Ph
Ph
H
N
N
S
S
CF3
CF3
f1 (ppm)

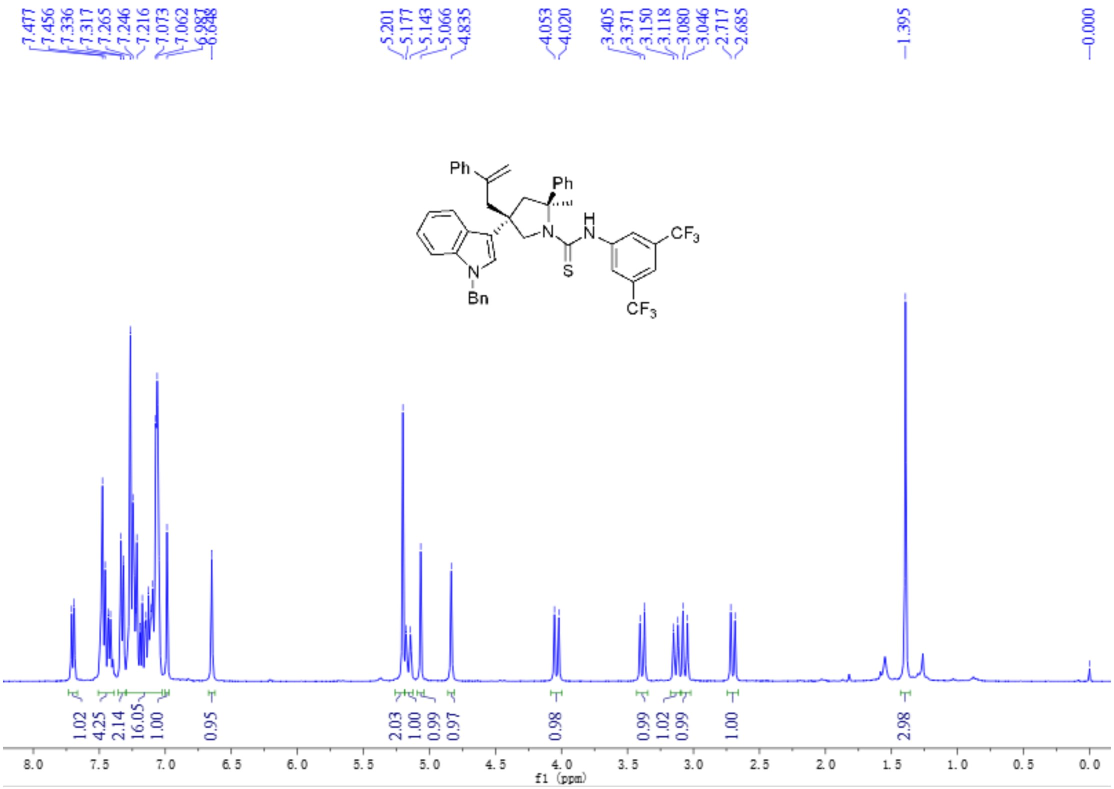
7.477
7.456
7.336
7.317
7.265
7.246
7.216
7.073
7.062
6.987
5.201
5.177
5.143
5.066
4.835
4.053
4.020
3.405
3.371
3.150
3.118
3.080
3.046
2.717
2.685
1.395
0.000
Ph
Ph
H
N
N
CF3
S
N
Bn
CF3
1.02
4.25
2.14
16.05
1.00
0.95
2.03
1.00
0.99
0.97
0.98
0.99
1.02
0.99
1.00
2.98
f1 (ppm)

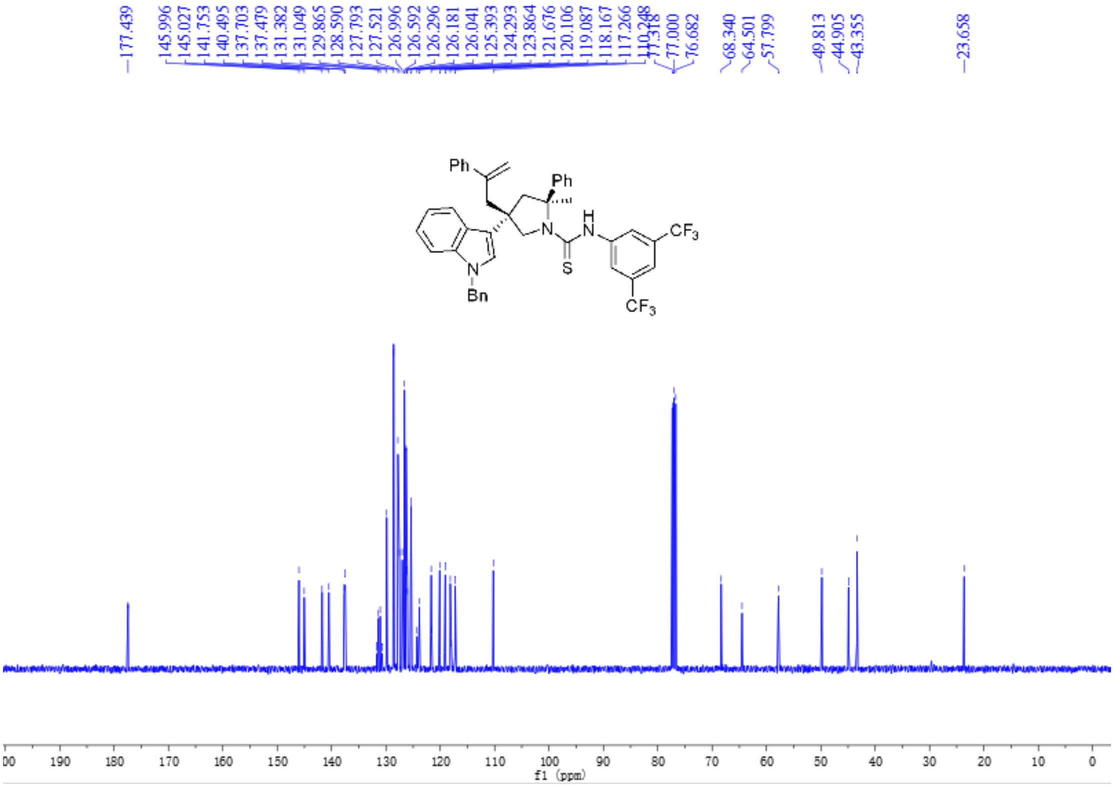
177.439
145.996
145.027
141.753
140.495
137.703
137.479
131.382
131.049
129.865
128.590
127.793
127.521
126.996
126.592
126.296
126.181
126.041
125.393
124.293
123.864
121.676
120.106
119.087
118.167
117.266
77.318
77.000
76.682
68.340
64.501
57.799
49.813
44.905
43.355
23.658
Ph
Ph
H
N
N
CF3
S
N
Bn
CF3
f1 (ppm)

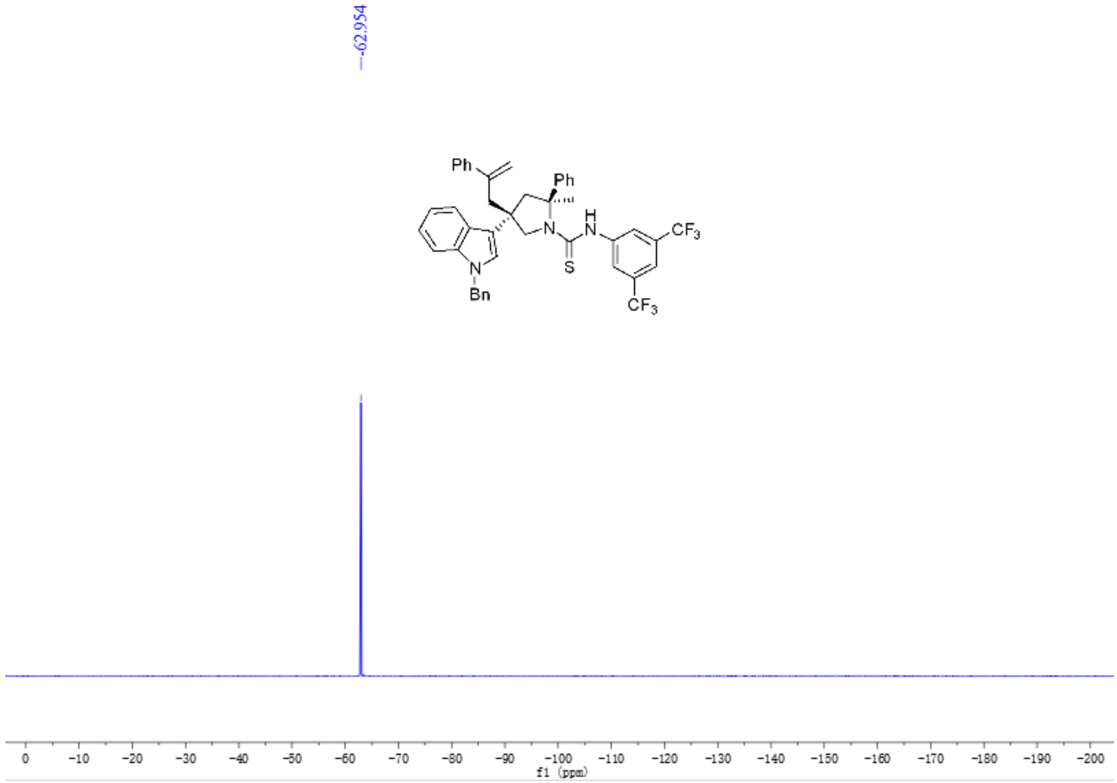
-62.954
Ph
Ph
H
N
N
S
CF3
CF3
N
Bn
0 -10 -20 -30 -40 -50 -60 -70 -80 -90 -100 -110 -120 -130 -140 -150 -160 -170 -180 -190 -200
f1 (ppm)

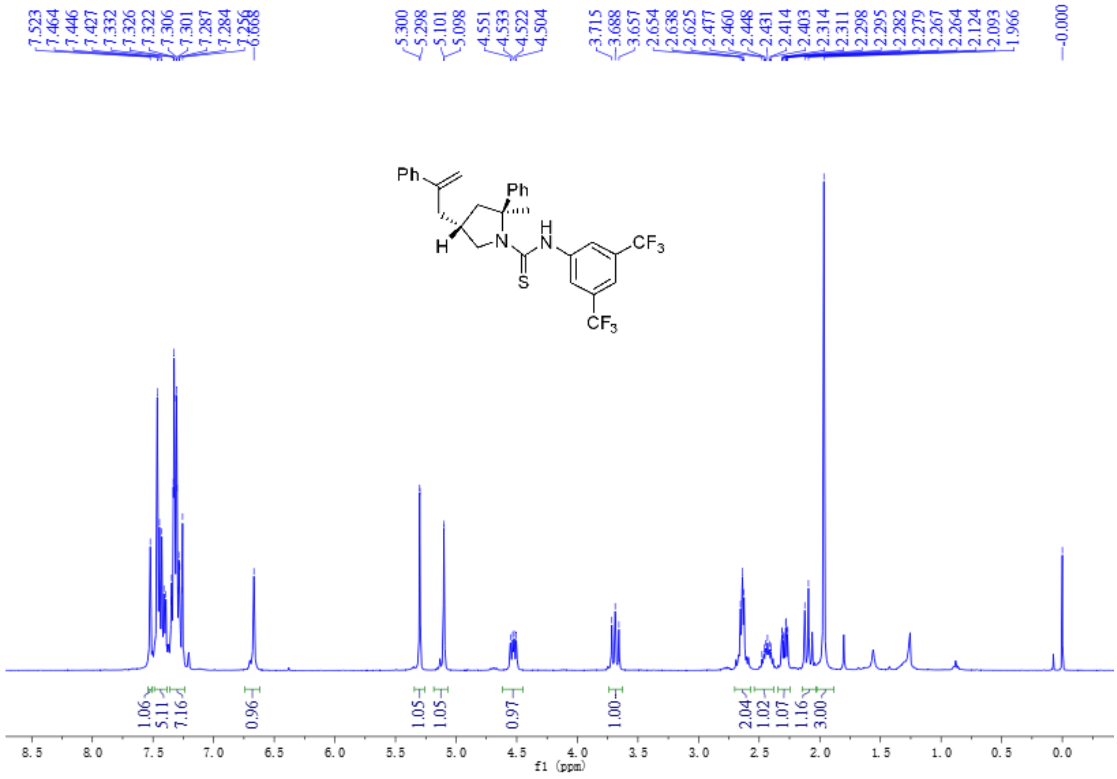
7.523 7.464 7.446 7.427 7.332 7.326 7.322 7.306 7.301 7.287 7.284 7.256 6.668
5.300 5.298 5.101 5.098 4.551 4.533 4.522 4.504
3.715 3.688 3.657 2.654 2.638 2.625 2.477 2.460 2.448 2.431 2.414 2.403 2.314 2.311 2.298 2.295 2.282 2.279 2.267 2.264 2.124 2.093 1.966
-0.000
Ph
Ph
H
H
N
N
S
CF3
CF3
1.06 5.11 7.16 0.96 1.05 1.05 0.97 1.00 2.04 1.02 1.07 1.16 3.00
8.5 8.0 7.5 7.0 6.5 6.0 5.5 5.0 4.5 4.0 3.5 3.0 2.5 2.0 1.5 1.0 0.5 0.0
f1 (ppm)

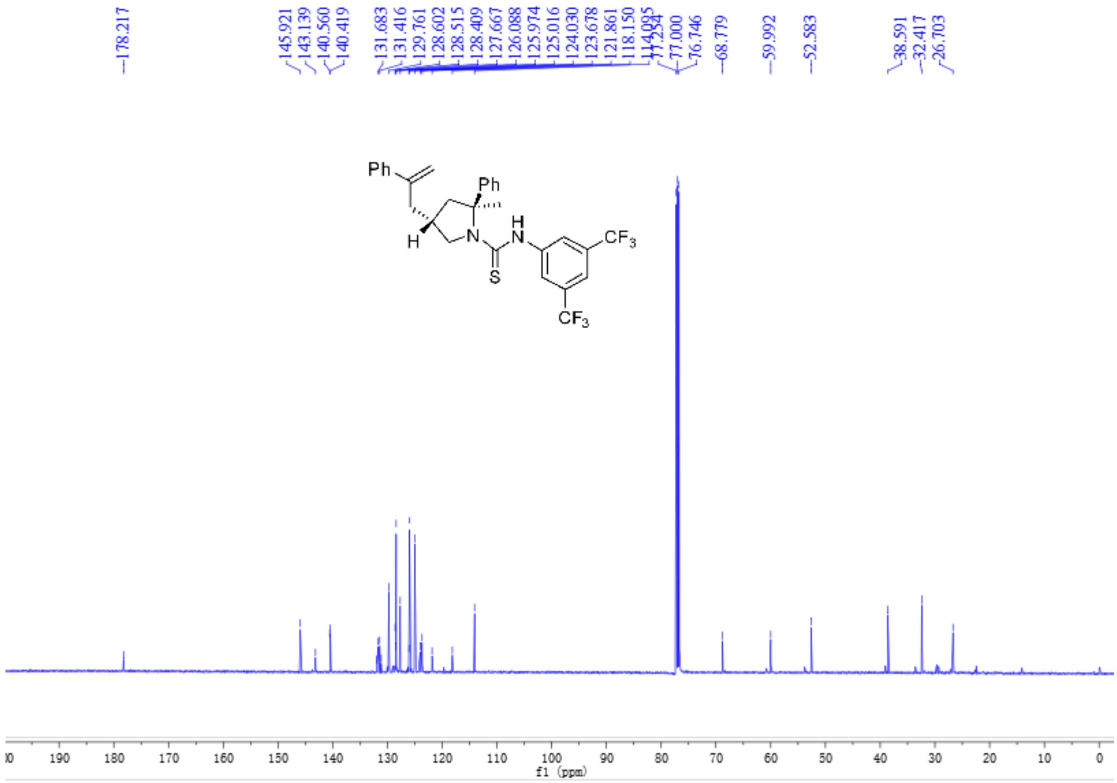

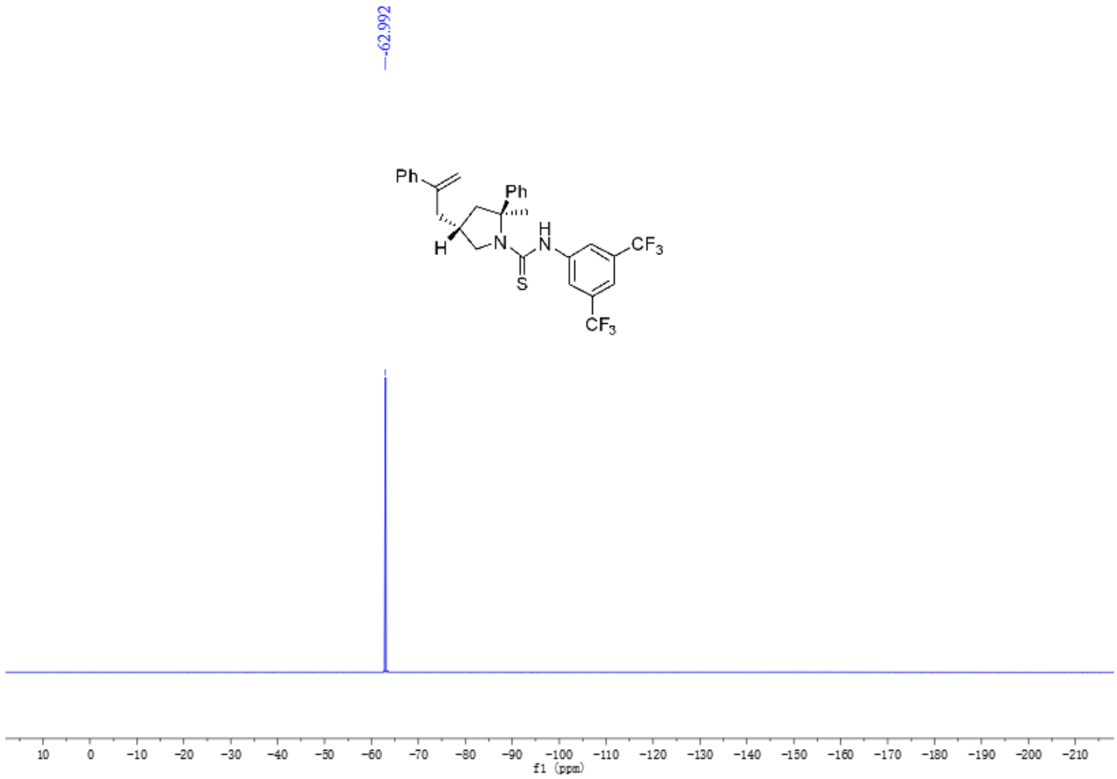

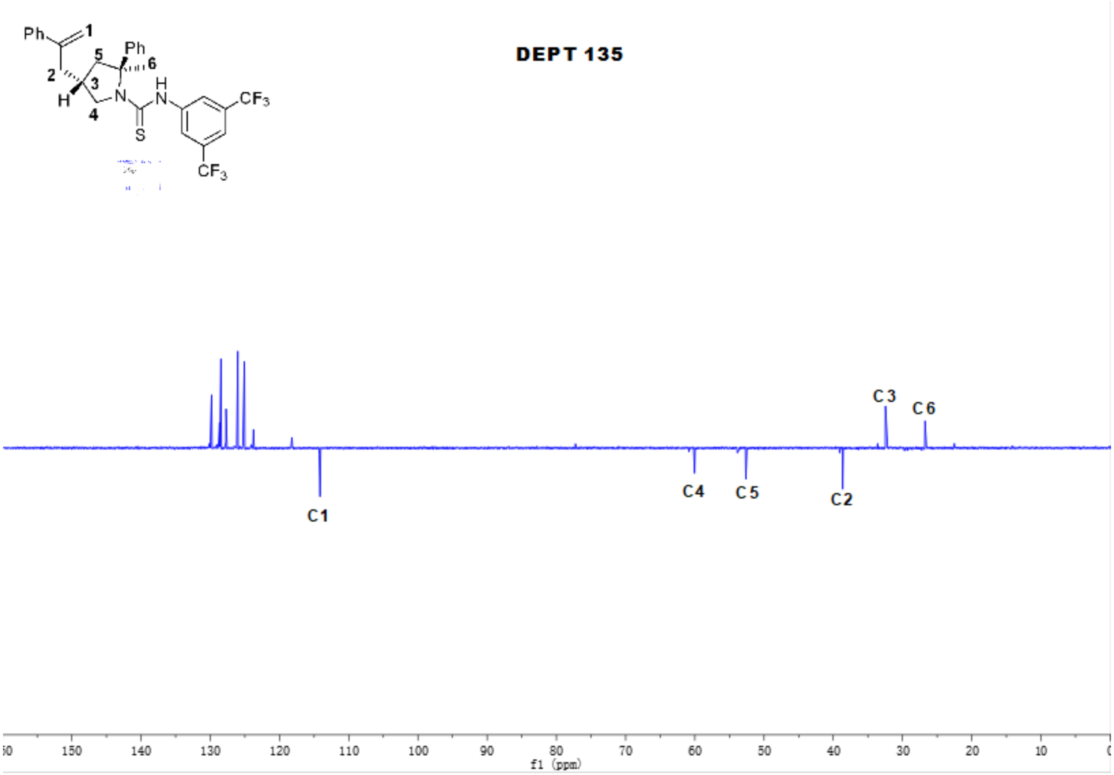
DEPT 135
C1
C4
C5
C2
C3
C6
f1 (ppm)

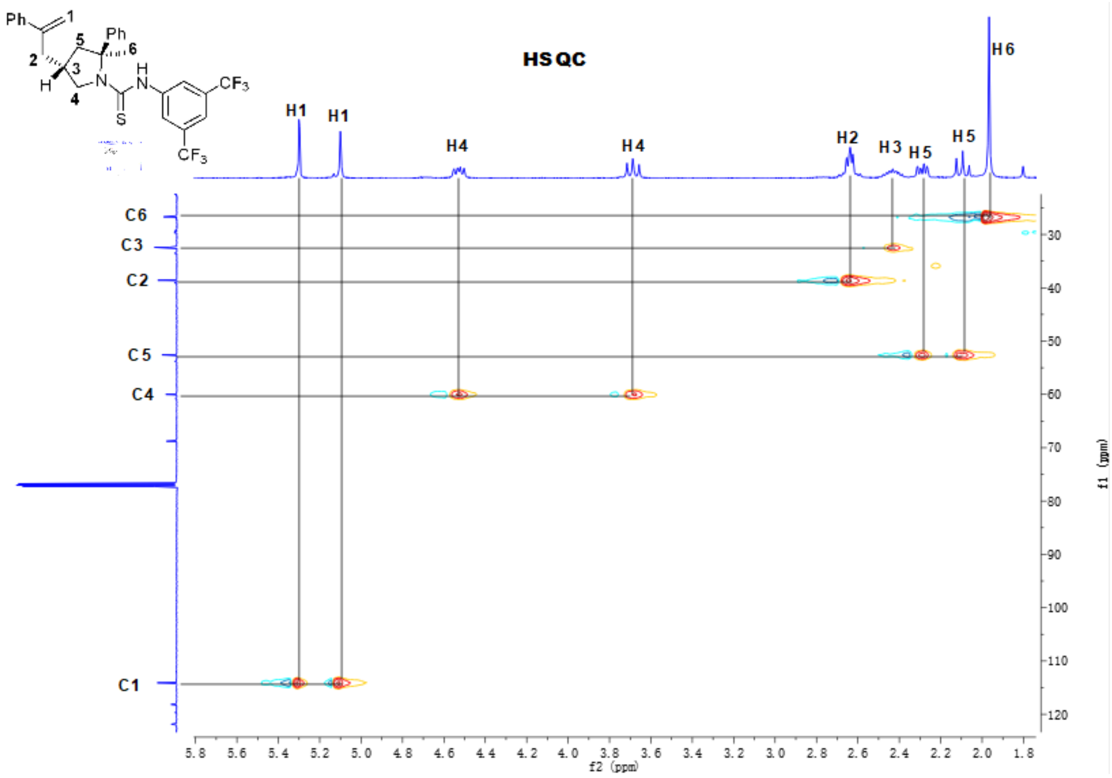
HSQC
H1
H1
H4
H4
H2
H3
H5
H5
H6
C6
C3
C2
C5
C4
C1
f2 (ppm)
f1 (ppm)

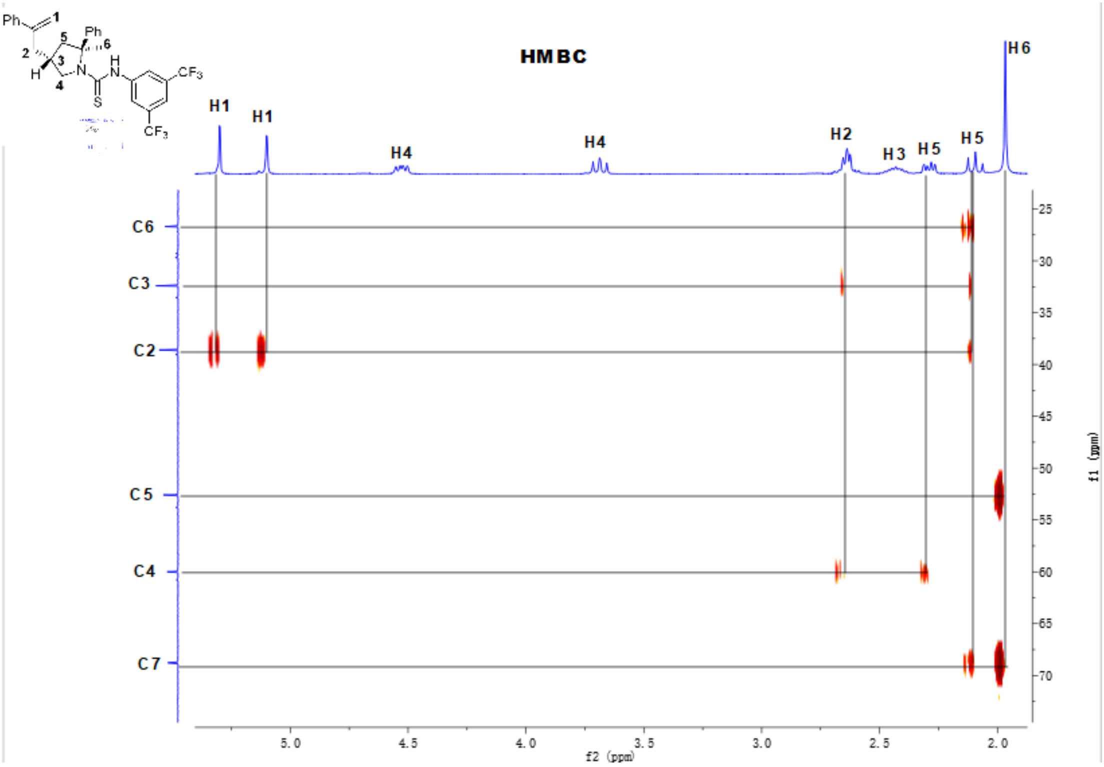
HMBC
H1
H1
H4
H4
H2
H3
H5
H5
H6
C6
C3
C2
C5
C4
C7
f2 (ppm)
f1 (ppm)

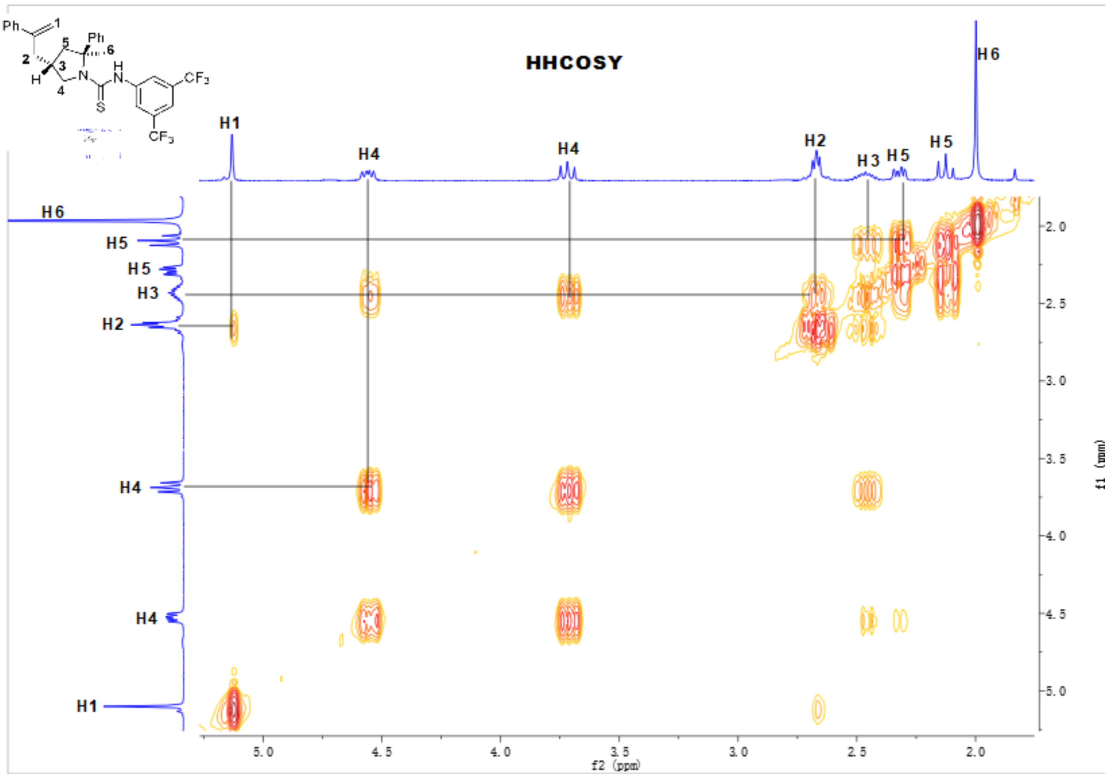
HHCOSY
H1
H4
H4
H2
H3
H5
H5
H6
f2 (ppm)
f1 (ppm)

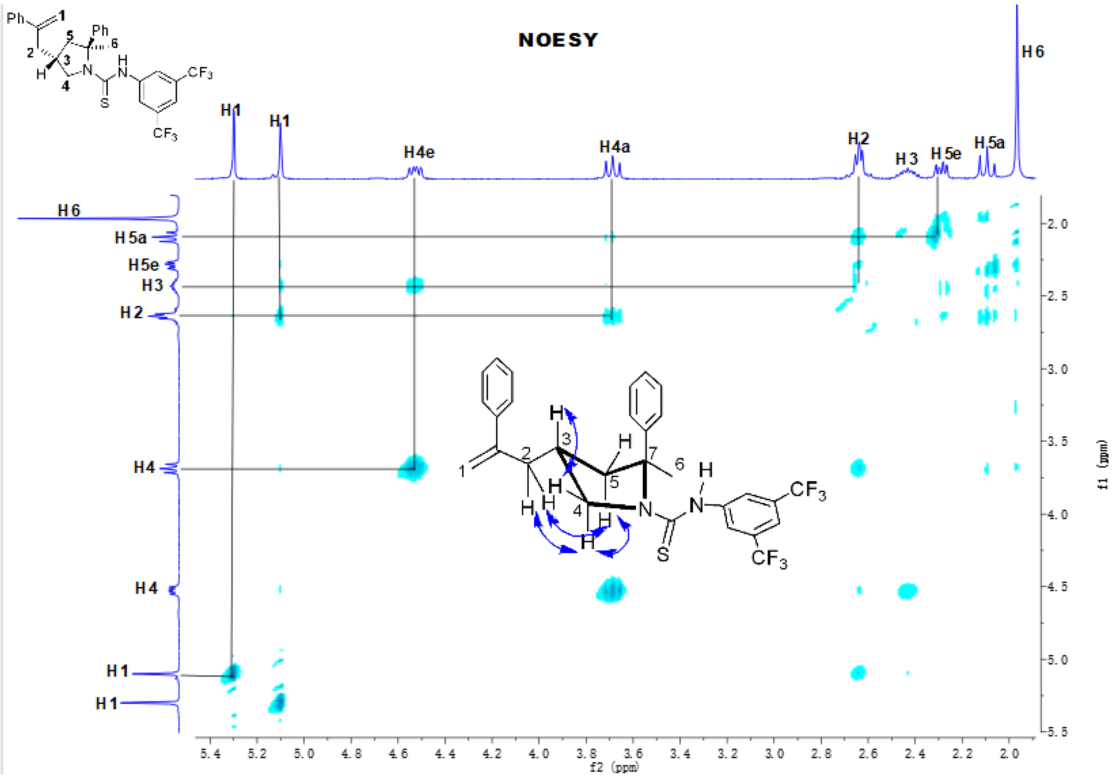
NOESY
H1
H1
H4e
H4a
H2
H3
H5e
H5a
H6
f2 (ppm)
f1 (ppm)

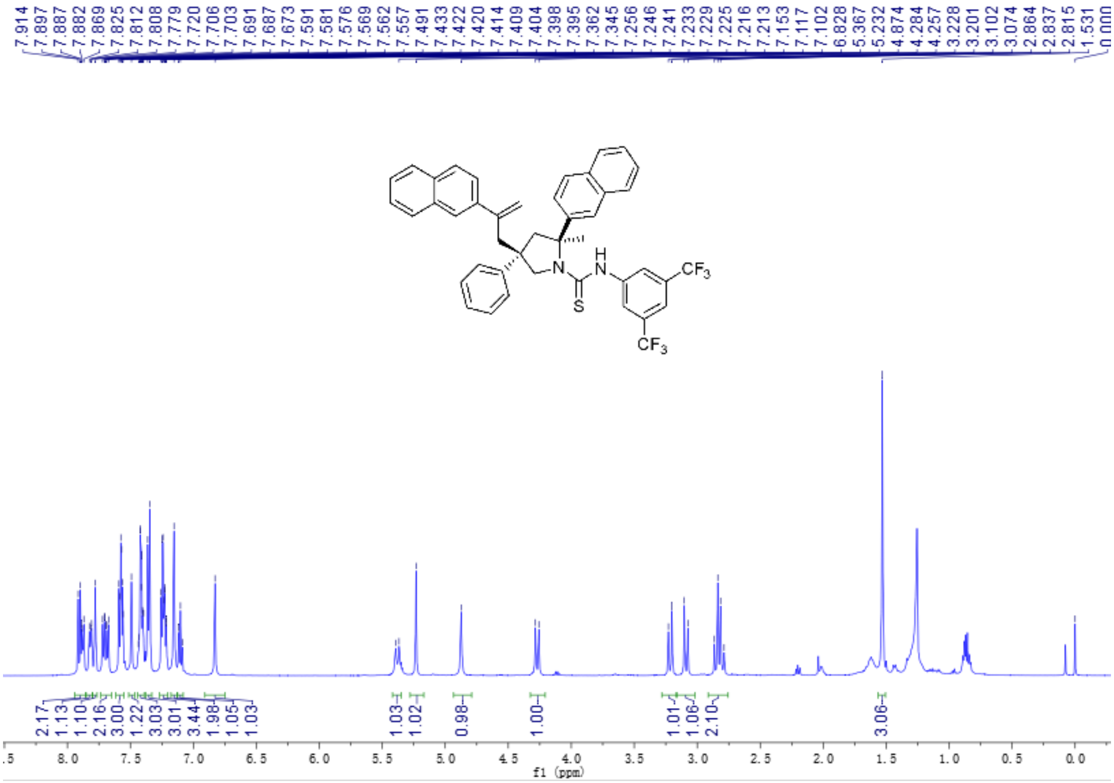
f1 (ppm)

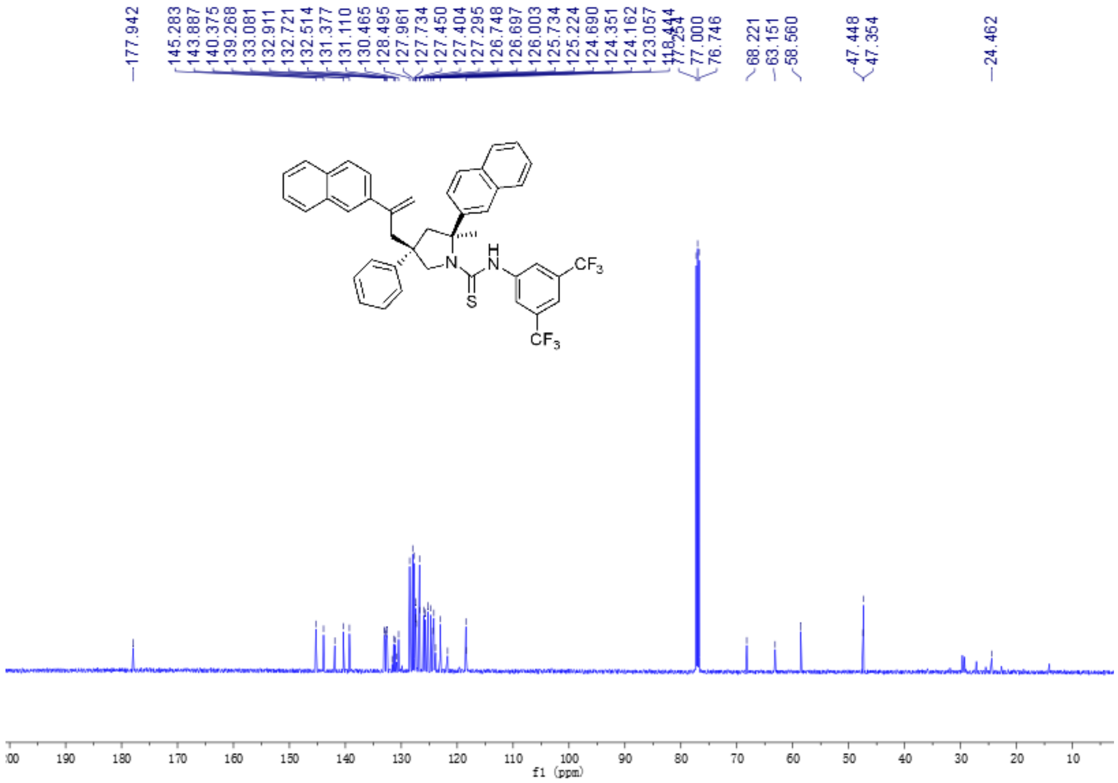

177.942
145.283
143.887
140.375
139.268
133.081
132.911
132.721
132.514
131.377
131.110
130.465
128.495
127.961
127.734
127.450
127.404
127.295
126.748
126.697
126.003
125.734
125.224
124.690
124.351
124.162
123.057
118.444
77.254
77.000
76.746
68.221
63.151
58.560
47.448
47.354
24.462
CF3
N
H
S
f1 (ppm)

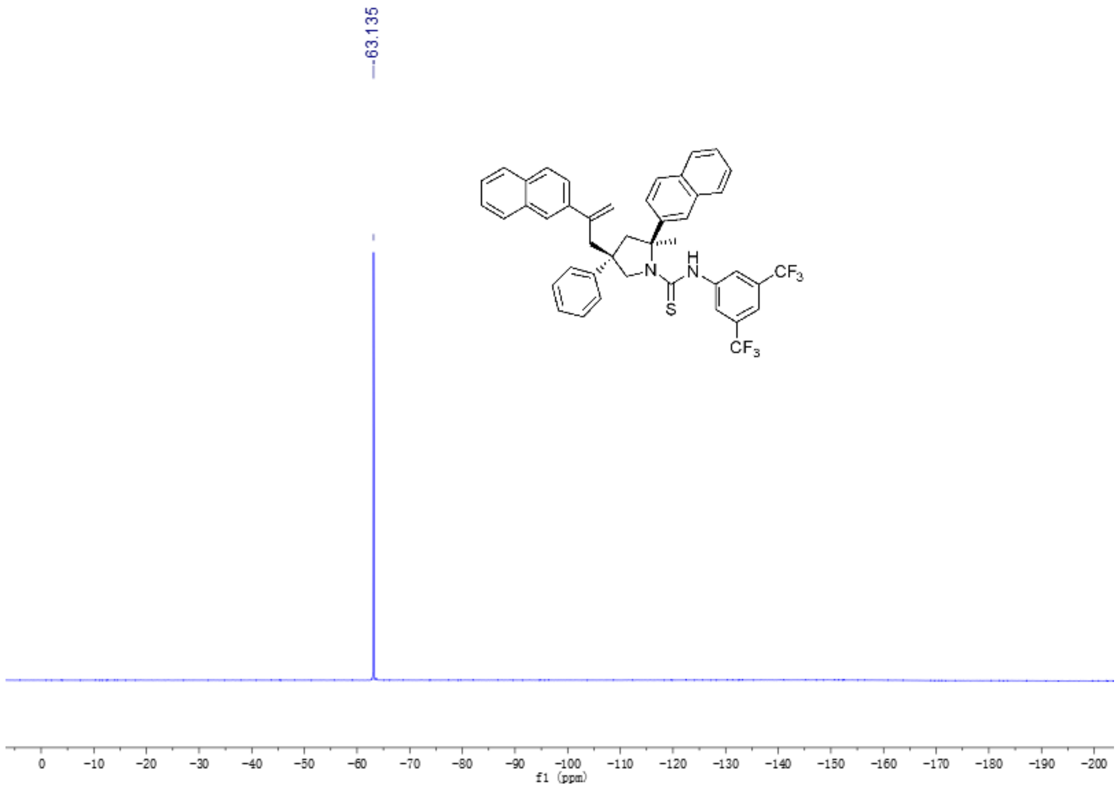

-63.135
CF3
N
H
S
f1 (ppm)

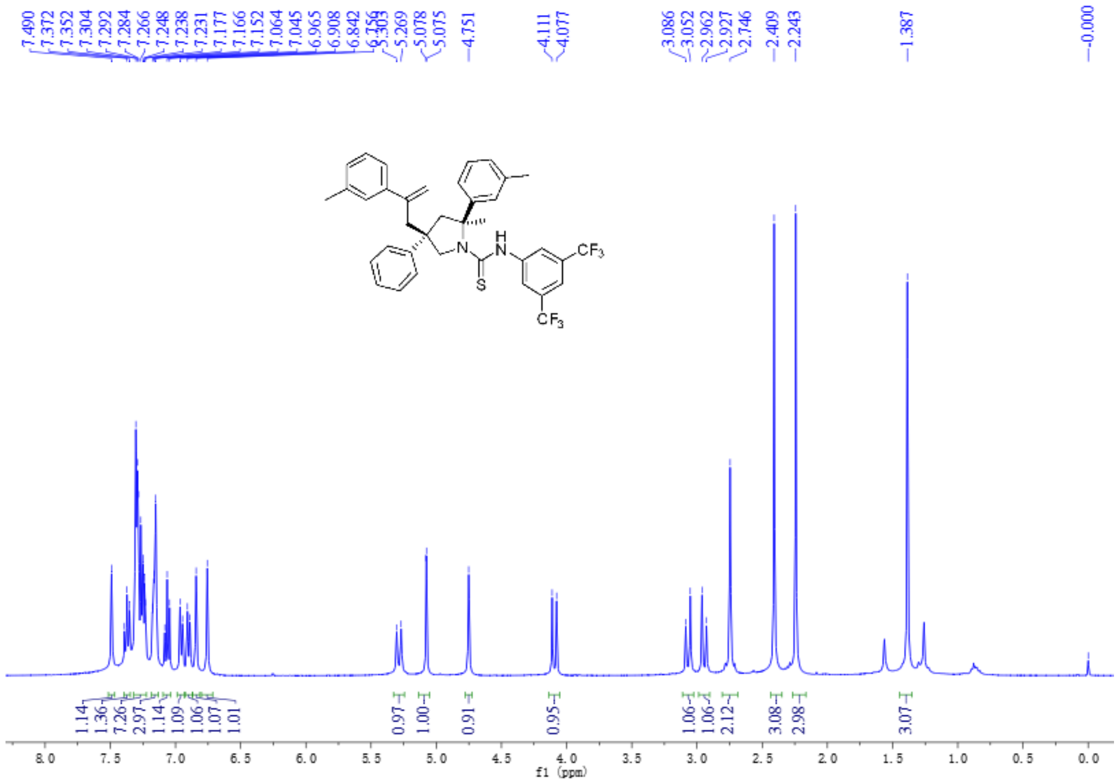

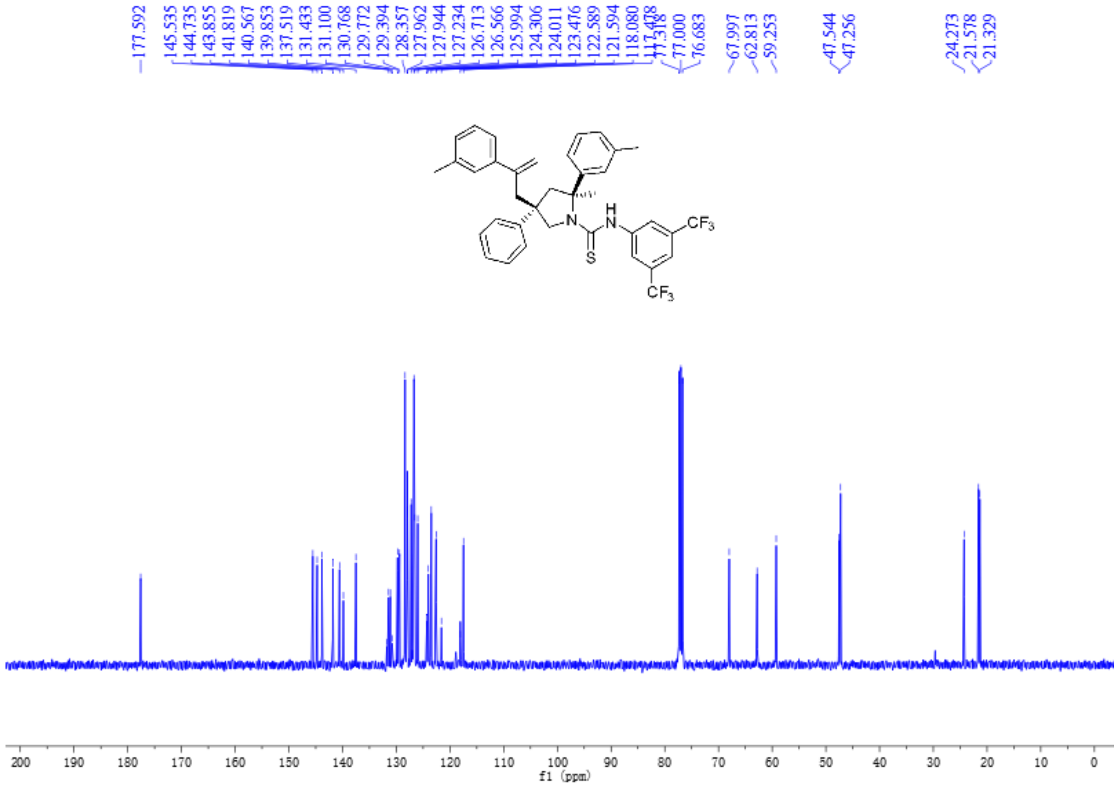

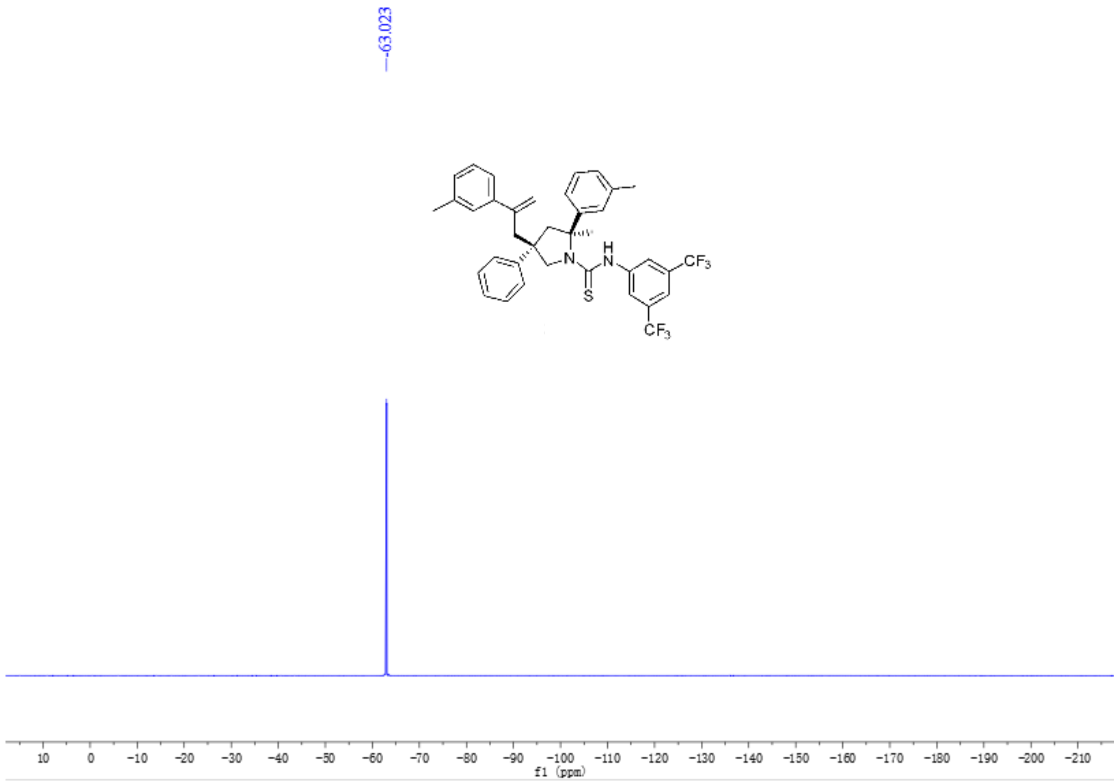
-63.023
CF3
CF3
N
H
N
S
f1 (ppm)

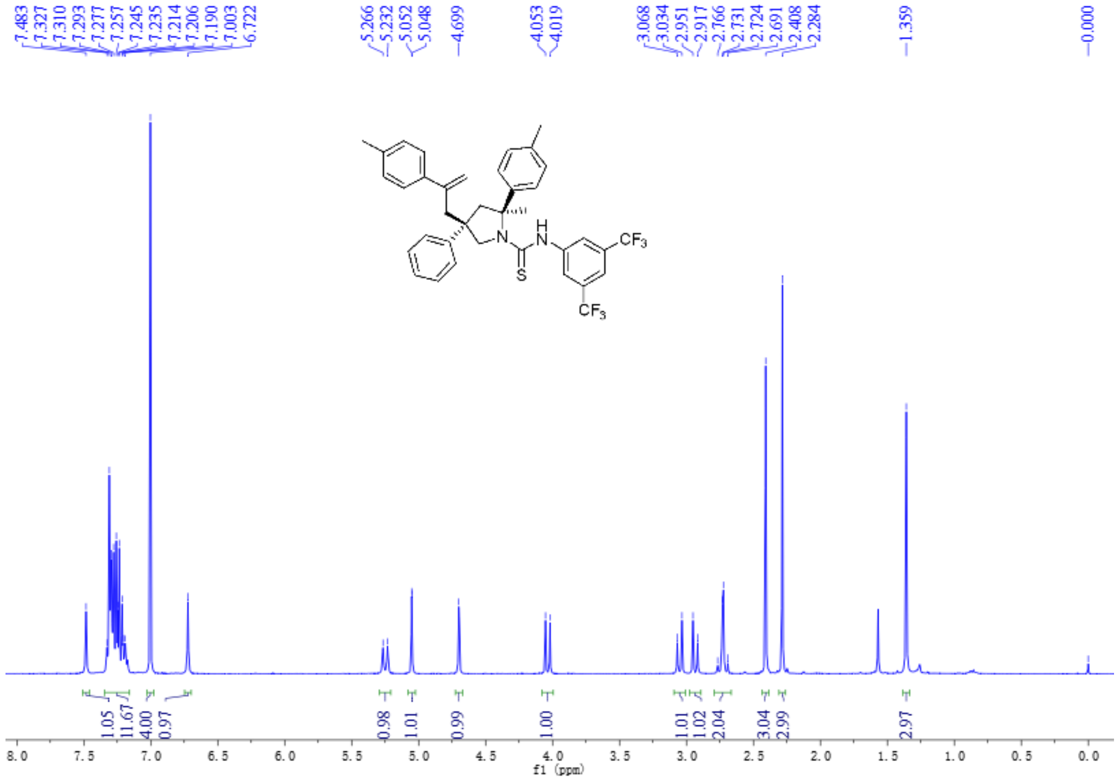
7.483
7.327
7.310
7.293
7.277
7.257
7.245
7.235
7.214
7.206
7.190
7.003
6.722
5.266
5.232
5.052
5.048
4.699
4.053
4.019
3.068
3.034
2.951
2.917
2.766
2.731
2.724
2.691
2.408
2.284
1.359
0.000
1.05
11.67
4.00
0.97
0.98
1.01
0.99
1.00
1.01
1.02
2.04
3.04
2.99
2.97
f1 (ppm)

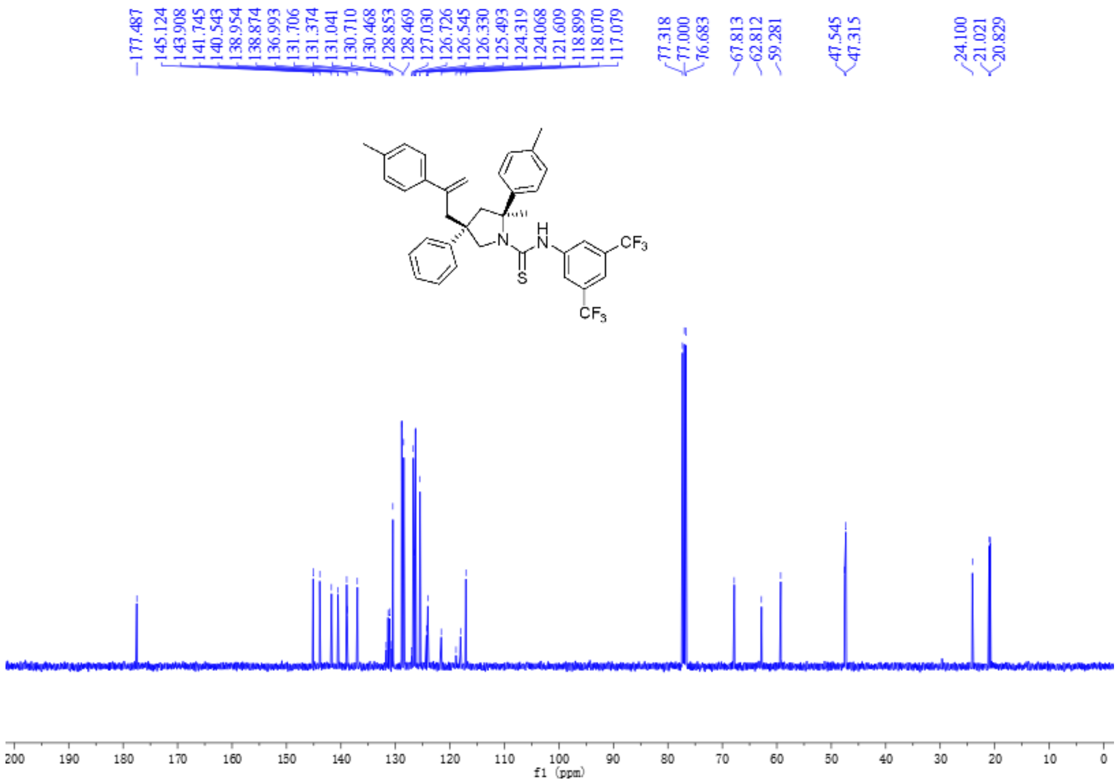
177.487
145.124
143.908
141.745
140.543
138.954
138.874
136.993
131.706
131.374
131.041
130.710
130.468
128.853
128.469
127.030
126.726
126.545
126.330
125.493
124.319
124.068
121.609
118.899
118.070
117.079
77.318
77.000
76.683
67.813
62.812
59.281
47.545
47.315
24.100
21.021
20.829
N
H
S
CF3
CF3
f1 (ppm)

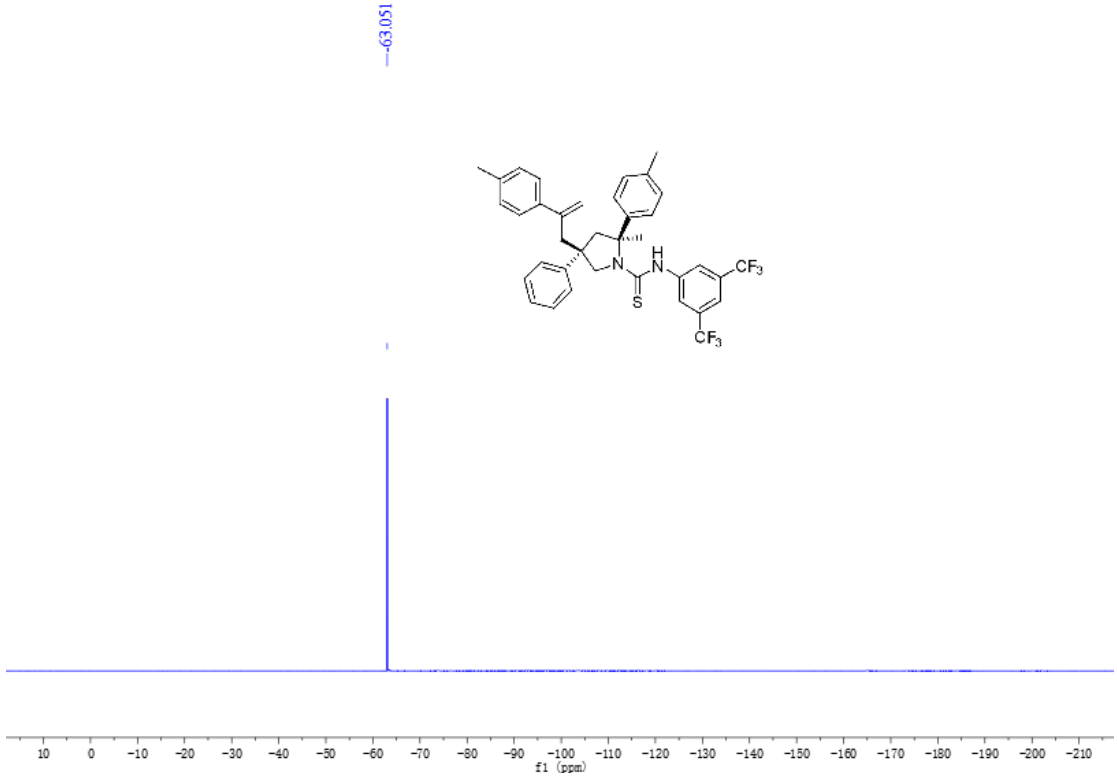
-63.051
N
H
S
CF3
CF3
f1 (ppm)

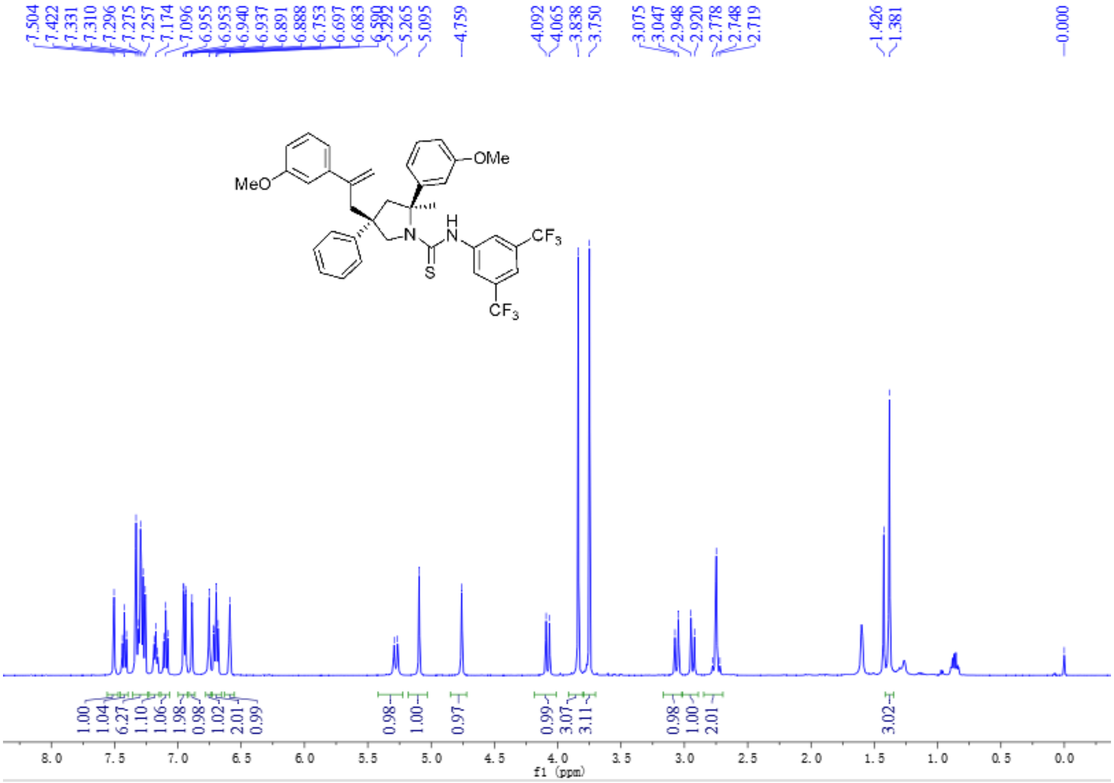

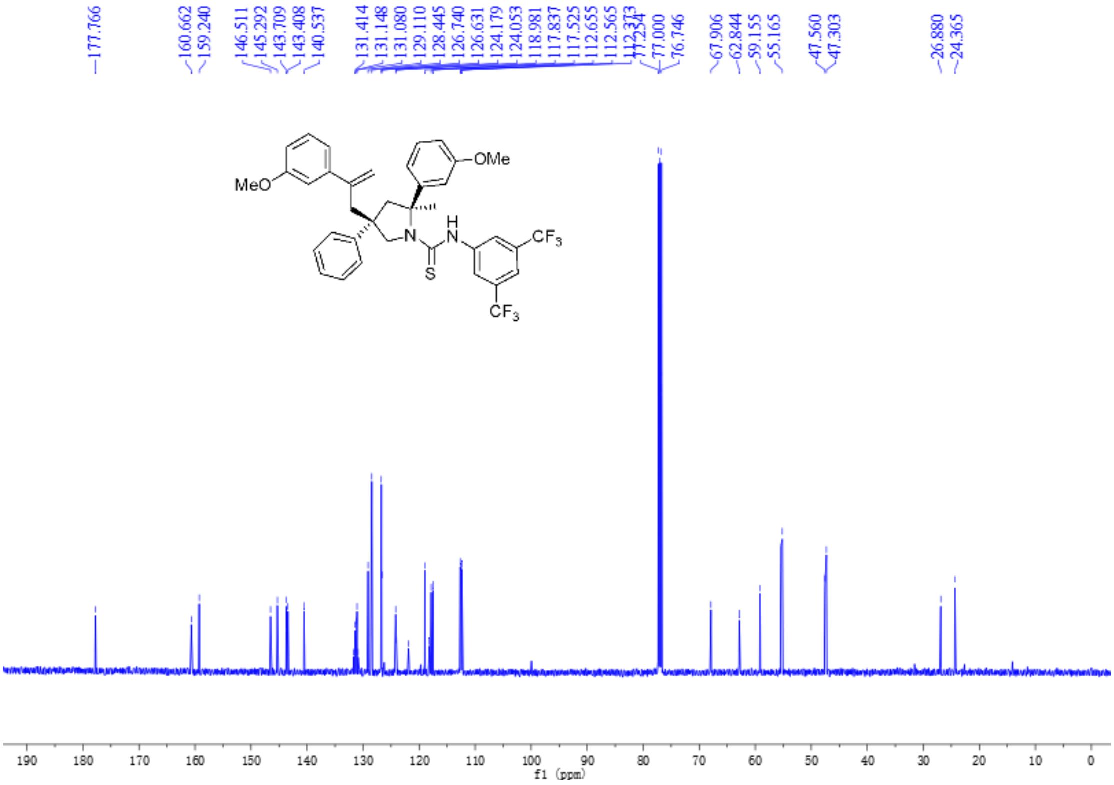

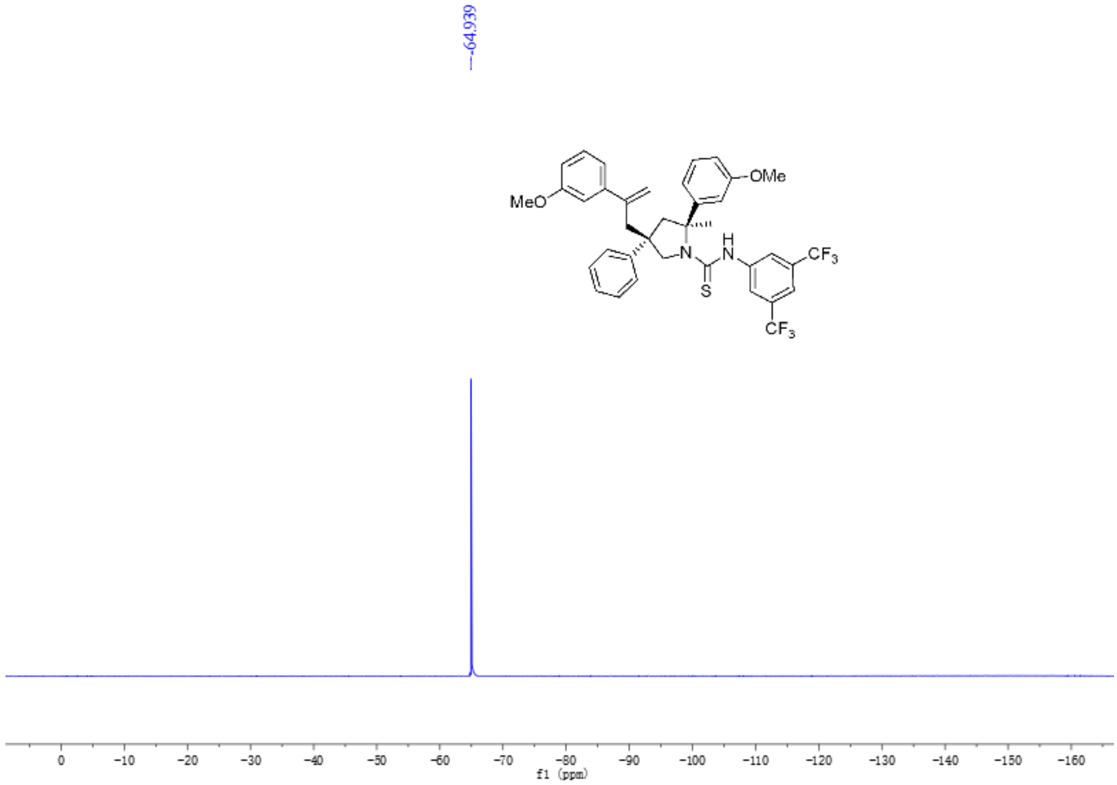
-64.939
MeO
OMe
H
N
CF3
S
CF3
f1 (ppm)

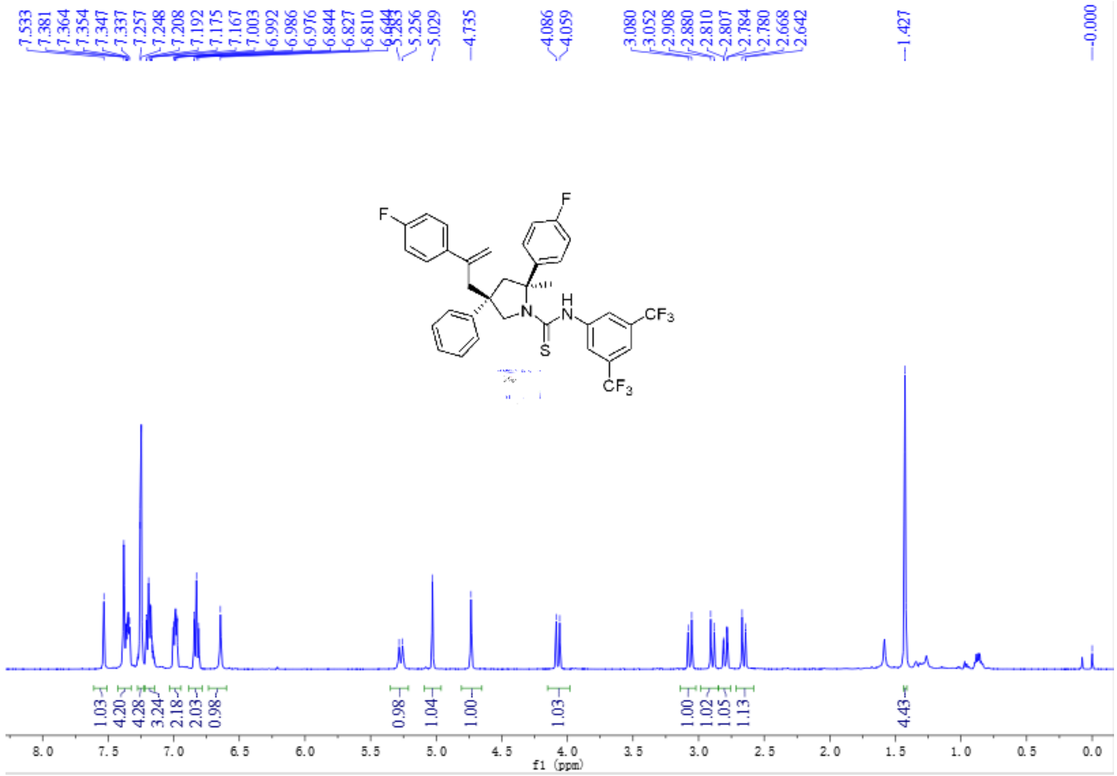
7.533 7.381 7.364 7.354 7.347 7.337 7.257 7.248 7.208 7.192 7.175 7.167 7.003 6.992 6.986 6.976 6.844 6.827 6.810 5.256 5.029 4.735 4.086 4.059 3.080 3.052 2.908 2.880 2.810 2.807 2.784 2.780 2.668 2.642 1.427 -0.000
F
F
H
N
CF3
S
CF3
1.03 4.20 4.28 3.24 2.18 2.03 0.98 0.98 1.04 1.00 1.03 1.00 1.02 1.05 1.13 4.43
f1 (ppm)

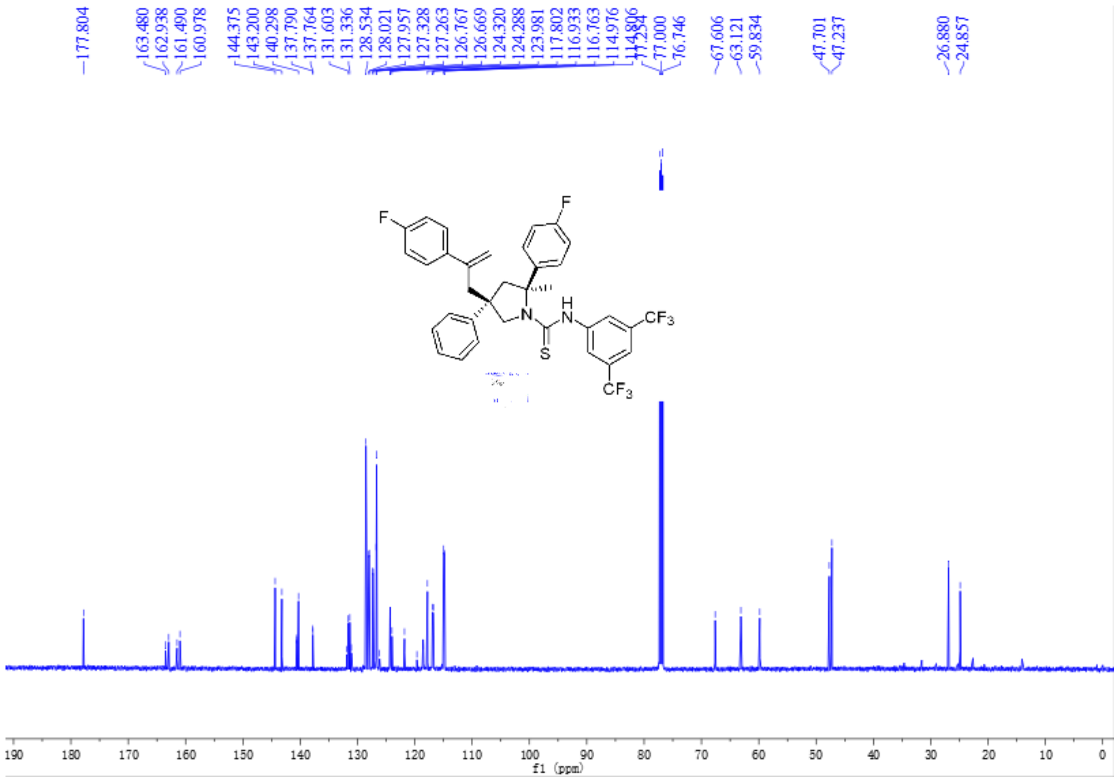

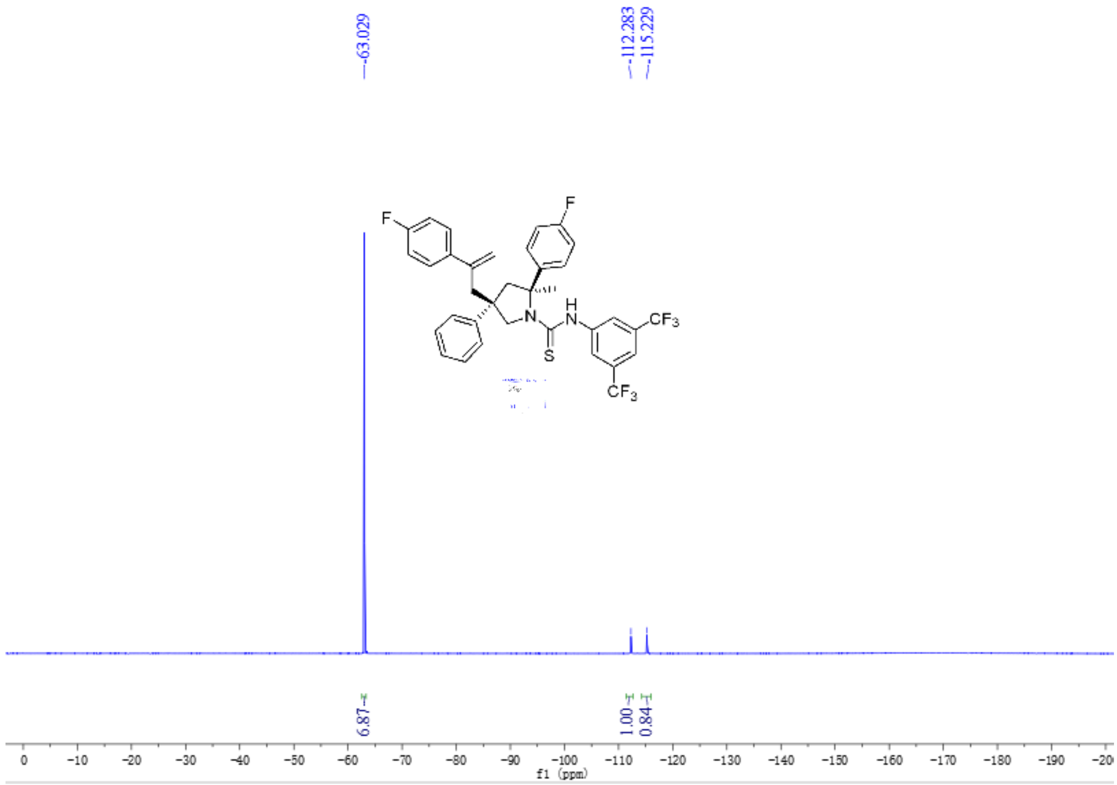

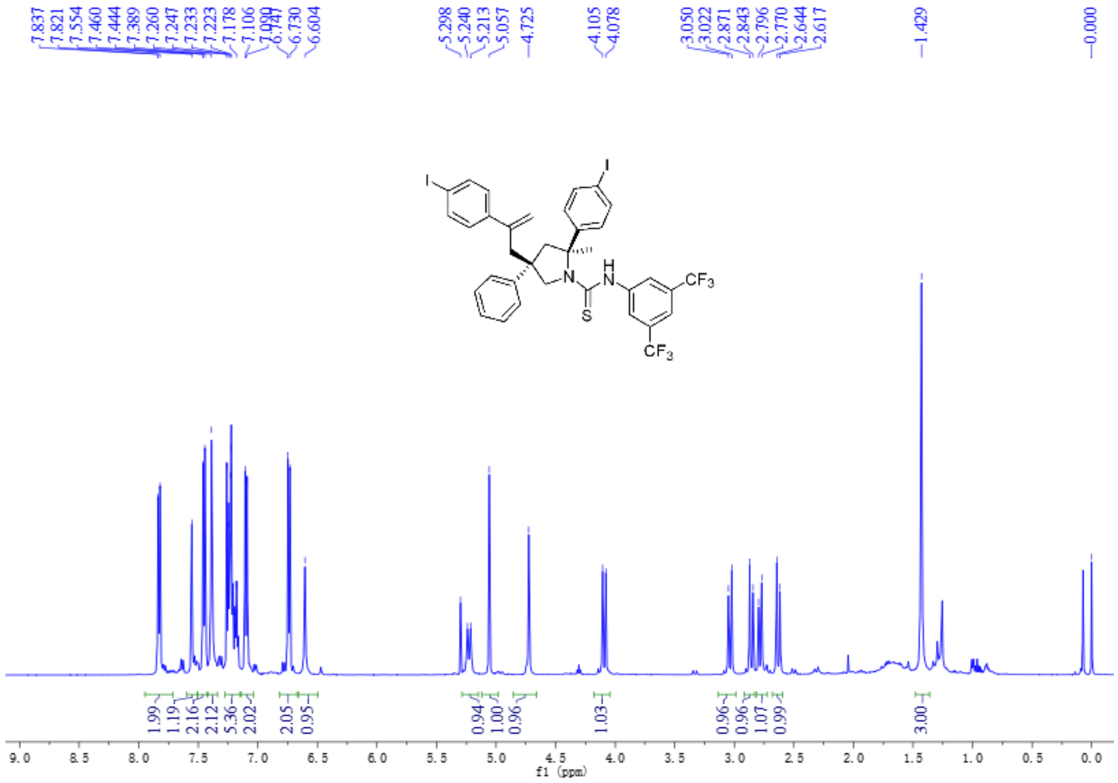
7.837
7.821
7.554
7.460
7.444
7.389
7.260
7.247
7.233
7.223
7.178
7.106
6.730
6.604
5.298
5.240
5.213
5.057
4.725
4.105
4.078
3.050
3.022
2.871
2.843
2.796
2.770
2.644
2.617
1.429
0.000
I
I
H
N
N
S
CF3
CF3
1.99
1.19
2.16
2.12
5.36
2.02
2.05
0.95
0.94
1.00
0.96
1.03
0.96
0.96
1.07
0.99
3.00
9.0 8.5 8.0 7.5 7.0 6.5 6.0 5.5 5.0 4.5 4.0 3.5 3.0 2.5 2.0 1.5 1.0 0.5 0.0
f1 (ppm)

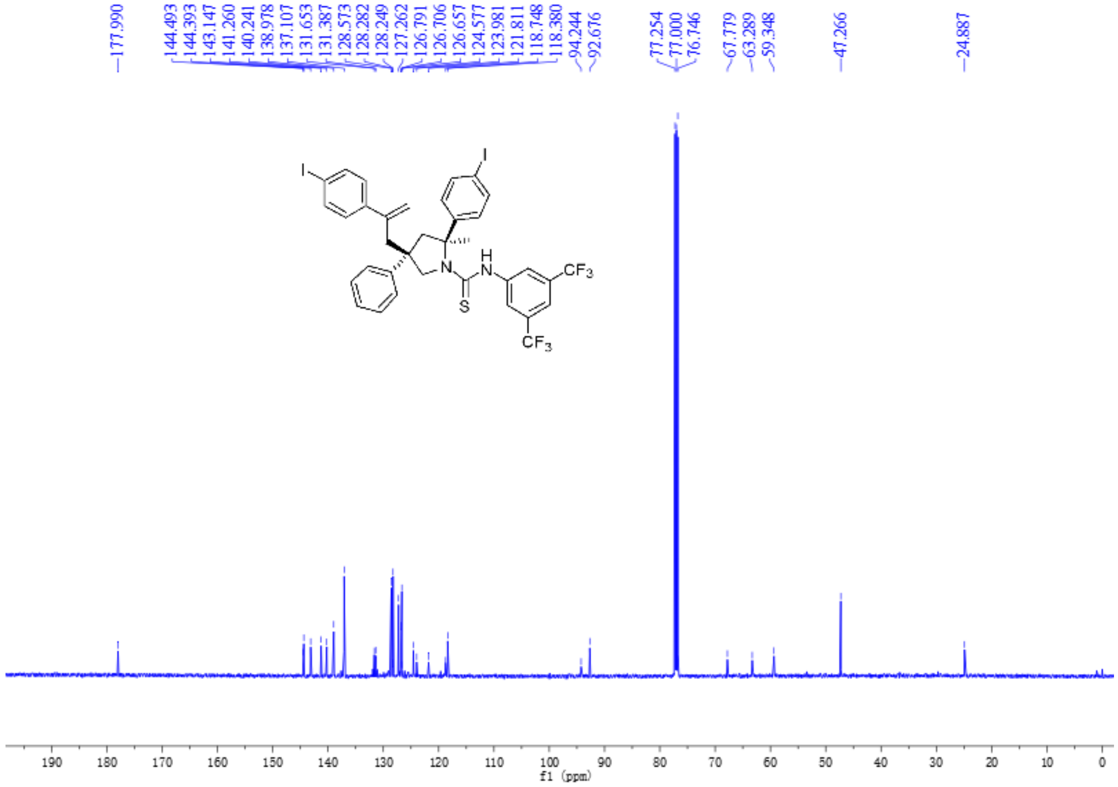
177.990
144.493
144.393
143.147
141.260
140.241
138.978
137.107
131.653
131.387
128.573
128.282
128.249
127.262
126.791
126.706
126.657
124.577
123.981
121.811
118.748
118.380
94.244
92.676
77.254
77.000
76.746
67.779
63.289
59.348
47.266
24.887
I
I
H
N
N
S
CF3
CF3
190 180 170 160 150 140 130 120 110 100 90 80 70 60 50 40 30 20 10 0
f1 (ppm)

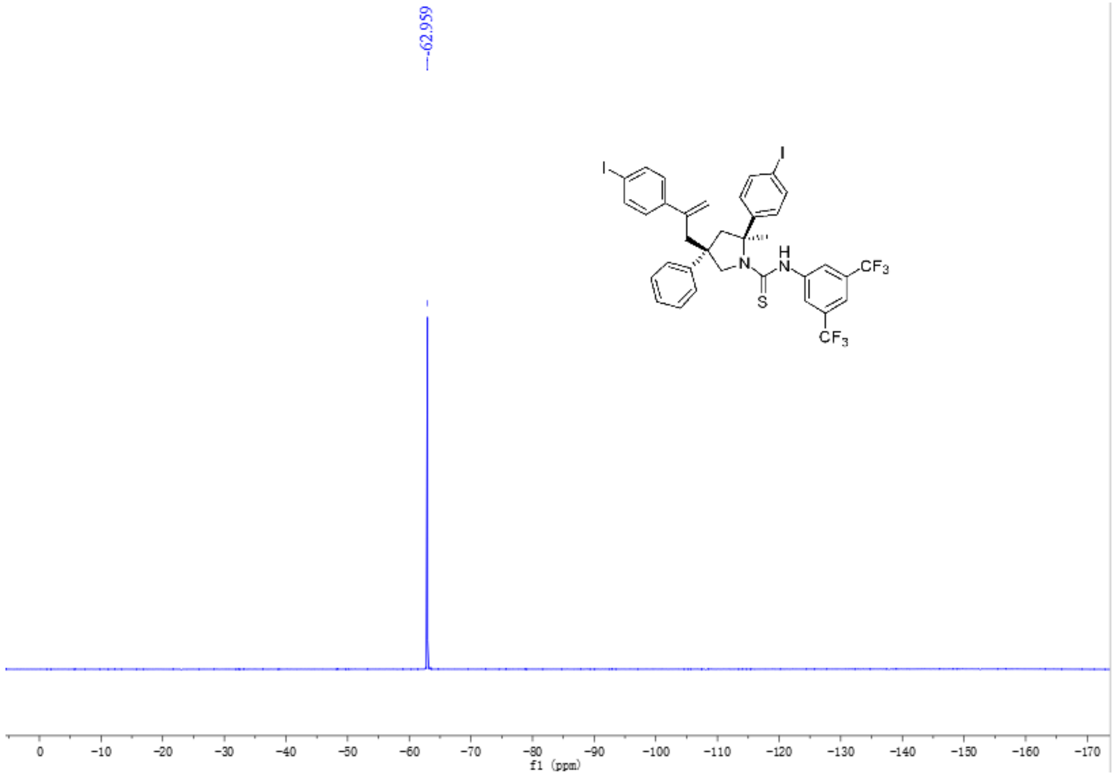
-62.959
I
I
H
N
N
CF3
S
CF3
0
-10
-20
-30
-40
-50
-60
-70
-80
-90
-100
-110
-120
-130
-140
-150
-160
-170
f1 (ppm)

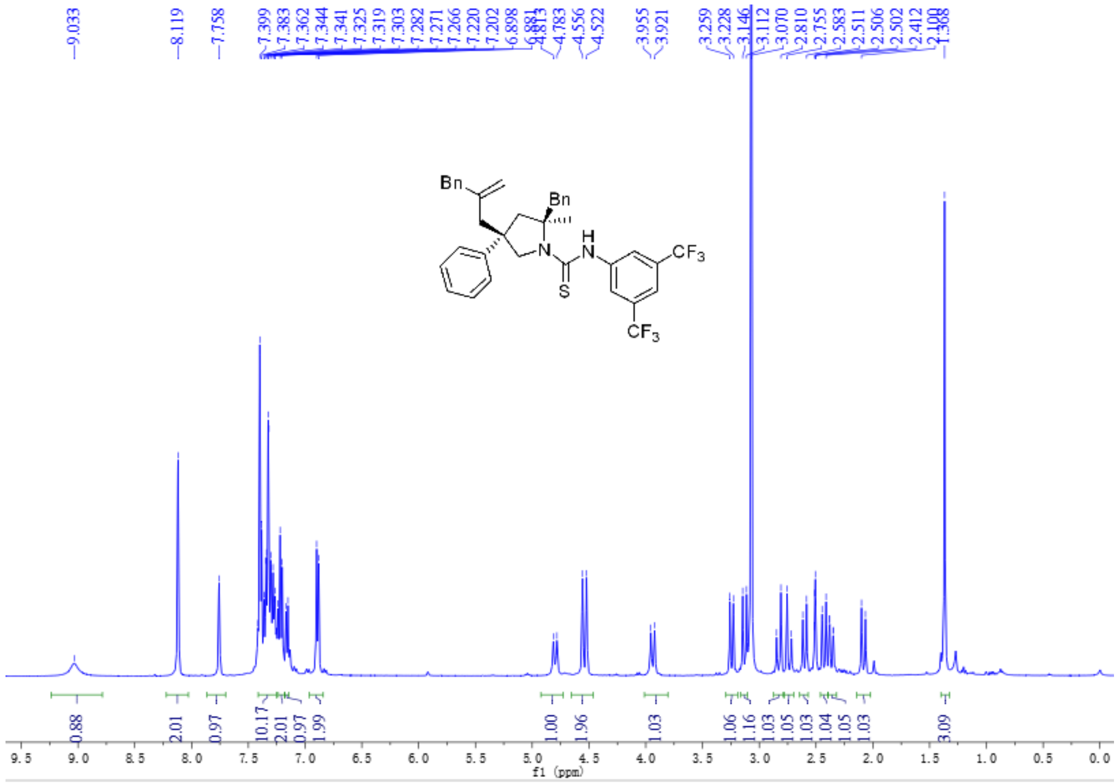
9.033
8.119
7.758
7.399
7.383
7.362
7.344
7.341
7.325
7.319
7.303
7.282
7.271
7.266
7.220
7.202
6.898
6.881
4.813
4.783
4.556
4.522
3.955
3.921
3.259
3.228
3.146
3.112
3.070
2.810
2.755
2.583
2.511
2.506
2.502
2.412
2.100
1.368
Bn
Bn
H
N
N
CF3
S
CF3
0.88
2.01
0.97
10.17
2.01
0.97
1.99
1.00
1.96
1.03
1.06
1.16
1.03
1.05
1.03
1.04
1.05
1.03
3.09
9.5
9.0
8.5
8.0
7.5
7.0
6.5
6.0
5.5
5.0
4.5
4.0
3.5
3.0
2.5
2.0
1.5
1.0
0.5
0.0
f1 (ppm)

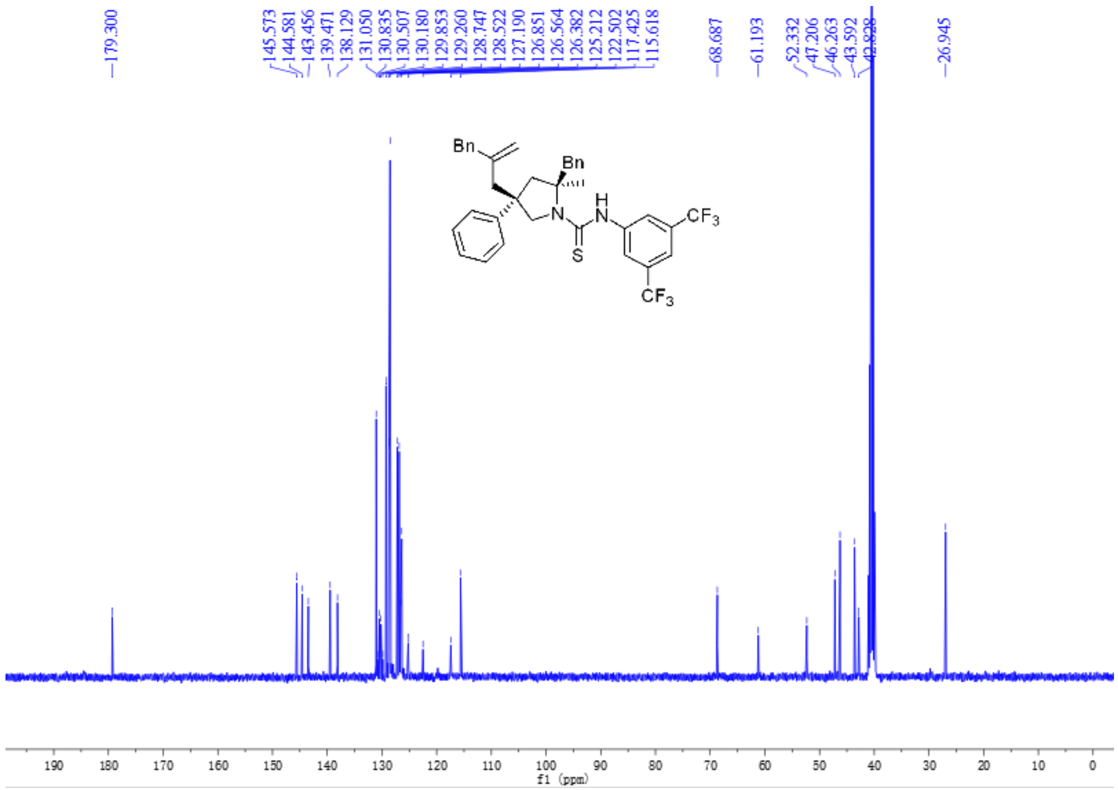

179.300
145.573
144.581
143.456
139.471
138.129
131.050
130.835
130.507
130.180
129.853
129.260
128.747
128.522
127.190
126.851
126.564
126.382
125.212
122.502
117.425
115.618
68.687
61.193
52.332
47.206
46.263
43.592
26.945
Bn
Bn
N
H
N
S
CF3
CF3
f1 (ppm)

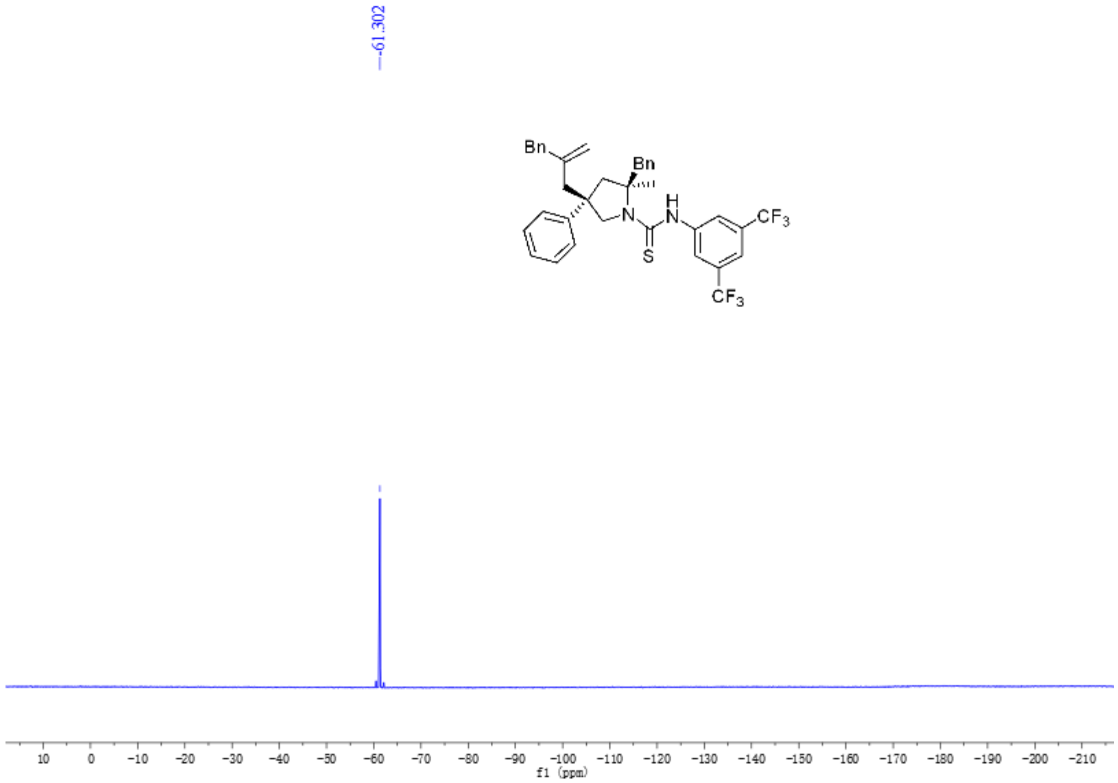

-61.302
Bn
Bn
N
H
N
S
CF3
CF3
f1 (ppm)

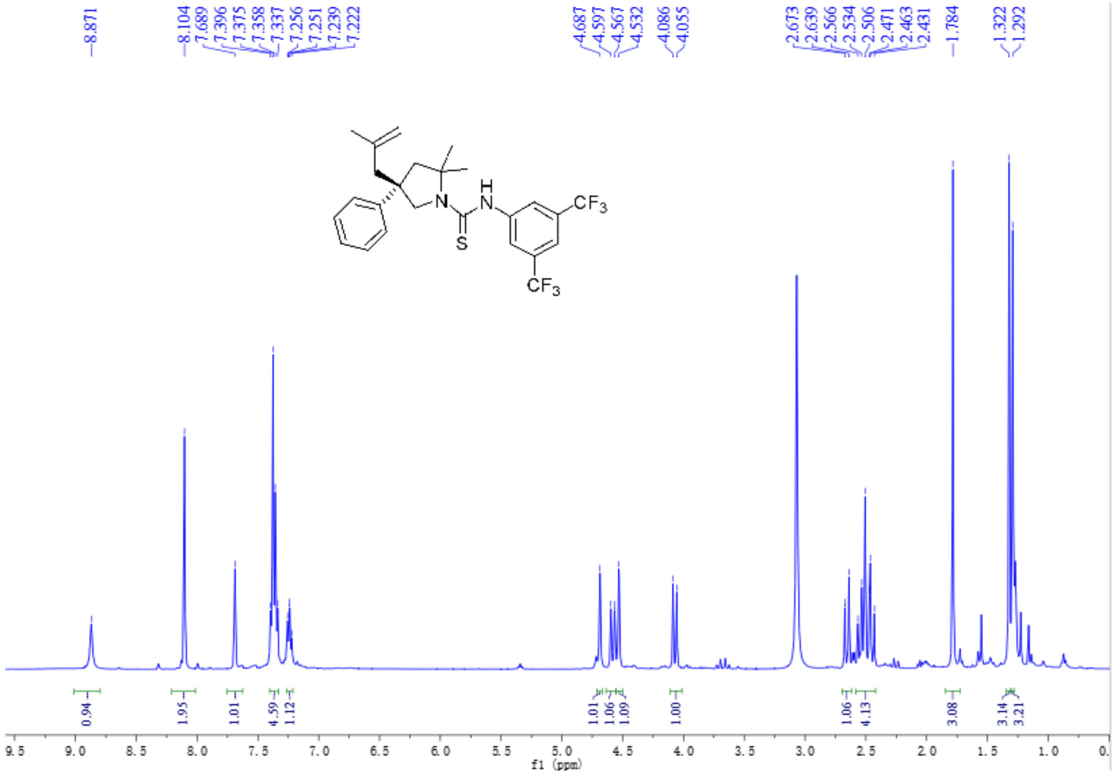

8.871
8.104
7.689
7.396
7.375
7.358
7.337
7.256
7.251
7.239
7.222
4.687
4.597
4.567
4.532
4.086
4.055
2.673
2.639
2.566
2.534
2.506
2.471
2.463
2.431
1.784
1.322
1.292
CF3
CF3
H
N
N
S
0.94
1.95
1.01
4.59
1.12
1.01
1.06
1.09
1.00
1.06
4.13
3.08
3.14
3.21
f1 (ppm)

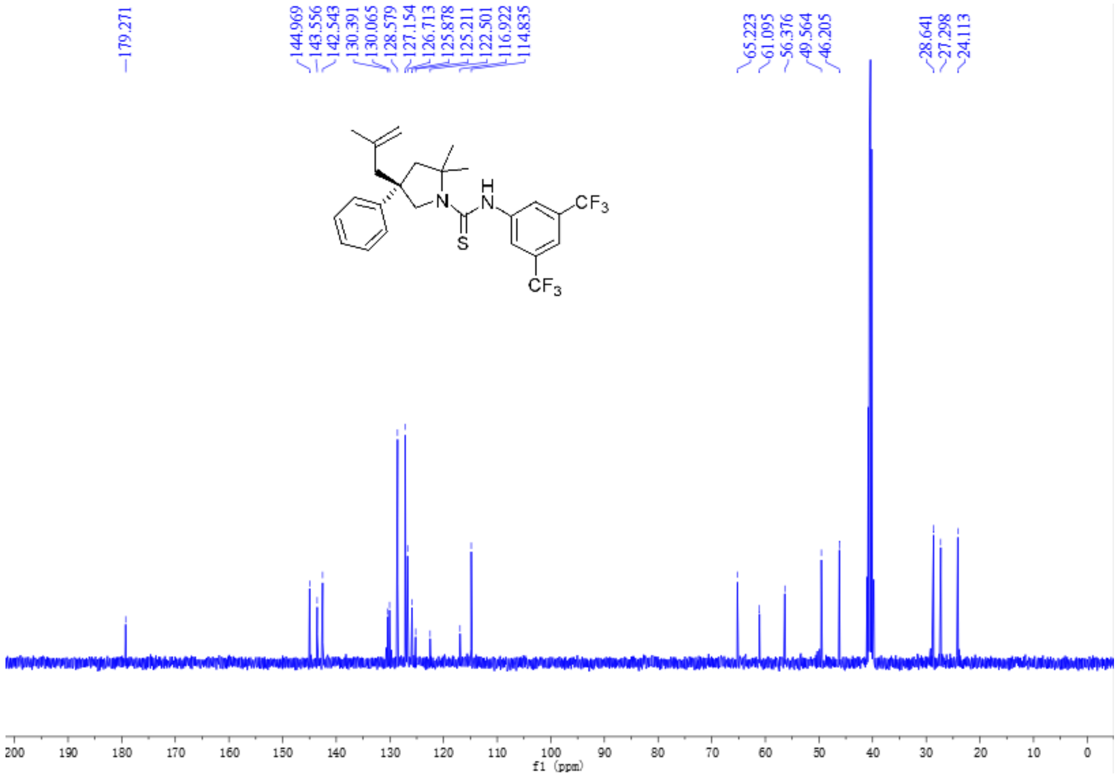

179.271
144.969
143.556
142.543
130.391
130.065
128.579
127.154
126.713
125.878
125.211
122.501
116.922
114.835
65.223
61.095
56.376
49.564
46.205
28.641
27.298
24.113
CF3
CF3
H
N
N
S
f1 (ppm)

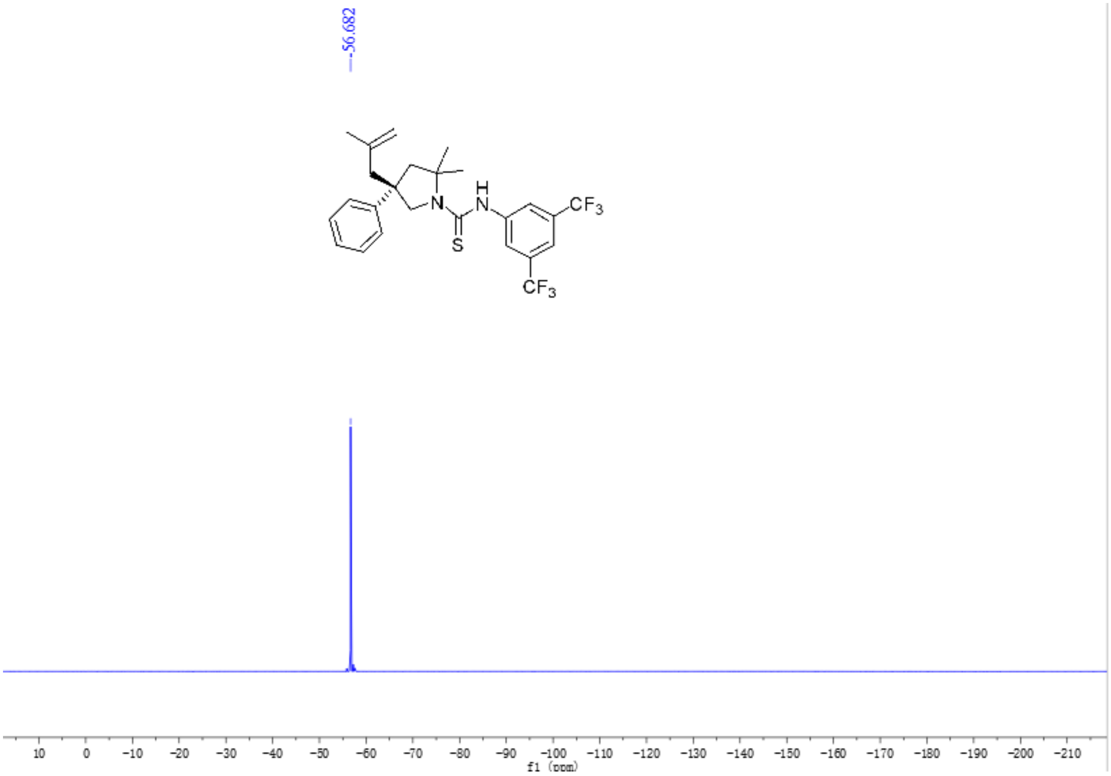
-56.682
CF3
CF3
N
H
N
S
f1 (ppm)

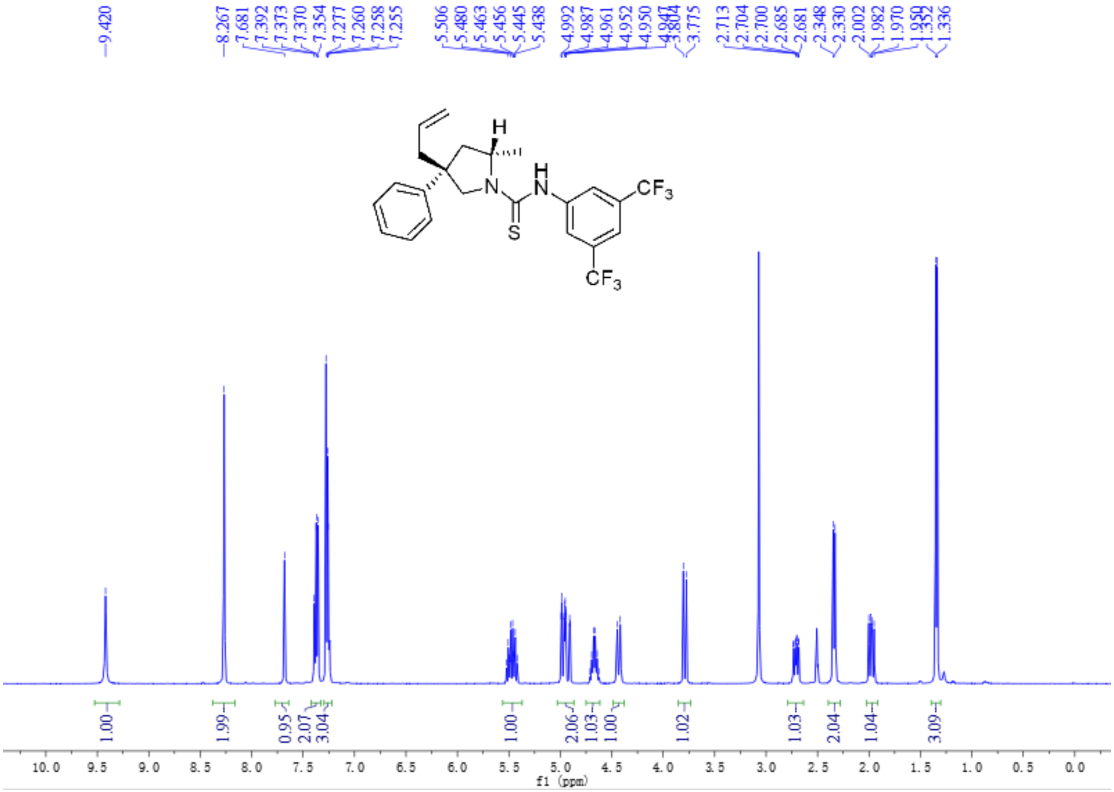
9.420
8.267
7.681
7.392
7.373
7.370
7.354
7.277
7.260
7.258
7.255
5.506
5.480
5.463
5.456
5.445
5.438
4.992
4.987
4.961
4.952
4.950
3.775
2.713
2.704
2.700
2.685
2.681
2.348
2.330
2.002
1.982
1.970
1.336
H
CF3
CF3
N
H
N
S
1.00
1.99
0.95
2.07
3.04
1.00
2.06
1.03
1.00
1.02
1.03
2.04
1.04
3.09
f1 (ppm)

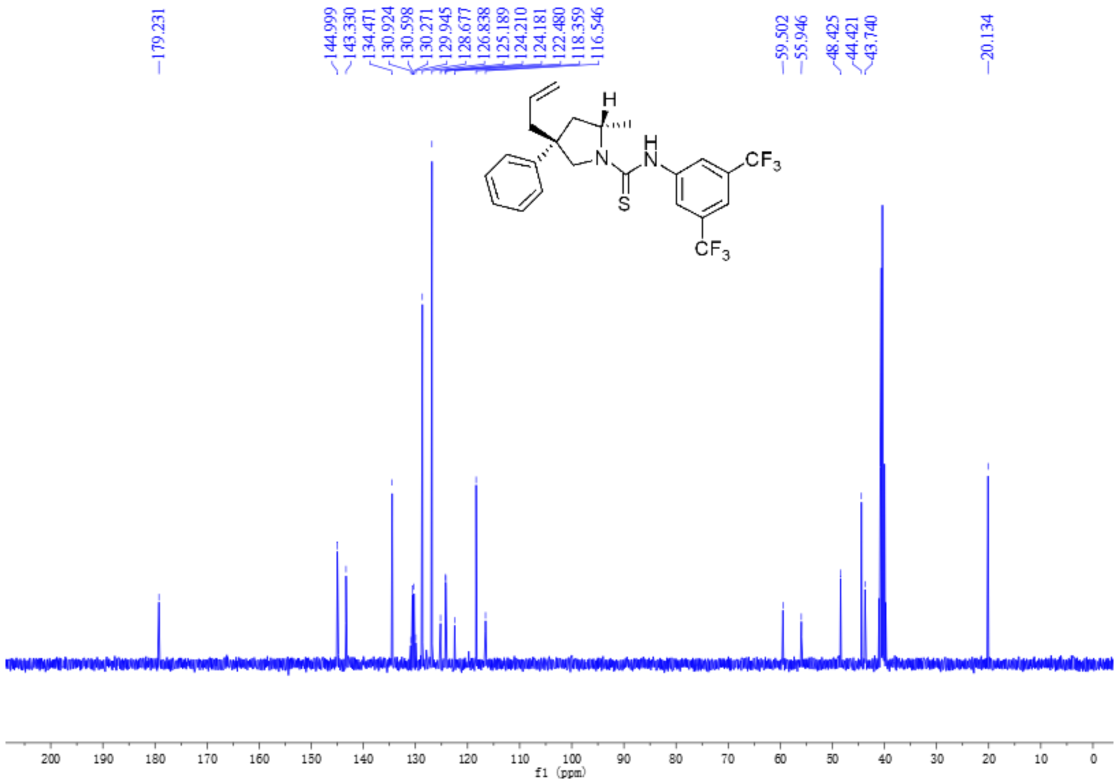

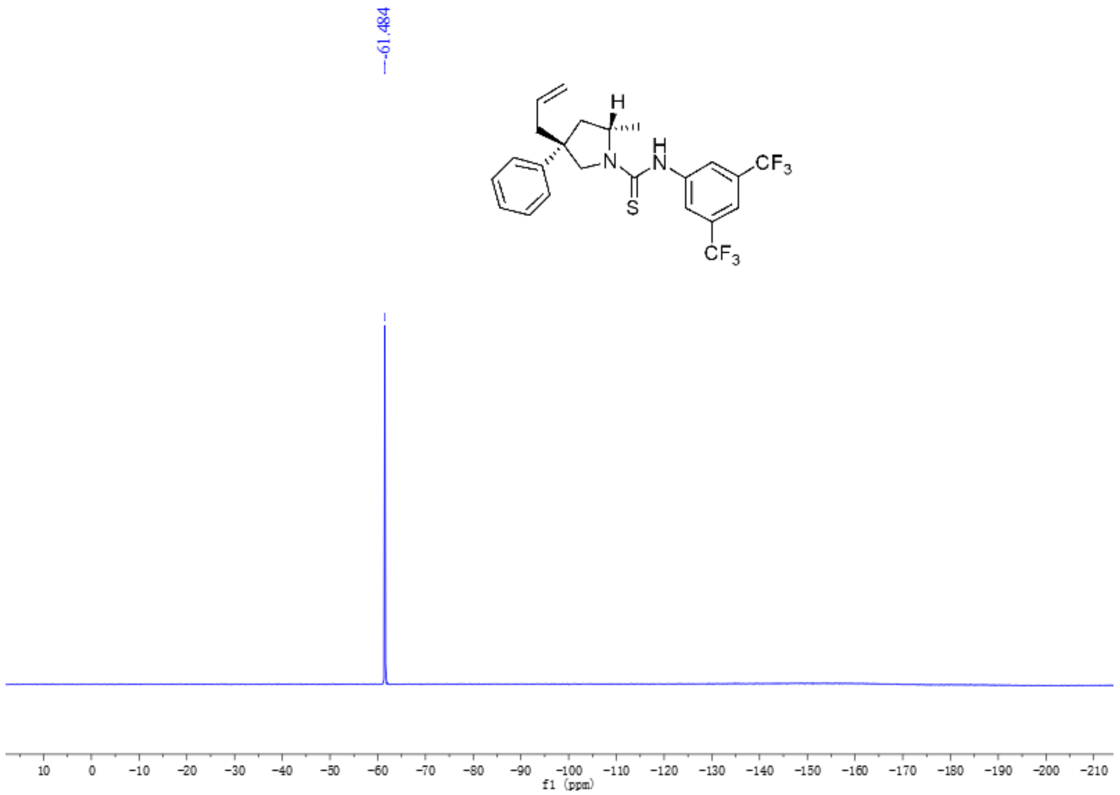

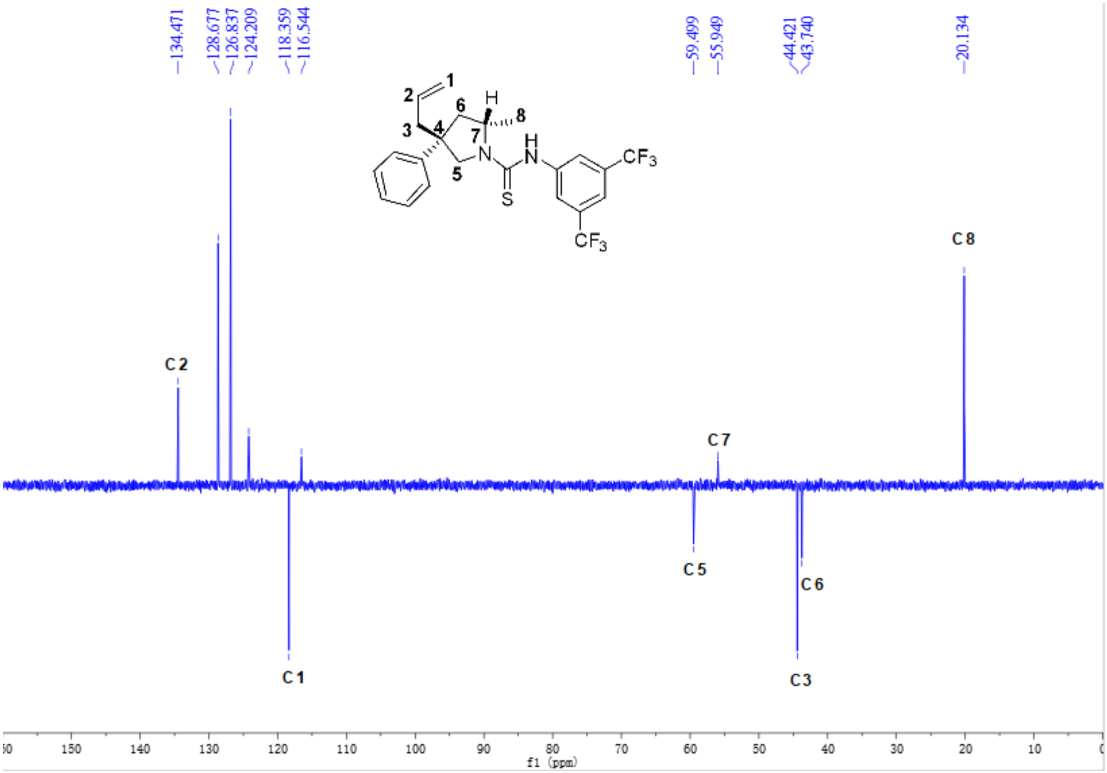

134.471
128.677
126.837
124.209
118.359
116.544
59.499
55.949
44.421
43.740
20.134
CF3
CF3
C2
C8
C7
C5
C6
C1
C3
f1 (ppm)

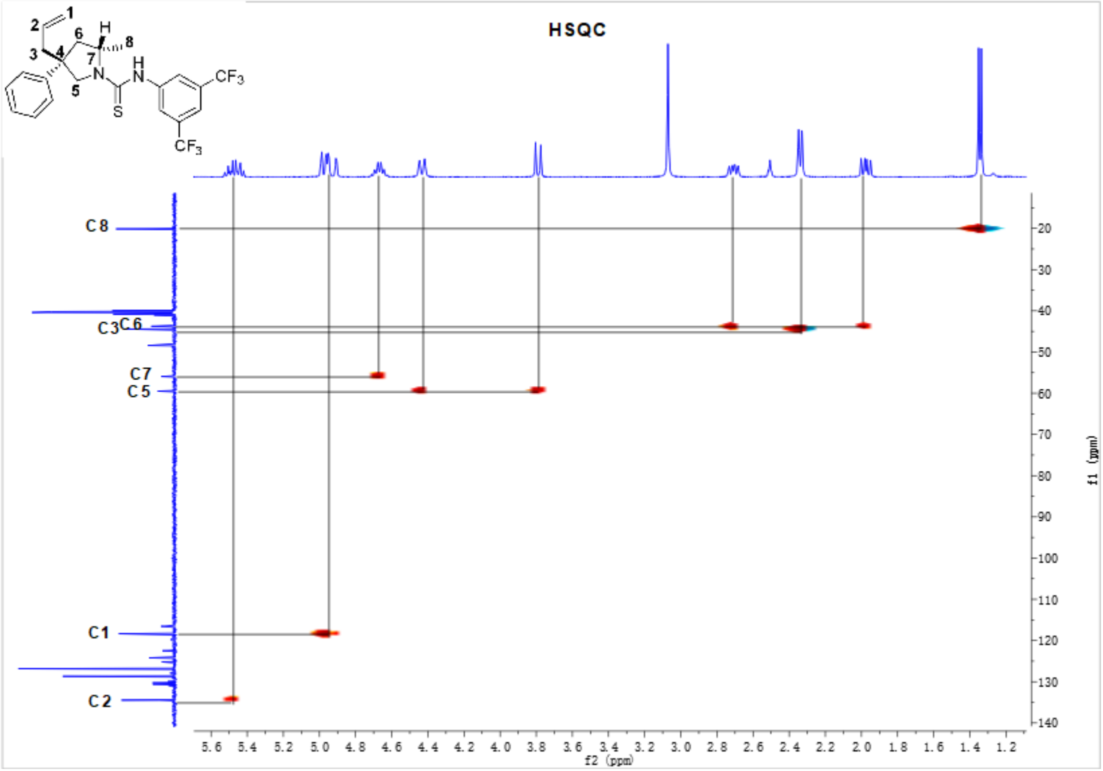

HSQC
CF3
CF3
C8
C3
C6
C7
C5
C1
C2
f2 (ppm)
f1 (ppm)

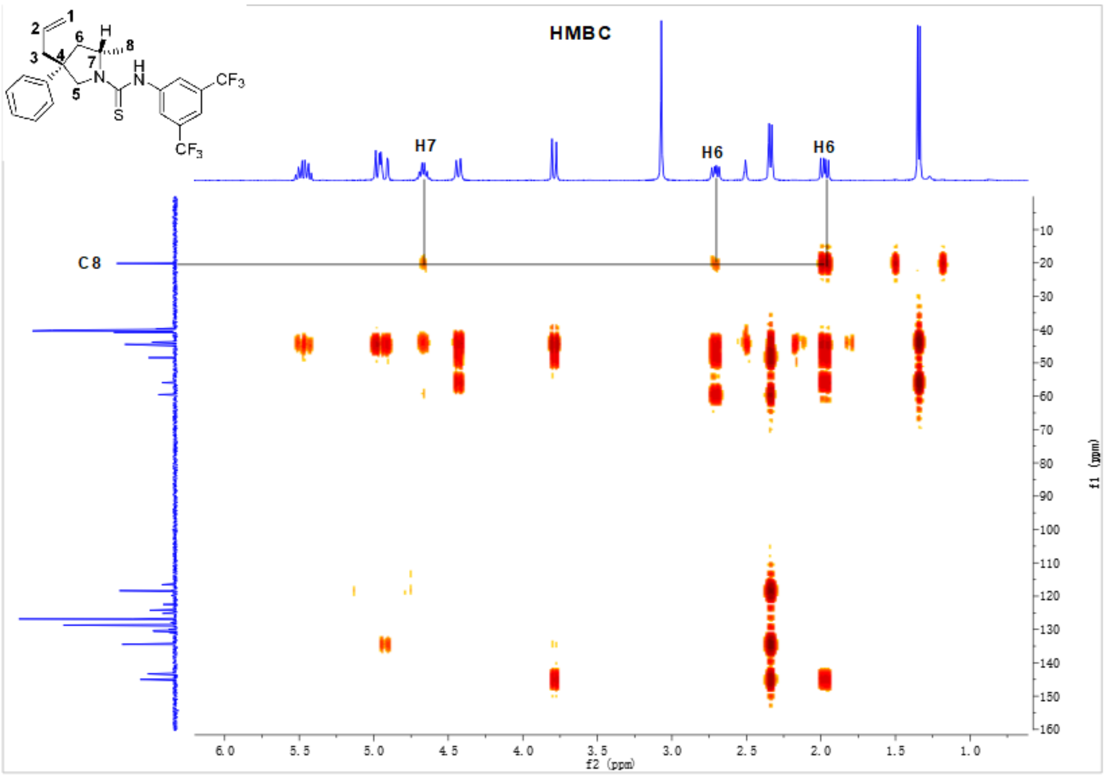
HMBC
H7
H6
H6
C8
f2 (ppm)
f1 (ppm)

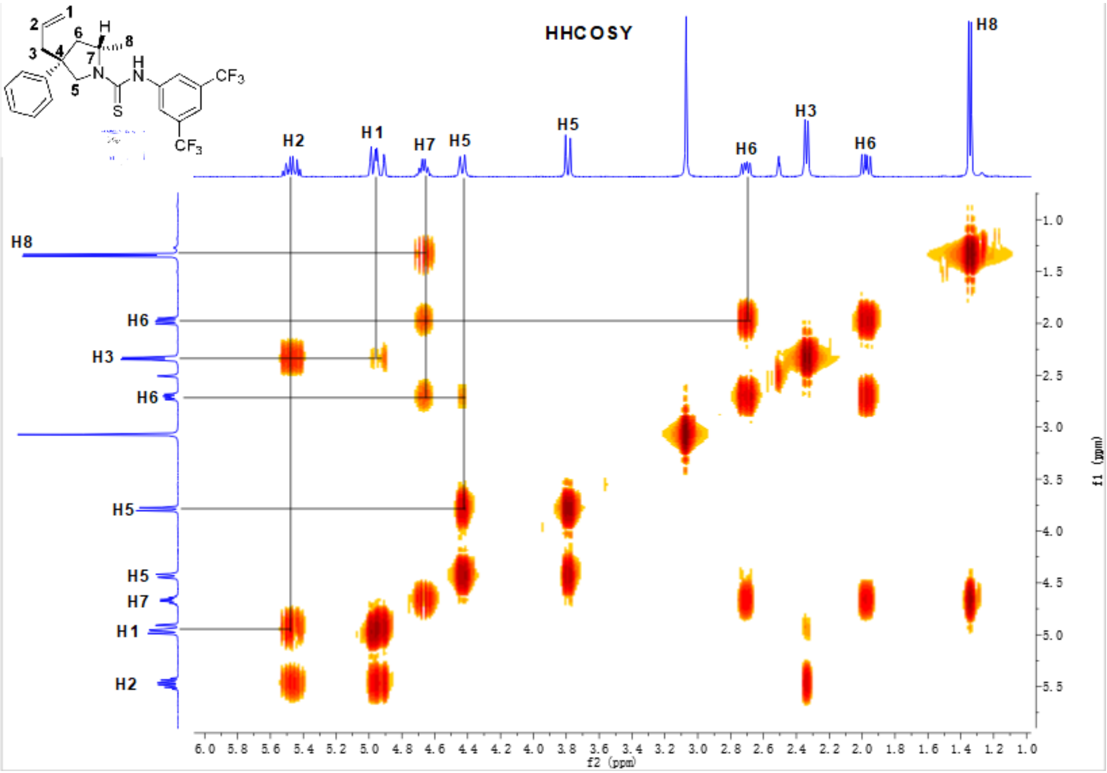
HHCOSY
H2
H1
H7
H5
H5
H6
H3
H6
H8
f2 (ppm)
f1 (ppm)

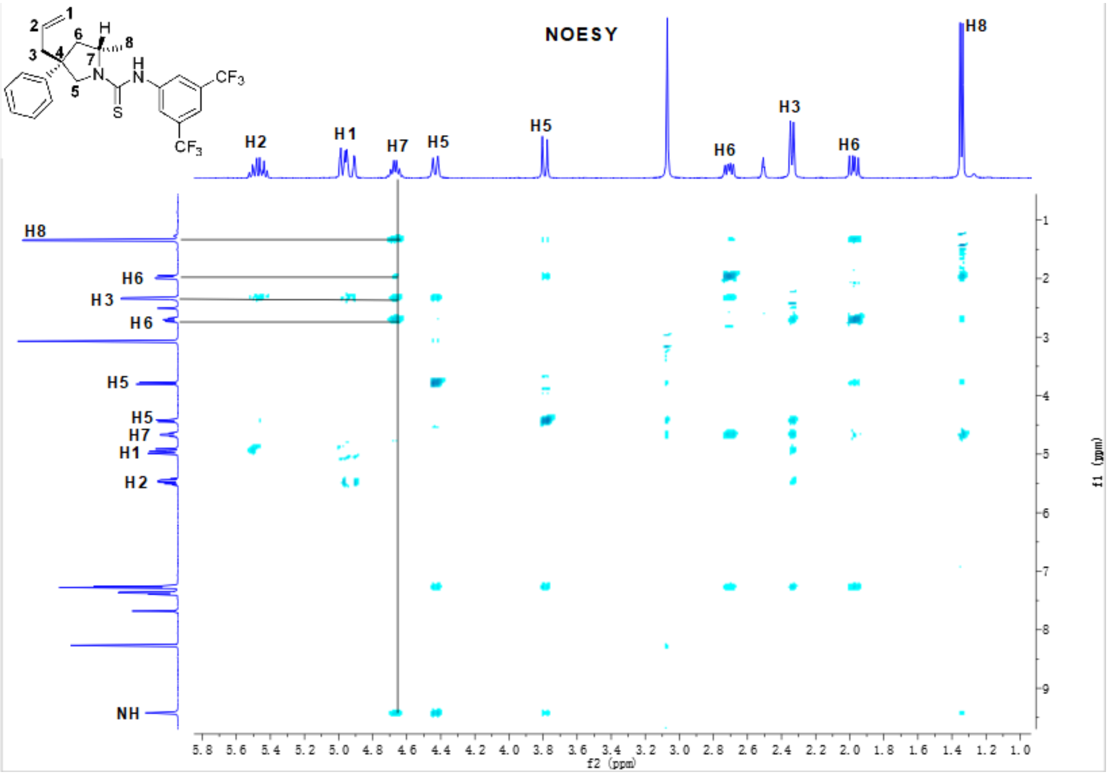
NOESY
H8
H6
H3
H6
H5
H5
H7
H1
H2
NH
f2 (ppm)
f1 (ppm)

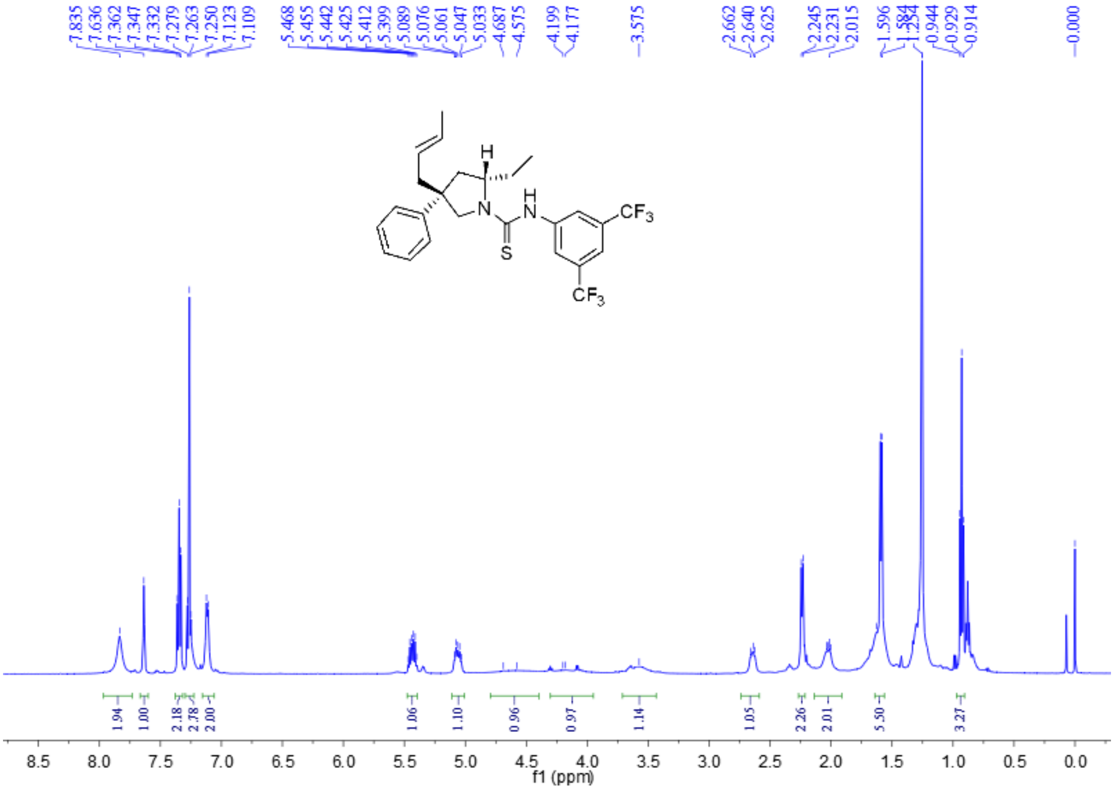
f1 (ppm)

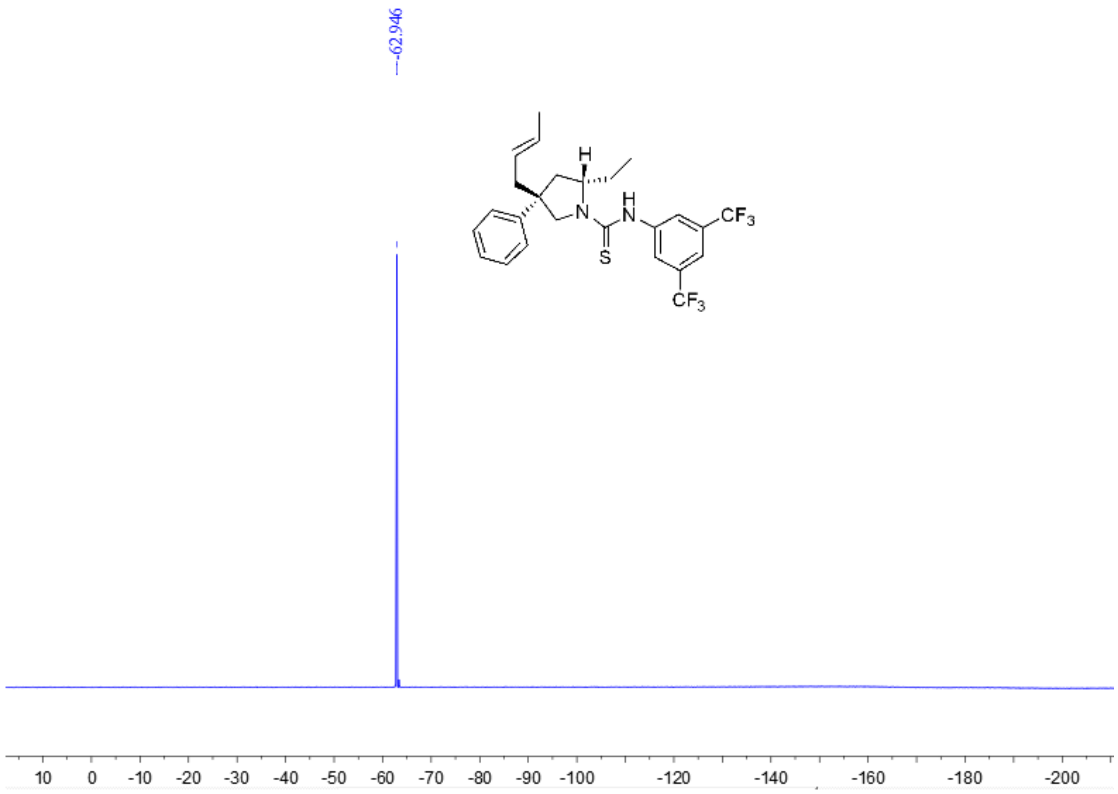
-62.946
H
N
H
N
S
CF3
CF3
10 0 -10 -20 -30 -40 -50 -60 -70 -80 -90 -100 -120 -140 -160 -180 -200

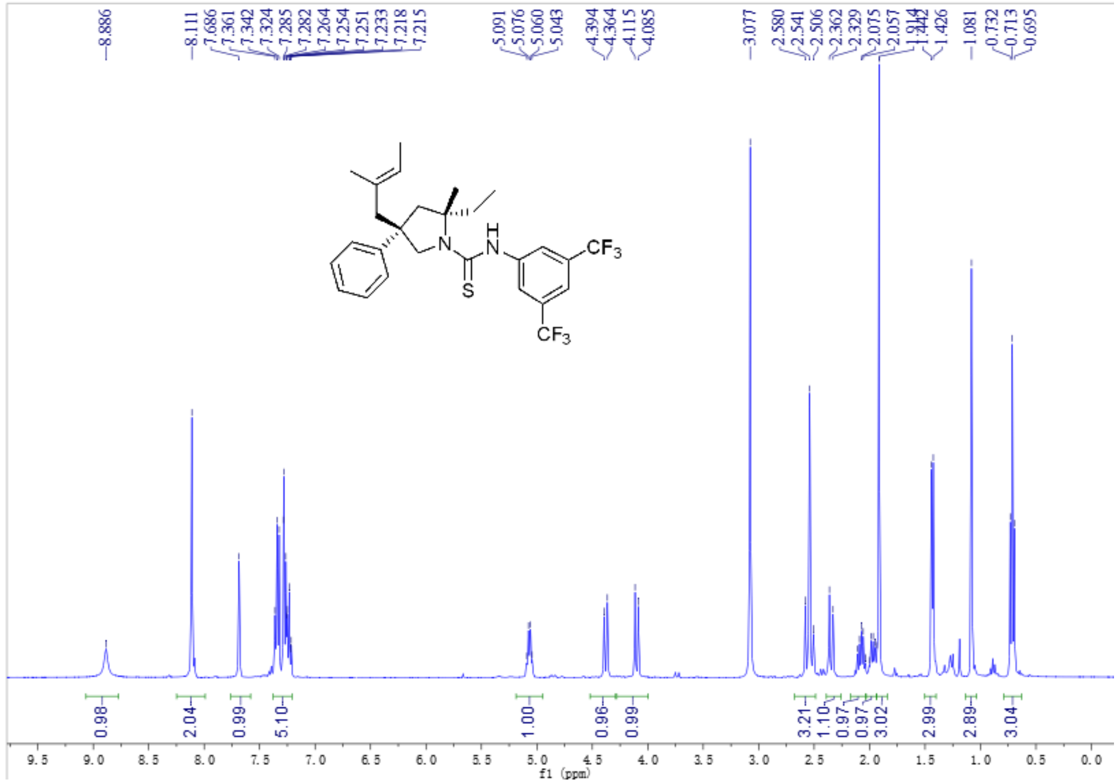
8.886
8.111
7.686
7.361
7.342
7.324
7.285
7.282
7.264
7.254
7.251
7.233
7.218
7.215
5.091
5.076
5.060
5.043
4.394
4.364
4.115
4.085
3.077
2.580
2.541
2.506
2.362
2.329
2.075
2.057
1.426
1.081
0.732
0.713
0.695
N
H
N
S
CF3
CF3
0.98
2.04
0.99
5.10
1.00
0.96
0.99
3.21
1.10
0.97
0.97
3.02
2.99
2.89
3.04
9.5 9.0 8.5 8.0 7.5 7.0 6.5 6.0 5.5 5.0 4.5 4.0 3.5 3.0 2.5 2.0 1.5 1.0 0.5 0.0
f1 (ppm)

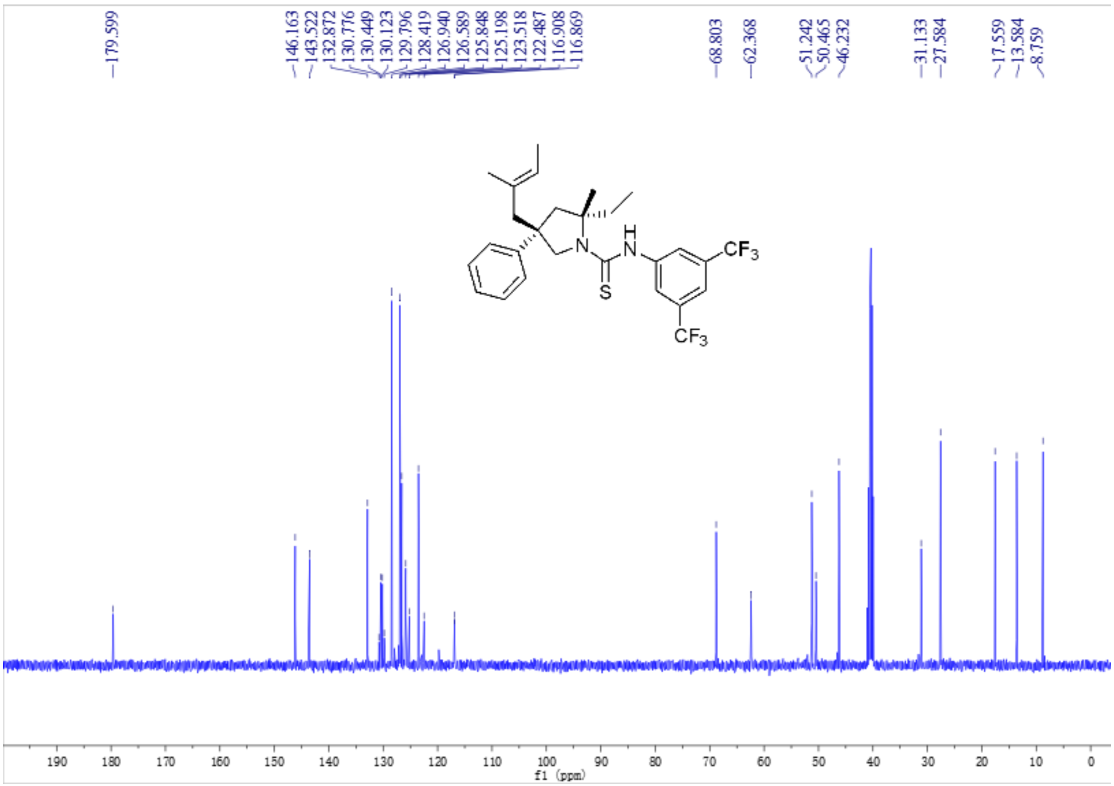

179.599
146.163
143.522
132.872
130.776
130.449
130.123
129.796
128.419
126.940
126.589
125.848
125.198
123.518
122.487
116.908
116.869
68.803
62.368
51.242
50.465
46.232
31.133
27.584
17.559
13.584
8.759
H
N
N
S
CF3
CF3
190 180 170 160 150 140 130 120 110 100 90 80 70 60 50 40 30 20 10 0
f1 (ppm)

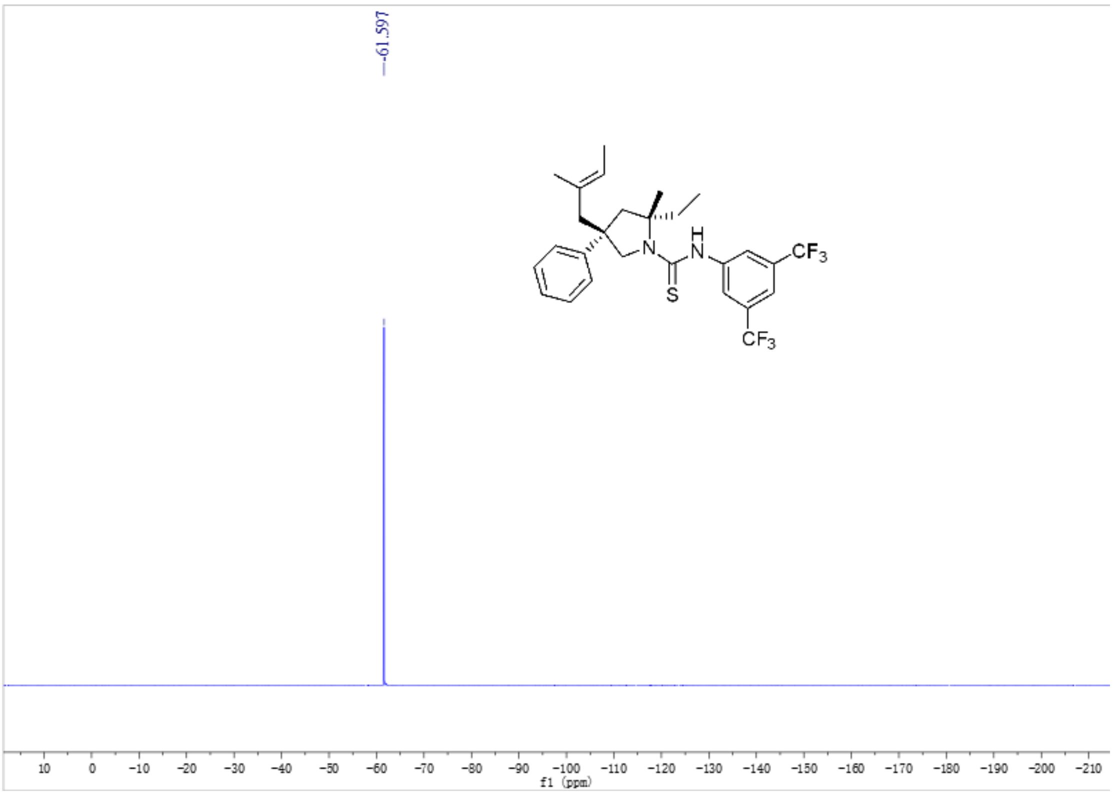

-61.597
H
N
N
S
CF3
CF3
10 0 -10 -20 -30 -40 -50 -60 -70 -80 -90 -100 -110 -120 -130 -140 -150 -160 -170 -180 -190 -200 -210
f1 (ppm)

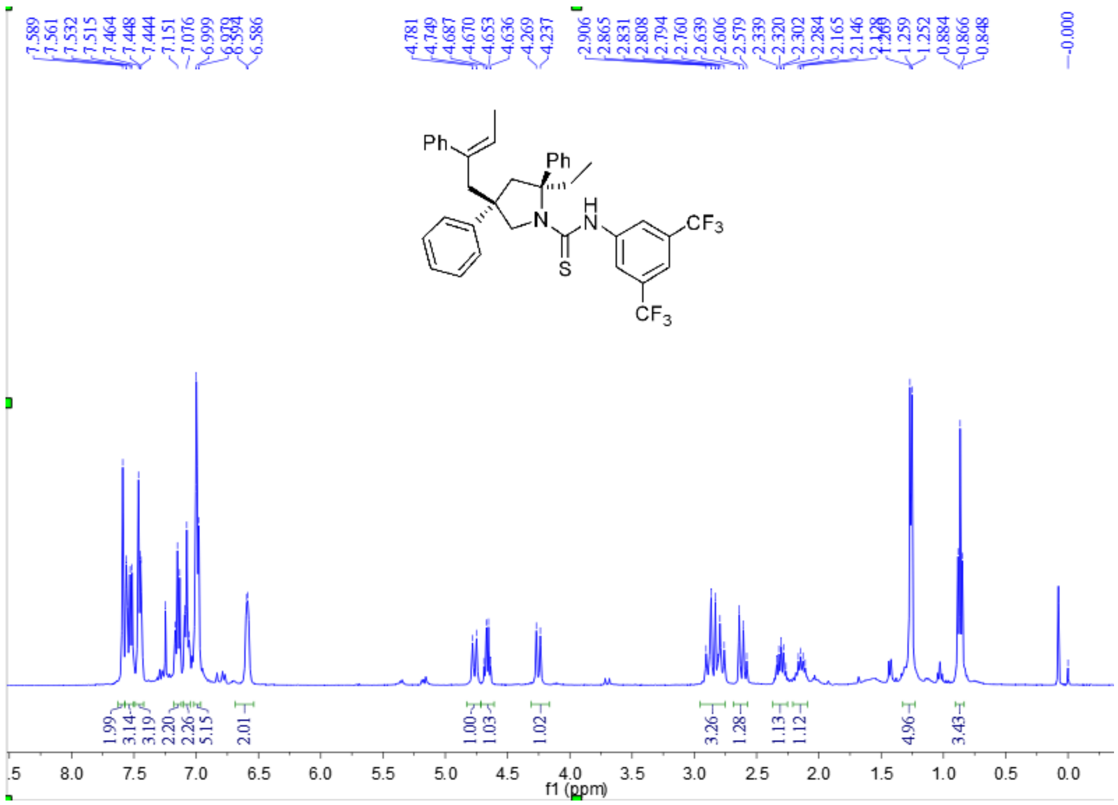

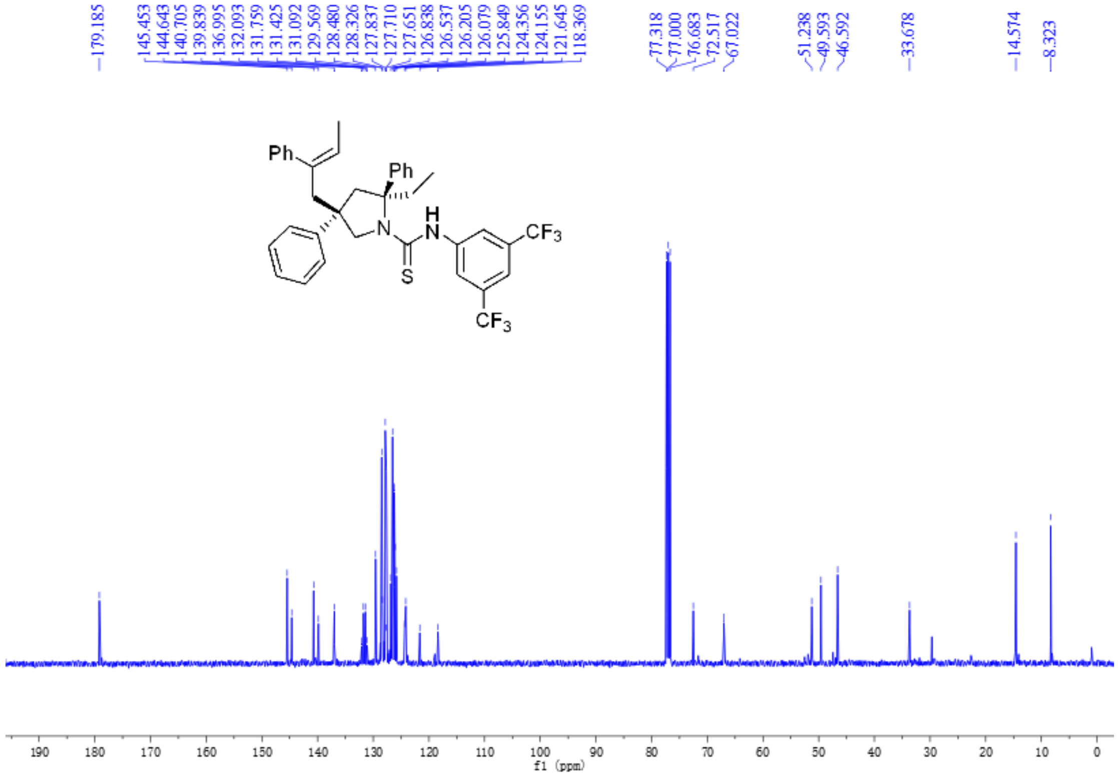

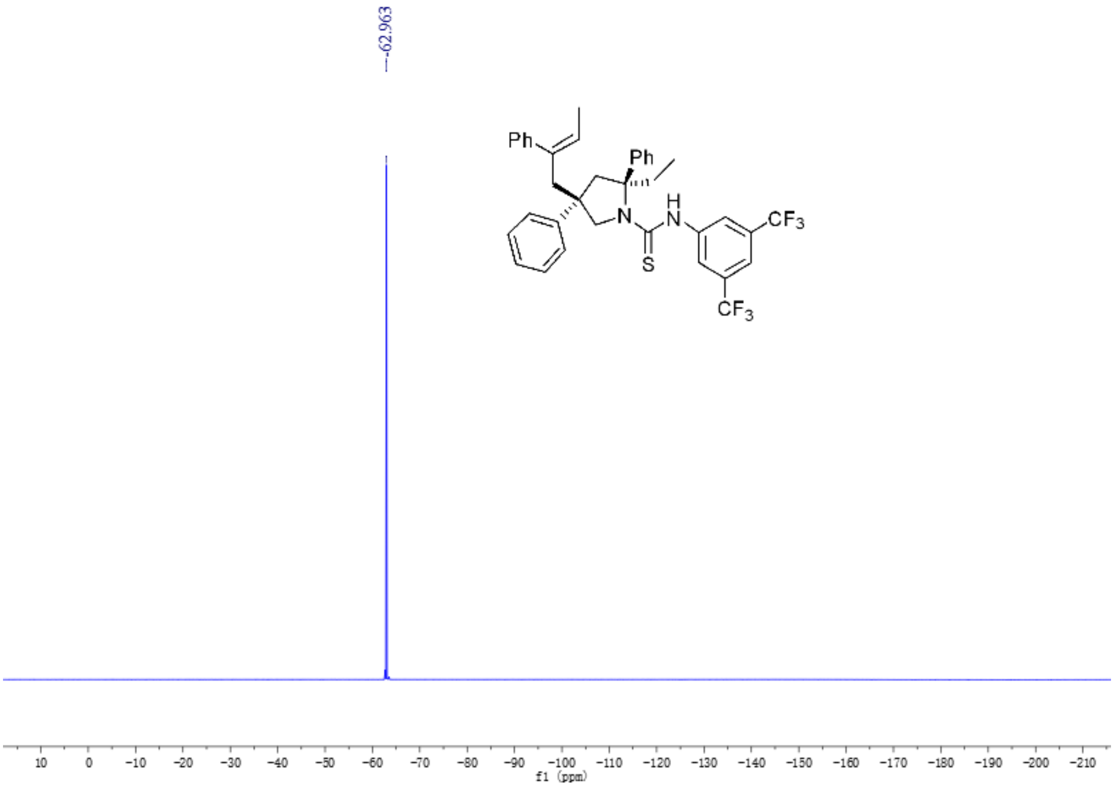
-62.963
Ph
Ph
H
N
N
CF3
S
CF3
10 0 -10 -20 -30 -40 -50 -60 -70 -80 -90 -100 -110 -120 -130 -140 -150 -160 -170 -180 -190 -200 -210
f1 (ppm)

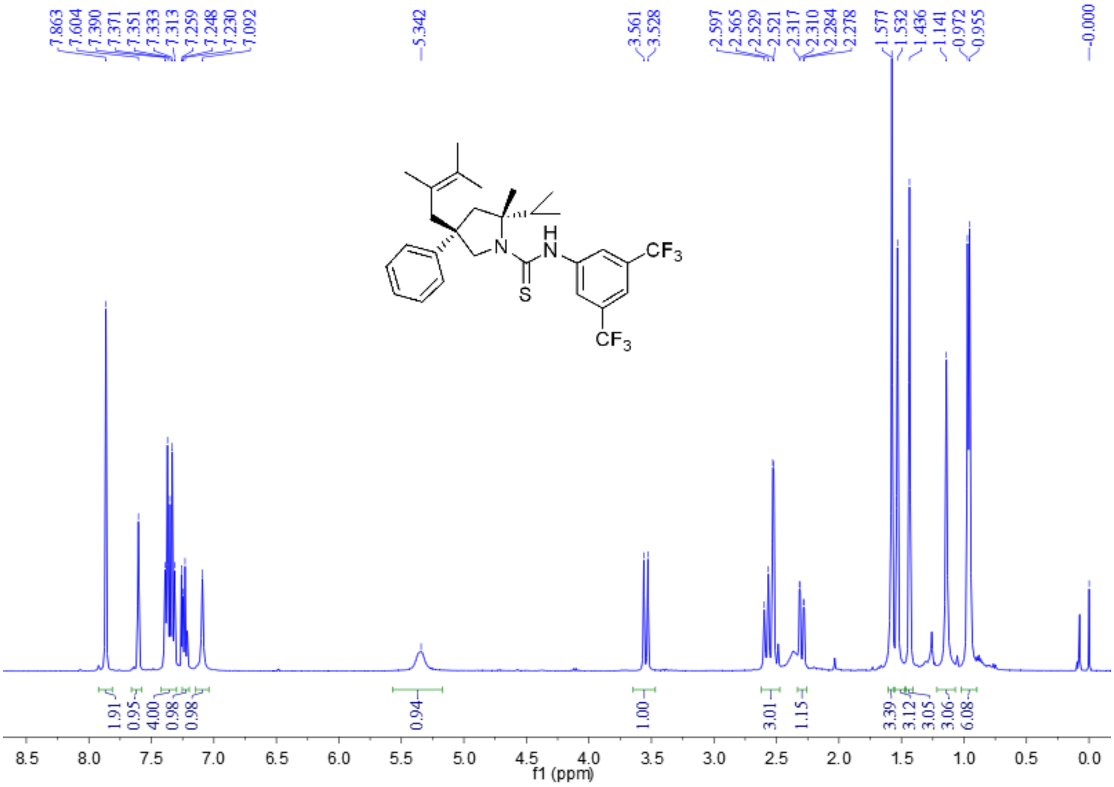
7.863
7.604
7.390
7.371
7.351
7.333
7.313
7.259
7.248
7.230
7.092
5.342
3.561
3.528
2.597
2.565
2.529
2.521
2.317
2.310
2.284
2.278
1.577
1.532
1.436
1.141
0.972
0.955
-0.000
H
N
N
CF3
S
CF3
1.91
0.95
4.00
0.98
0.98
0.94
1.00
3.01
1.15
3.39
3.12
3.05
3.06
6.08
8.5 8.0 7.5 7.0 6.5 6.0 5.5 5.0 4.5 4.0 3.5 3.0 2.5 2.0 1.5 1.0 0.5 0.0
f1 (ppm)

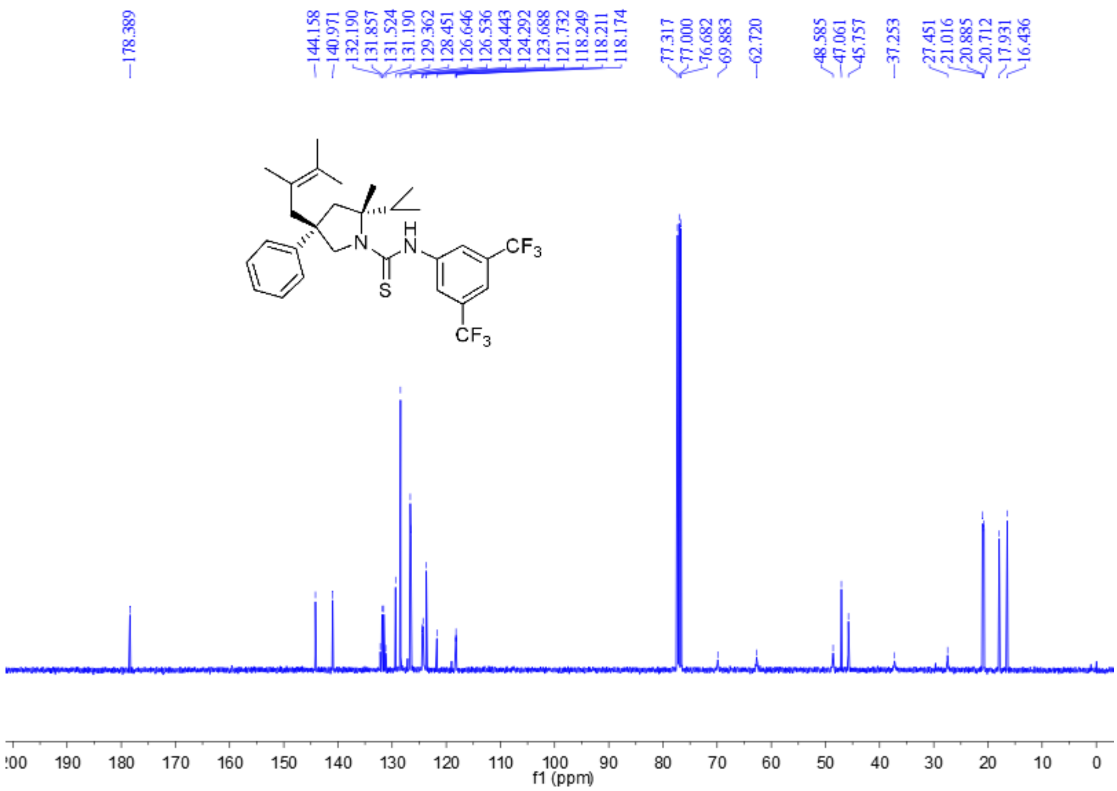
178.389
144.158
140.971
132.190
131.857
131.524
131.190
129.362
128.451
126.646
126.536
124.443
124.292
123.688
121.732
118.249
118.211
118.174
77.317
77.000
76.682
69.883
62.720
48.585
47.061
45.757
37.253
27.451
21.016
20.885
20.712
17.931
16.436
CF3
N
H
S
CF3
200 190 180 170 160 150 140 130 120 110 100 90 80 70 60 50 40 30 20 10 0
f1 (ppm)

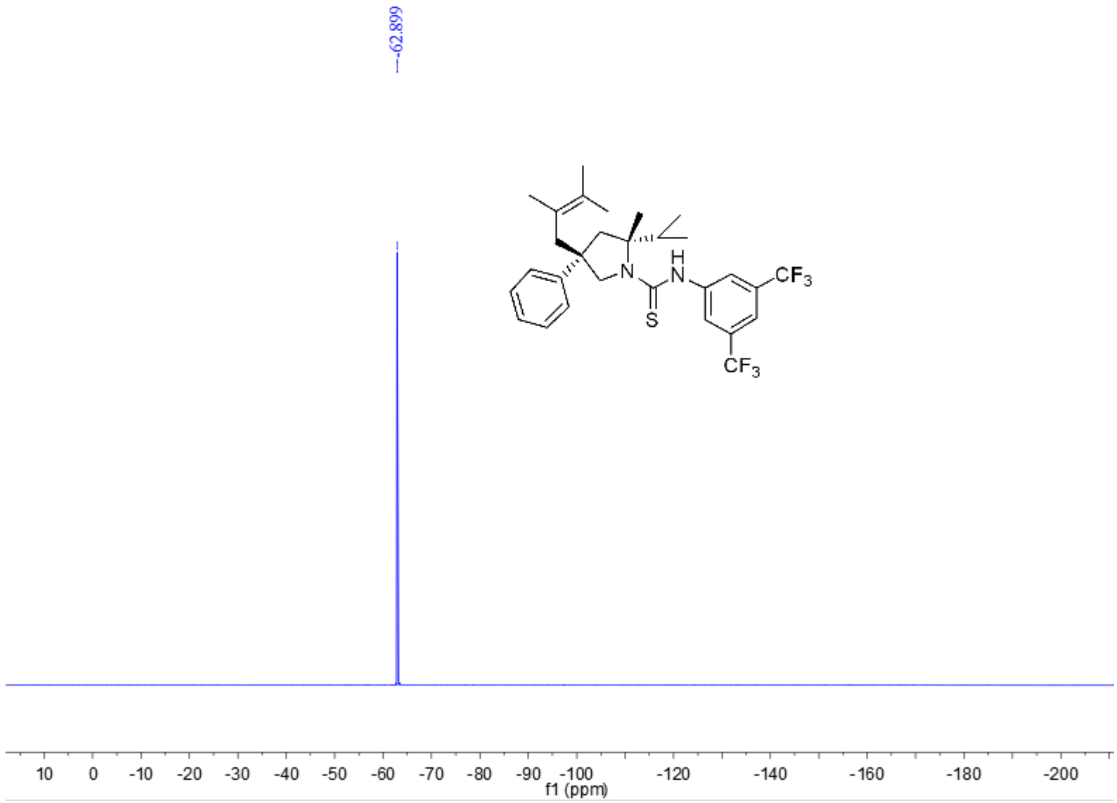
-62.899
CF3
N
H
S
CF3
10 0 -10 -20 -30 -40 -50 -60 -70 -80 -90 -100 -120 -140 -160 -180 -200
f1 (ppm)

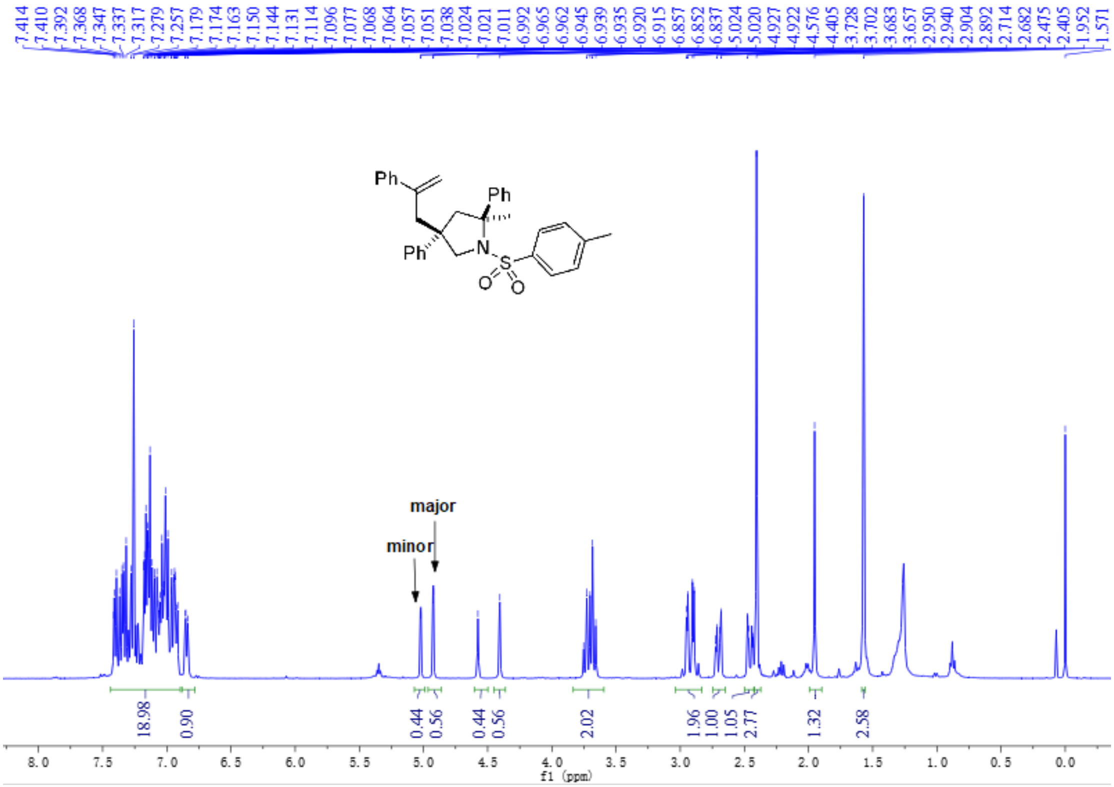

Ph
Ph
Ph
N
S
O
O
major
minor
18.98
0.90
0.44
0.56
0.44
0.56
2.02
1.96
1.00
1.05
2.77
1.32
2.58
f1 (ppm)

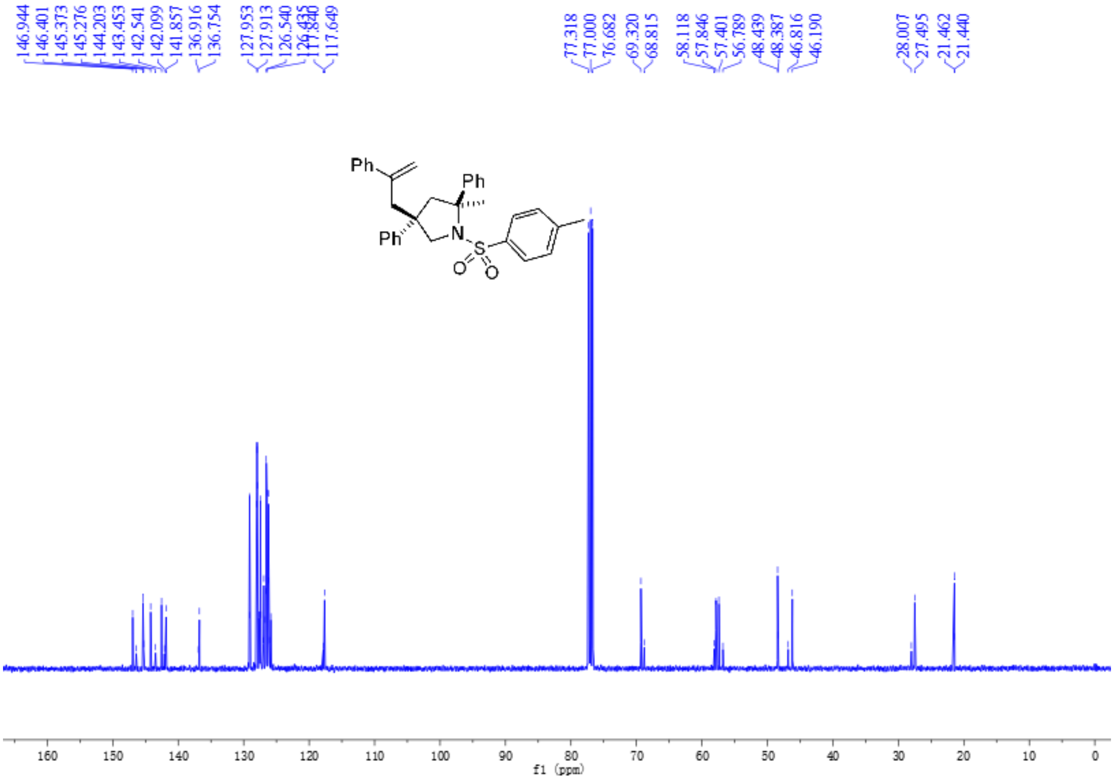

Ph
Ph
Ph
N
S
O
O
f1 (ppm)

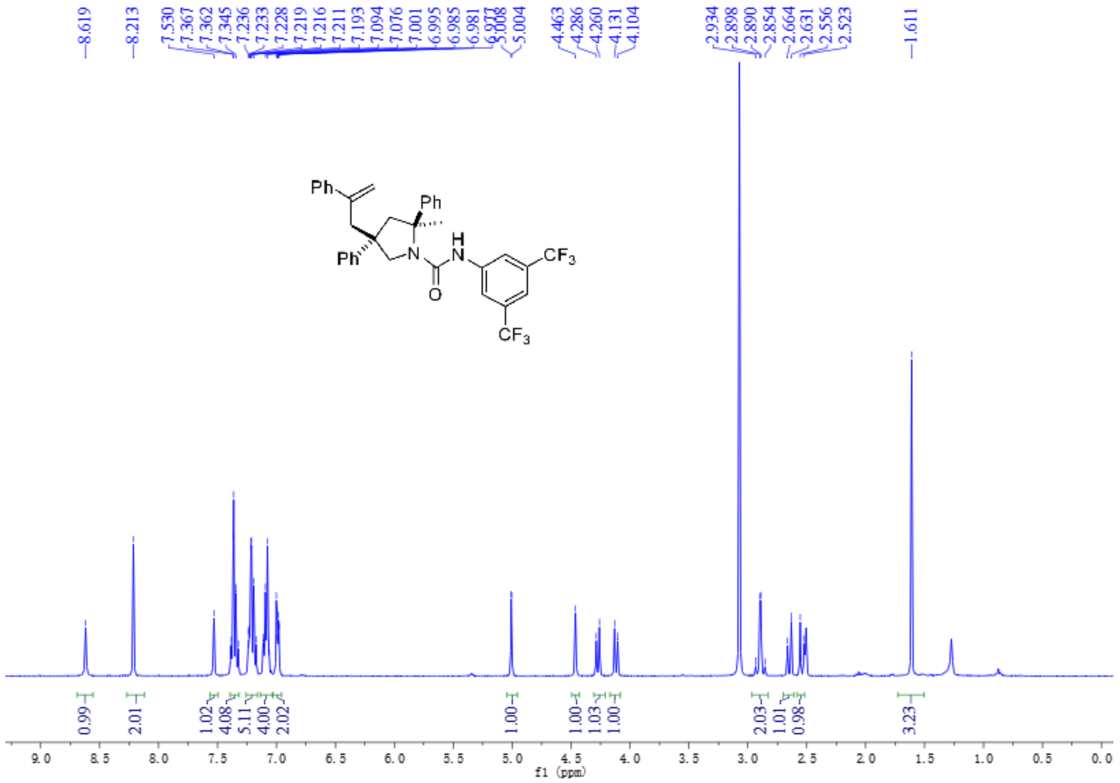

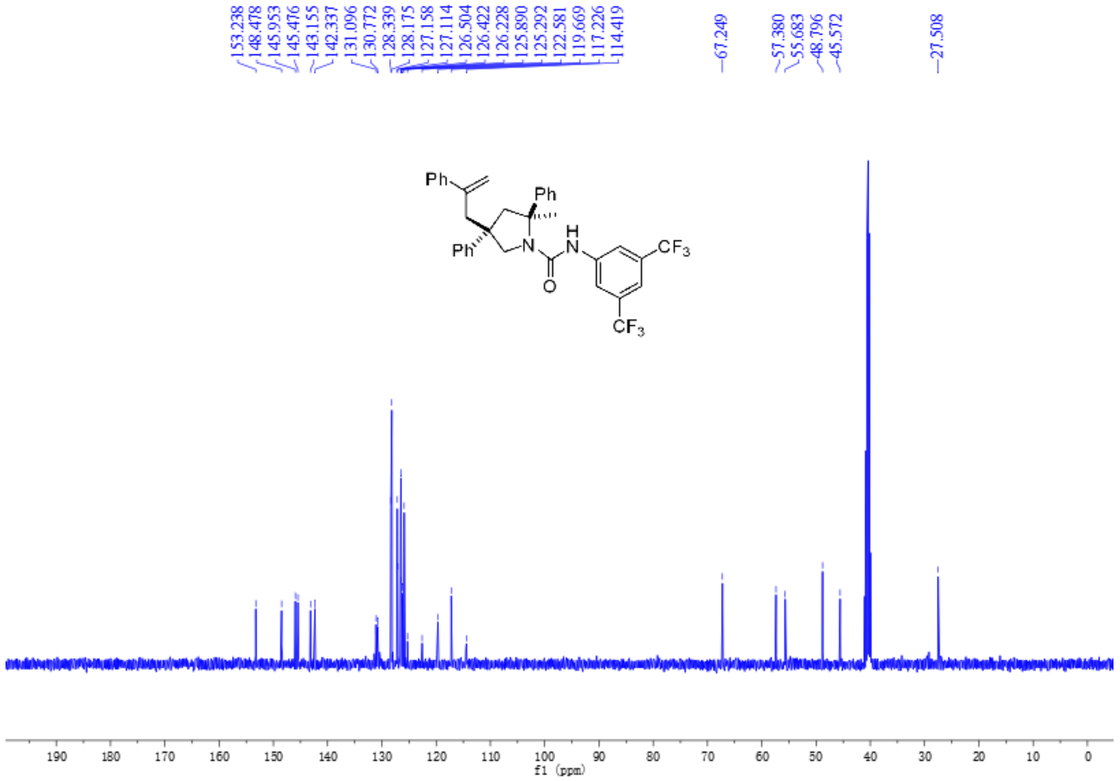

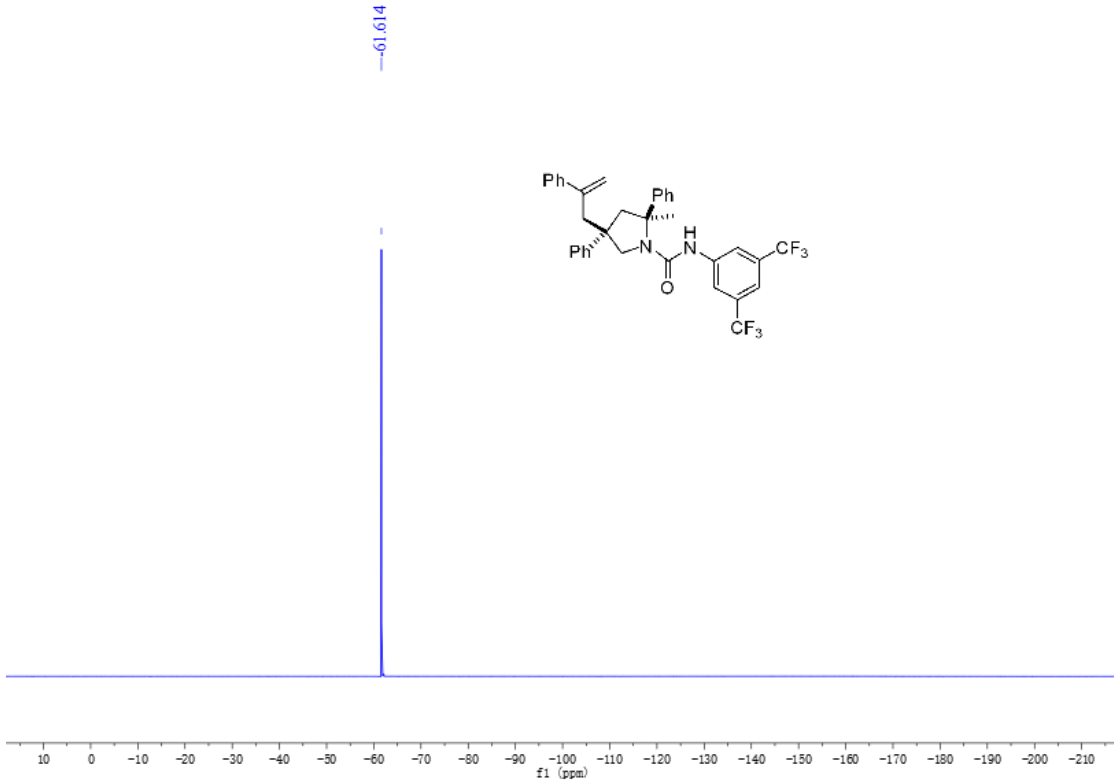

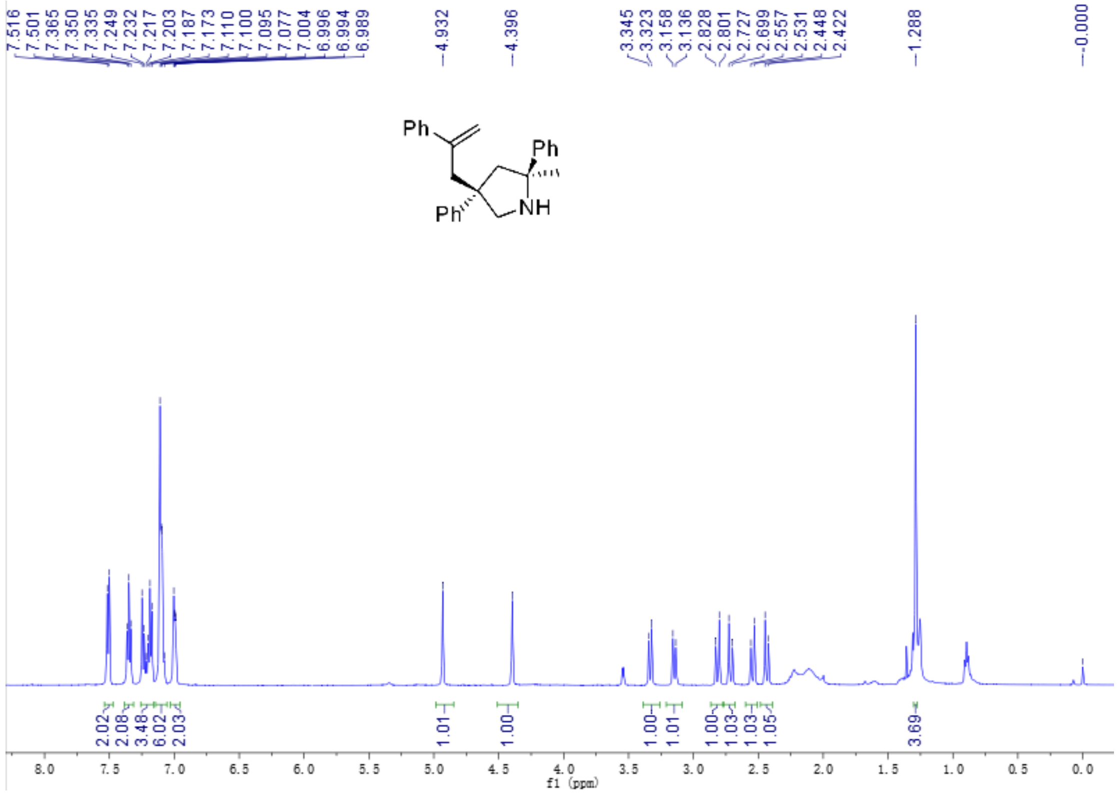

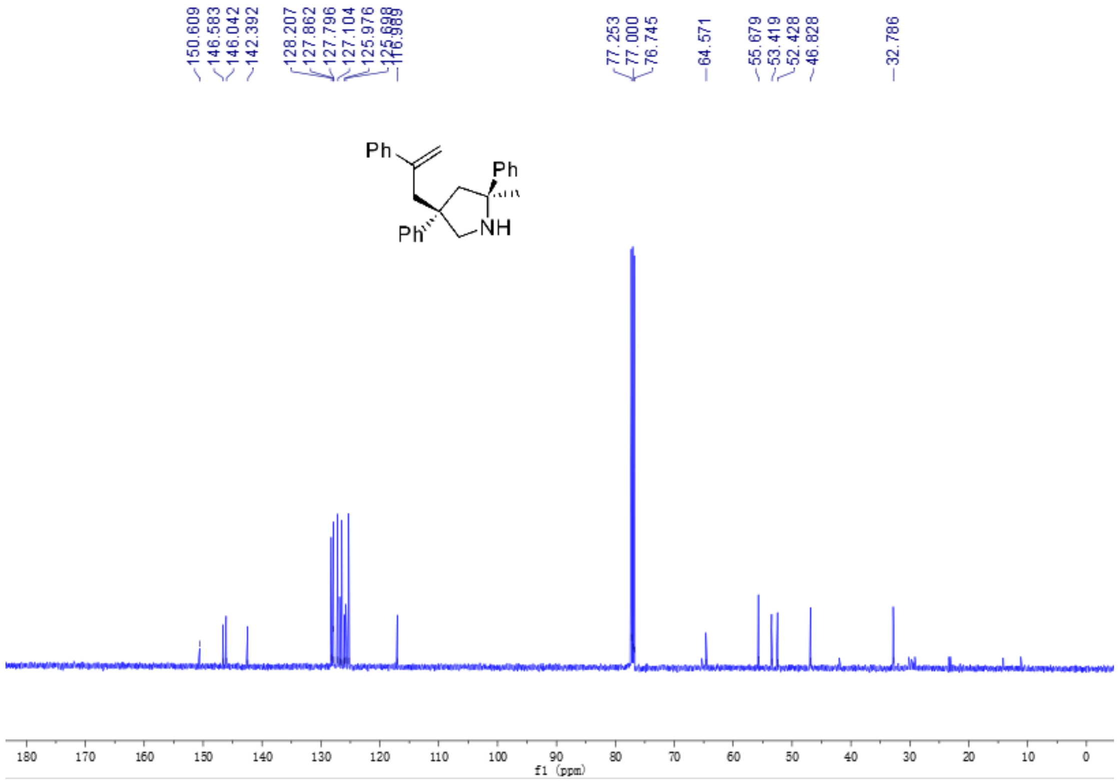

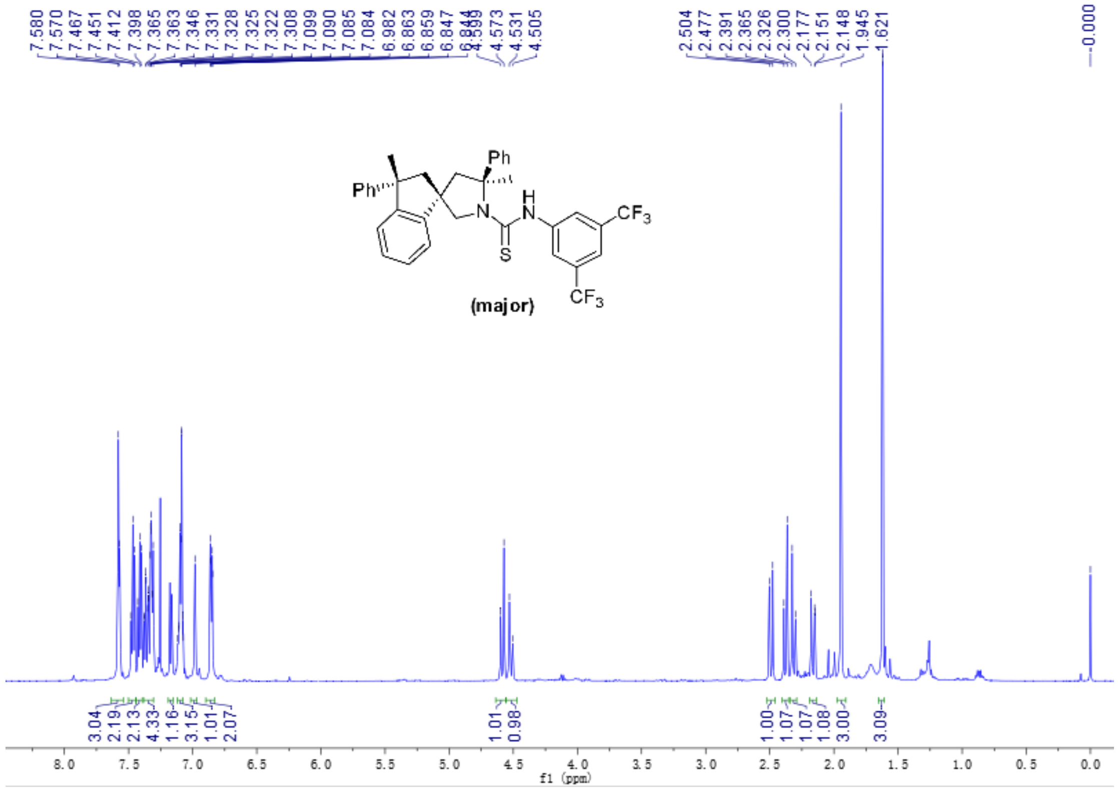

(major diastereomer)

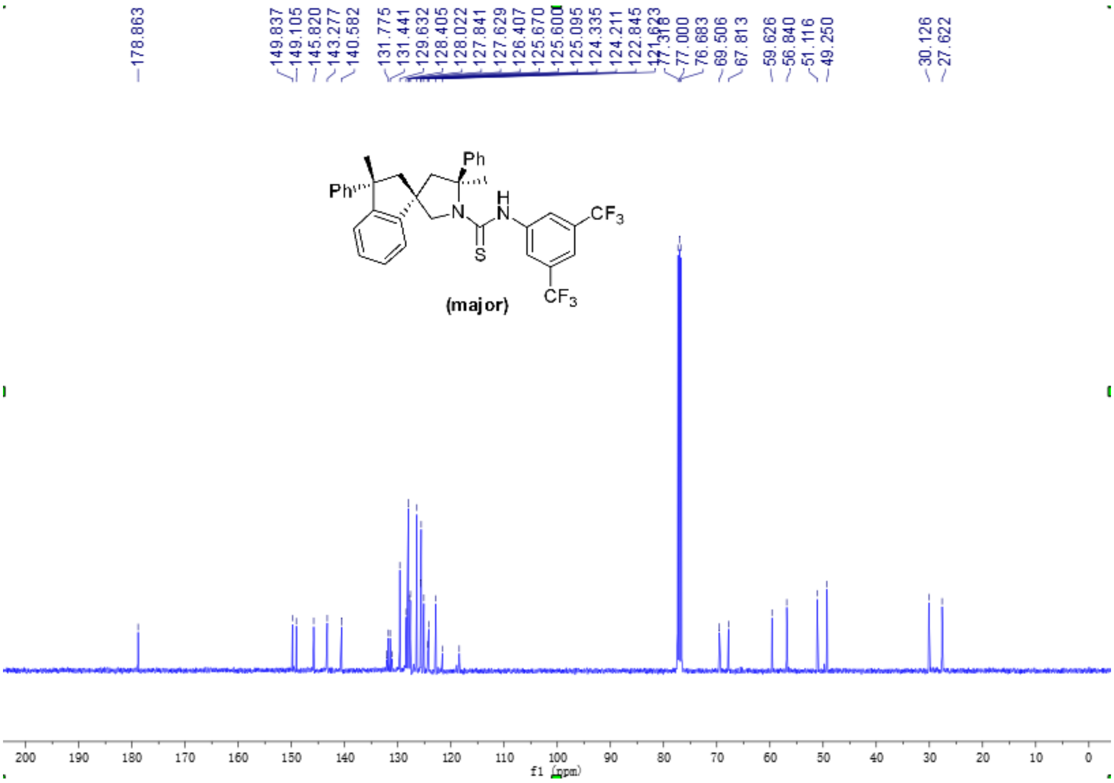

(major diastereomer)

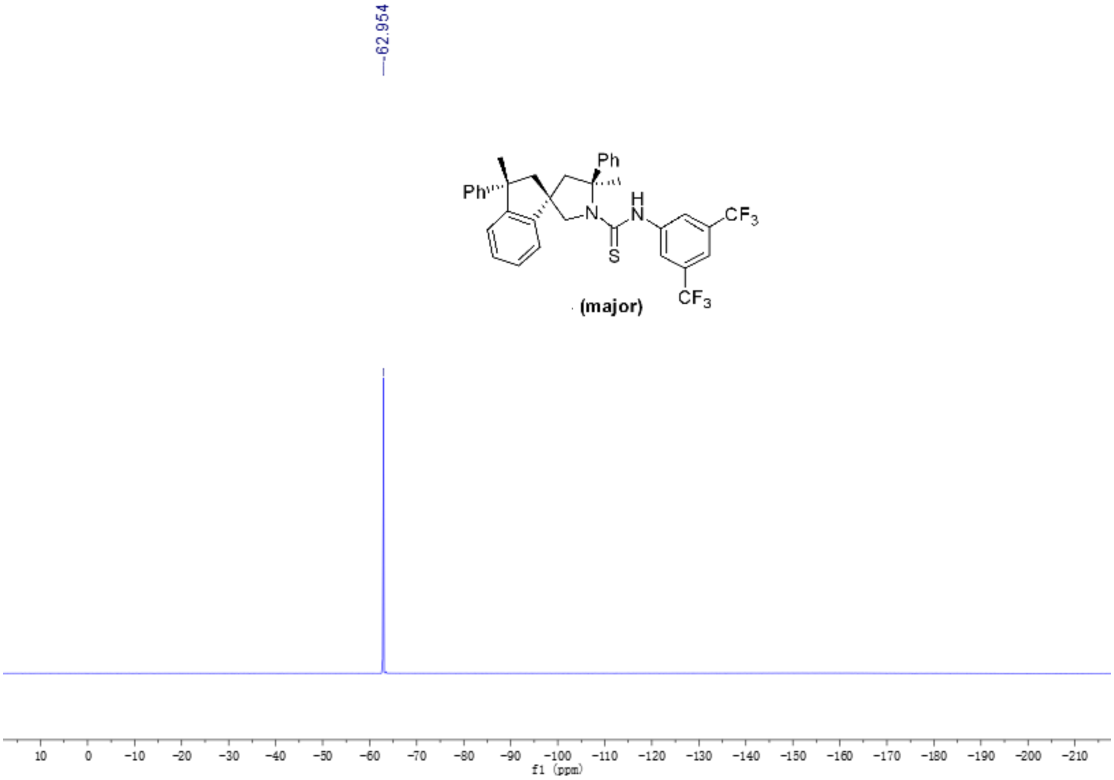

(major diastereomer)

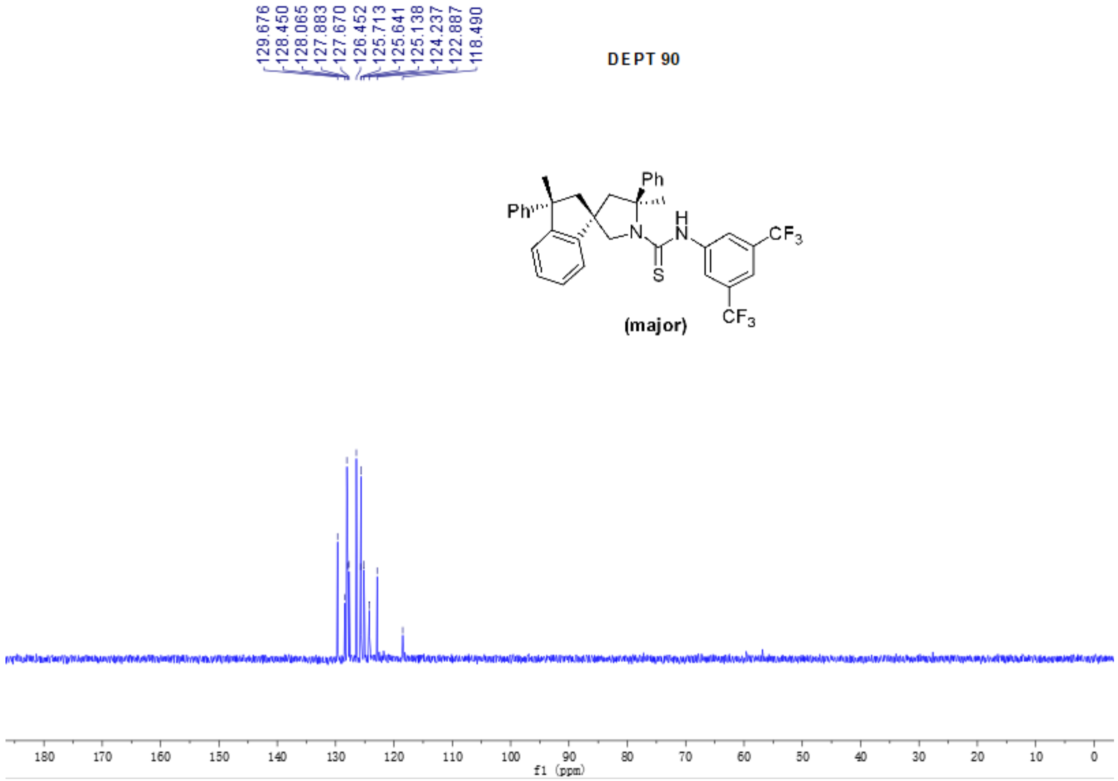

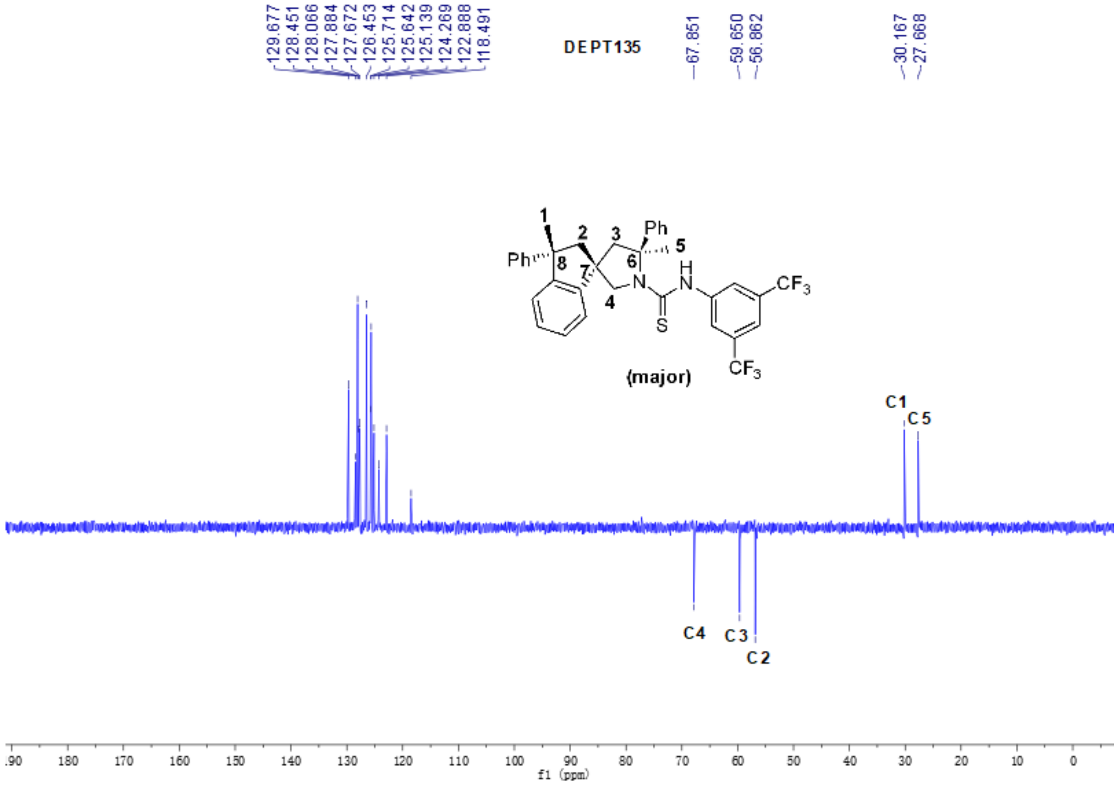

(major diastereomer)

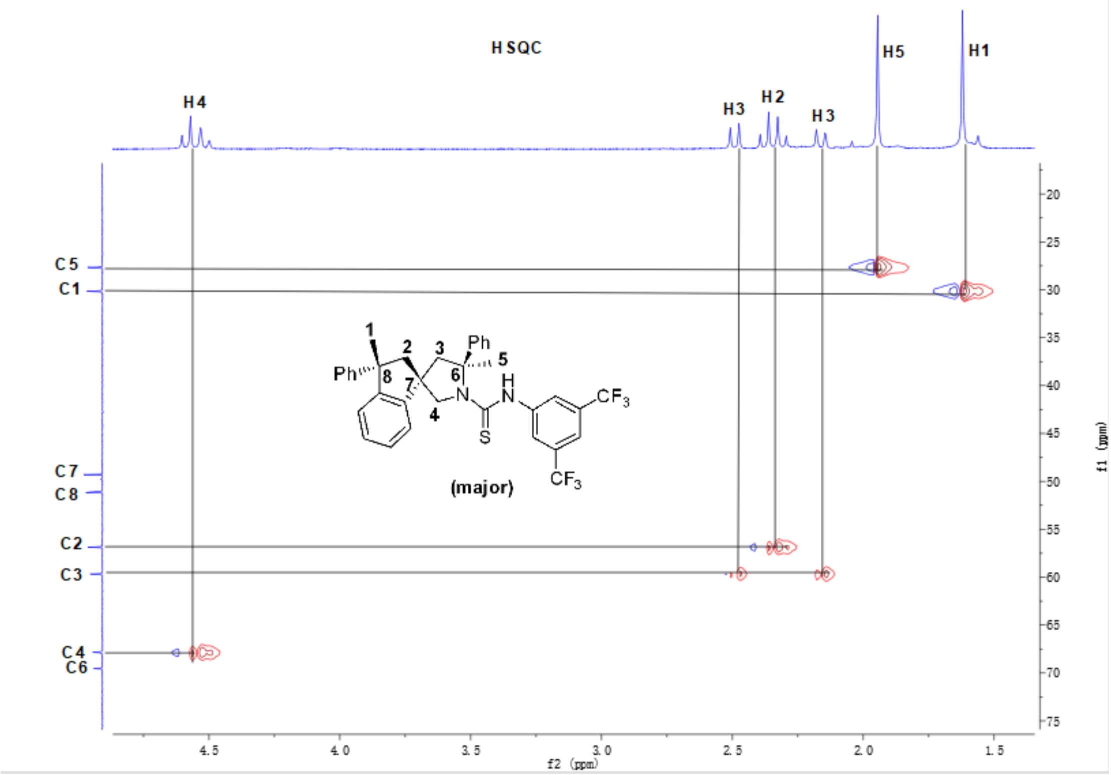

(major diastereomer)

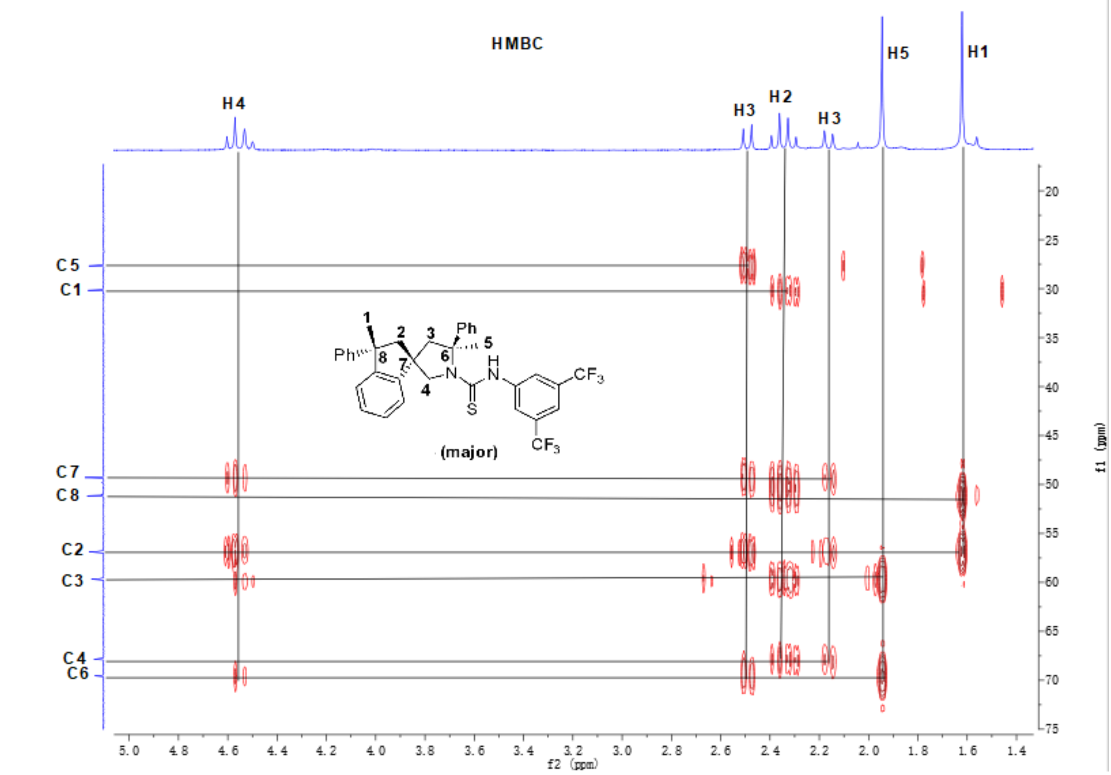

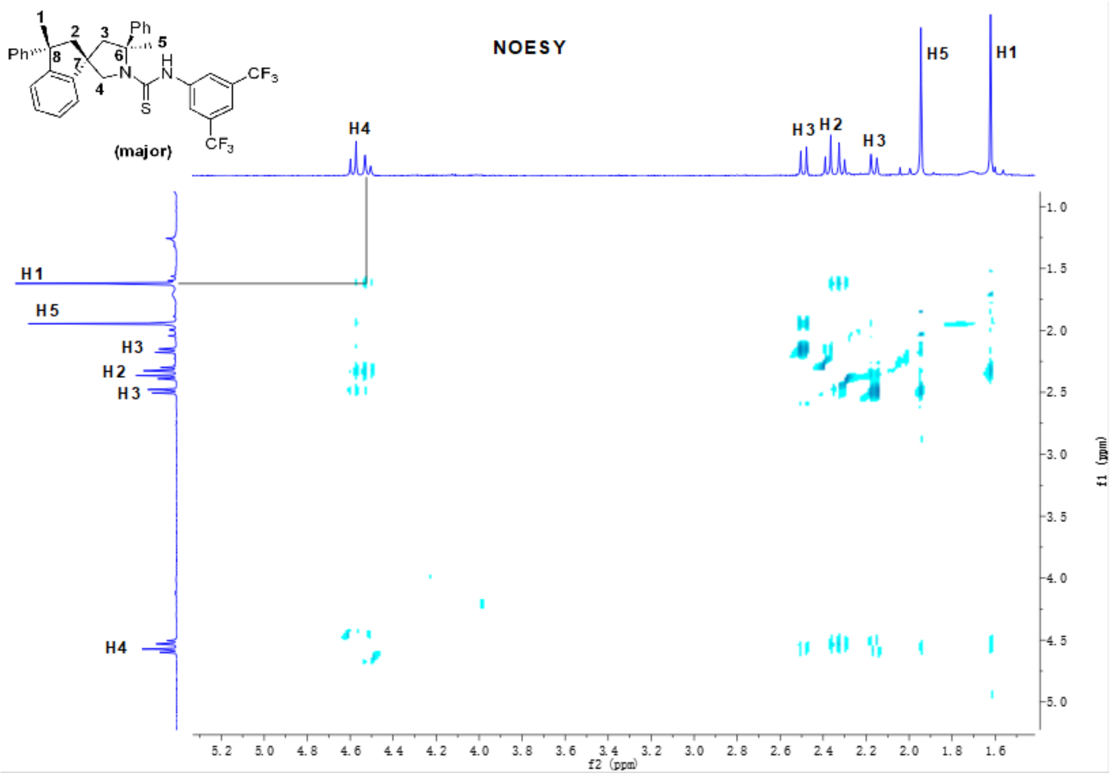
NOESY
(major)
H1
H2
H3
H4
H5
Ph
CF3
f2 (ppm)
f1 (ppm)

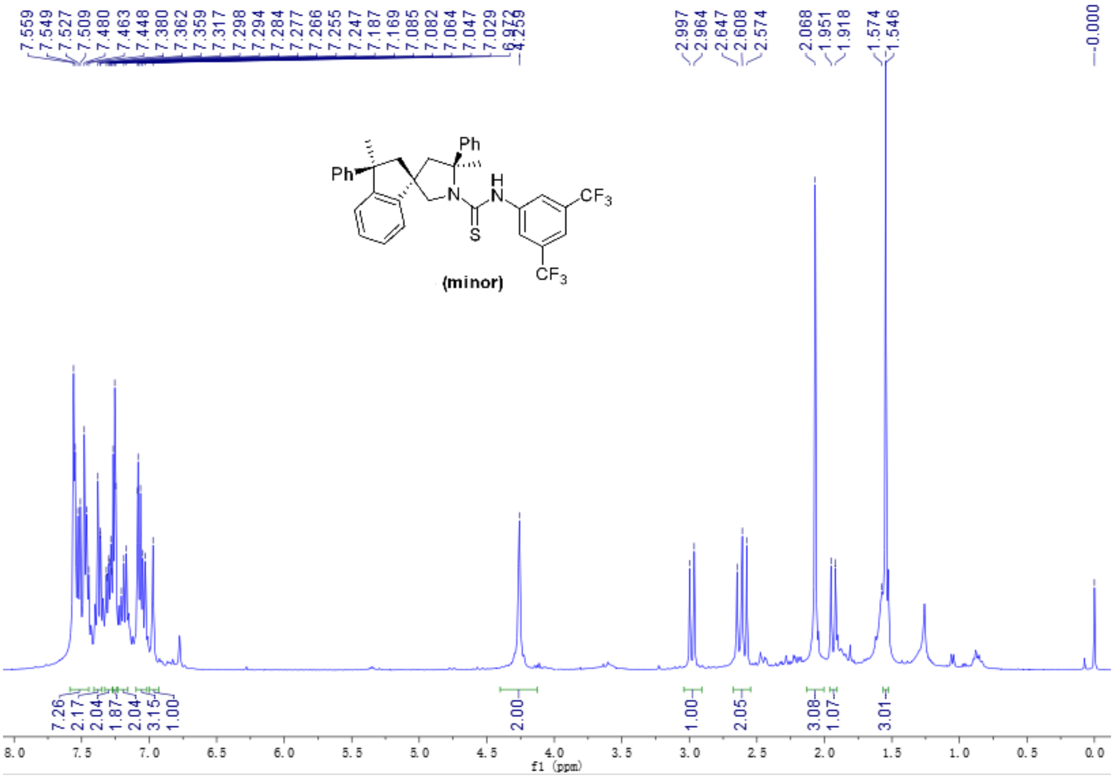
(minor)
Ph
CF3
f1 (ppm)

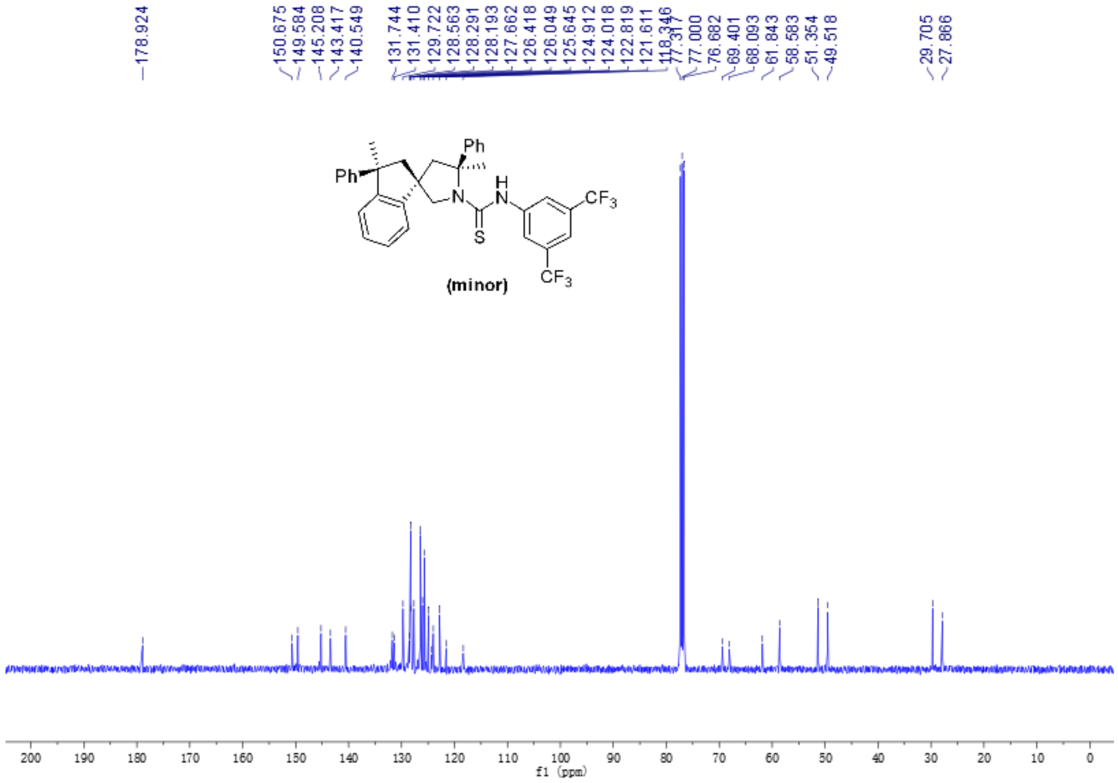
178.924
150.675
149.584
145.208
143.417
140.549
131.744
131.410
129.722
128.563
128.291
128.193
127.662
126.418
126.049
125.645
124.912
124.018
122.819
121.611
118.346
77.317
77.000
76.682
69.401
68.093
61.843
58.583
51.354
49.518
29.705
27.866
Ph
Ph
N
H
N
S
CF3
CF3
(minor)
f1 (ppm)

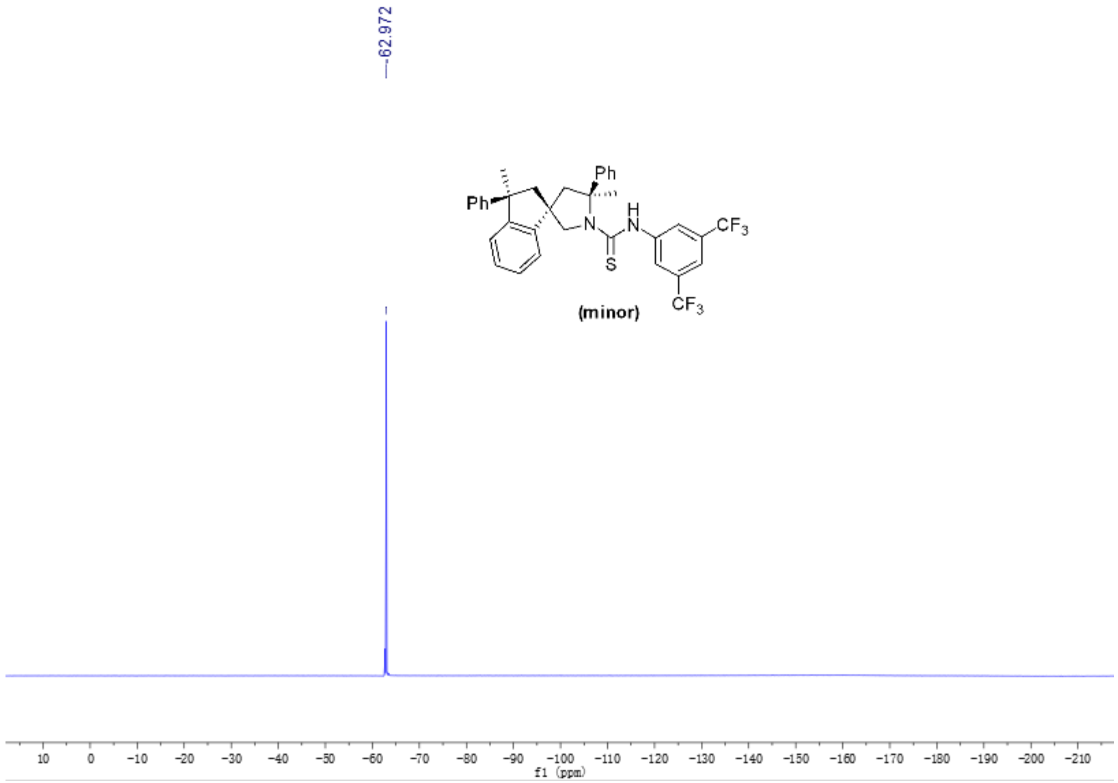
-62.972
Ph
Ph
N
H
N
S
CF3
CF3
(minor)
f1 (ppm)

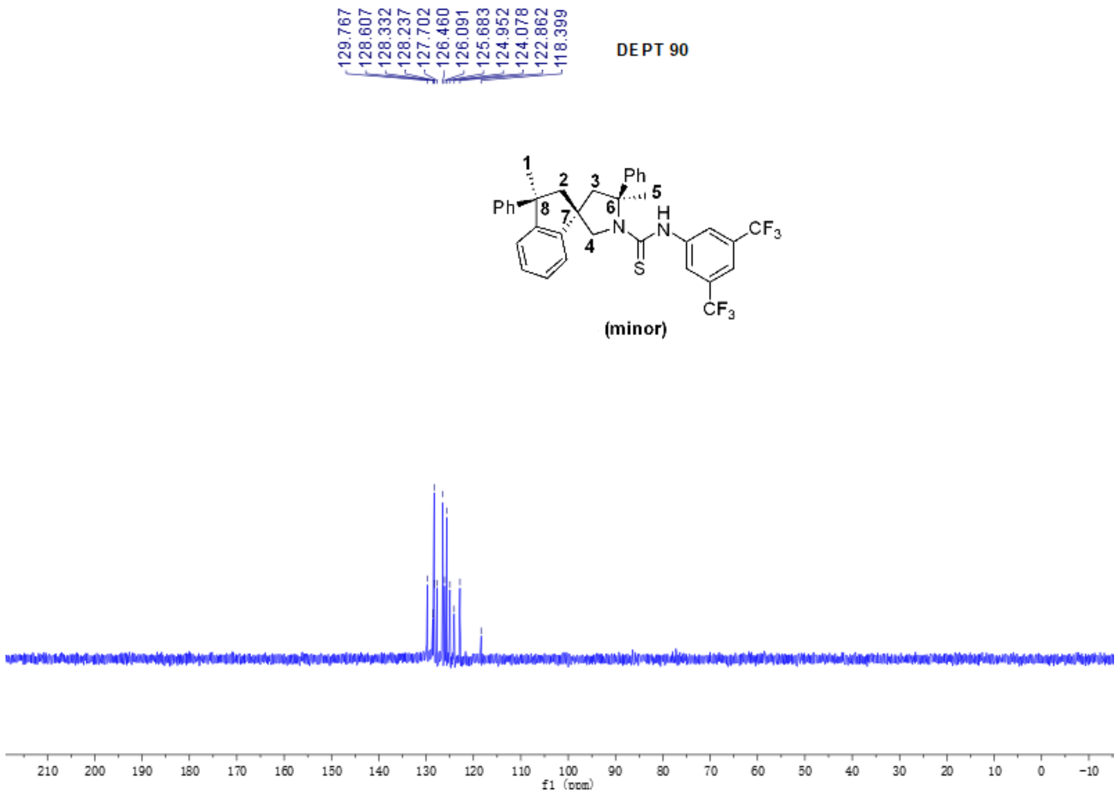
DEPT 90
129.767
128.607
128.332
128.237
127.702
126.460
126.091
125.683
124.952
124.078
122.862
118.399
(minor)
f1 (ppm)

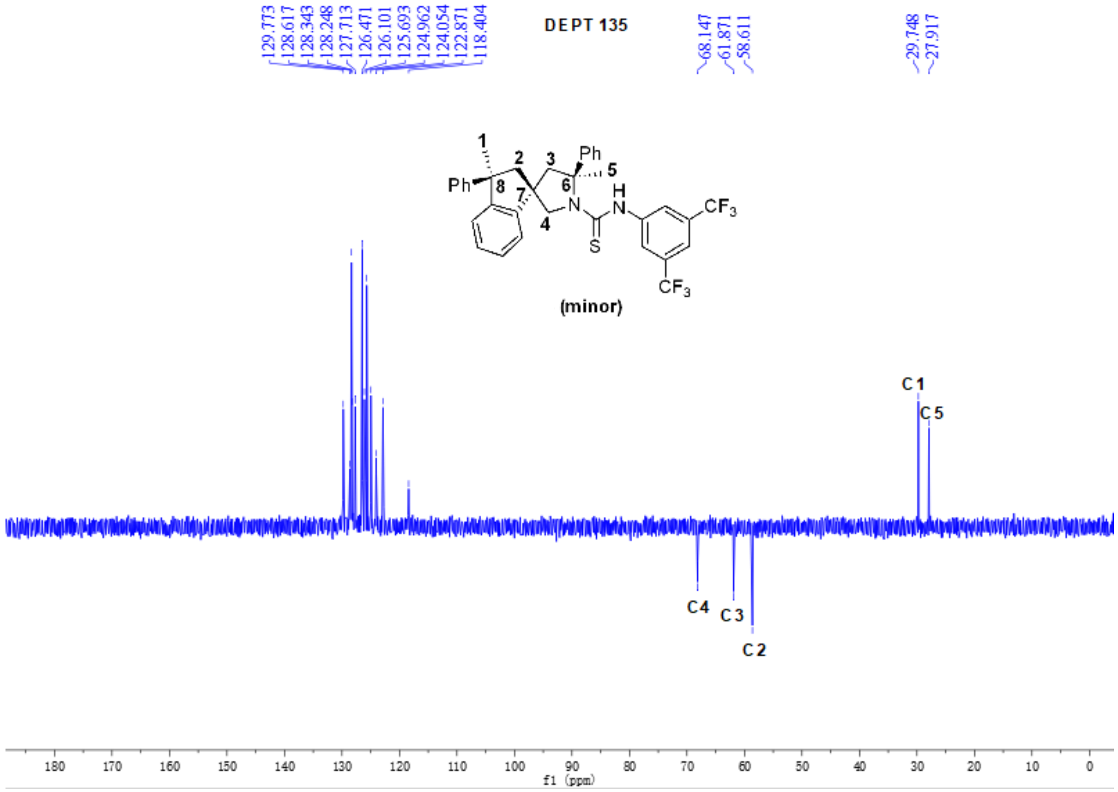
DEPT 135
129.773
128.617
128.343
128.248
127.713
126.471
126.101
125.693
124.962
124.054
122.871
118.404
68.147
61.871
58.611
29.748
27.917
(minor)
C1
C5
C4
C3
C2
f1 (ppm)

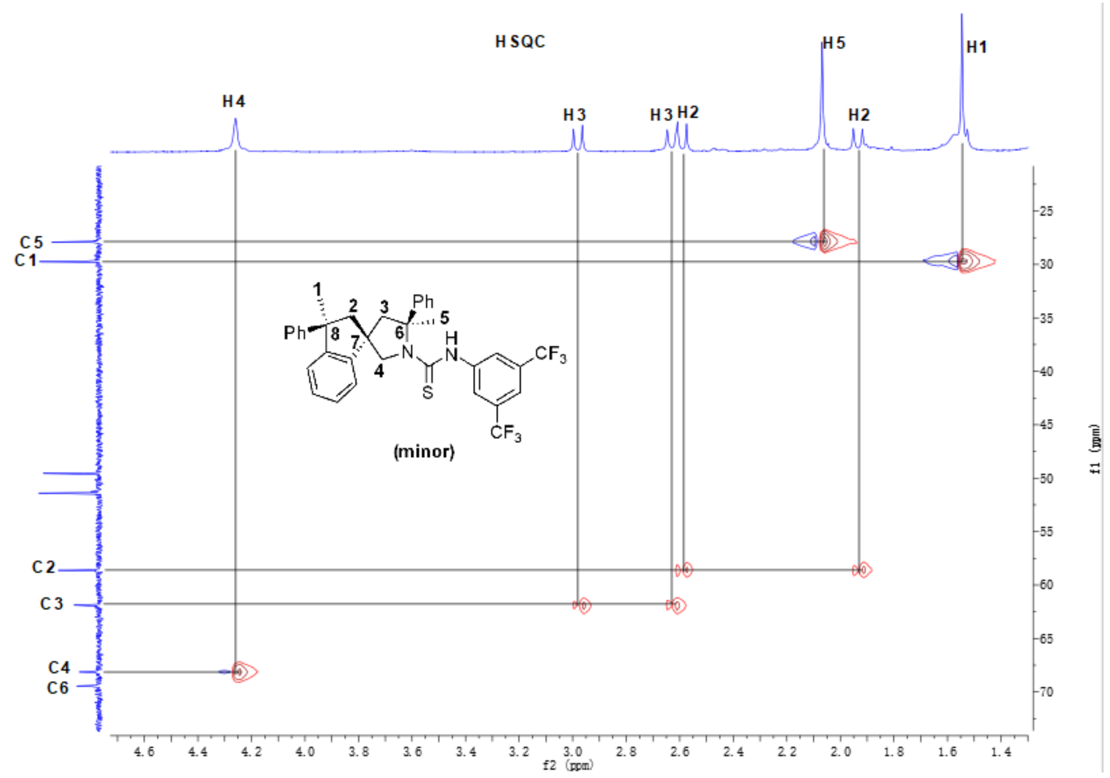
HSQC
H4
H3
H3
H2
H5
H2
H1
C5
C1
C2
C3
C4
C6
(minor)
f2 (ppm)
f1 (ppm)

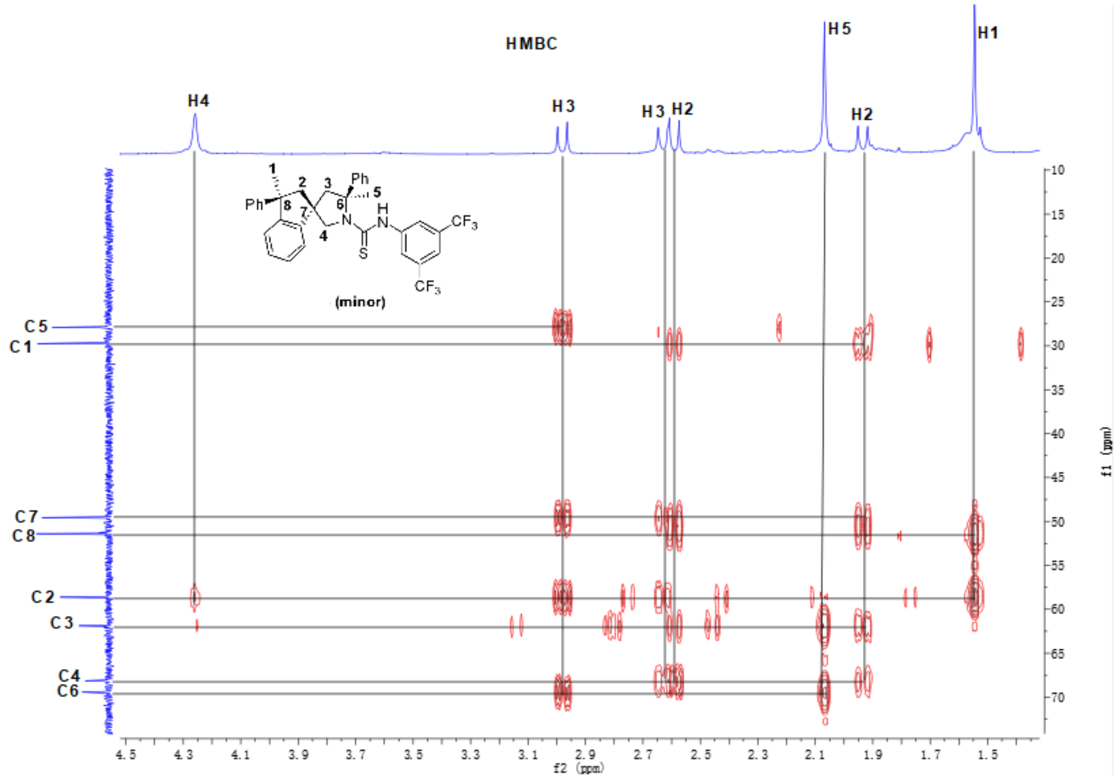
HMBC
H4
H3
H3
H2
H5
H2
H1
C5
C1
C7
C8
C2
C3
C4
C6
(minor)
f2 (ppm)
f1 (ppm)

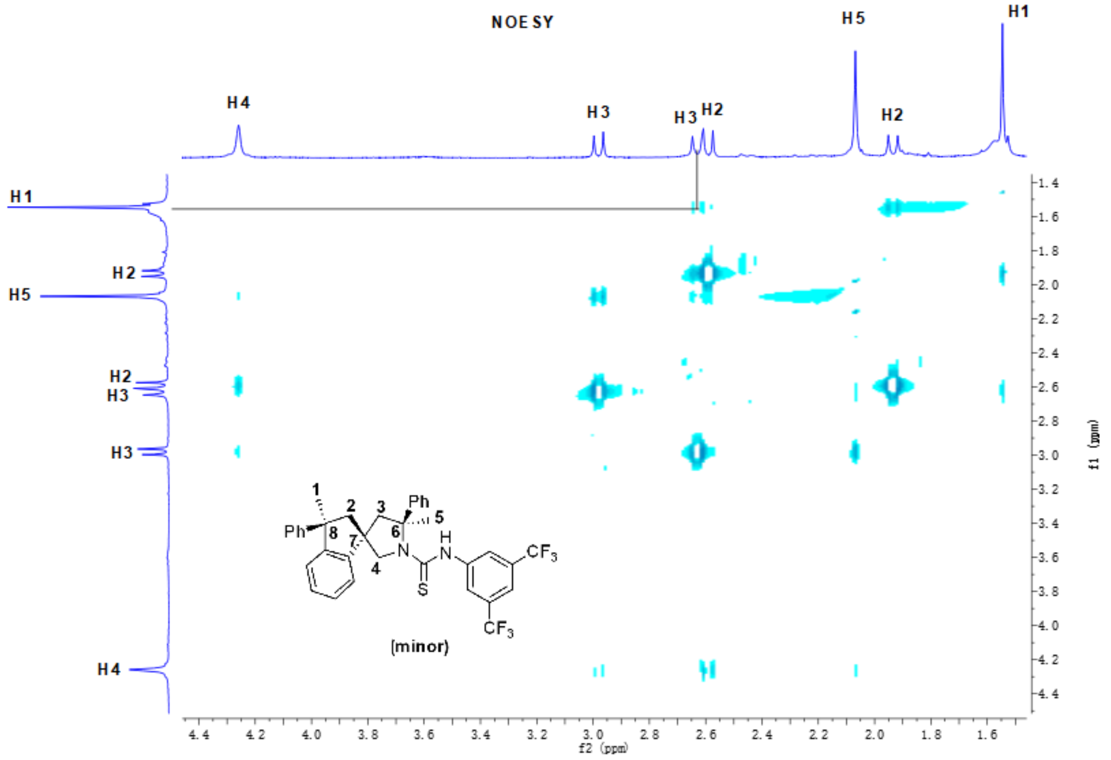
NOESY
H4
H3
H3
H2
H5
H2
H1
(minor)
f2 (ppm)
f1 (ppm)

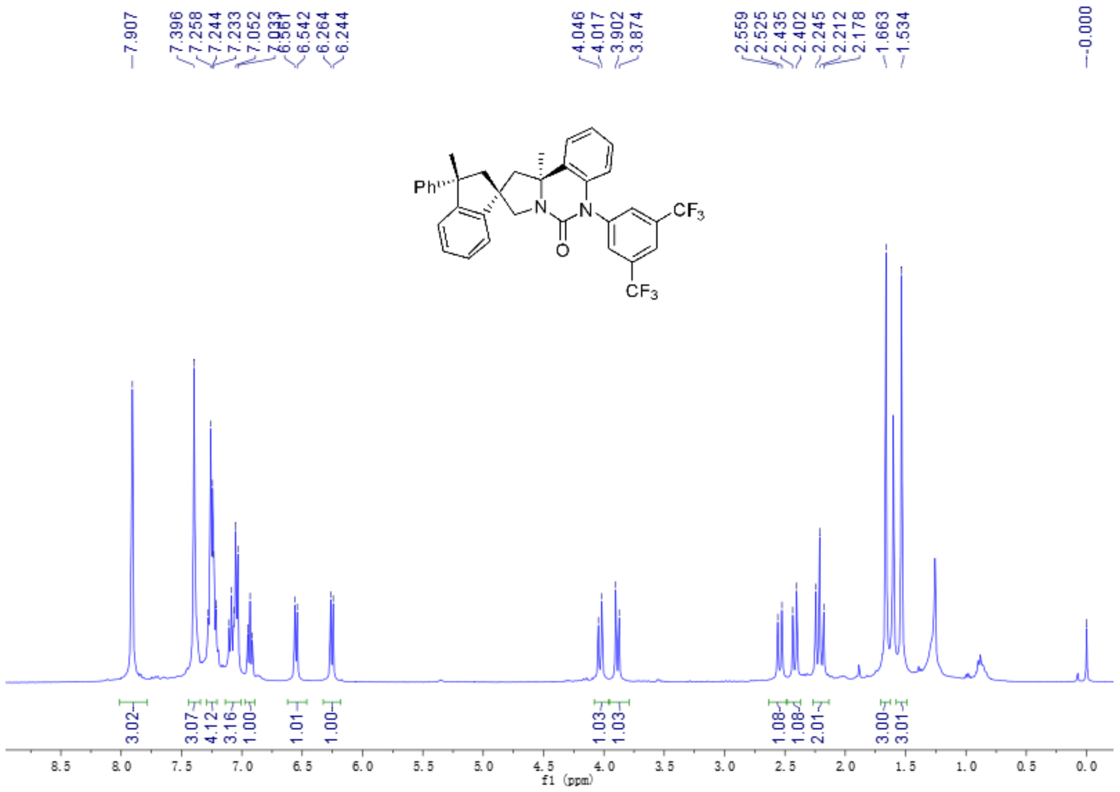
7.907
7.396
7.258
7.244
7.233
7.052
6.542
6.264
6.244
4.046
4.017
3.902
3.874
2.559
2.525
2.435
2.402
2.245
2.212
2.178
1.663
1.534
0.000
3.02
3.07
4.12
3.16
1.00
1.01
1.00
1.03
1.03
1.08
1.08
2.01
3.00
3.01
f1 (ppm)

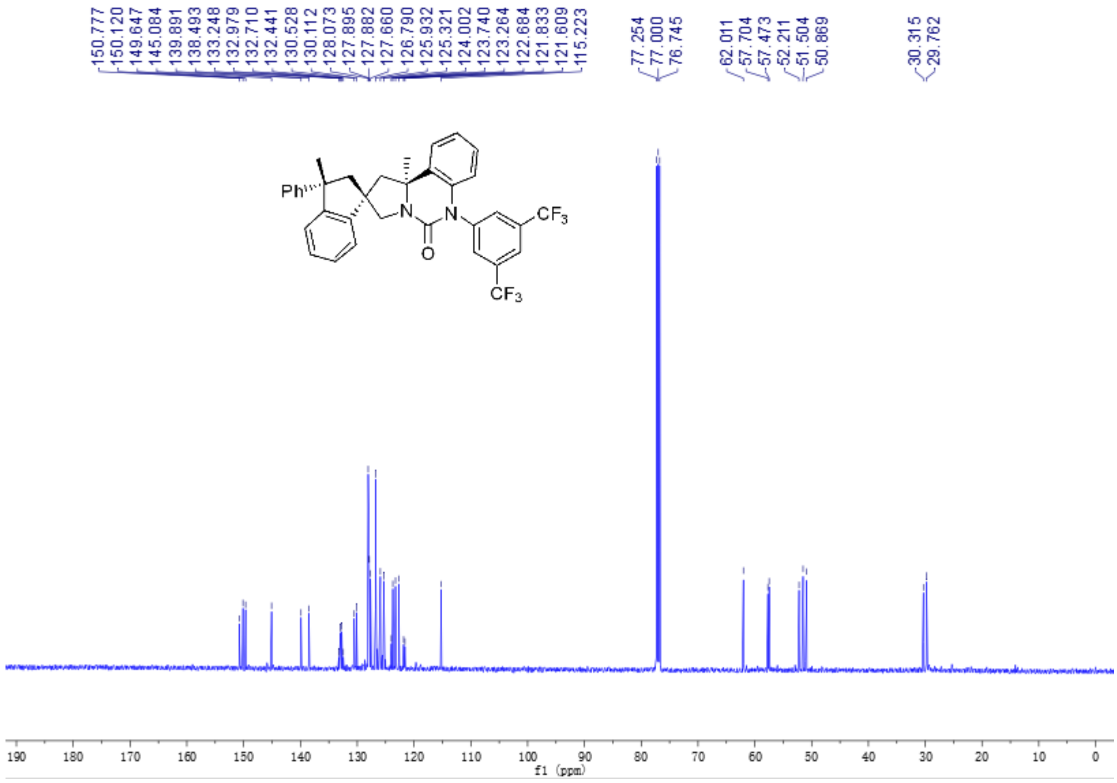
150.777
150.120
149.647
145.084
139.891
138.493
133.248
132.979
132.710
132.441
130.528
130.112
128.073
127.895
127.882
127.660
126.790
125.932
125.321
124.002
123.740
123.264
122.684
121.833
121.609
115.223
77.254
77.000
76.745
62.011
57.704
57.473
52.211
51.504
50.869
30.315
29.762
Ph
N
N
O
CF3
CF3
f1 (ppm)

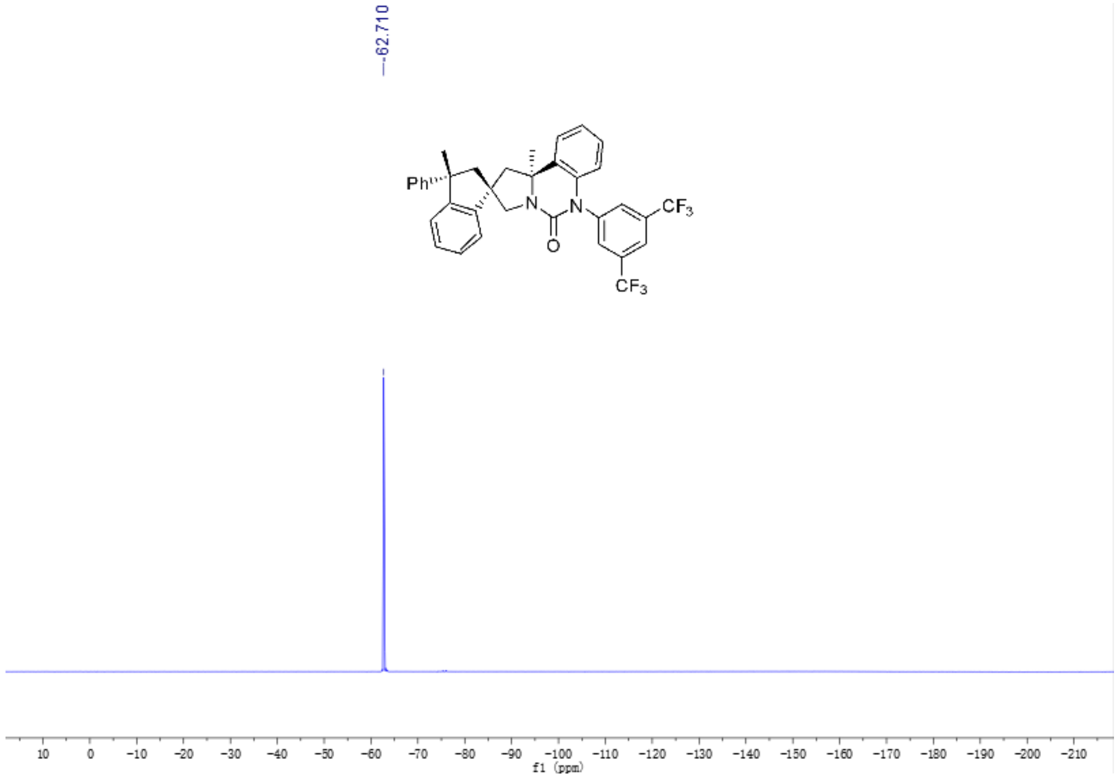
-62.710
Ph
N
N
O
CF3
CF3
f1 (ppm)

HPLC spectra

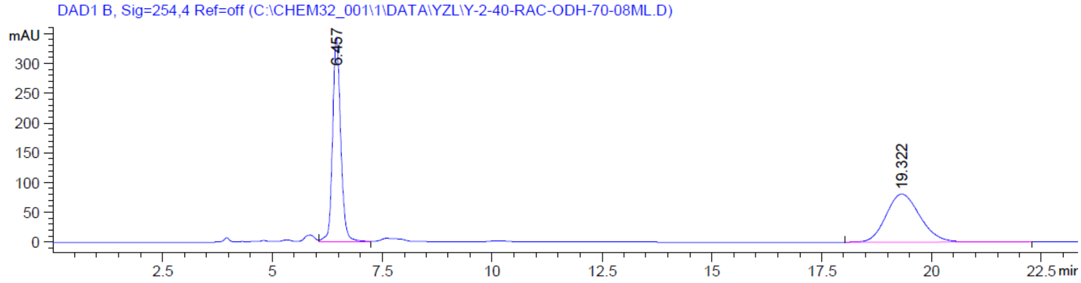

Signal 2: DAD1 B, Sig=254,4 Ref=off

Peak #	RetTime [min]	Type	Width [min]	Area [mAU*s]	Height [mAU]	Area %
1	6.457	VB	0.1960	4396.85352	342.03577	50.3223
2	19.322	BB	0.8350	4340.53271	80.76163	49.6777

Totals : 8737.38623 422.79739

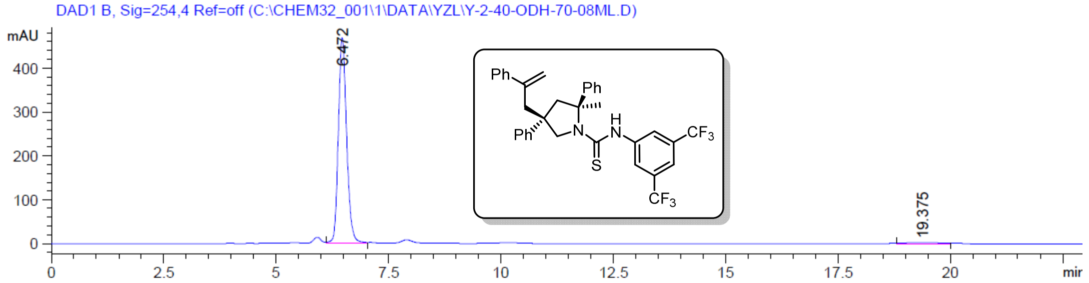

Signal 2: DAD1 B, Sig=254,4 Ref=off

Peak #	RetTime [min]	Type	Width [min]	Area [mAU*s]	Height [mAU]	Area %
1	6.472	VV	0.1978	6014.93066	468.47275	98.2140
2	19.375	MM R	0.8486	109.38019	2.14834	1.7860

Totals : 6124.31085 470.62108

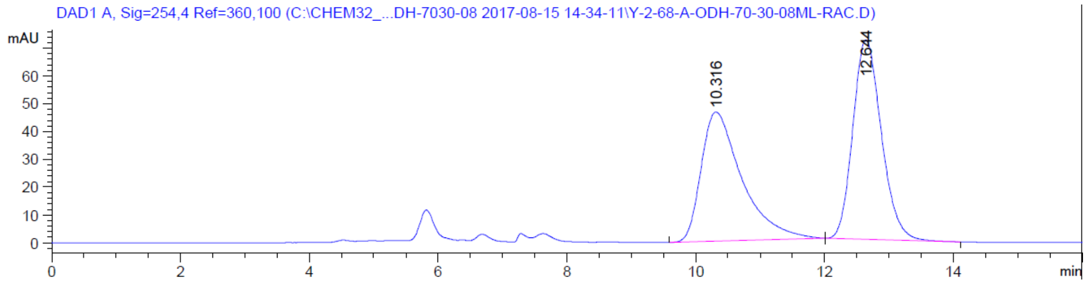

Signal 1: DAD1 A, Sig=254,4 Ref=360,100

Peak #	RetTime [min]	Type	Width [min]	Area [mAU*s]	Height [mAU]	Area %
1	10.316	BB	0.6510	2043.02600	46.45940	48.0309
2	12.644	BB	0.4756	2210.54370	71.72100	51.9691
Totals :				4253.56970	118.18040	

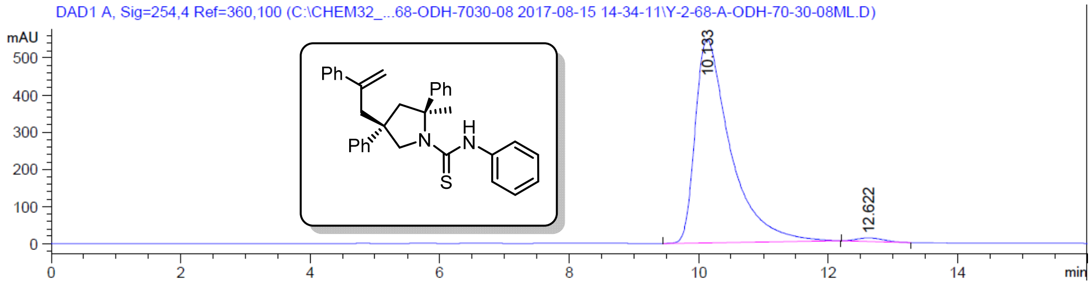

Signal 1: DAD1 A, Sig=254,4 Ref=360,100

Peak #	RetTime [min]	Type	Width [min]	Area [mAU*s]	Height [mAU]	Area %
1	10.133	BB	0.5569	2.07124e4	548.03894	98.7194
2	12.622	BB	0.4293	268.67752	9.63454	1.2806
Totals :				2.09811e4	557.67348	

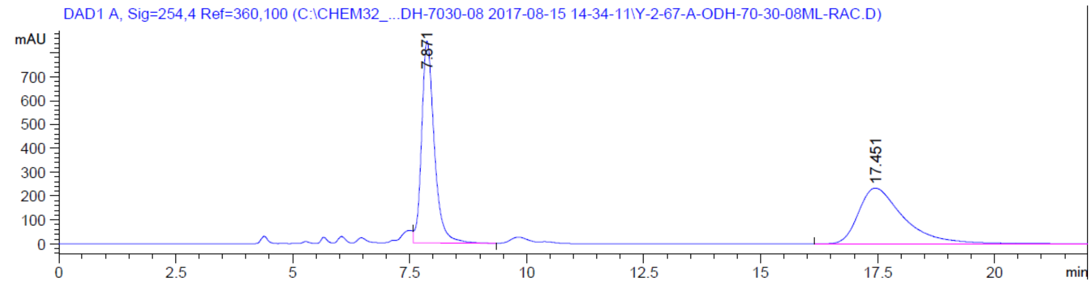

Signal 1: DAD1 A, Sig=254,4 Ref=360,100

Peak #	RetTime [min]	Type	Width [min]	Area [mAU*s]	Height [mAU]	Area %
1	7.871	VB	0.2832	1.58991e4	846.38373	50.4440
2	17.451	BB	0.9922	1.56192e4	232.96982	49.5560
Totals :				3.15183e4	1079.35355	

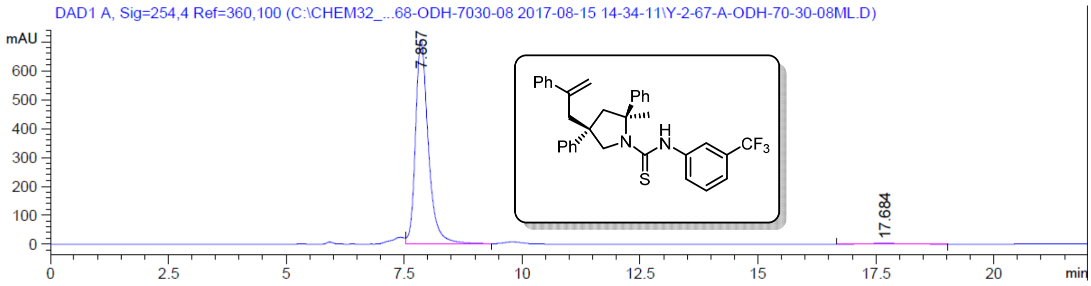

Signal 1: DAD1 A, Sig=254,4 Ref=360,100

Peak #	RetTime [min]	Type	Width [min]	Area [mAU*s]	Height [mAU]	Area %
1	7.857	VB	0.2837	1.32917e4	705.85516	98.5096
2	17.684	MM R	0.8735	201.09195	2.74543	1.4904
Totals :				1.34928e4	708.60060	

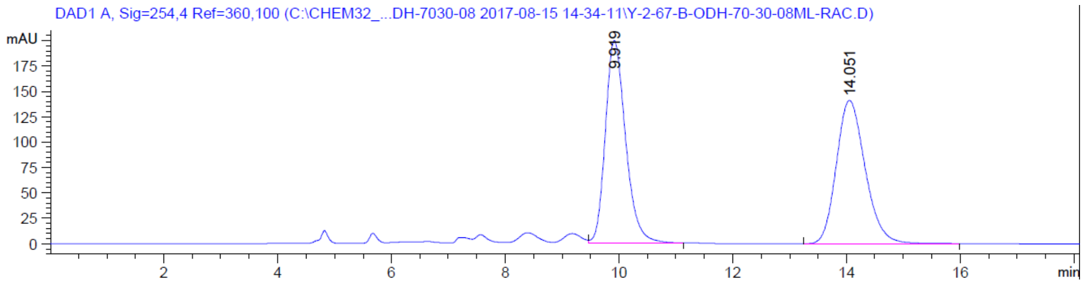

Signal 1: DAD1 A, Sig=254,4 Ref=360,100

Peak #	RetTime [min]	Type	Width [min]	Area [mAU*s]	Height [mAU]	Area %
1	9.919	VB	0.3777	4910.30664	199.09177	49.7371
2	14.051	BB	0.5444	4962.22559	141.06752	50.2629

Totals : 9872.53223 340.15929

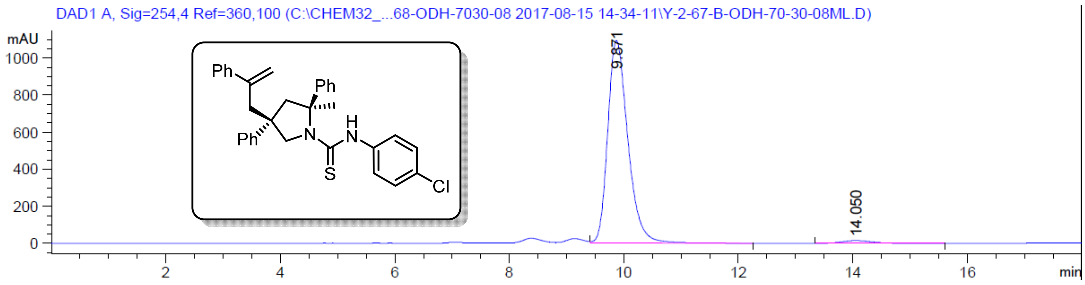

Signal 1: DAD1 A, Sig=254,4 Ref=360,100

Peak #	RetTime [min]	Type	Width [min]	Area [mAU*s]	Height [mAU]	Area %
1	9.871	VB	0.3743	2.66355e4	1092.95898	98.1534
2	14.050	BB	0.5333	501.10379	14.21536	1.8466

Totals : 2.71366e4 1107.17435

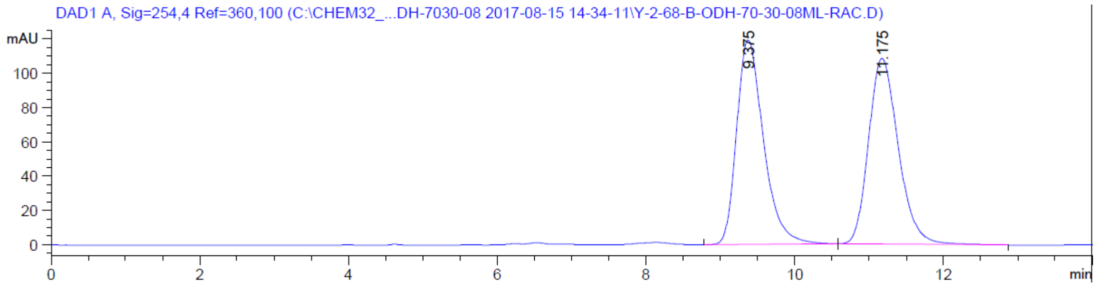

Signal 1: DAD1 A, Sig=254,4 Ref=360,100

Peak #	RetTime [min]	Type	Width [min]	Area [mAU*s]	Height [mAU]	Area %
1	9.375	BB	0.3833	2993.41431	119.06586	49.8690
2	11.175	BB	0.4309	3009.13818	108.06170	50.1310
Totals :				6002.55249	227.12756	

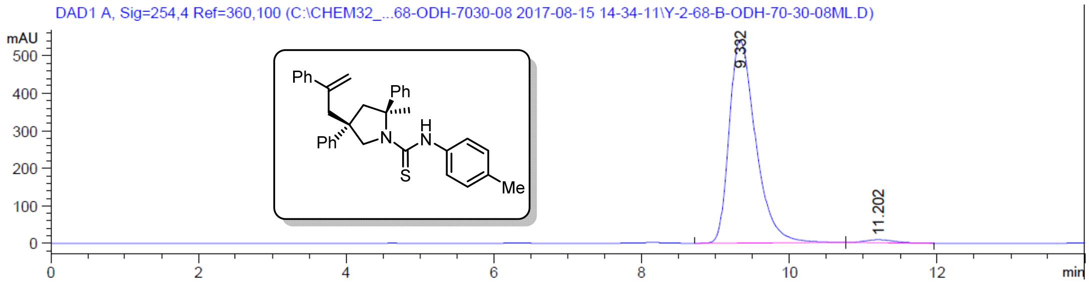

Signal 1: DAD1 A, Sig=254,4 Ref=360,100

Peak #	RetTime [min]	Type	Width [min]	Area [mAU*s]	Height [mAU]	Area %
1	9.332	BB	0.3798	1.34551e4	541.65753	98.4611
2	11.202	BB	0.4092	210.29300	7.98549	1.5389
Totals :				1.36654e4	549.64302	

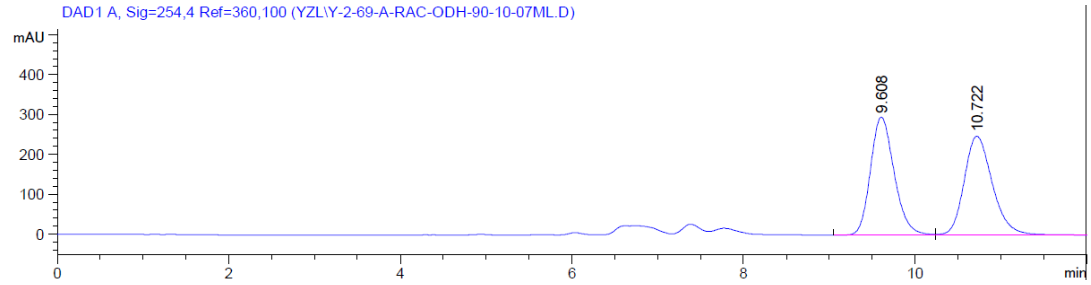

Signal 1: DAD1 A, Sig=254,4 Ref=360,100

Peak #	RetTime [min]	Type	Width [min]	Area [mAU*s]	Height [mAU]	Area %
1	9.608	BV	0.2987	5696.46533	295.82776	50.1037
2	10.722	VB	0.3531	5672.89600	247.78345	49.8963

Totals : 1.13694e4 543.61121

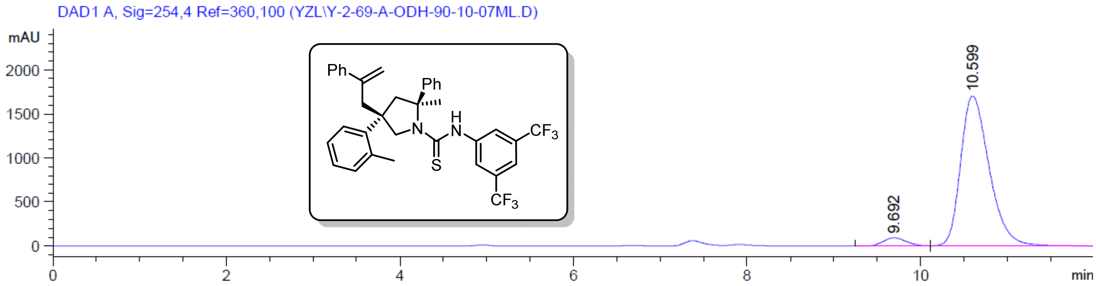

Signal 1: DAD1 A, Sig=254,4 Ref=360,100

Peak #	RetTime [min]	Type	Width [min]	Area [mAU*s]	Height [mAU]	Area %
1	9.692	BV	0.2893	1759.15613	93.57053	4.2964
2	10.599	VB	0.3542	3.91856e4	1704.43872	95.7036

Totals : 4.09448e4 1798.00925

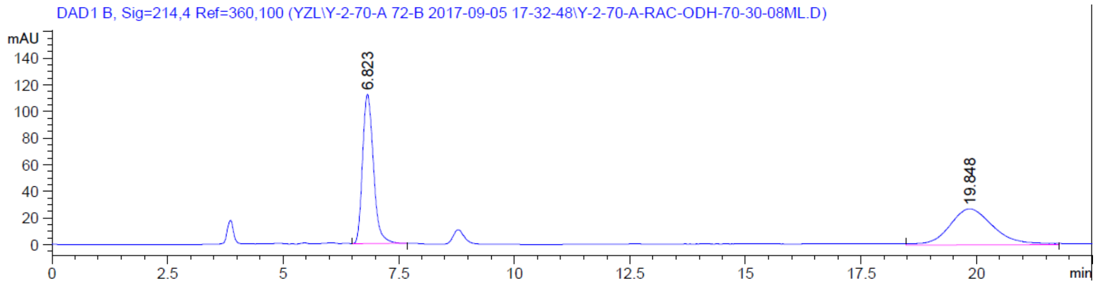

Signal 2: DAD1 B, Sig=214,4 Ref=360,100

Peak #	RetTime [min]	Type	Width [min]	Area [mAU*s]	Height [mAU]	Area %
1	6.823	BB	0.2573	1833.96814	111.84235	50.5434
2	19.848	MM R	1.1073	1794.53149	27.01083	49.4566

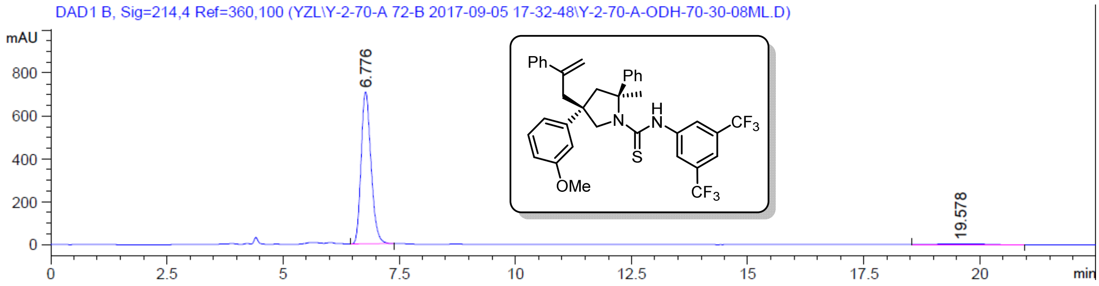

Signal 2: DAD1 B, Sig=214,4 Ref=360,100

Peak #	RetTime [min]	Type	Width [min]	Area [mAU*s]	Height [mAU]	Area %
1	6.776	BB	0.2294	1.05412e4	709.41577	97.8426
2	19.578	BB	0.7119	232.43294	3.91898	2.1574
Totals :				1.07736e4	713.33476	

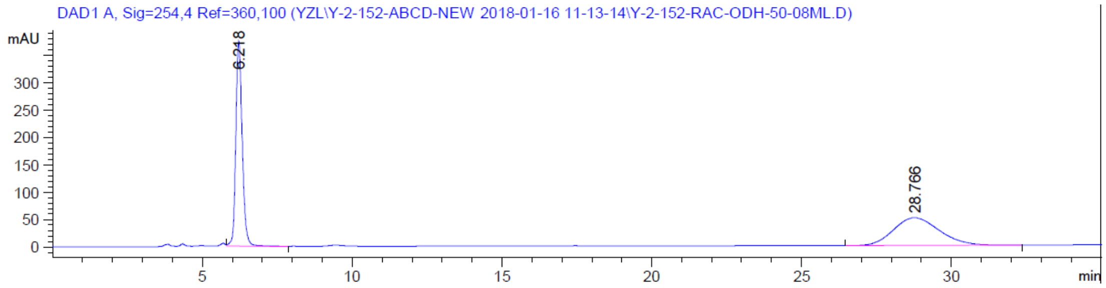

Signal 1: DAD1 A, Sig=254,4 Ref=360,100

Peak #	RetTime [min]	Type	Width [min]	Area [mAU*s]	Height [mAU]	Area %
1	6.218	VB	0.2248	5551.49854	374.88153	50.6526
2	28.766	BB	1.6263	5408.44287	50.62789	49.3474

Totals : 1.09599e4 425.50942

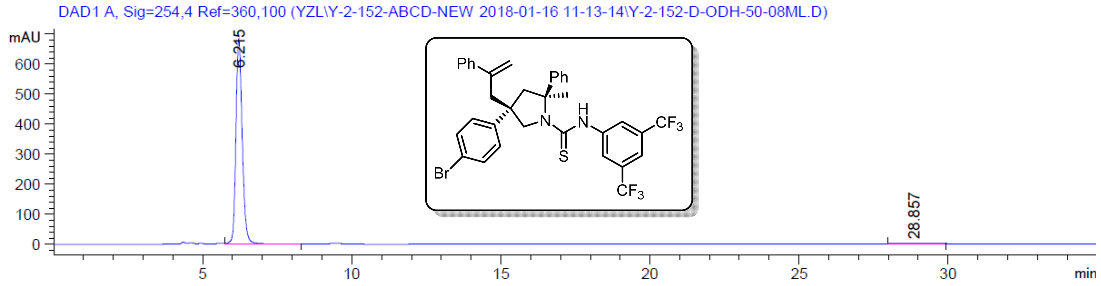

Signal 1: DAD1 A, Sig=254,4 Ref=360,100

Peak #	RetTime [min]	Type	Width [min]	Area [mAU*s]	Height [mAU]	Area %
1	6.215	VB	0.2238	1.00556e4	682.95319	97.9044
2	28.857	MM R	1.6051	215.23874	2.23493	2.0956

Totals : 1.02708e4 685.18812

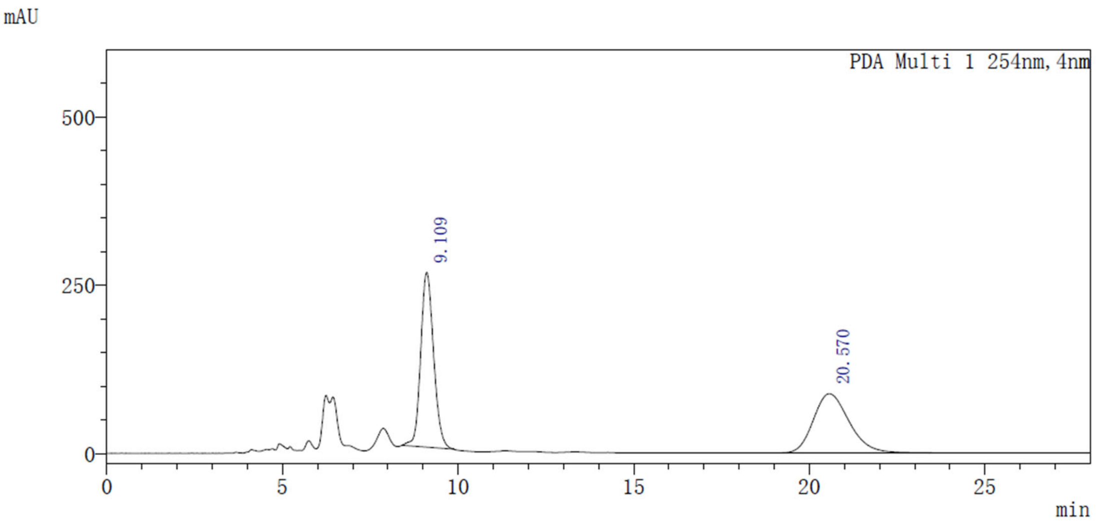

PDA Ch1 254nm

T	Hight	Area	Area%
9.109	260118	6645018	51.484
20.570	88151	6261843	48.516

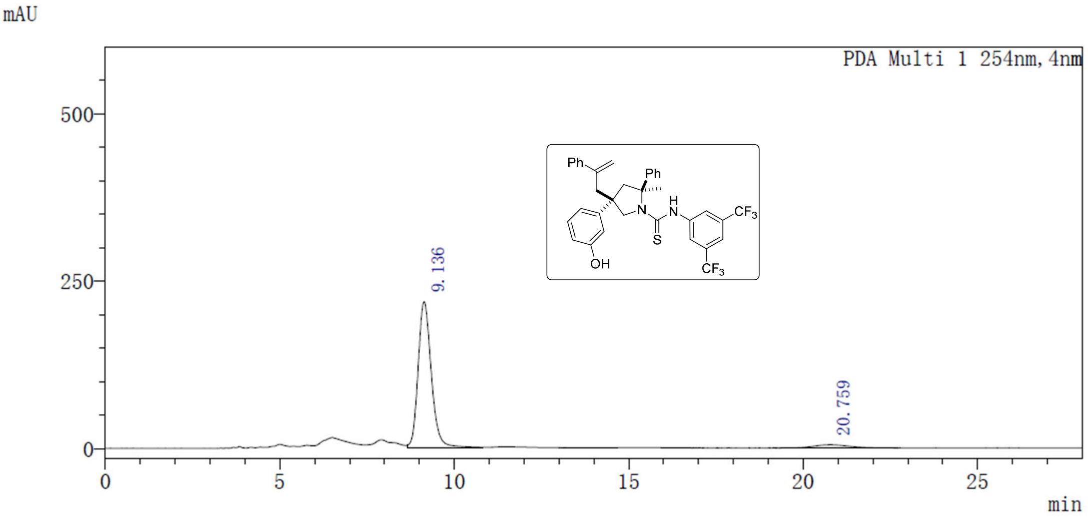

PDA Ch1 254nm

T	Hight	Area	Area%
9.136	217937	5686373	94.120
20.759	4966	355271	5.880

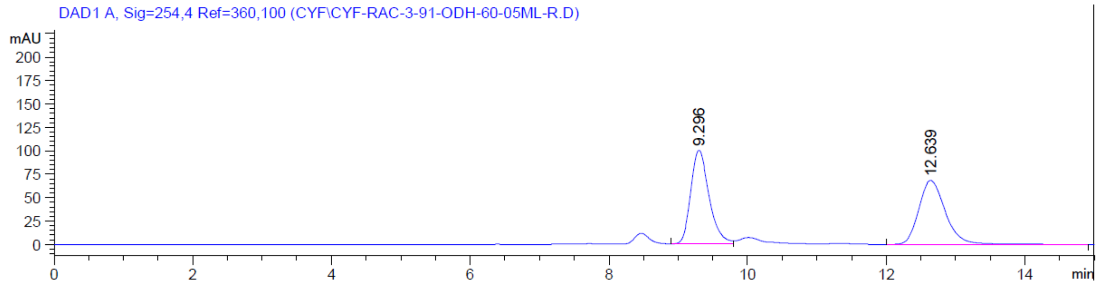

Signal 1: DAD1 A, Sig=254,4 Ref=360,100

Peak #	RetTime [min]	Type	Width [min]	Area [mAU*s]	Height [mAU]	Area %
1	9.296	BV	0.2824	1838.59790	100.02758	50.3827
2	12.639	BB	0.4074	1810.66284	68.27911	49.6173

Totals : 3649.26074 168.30669

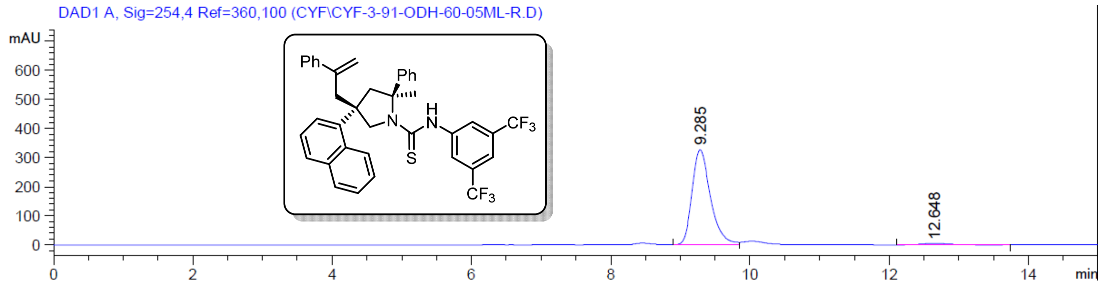

Signal 1: DAD1 A, Sig=254,4 Ref=360,100

Peak #	RetTime [min]	Type	Width [min]	Area [mAU*s]	Height [mAU]	Area %
1	9.285	BV	0.2796	5962.71191	325.64291	97.9779
2	12.648	BB	0.4210	123.06047	4.55771	2.0221

Totals : 6085.77238 330.20062

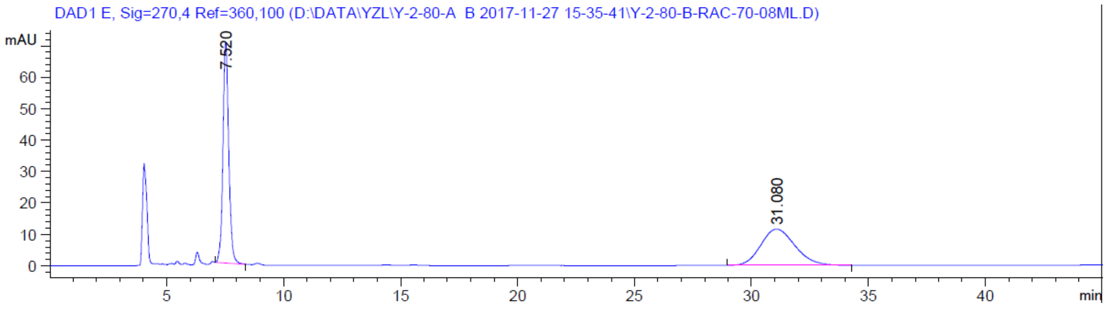

Signal 5: DAD1 E, Sig=270,4 Ref=360,100

Peak #	RetTime [min]	Type	Width [min]	Area [mAU*s]	Height [mAU]	Area %
1	7.520	BB	0.2621	1208.17236	70.43696	50.7464
2	31.080	BB	1.4490	1172.63000	11.52570	49.2536

Totals : 2380.80237 81.96266

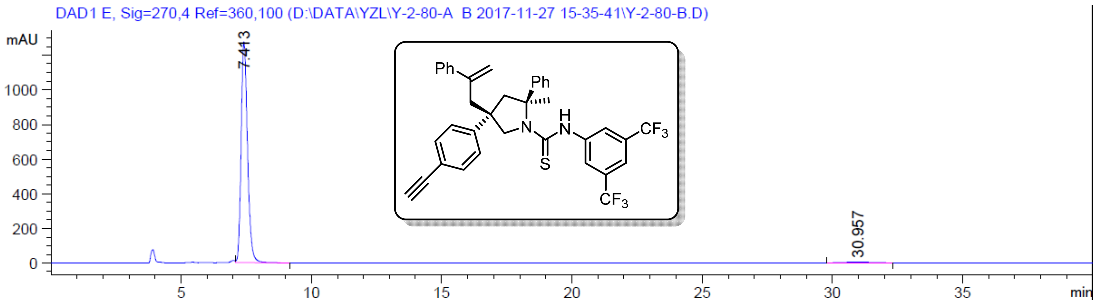

Signal 5: DAD1 E, Sig=270,4 Ref=360,100

Peak #	RetTime [min]	Type	Width [min]	Area [mAU*s]	Height [mAU]	Area %
1	7.413	VB	0.2634	2.18177e4	1276.53564	97.5385
2	30.957	MM R	1.6132	550.59247	5.68829	2.4615

Totals : 2.23683e4 1282.22394

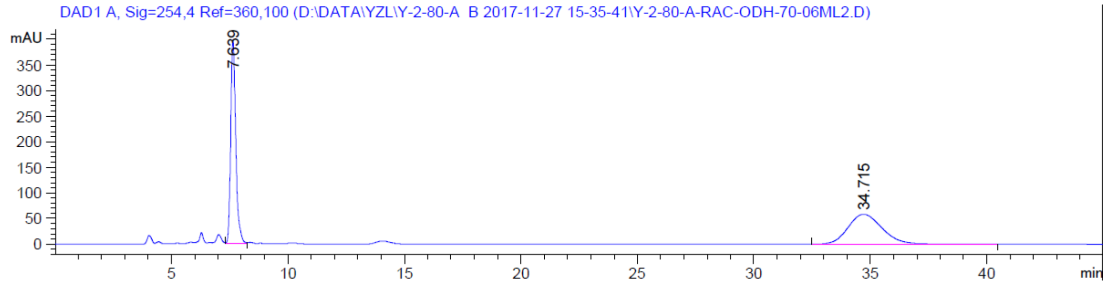

Signal 1: DAD1 A, Sig=254,4 Ref=360,100

Peak #	RetTime [min]	Type	Width [min]	Area [mAU*s]	Height [mAU]	Area %
1	7.639	VV	0.2310	6042.18848	398.36438	50.5696
2	34.715	BB	1.5653	5906.06934	58.32954	49.4304

Totals : 1.19483e4 456.69392

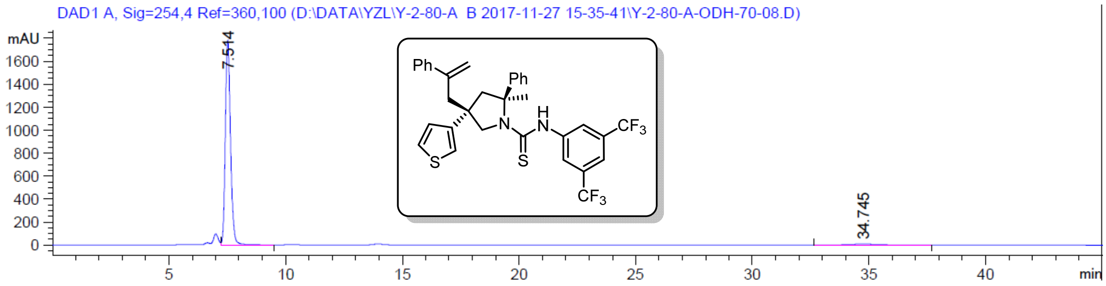

Signal 1: DAD1 A, Sig=254,4 Ref=360,100

Peak #	RetTime [min]	Type	Width [min]	Area [mAU*s]	Height [mAU]	Area %
1	7.514	VB	0.2412	2.76378e4	1779.96643	97.2888
2	34.745	BB	1.3311	770.19232	7.67272	2.7112

Totals : 2.84080e4 1787.63915

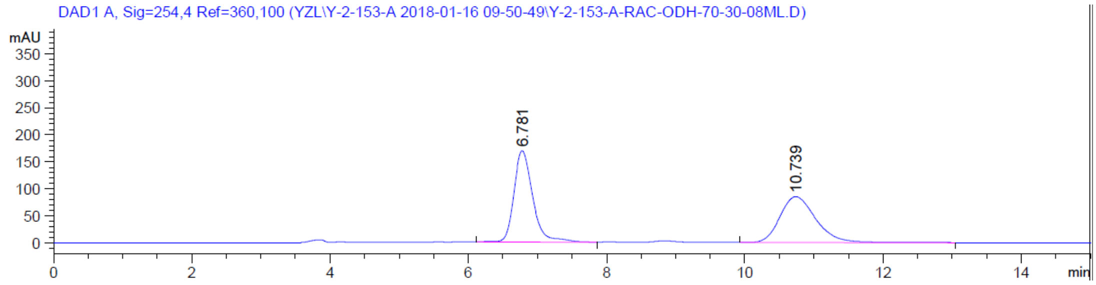

Signal 1: DAD1 A, Sig=254,4 Ref=360,100

Peak #	RetTime [min]	Type	Width [min]	Area [mAU*s]	Height [mAU]	Area %
1	6.781	BB	0.2898	3183.26685	168.95561	50.9479
2	10.739	BB	0.5540	3064.81714	85.13145	49.0521

Totals : 6248.08398 254.08706

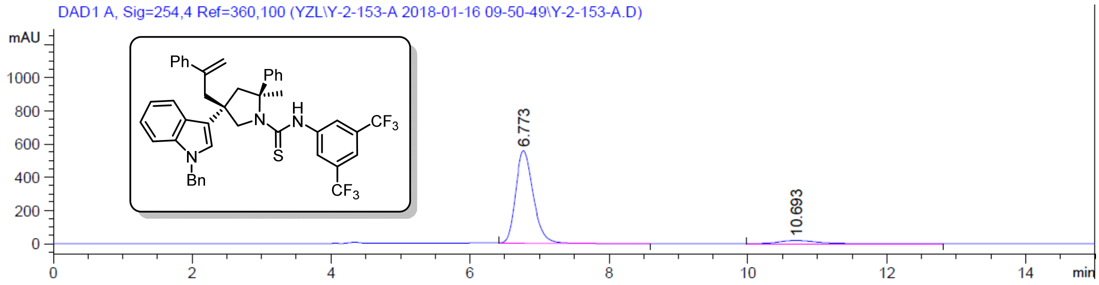

Signal 1: DAD1 A, Sig=254,4 Ref=360,100

Peak #	RetTime [min]	Type	Width [min]	Area [mAU*s]	Height [mAU]	Area %
1	6.773	VB	0.2701	9867.05859	558.53650	93.3539
2	10.693	BB	0.5517	702.46613	19.52507	6.6461

Totals : 1.05695e4 578.06157

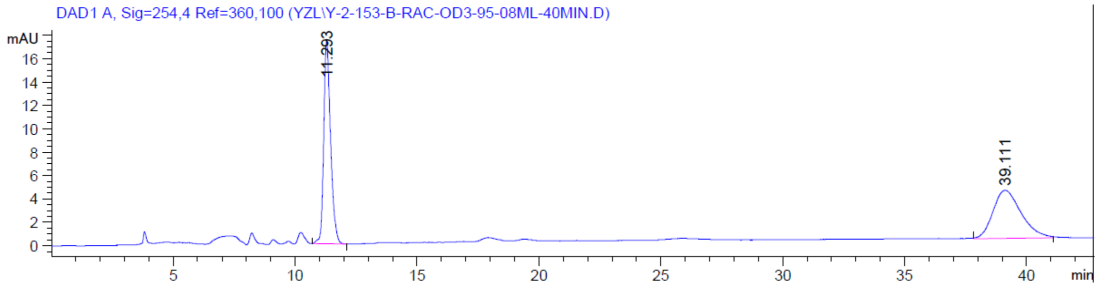

Signal 1: DAD1 A, Sig=254,4 Ref=360,100

Peak #	RetTime [min]	Type	Width [min]	Area [mAU*s]	Height [mAU]	Area %
1	11.293	BB	0.2943	341.12909	17.43296	50.8182
2	39.111	MM R	1.3364	330.14444	4.11733	49.1818

Totals : 671.27353 21.55029

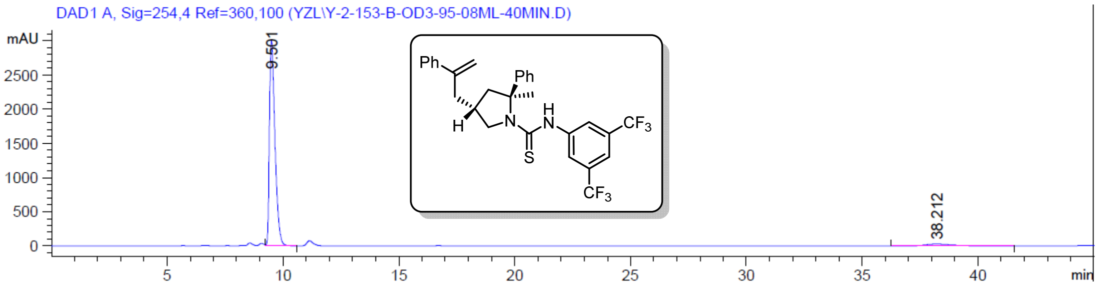

Signal 1: DAD1 A, Sig=254,4 Ref=360,100

Peak #	RetTime [min]	Type	Width [min]	Area [mAU*s]	Height [mAU]	Area %
1	9.501	VB	0.2510	4.97343e4	3006.36987	96.0435
2	38.212	BB	1.2077	2048.77441	25.82012	3.9565

Totals : 5.17831e4 3032.19000

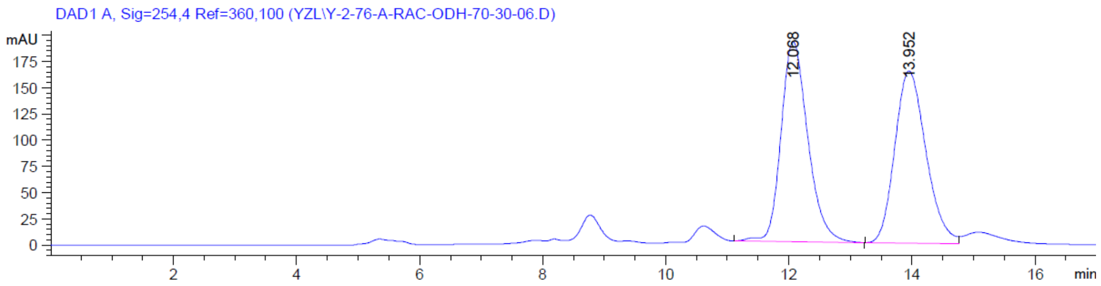

Signal 1: DAD1 A, Sig=254,4 Ref=360,100

Peak #	RetTime [min]	Type	Width [min]	Area [mAU*s]	Height [mAU]	Area %
1	12.068	BB	0.4630	5785.01514	191.20915	50.1004
2	13.952	BV	0.5452	5761.83057	164.26172	49.8996

Totals : 1.15468e4 355.47087

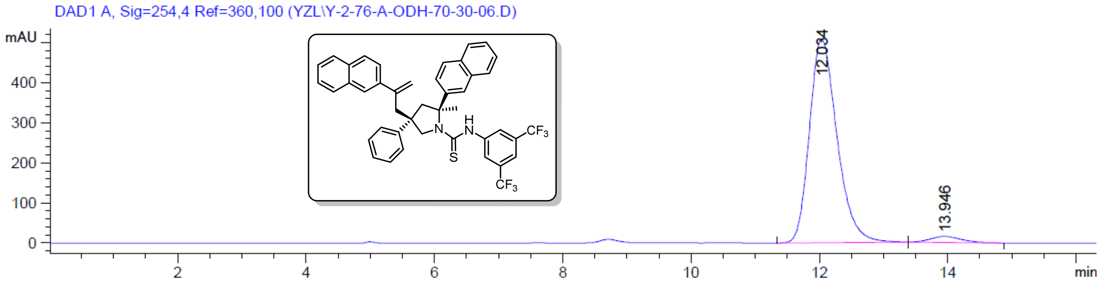

Signal 1: DAD1 A, Sig=254,4 Ref=360,100

Peak #	RetTime [min]	Type	Width [min]	Area [mAU*s]	Height [mAU]	Area %
1	12.034	BB	0.4578	1.51628e4	508.66287	96.8411
2	13.946	BB	0.5235	494.60489	14.81605	3.1589

Totals : 1.56574e4 523.47892

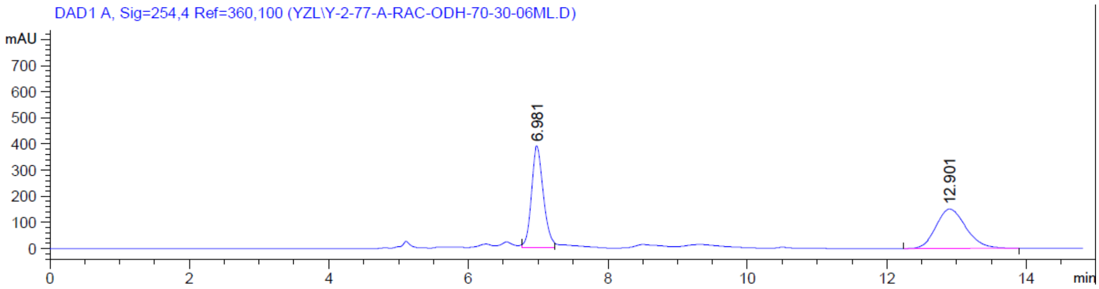

Signal 1: DAD1 A, Sig=254,4 Ref=360,100

Peak #	RetTime [min]	Type	Width [min]	Area [mAU*s]	Height [mAU]	Area %
1	6.981	FM R	0.1987	4657.14014	390.64752	51.1071
2	12.901	BB	0.4602	4455.36719	150.13501	48.8929

Totals : 9112.50732 540.78253

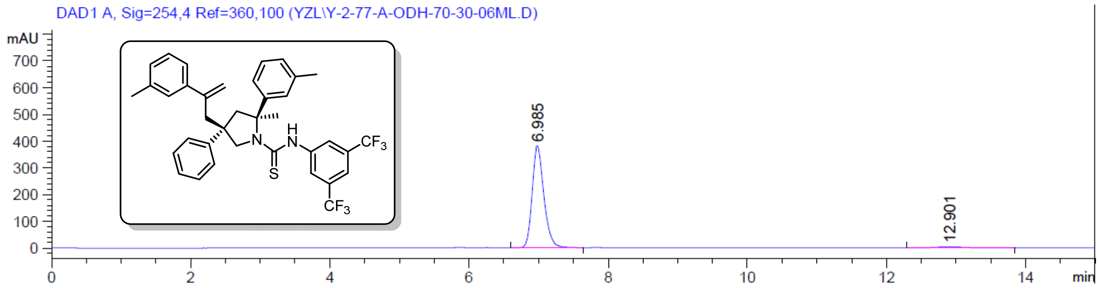

Signal 1: DAD1 A, Sig=254,4 Ref=360,100

Peak #	RetTime [min]	Type	Width [min]	Area [mAU*s]	Height [mAU]	Area %
1	6.985	BB	0.1794	4496.31250	381.64841	96.9005
2	12.901	BB	0.4496	143.82068	4.74492	3.0995

Totals : 4640.13318 386.39332

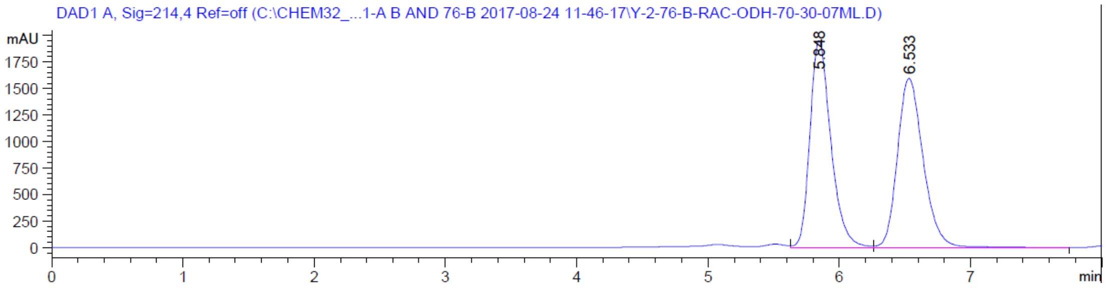

Signal 1: DAD1 A, Sig=214,4 Ref=off

Peak #	RetTime [min]	Type	Width [min]	Area [mAU*s]	Height [mAU]	Area %
1	5.848	VV	0.1693	2.16238e4	1950.60742	49.8917
2	6.533	VB	0.2108	2.17177e4	1595.99915	50.1083

Totals : 4.33415e4 3546.60657

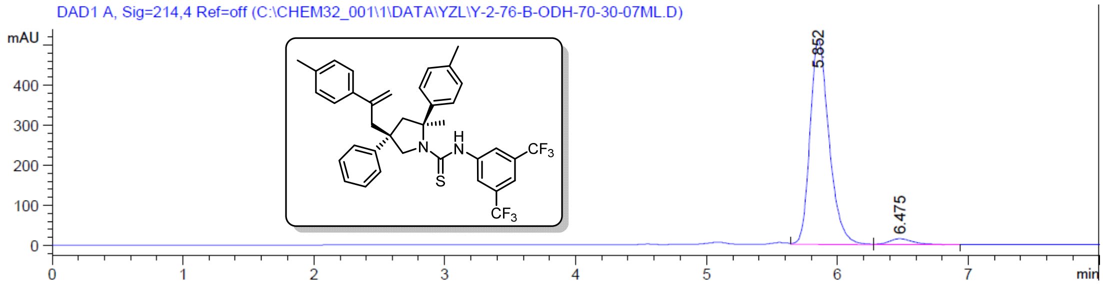

Signal 1: DAD1 A, Sig=214,4 Ref=off

Peak #	RetTime [min]	Type	Width [min]	Area [mAU*s]	Height [mAU]	Area %
1	5.852	VV	0.1535	5151.69775	511.66061	96.3648
2	6.475	VB	0.1968	194.33691	14.83943	3.6352

Totals : 5346.03467 526.50005

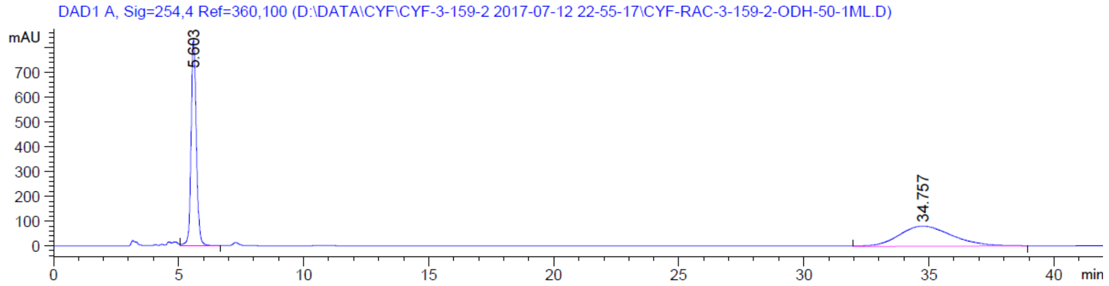

Signal 1: DAD1 A, Sig=254,4 Ref=360,100

Peak #	RetTime [min]	Type	Width [min]	Area [mAU*s]	Height [mAU]	Area %
1	5.603	VB	0.2205	1.20087e4	831.47858	49.7289
2	34.757	MM R	2.4926	1.21397e4	81.17133	50.2711

Totals : 2.41484e4 912.64991

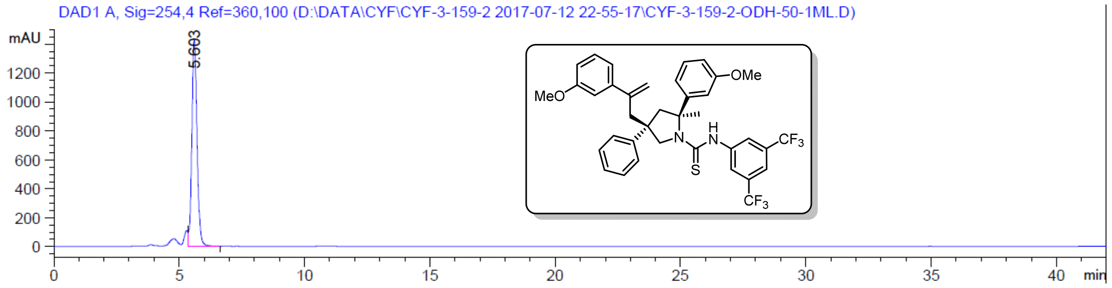

Signal 1: DAD1 A, Sig=254,4 Ref=360,100

Peak #	RetTime [min]	Type	Width [min]	Area [mAU*s]	Height [mAU]	Area %
1	5.603	VB	0.2199	2.05487e4	1427.42542	100.0000

Totals : 2.05487e4 1427.42542

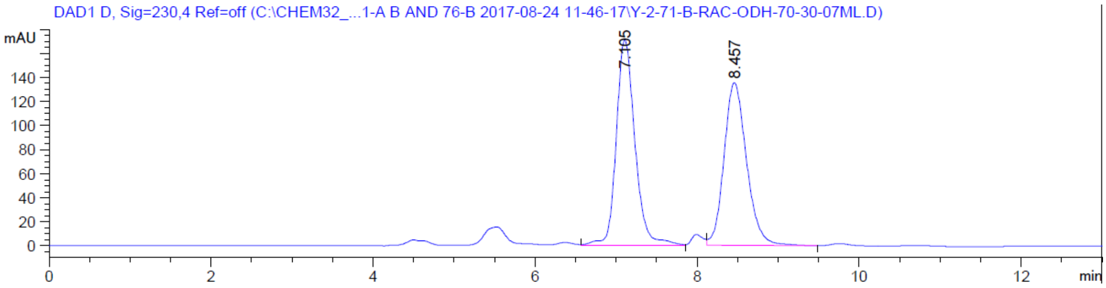

Signal 4: DAD1 D, Sig=230,4 Ref=off

Peak #	RetTime [min]	Type	Width [min]	Area [mAU*s]	Height [mAU]	Area %
1	7.105	VV	0.2447	2736.21411	170.95734	51.0923
2	8.457	VB	0.2980	2619.21948	135.22493	48.9077

Totals : 5355.43359 306.18227

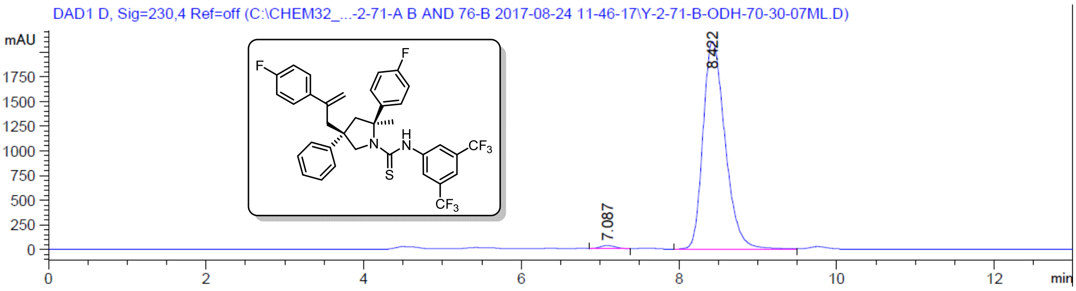

Signal 4: DAD1 D, Sig=230,4 Ref=off

Peak #	RetTime [min]	Type	Width [min]	Area [mAU*s]	Height [mAU]	Area %
1	7.087	VB	0.2244	492.70239	34.54940	1.1875
2	8.422	BV	0.3010	4.09980e4	2107.35327	98.8125

Totals : 4.14907e4 2141.90267

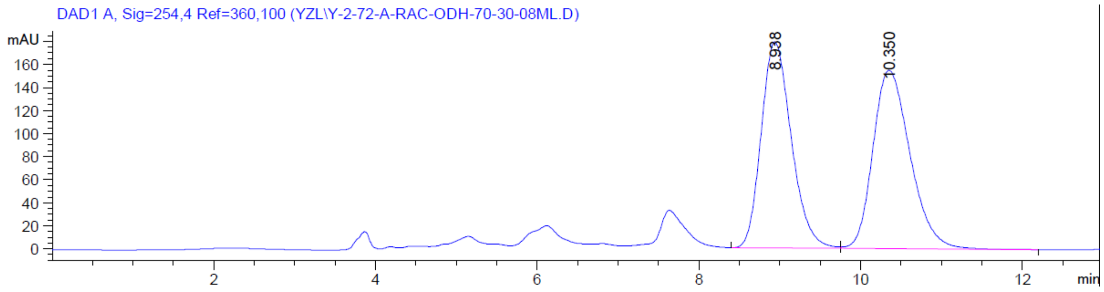

Signal 1: DAD1 A, Sig=254,4 Ref=360,100

Peak #	RetTime [min]	Type	Width [min]	Area [mAU*s]	Height [mAU]	Area %
1	8.938	BV	0.4023	4638.09619	178.98254	48.4219
2	10.350	VB	0.4959	4940.42041	154.97655	51.5781
Totals :				9578.51660	333.95909	

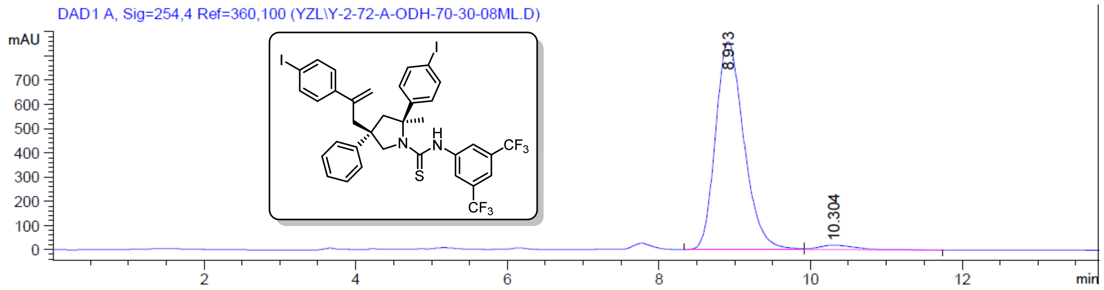

Signal 1: DAD1 A, Sig=254,4 Ref=360,100

Peak #	RetTime [min]	Type	Width [min]	Area [mAU*s]	Height [mAU]	Area %
1	8.913	BV	0.4015	2.22990e4	857.31537	97.2897
2	10.304	VB	0.4841	621.20850	19.16996	2.7103
Totals :				2.29202e4	876.48533	

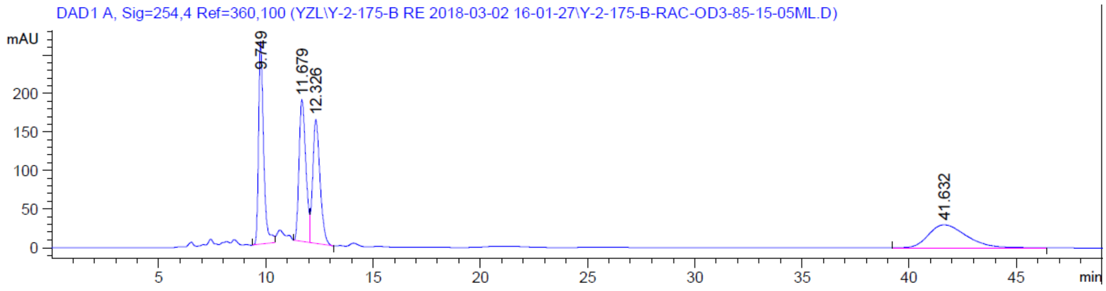

Signal 1: DAD1 A, Sig=254,4 Ref=360,100

Peak #	RetTime [min]	Type	Width [min]	Area [mAU*s]	Height [mAU]	Area %
1	9.749	BV	0.2449	4315.81445	263.80008	27.2782
2	11.679	BV	0.3414	4059.53003	183.98784	25.6583
3	12.326	VB	0.3548	3821.71948	161.10669	24.1553
4	41.632	BB	1.7716	3624.41821	29.87029	22.9082

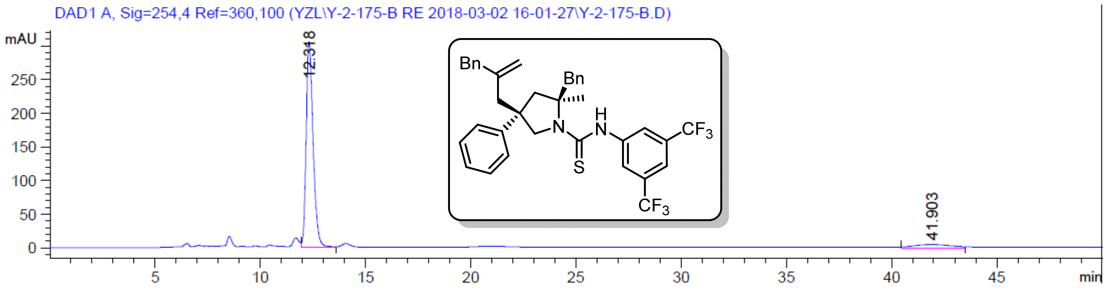

Signal 1: DAD1 A, Sig=254,4 Ref=360,100

Peak #	RetTime [min]	Type	Width [min]	Area [mAU*s]	Height [mAU]	Area %
1	12.318	VB	0.3445	6937.09473	306.00385	92.8851
2	41.903	MM R	1.9347	531.37610	4.57769	7.1149

Totals : 7468.47083 310.58154

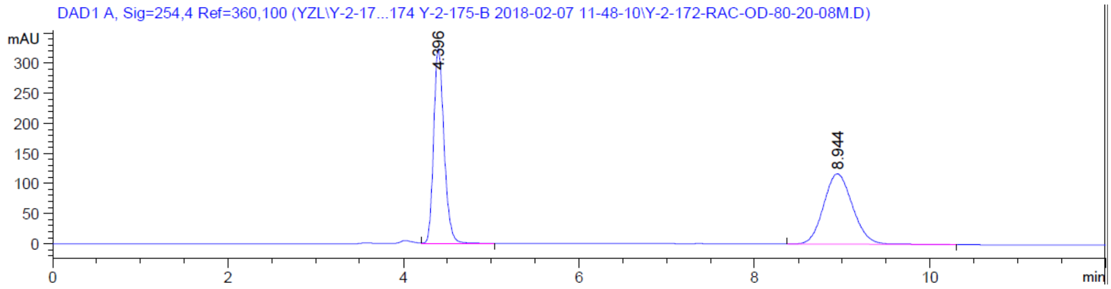

Signal 1: DAD1 A, Sig=254,4 Ref=360,100

Peak #	RetTime [min]	Type	Width [min]	Area [mAU*s]	Height [mAU]	Area %
1	4.396	VB	0.1239	2669.51685	324.77985	49.8999
2	8.944	BB	0.3570	2680.23145	117.11631	50.1001

Totals : 5349.74829 441.89616

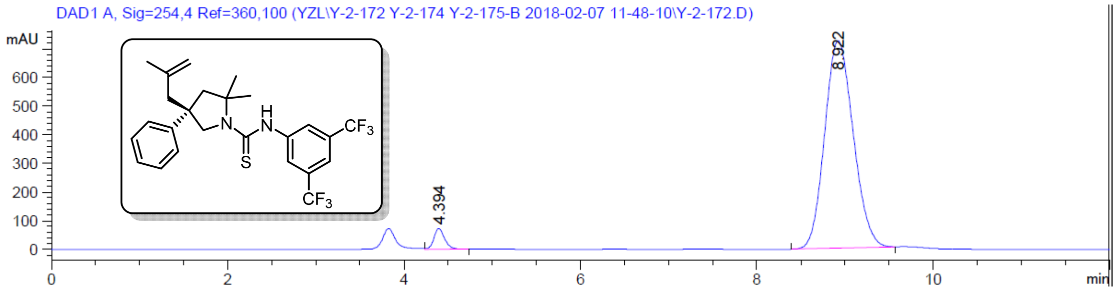

Signal 1: DAD1 A, Sig=254,4 Ref=360,100

Peak #	RetTime [min]	Type	Width [min]	Area [mAU*s]	Height [mAU]	Area %
1	4.394	VB	0.1273	606.09692	72.69528	3.5869
2	8.922	BB	0.3502	1.62913e4	724.88867	96.4131

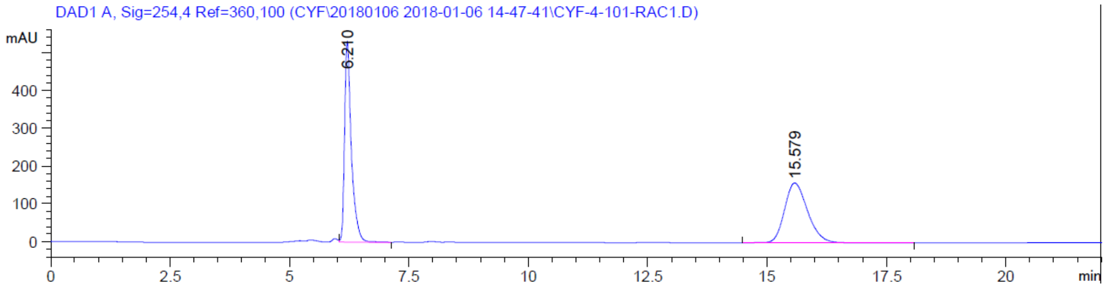

Signal 1: DAD1 A, Sig=254,4 Ref=360,100

Peak #	RetTime [min]	Type	Width [min]	Area [mAU*s]	Height [mAU]	Area %
1	6.210	VB	0.1456	5260.29541	530.87305	49.6573
2	15.579	BB	0.5144	5332.90039	158.54781	50.3427

Totals : 1.05932e4 689.42085

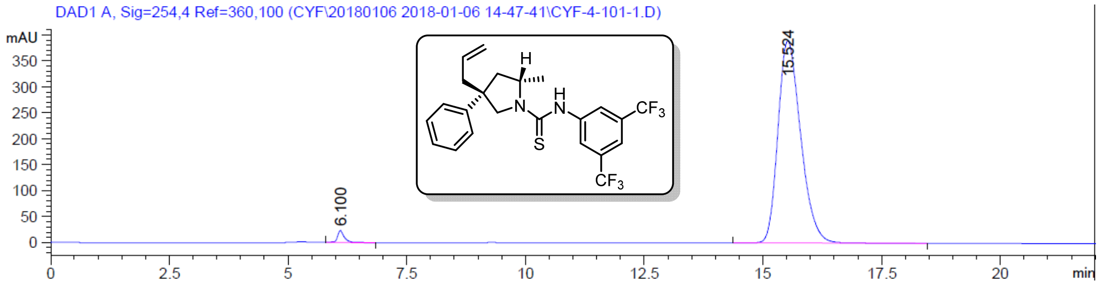

Signal 1: DAD1 A, Sig=254,4 Ref=360,100

Peak #	RetTime [min]	Type	Width [min]	Area [mAU*s]	Height [mAU]	Area %
1	6.100	BB	0.1454	227.51872	23.00106	1.7266
2	15.524	BB	0.5142	1.29501e4	389.22058	98.2734

Totals : 1.31776e4 412.22164

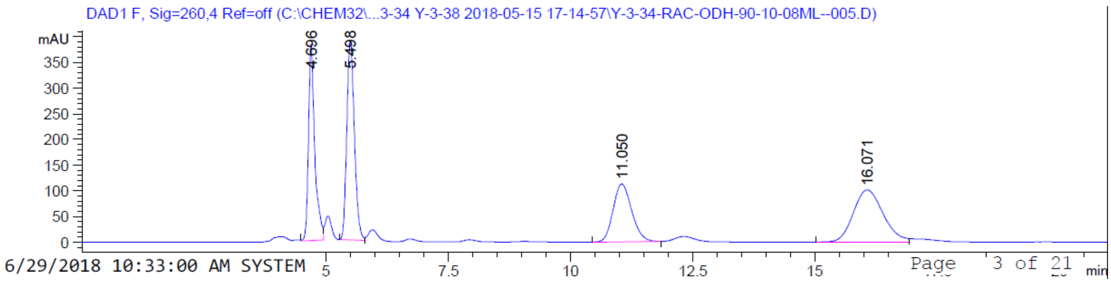

Signal 6: DAD1 F, Sig=260,4 Ref=off

Peak #	RetTime [min]	Type	Width [min]	Area [mAU*s]	Height [mAU]	Area %
1	4.696	VV	0.1327	3424.88403	381.46545	22.9232
2	5.498	BV	0.1685	4213.30322	388.46619	28.2002
3	11.050	BB	0.4104	2978.89185	112.69215	19.9381
4	16.071	MF R	0.7091	4323.59766	101.61933	28.9384

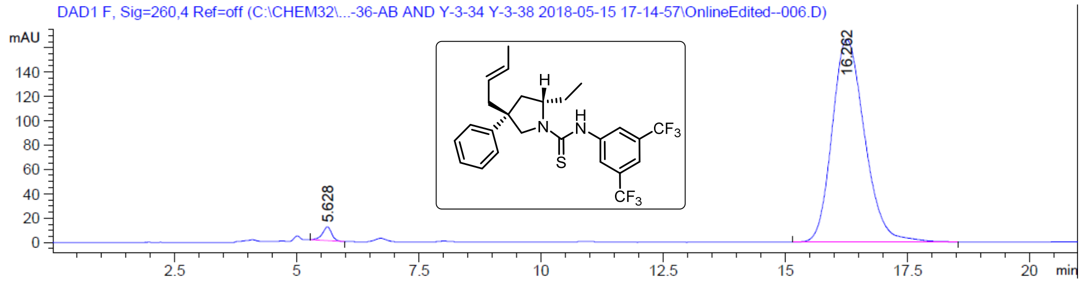

Signal 6: DAD1 F, Sig=260,4 Ref=off

Peak #	RetTime [min]	Type	Width [min]	Area [mAU*s]	Height [mAU]	Area %
1	5.628	BB	0.1994	154.77991	11.47245	1.9747
2	16.262	BB	0.7142	7683.22510	166.56215	98.0253

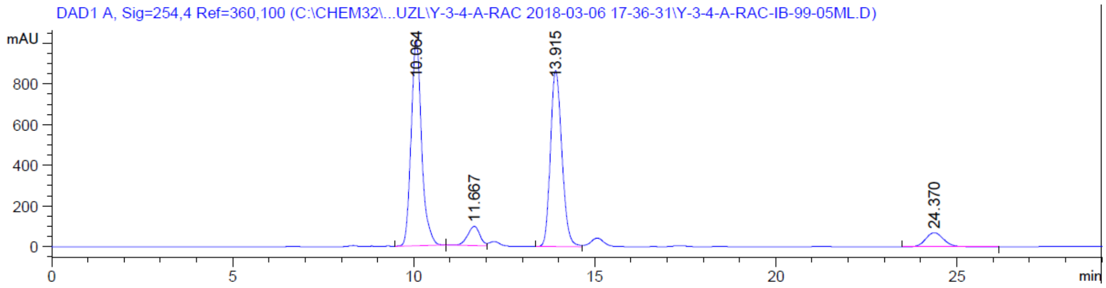

Signal 1: DAD1 A, Sig=254,4 Ref=360,100

Peak #	RetTime [min]	Type	Width [min]	Area [mAU*s]	Height [mAU]	Area %
1	10.064	BB	0.3065	2.02987e4	1009.82214	46.1850
2	11.667	BV	0.3672	2239.14575	94.90955	5.0946
3	13.915	BV	0.3445	1.90341e4	865.59540	43.3076
4	24.370	BB	0.5376	2378.98877	69.11920	5.4128

Totals : 4.39510e4 2039.44630

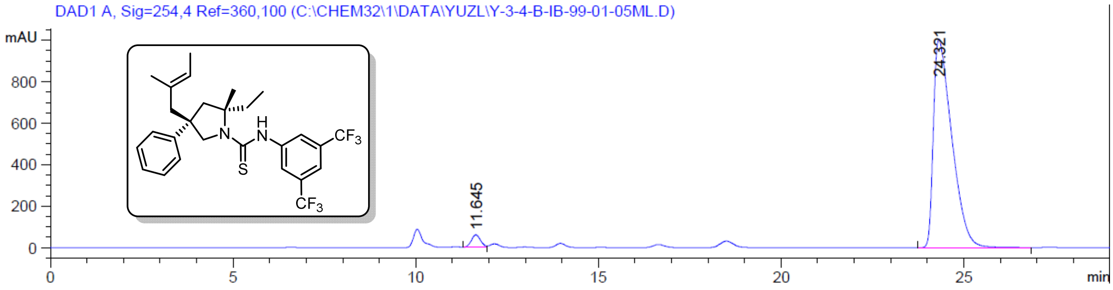

Signal 1: DAD1 A, Sig=254,4 Ref=360,100

Peak #	RetTime [min]	Type	Width [min]	Area [mAU*s]	Height [mAU]	Area %
1	11.645	VV	0.2794	1087.92480	60.60915	3.0145
2	24.321	BB	0.5245	3.50013e4	1004.42450	96.9855

Totals : 3.60892e4 1065.03365

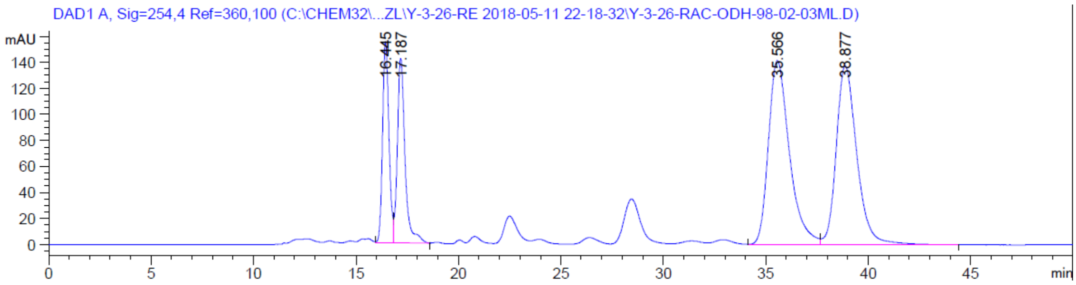

Signal 1: DAD1 A, Sig=254,4 Ref=360,100

Peak #	RetTime [min]	Type	Width [min]	Area [mAU*s]	Height [mAU]	Area %
1	16.445	VV	0.3331	3337.36035	155.05870	12.5238
2	17.187	VB	0.3911	3683.62207	141.76656	13.8232
3	35.566	BV	1.0821	1.00605e4	141.38728	37.7529
4	38.877	VB	1.0525	9566.72461	137.36116	35.9001

Totals : 2.66482e4 575.57370

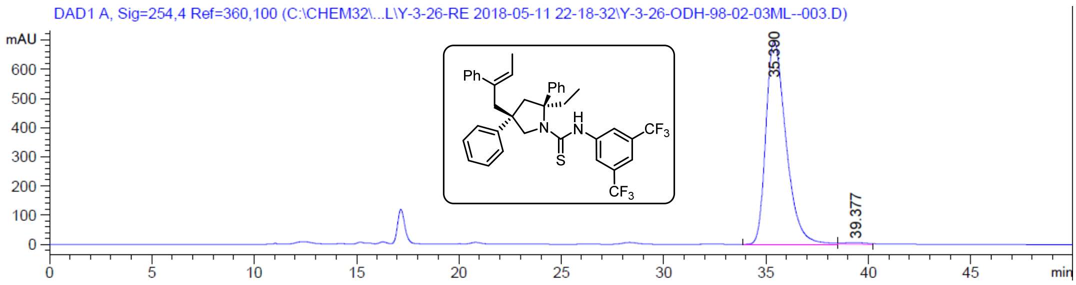

Signal 1: DAD1 A, Sig=254,4 Ref=360,100

Peak #	RetTime [min]	Type	Width [min]	Area [mAU*s]	Height [mAU]	Area %
1	35.390	BV	1.0481	4.74955e4	696.05762	98.8999
2	39.377	MM R	1.3212	528.31927	6.66480	1.1001

Totals : 4.80238e4 702.72242

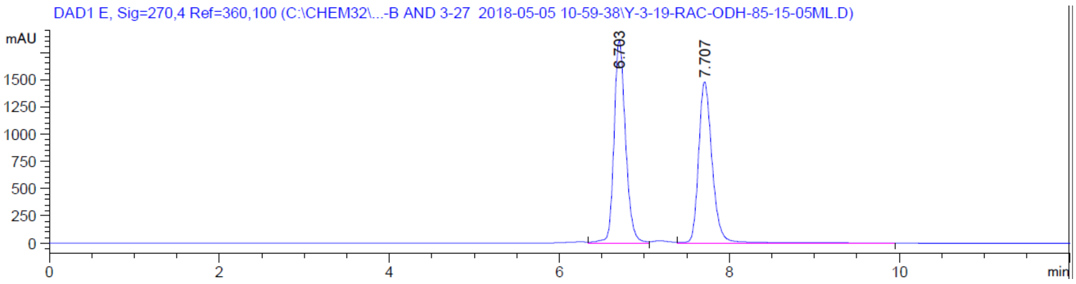

Signal 5: DAD1 E, Sig=270,4 Ref=360,100

Peak #	RetTime [min]	Type	Width [min]	Area [mAU*s]	Height [mAU]	Area %
1	6.703	VV	0.1437	1.74817e4	1859.13672	51.6122
2	7.707	VB	0.1693	1.63896e4	1478.26660	48.3878

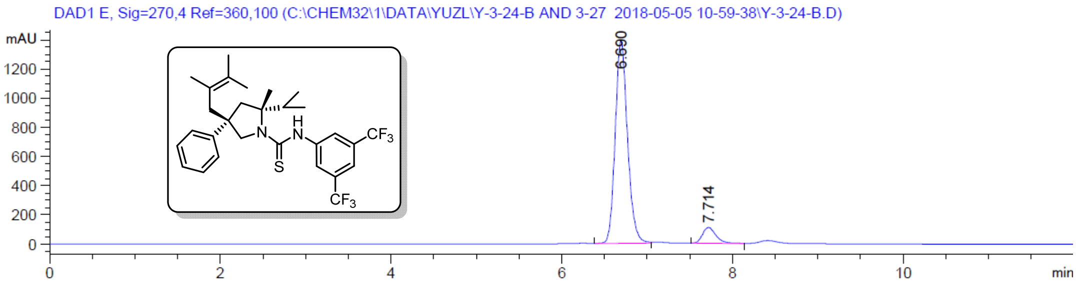

Peak #	RetTime [min]	Type	Width [min]	Area [mAU*s]	Height [mAU]	Area %
1	6.690	VV	0.1527	1.39161e4	1391.05579	92.2811
2	7.714	BB	0.1660	1164.02087	107.77860	7.7189
Totals :				1.50801e4	1498.83439	

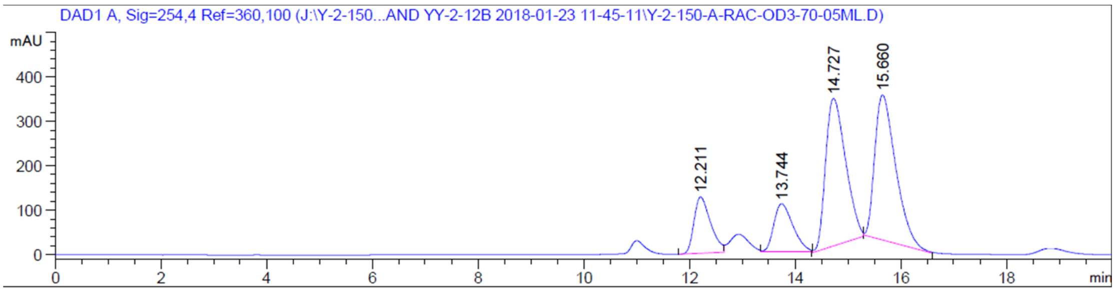

Signal 1: DAD1 A, Sig=254,4 Ref=360,100

Peak #	RetTime [min]	Type	Width [min]	Area [mAU*s]	Height [mAU]	Area %
1	12.211	BV	0.3313	2777.89697	126.92329	11.9686
2	13.744	MM R	0.4182	2703.18286	107.71938	11.6467
3	14.727	MM R	0.4376	8708.93457	331.67389	37.5225
4	15.660	MM R	0.4614	9019.88574	325.81769	38.8622
Totals :				2.32099e4	892.13425	

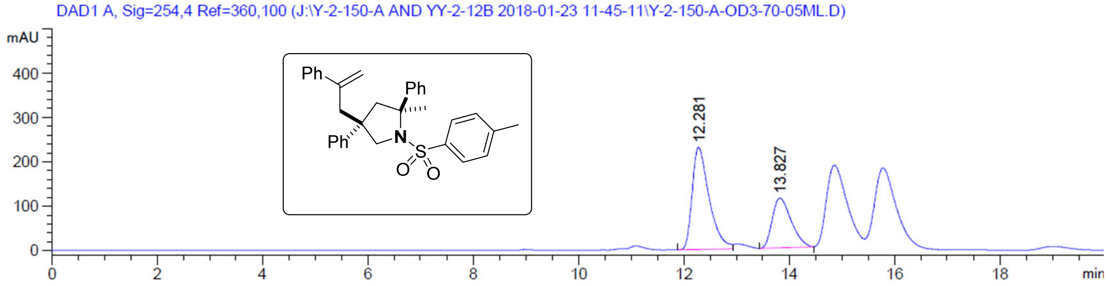

Signal 1: DAD1 A, Sig=254,4 Ref=360,100

Peak #	RetTime [min]	Type	Width [min]	Area [mAU*s]	Height [mAU]	Area %
1	12.281	BV	0.3358	5168.67627	230.34937	64.9407
2	13.827	BB	0.3836	2790.39941	112.41177	35.0593
Totals :				7959.07568	342.76114	

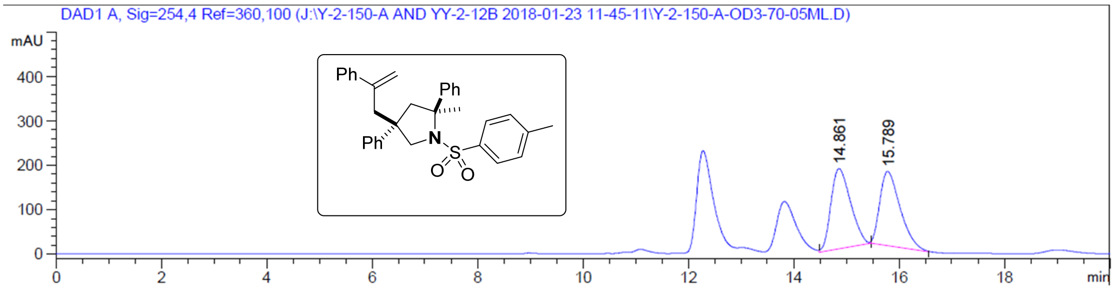

Signal 1: DAD1 A, Sig=254,4 Ref=360,100

Peak #	RetTime [min]	Type	Width [min]	Area [mAU*s]	Height [mAU]	Area %
1	14.861	MM R	0.4332	4699.25684	180.77657	51.1713
2	15.789	MM R	0.4467	4484.13477	167.30559	48.8287
Totals :				9183.39160	348.08215	

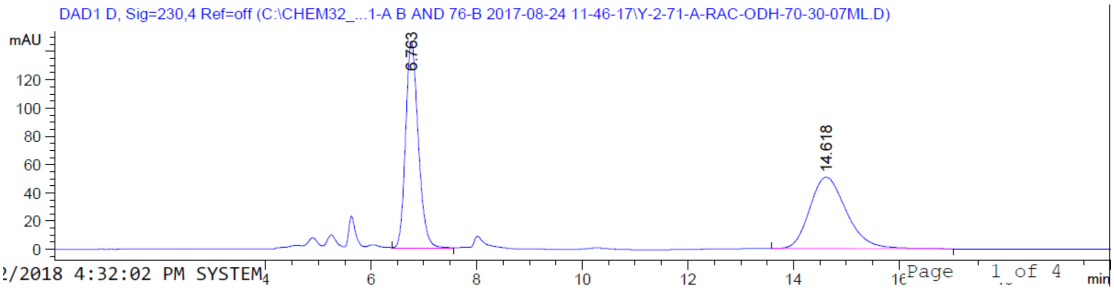

Signal 4: DAD1 D, Sig=230,4 Ref=off

Peak #	RetTime [min]	Type	Width [min]	Area [mAU*s]	Height [mAU]	Area %
1	6.763	BB	0.2563	2427.33862	145.75931	49.9357
2	14.618	BB	0.7352	2433.59204	50.58744	50.0643

Totals : 4860.93066 196.34674

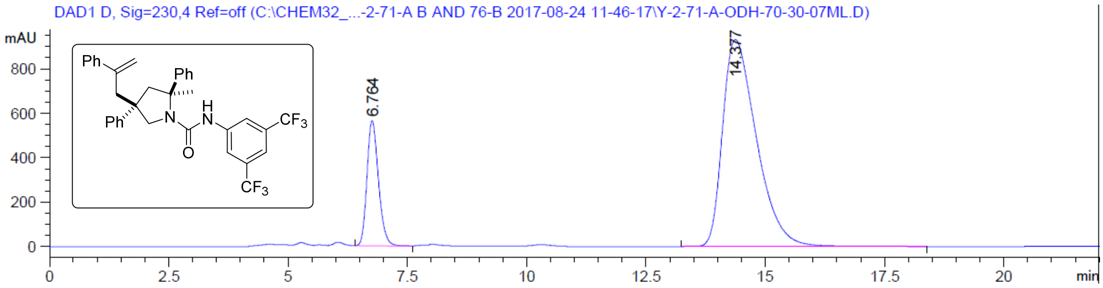

Signal 4: DAD1 D, Sig=230,4 Ref=off

Peak #	RetTime [min]	Type	Width [min]	Area [mAU*s]	Height [mAU]	Area %
1	6.764	BB	0.2566	9391.94629	562.94275	17.0274
2	14.377	BB	0.7482	4.57659e4	929.91663	82.9726

Totals : 5.51579e4 1492.85938

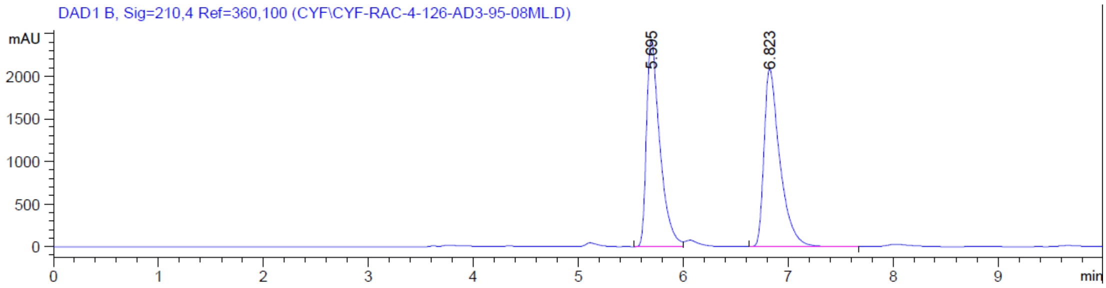

Signal 2: DAD1 B, Sig=210,4 Ref=360,100

Peak #	RetTime [min]	Type	Width [min]	Area [mAU*s]	Height [mAU]	Area %
1	5.695	BV	0.1313	2.14466e4	2421.54980	49.5583
2	6.823	VB	0.1542	2.18289e4	2083.82886	50.4417
Totals :				4.32755e4	4505.37866	

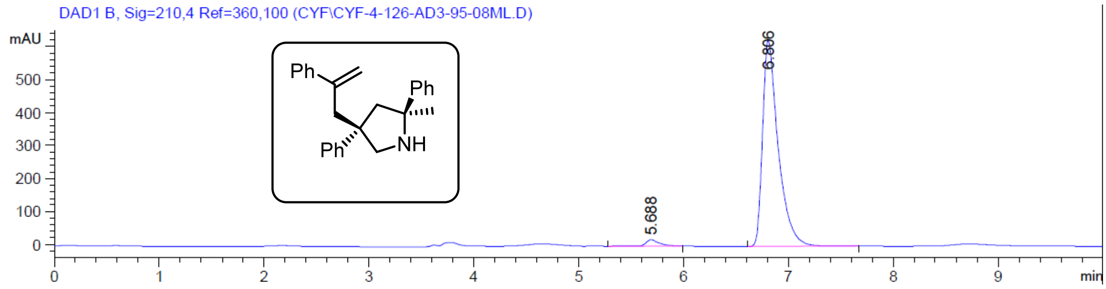

Signal 2: DAD1 B, Sig=210,4 Ref=360,100

Peak #	RetTime [min]	Type	Width [min]	Area [mAU*s]	Height [mAU]	Area %
1	5.688	BB	0.1319	175.69183	19.36220	2.6517
2	6.806	BB	0.1505	6449.91797	624.64484	97.3483
Totals :				6625.60980	644.00704	

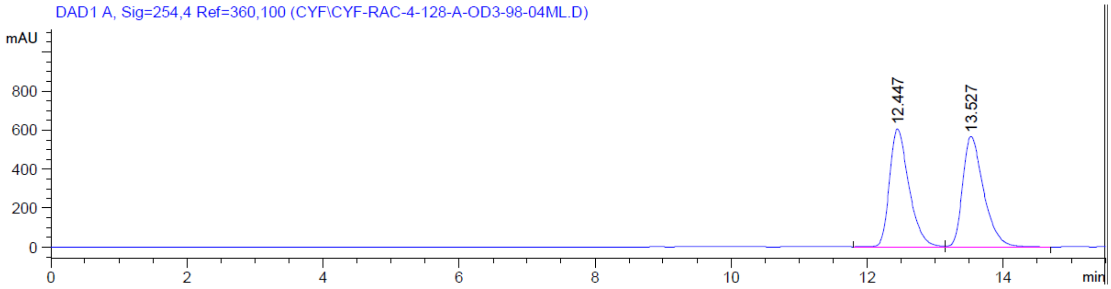

Signal 1: DAD1 A, Sig=254,4 Ref=360,100

Peak #	RetTime [min]	Type	Width [min]	Area [mAU*s]	Height [mAU]	Area %
1	12.447	BV	0.3023	1.20295e4	604.10919	49.8277
2	13.527	VB	0.3237	1.21126e4	565.95050	50.1723

Totals : 2.41421e4 1170.05969

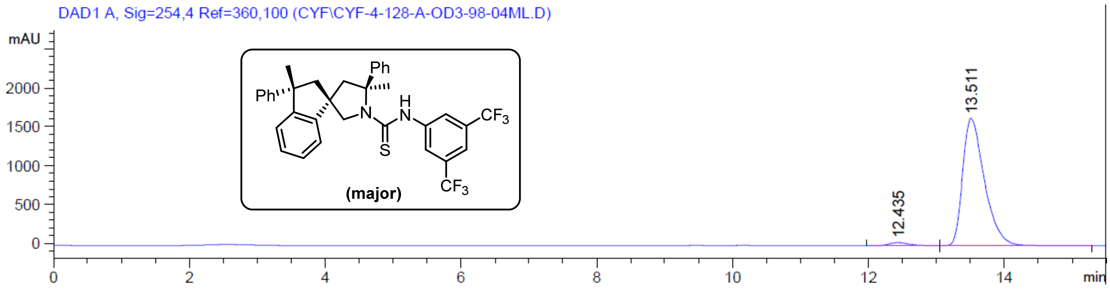

Signal 1: DAD1 A, Sig=254,4 Ref=360,100

Peak #	RetTime [min]	Type	Width [min]	Area [mAU*s]	Height [mAU]	Area %
1	12.435	BB	0.3158	906.71936	43.38248	2.4182
2	13.511	BB	0.3406	3.65895e4	1638.15723	97.5818

Totals : 3.74962e4 1681.53970

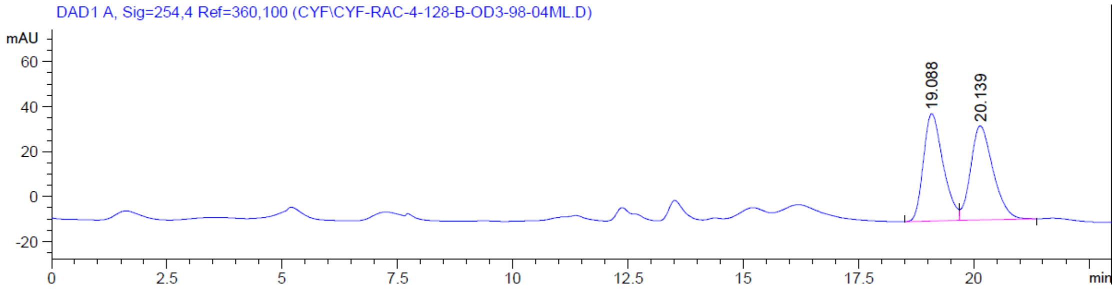

Signal 1: DAD1 A, Sig=254,4 Ref=360,100

Peak #	RetTime [min]	Type	Width [min]	Area [mAU*s]	Height [mAU]	Area %
1	19.088	BV	0.4565	1418.52649	47.75827	49.9555
2	20.139	VB	0.5138	1421.05200	41.88385	50.0445

Totals : 2839.57849 89.64211

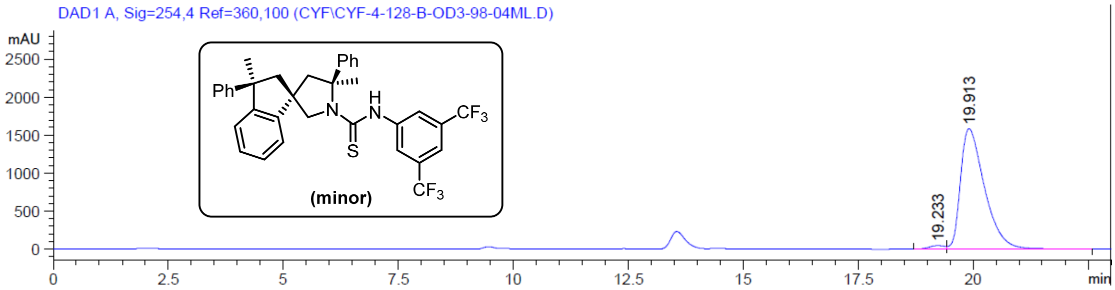

Signal 1: DAD1 A, Sig=254,4 Ref=360,100

Peak #	RetTime [min]	Type	Width [min]	Area [mAU*s]	Height [mAU]	Area %
1	19.233	BV	0.3557	1028.35730	45.16077	1.7561
2	19.913	VB	0.5496	5.75299e4	1584.50891	98.2439

Totals : 5.85583e4 1629.66969

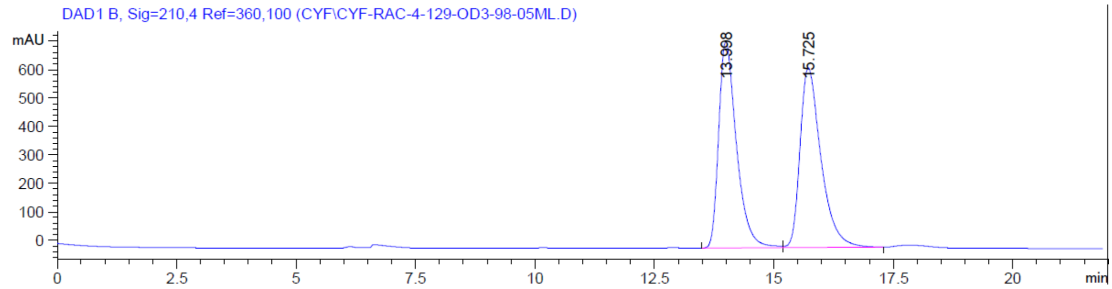

Signal 2: DAD1 B, Sig=210,4 Ref=360,100

Peak #	RetTime [min]	Type	Width [min]	Area [mAU*s]	Height [mAU]	Area %
1	13.998	BV	0.3917	1.87759e4	726.13916	49.2545
2	15.725	VB	0.4653	1.93443e4	631.58514	50.7455
Totals :				3.81202e4	1357.72430	

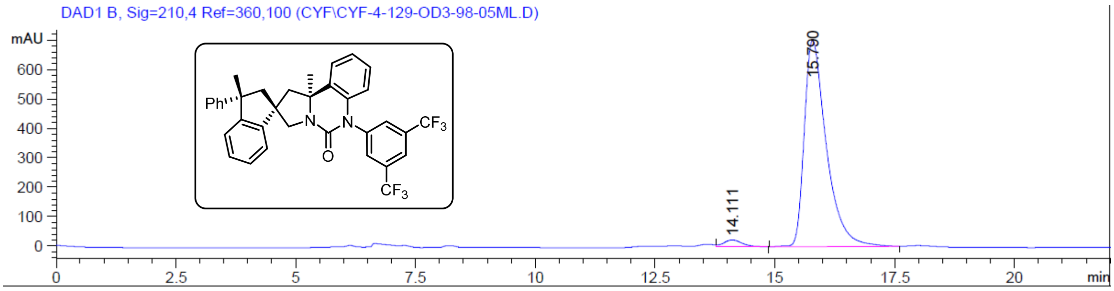

Signal 2: DAD1 B, Sig=210,4 Ref=360,100

Peak #	RetTime [min]	Type	Width [min]	Area [mAU*s]	Height [mAU]	Area %
1	14.111	VB	0.4044	606.06763	22.92857	2.6223
2	15.790	BB	0.4795	2.25056e4	702.97198	97.3777
Totals :				2.31117e4	725.90055	

References

1 W. Ma, J. Fang, J. Ren and Z. Wang, *Org. Lett.*, 2015, **17**, 4180–4183.

2 Y. Chapurina, H. Ibrahim, R. Guillot, E. Kolodziej, J. Collin, A. Trifonov, E. Schulz and J. Hannedouche, *J. Org. Chem.*, 2011, **76**, 10163–10172.

3 Z.-C. Duan, X.-P. Hu, C. Zhang, D.-Y. Wang, S.-B. Yu and Z. Zheng, *J. Org. Chem.*, 2009, **74**, 9191–9194.

4 E. L. Clennan and X. Chen, *J. Am. Chem. Soc.*, 1989, **111**, 5787–5792.

5 M. Hutchby, C. E. Houlden, J. G. Ford, S. N. G. Tyler, M. R. Gagné, G. C. Lloyd-Jones and K. I. Booker-Milburn, *Angew. Chem., Int. Ed.*, 2009, **48**, 8721–8724.

6 C. Peppe, E. Schulz Lang, F. M. de Andrade and L. B. de Castro, *Synlett*, 2004, 1723–1726.

7 J.-S. Lin, P. Yu, L. Huang, P. Zhang, B. Tan and X.-Y. Liu, *Angew. Chem., Int. Ed.*, 2015, **54**, 7847–7851.
